# A Riboflavin‐Derived Flavinium Salt Mediates Chemoselective Methylation Reactions

**DOI:** 10.1002/chem.202503590

**Published:** 2025-12-21

**Authors:** Tim Langschwager, Ekrem Suylu, Julian Zuber, Golo Storch

**Affiliations:** ^1^ Technical University of Munich (TUM) Garching Germany; ^2^ Department of Chemistry Johannes Gutenberg University Mainz (JGU) Mainz Germany

**Keywords:** chemoselective functionalization, flavin Chemistry, methylation, organic cofactors, redox condensation

## Abstract

Selective methylation is among the most relevant transformations in synthetic chemistry and the discovery of new drug molecules. A methyl group is typically installed using strong electrophiles such as methyl iodide or dimethyl sulfate, which are associated with safety hazards and limited chemoselectivity. A promising strategy for circumventing these limitations relies on splitting the methylation into a two‐step procedure under mediator control. However, such reactions, including the Mukaiyama redox condensation, currently lack applicability since the mediator is lost as organic waste, resulting in a low atom economy. We have developed a flavin‐mediated methylation strategy with easily accessible methyl diphenylphosphinite (Ph_2_POMe) as the source of the methyl group. The flavin mediator is easily recovered by a simple acidic treatment followed by oxidation with air. Detailed NMR spectroscopic studies and structural information by X‐ray crystallography paint a clear mechanistic picture, while we show the chemoselective modification of a variety of organic substrates and also demonstrate trideuteromethylation. Methylation is accomplished with complex molecules, including venetoclax and nevirapine. We envision the flavin‐mediated methodology to be adaptable to other alkylation reactions besides methylation. Within the realm of the latter, chemoselective modification of nucleobases stands out as a promising target.

## Graphical abstract

A two‐component methylation protocol was developed, where the active flavin mediator can be recycled in a single step. Crystallographic data and NMR spectroscopy support the mechanistic proposal, while chemoselective methylation was observed for a broad range of biologically relevant substrates.

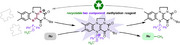

## 1 Introduction

**FIGURE 1:**
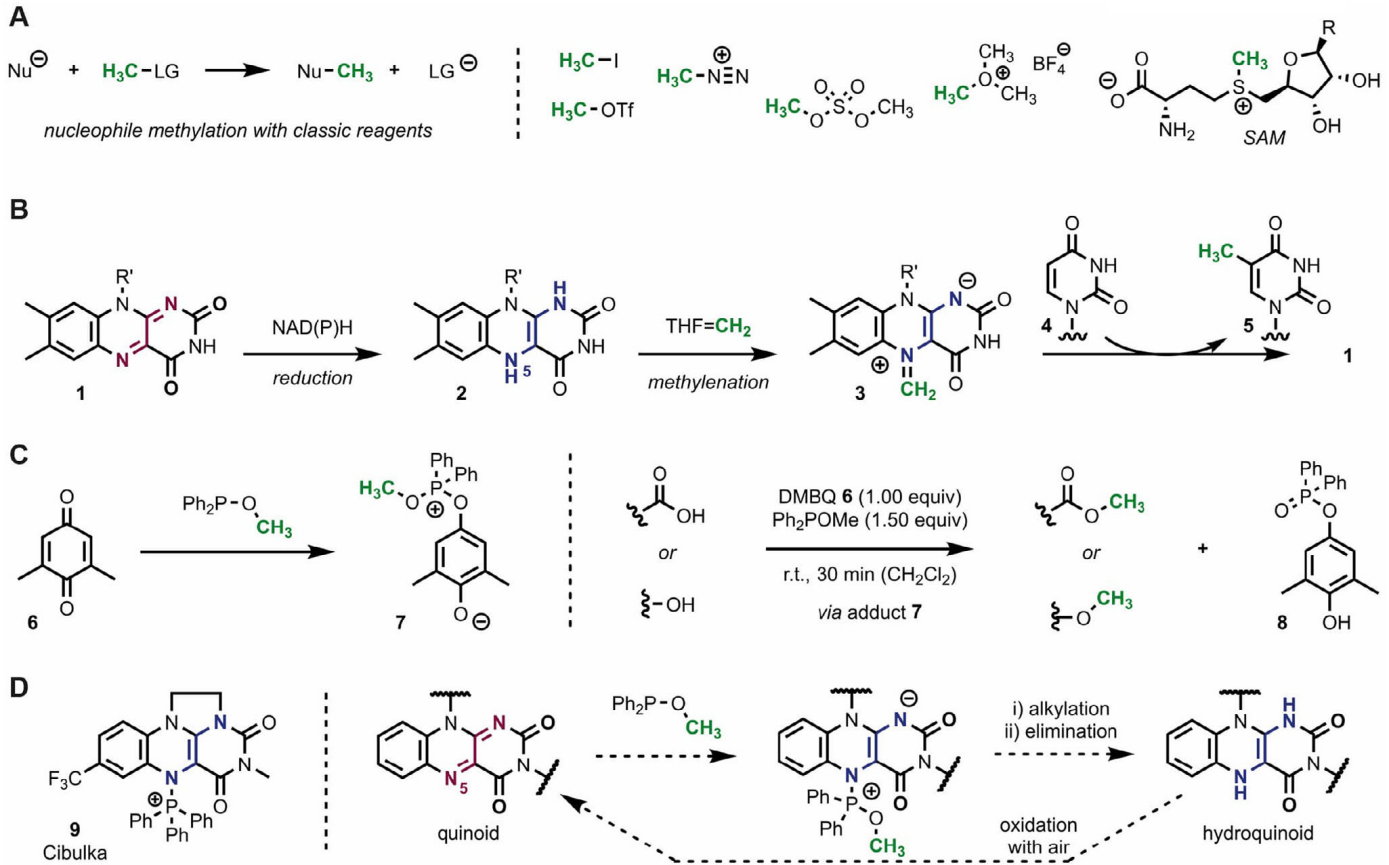
Overview of selected methylation strategies. (A) Classic methylation reagents in synthesis and in biology. Nu = nucleophile; LG = leaving group; R = 6‐aminopurinyl. (B) Methylation with flavin‐dependent enzymes via a two‐step mechanism. R’ = Ribityl‐adenosine diphosphate. (C) The Mukaiyama redox condensation. (D) Our conceptual idea of a flavin‐mediated methylation with regeneration of the mediator.

Alkylation reactions are broadly applied transformations in organic chemistry. Methylation is a prominent case amongst this reaction class with widespread applications in pharmaceutical chemistry. Known as the magic methyl effect, interest in this particular modification stems from the often substantial increase in biological activity by seemingly simple addition of a CH_3_ group [1, 2]. Several methodologies for methylation have been developed, including reductive alkylation [3], transition‐metal protocols [4, 5], and photochemical approaches [6]. While a methyl group can also be installed by using a methyl nucleophile such as methyl lithium, electrophilic methylation is most broadly applicable since heteroatoms and carbanions are typical positions for desired functionalization. However, these reactions require strong electrophiles, which are prone to unselective reactions and pose significant health risks (Figure 1A). Therefore, we took the opportunity to explore orthogonal methylation reactions that circumvent these limitations.

The archetypal methylation reaction in nature—alkylation with *S*‐adenosyl methionine (SAM) [7, 8]—also follows the paradigm outlined above, since a strong sulfonium electrophile serves as the active species. In contrast, flavin‐dependent methyltransferases enable alkylation by an intriguing combination of two independent reaction steps (Figure 1B). The quinoid flavin cofactor (**1**) is initially reduced to the hydroquinone **2**, and 5,10‐methylenetetrahydrofolate (THF = CH_2_) transfers its methylene unit to the flavin N5 position, which leads to electrophilic iminium adduct **3** [9, 10, 11]. In the case of *thymidylate synthase* ThyX, the enzymatic substrate deoxyuridine monophosphate (dUMP, **4**) then attacks with its nucleophilic position, and the methylated analog **5** is formed together with quinoid flavin **1** [12].

In organic synthesis, the Mukaiyama redox condensation also segments the alkylation reaction into two separate steps [13, 14]. The transformation relies on a phosphinite reagent such as methyl diphenylphosphinite (Ph_2_POMe), which undergoes nucleophilic attack on the electrophilic 2,6‐dimethylbenzoquinone (DMBQ, **6**) and generates the active alkylating agent **7** (Figure 1C) [15, 16]. The strategy allows turning two relatively benign reagents into a reactive species *in situ*, and subsequent alkylation typically of oxygen heteroatoms. While elegant from a conceptual standpoint, the benzoquinone is not regenerated and ultimately collected as organic waste in the form of hydroquinone **8**. Similar to the Mukaiyama redox condensation, Wang et al. broadened the scope of this reaction by exchanging DMBQ with ethyl acrylate, which, however, still led to the stoichiometric production of phosphinoxide byproducts [17].

Building on the general similarity between benzoquinones and flavins, we hypothesized that the latter might also react with phosphinites, leading to active methylating species. While several reversible adduct formations of flavins are known in enzymes and model systems [18, 19, 20, 21], only the reversible addition of phosphines to modified synthetic flavins was reported by Cibulka [22, 23]. Their results document that triphenylphosphine readily adds to cationic flavinium salts [24], leading to N5 adduct **9**. If possible with phosphinites, this would ultimately lead to an electrophilic species similar to the one formed in the Mukaiyama redox condensation (Figure 1D). Regeneration of the initial quinoid flavin would then require elimination and oxidation of the hydroquinoid form—two steps that are well‐explored and feasible with organic flavin cofactors.

## 2 Results and Discussion

**FIGURE 2:**
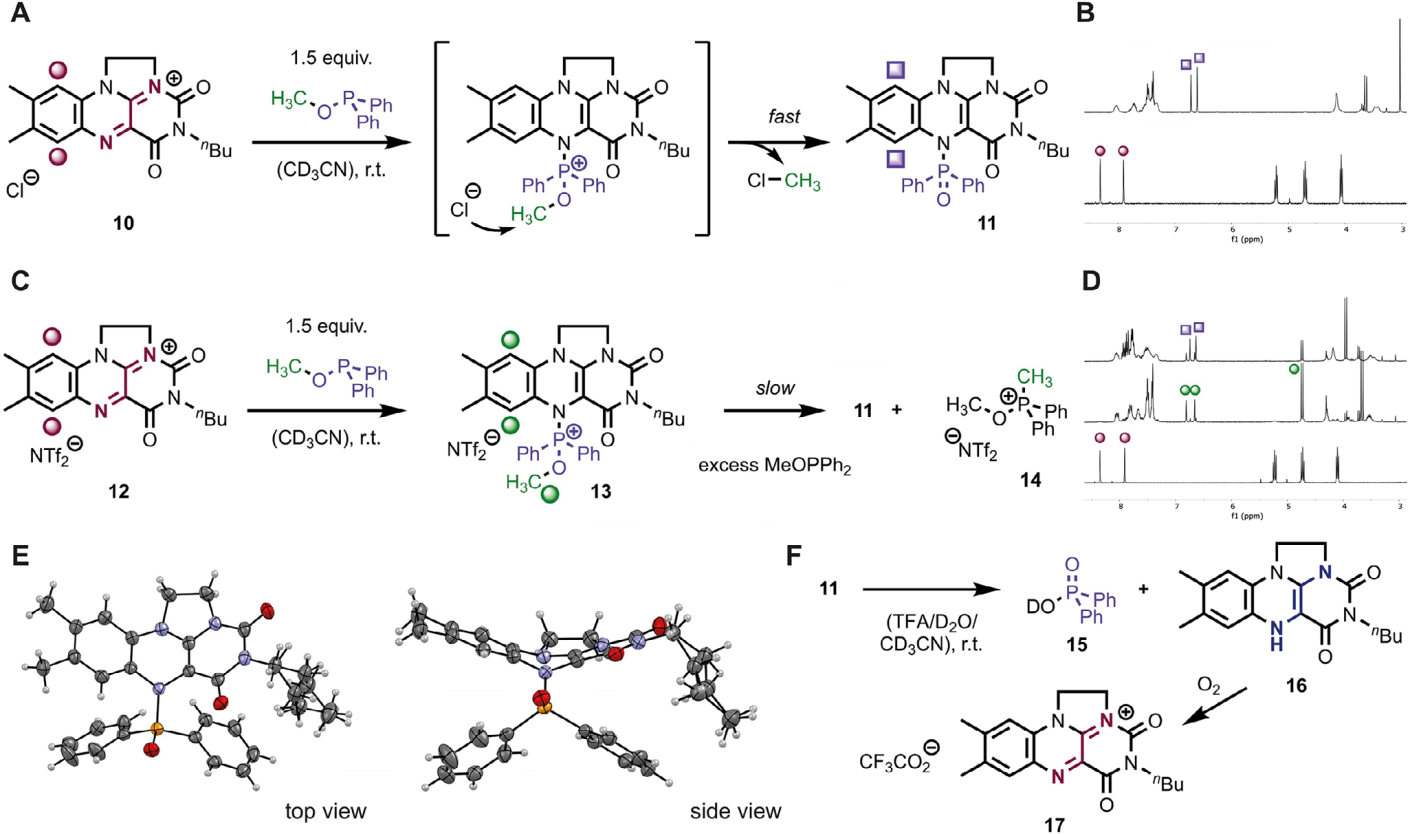
Reaction of flavinium salts with methyl diphenylphosphinite. (A) Reaction scheme in deuterated acetonitrile. (B) Comparison of the proton NMR spectra. (C) The analogous reaction with the NTf_2_ salt. (D) NMR spectra of formation and slow conversion of the active electrophile **13**. (E) Molecular structure of adduct **11** with a disordered butyl group obtained by single‐crystal X‐ray diffraction. (F) Reactivity of adduct **11** under acidic conditions.

Our initial interest was centered around the so far unexplored reactivity of flavinium salt **10** with phosphinites. The flavinium salt was prepared by a modified literature procedure in only four steps from (–)‐riboflavin (see Supporting Information for details) [24, 25]. This compound has improved solubility properties and is accessible without a single chromatographic purification step. In a promising first experiment, we directly observed a clean reaction with methyl diphenylphosphinite in deuterated acetonitrile solution (Figure 2A). Analysis of the NMR spectra (Figure 2B) revealed that the sole flavin‐containing product is P‐adduct **11**, which was identified in combination with chloromethane in solution (^1^H NMR: *δ* = 3.03 ppm) [26]. The latter gaseous compound was presumably observed in solution since these experiments were conducted in Teflon screw‐cap NMR tubes. While unproductive for the desired substrate methylation, this result already confirmed that the intermediate is an activated methylation agent that transfers the methyl group to the moderately nucleophilic chloride anion. A logical step toward circumventing this undesired anion methylation was to use flavinium bistriflimide **12** in the analogous reaction (Figure 2C). This compound was prepared in a single step by treating chloride **10** with AgNTf_2_ in 88% yield (see Supporting Information for details). Indeed, these conditions allowed characterization of the immediate adduct **13** in solution by proton NMR spectroscopy and high‐resolution ESI‐MS (Figure 2D and Supporting Information). A ^3^ *J*_H‐P_ coupling constant of 12.0 Hz was observed between the protons at the methyl group and the phosphorous atom, indicative of an intact C─O bond. No reaction with the bistriflimide anion was observed. However, prolonged reaction times led to methylation of the excess methyl diphenylphosphinite and generation of salt **14**. Having confirmed the formation and reactivity of the immediate adduct **13**, we next focused on the neutral adduct **11**. While we initially suspected the compound to be sensitive toward moisture and air due to its structural relation to hydroquinoid flavins, it turned out to be relatively stable and could even be isolated as a faint‐orange solid. Single‐crystal analysis (Figure 2E) confirmed the connectivity and the new P─N bond at the flavin N5 position. The side view clearly indicates the bent flavin geometry, indicative of the reduced state. To the best of our knowledge, this is the first single‐crystal structure of a flavin adduct with heteroatom substitution at the N5 position. While stable under neutral conditions, we found that subjecting adduct **11** to trifluoroacetic acid (TFA) in deuterated acetonitrile and deuterated water leads to conversion. We observed hydrolysis of the adduct concomitant with an oxidation of the formed reduced flavin by air (Figure 2F). These results already indicate that the flavinium salt **12** might be recyclable from adduct **11**.

**FIGURE 3:**
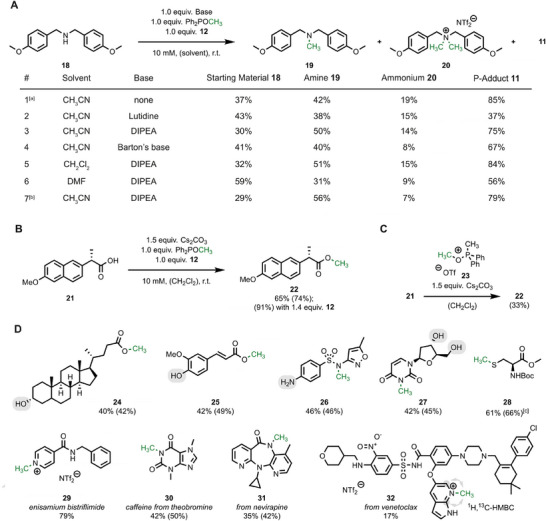
Methylation reaction with flavinium salt **12** and Ph_2_POMe. (A) Secondary amine substrate and reaction screening. Percentages refer to yields determined by NMR spectroscopy with an internal standard. (B) Esterification of (*S*)‐naproxen using the flavin methodology. (C) Probing for the alkylation ability of salt **23** in the esterification reaction. (D) Scope of the selective methylation reaction. Yields in parentheses refer to NMR experiments with an internal standard, all other values correspond to isolated material. [a] Reaction set up at −30 °C and warmed up to room temperature (r.t.). [b] Adduct **13** was added by syringe pump over a period of 30 min. Yield of isolated material of amine **19** is 46%. [c]: Here, 1.5 equiv. phosphinite were used.

We then explored the activity of phosphinite adduct **13** in alkylation reactions with organic substrates. The secondary amine **18** served as an informative model substrate since several challenges arise here with alkylation reactions, including overalkylation to the ammonium salt. The flavinium bistriflimide **12** was chosen, activated with commercially available Ph_2_POCH_3_, and combined in equimolar ratio with amine **18** (Figure 3A; for a full screening of conditions, see Supporting Information). Indeed, we observed substrate methylation to tertiary amine **19** as the major product, but overalkylation to ammonium salt **20** occurred with considerable amounts (Entry 1). Since the net reaction includes formation of an HNTf_2_ equivalent, we hypothesized that the slightly more basic secondary amine substrate might be protonated predominantly, hindering its activity as a nucleophile. Following this rationale, methylation of the tertiary amine **19** is preferred. A quick screen of several bases (Entries 2 – 4) confirmed that selectivity of the methylation was improved under these conditions, with DIPEA being a good compromise of reactivity and selectivity. Dichloromethane as solvent performed equally well, while DMF provided decreased levels of conversion (Entries 5 and 6). Slow addition of the activated flavin adduct over a period of 30 min turned out to be beneficial and led to 56% yield of the tertiary amine, while only 7% of the ammonium salt were formed (Entry 7). The flavin‐mediated methylation protocol is not limited to amine nucleophiles, and slight adaptations in solvent and base led to a straightforward method for methylation of (*S*)‐naproxen **21** to the corresponding methyl ester **22** (Figure 3B). An even higher yield of 91% was observed with a small excess of flavin **12** (0.4 equiv.) and phosphinite. We also studied the possibility that the methylated phosphinite **14** might serve as an additional methylating agent (Figure 3C). While competent under isolated conditions (tested with triflate **23**), the contribution of this pathway in the flavin‐mediated reaction is marginal since only one equivalent of Ph_2_POMe is used, and 79% of flavin adduct **11** was observed (c.f., Figure 3A Entry 7).

Having established an easily adaptable methodology, we investigated the substrate scope with increasing levels of complexity (Figure 3D). Carboxylic acids were smoothly methylated in the presence of alcohols and phenols, as exemplified by the esters of lithocholic acid (**24**) and ferulic acid (**25**). Phenolates and carboxylates usually possess similar nucleophilicity (*N*  =  16.45 for benzoate in MeCN and *N*  =  18.53 for phenolate in MeCN) [27, 28]. Accordingly, product mixtures were obtained from ferulic acid with Ph_2_POCH_3_ and ethyl acrylate (see Supporting Information for details) [17]. Selective methylation was observed for the antibiotic drug sulfamethoxazole, where the aniline did not react, and sole methylation of the sulfonamide was obtained (**26**). Similarly, methylation of the imide in desoxyuridine (**27**) proceeded without any concurrent methylation of the oxygen nucleophiles. The thiol position in a protected cysteine (**28**) was also confirmed as a suitable nucleophile for methylation. Our mild methylation protocol also provides access to selective methylation in complex molecules. Enisamium bistriflimide (**29**) was prepared by methylation of the pyridine nitrogen position in 79% yield, and caffeine (**30**) resulted in 50% yield from methylation of theobromine. Despite the presence of four competing nucleophilic positions, methylation of the HIV drug nevirapine occurred with high site‐selectivity and led to analog **31** in a yield of 42%. Venetoclax, a drug molecule for treating leukemia, was also subjected to our flavin‐mediated methylation protocol, which led to the isolation of derivative **32**. These reactions clearly highlight that the combination of flavinium salts and phosphinites is beneficial for selective methylation under mild conditions.

**FIGURE 4:**
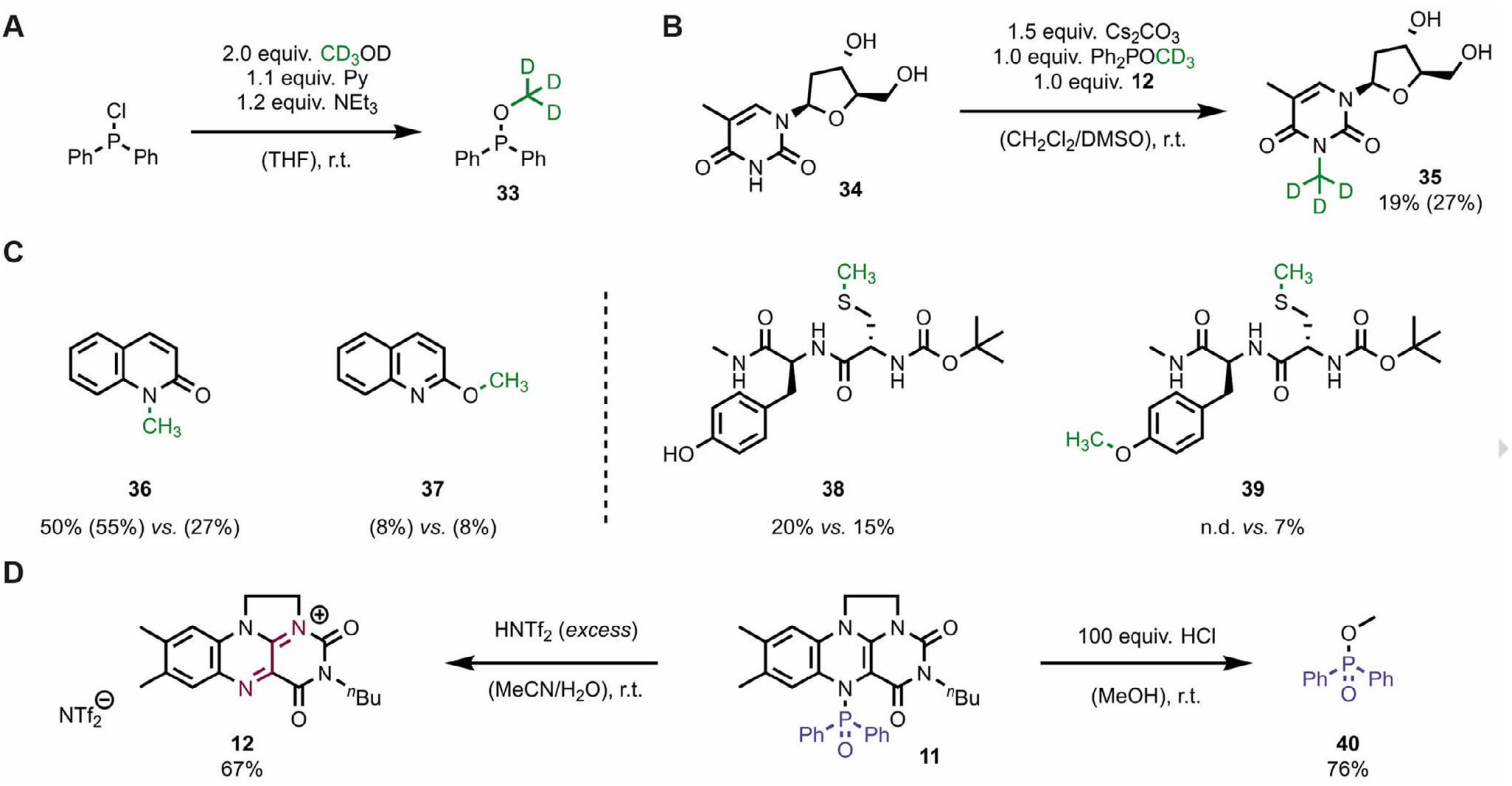
Application of the flavin‐mediated methylation protocol. (A) Preparation of trideuterated phosphinite **33**. (B) Trideuteromethylation of thymidine **34**. (C) Comparative reactivity of the flavin methylation protocol (left entries) *versus* methyl triflate (right entries). Conditions: 1.0 equiv. methyl triflate. For conditions of the flavin‐mediated reaction, see Figure 3 and Supporting Information. Yields in parentheses were determined by NMR spectroscopy relative to an internal standard. All other values refer to isolated material. (D) Recycling of the flavinium mediator under acidic reaction conditions.

The broad applicability of our flavin‐mediated methylation methodology was then explored in detail. Trideuteromethylation was identified as a target reaction given its relevance in drug discovery [29, 30]. Several trideuteromethyl‐containing drug candidates are currently being developed or are approved by the FDA [31], including the tyrosine kinase 2 inhibitor deucravacitinib [32]. As an advantage of our strategy, the required trideuteromethyl diphenylphosphinite **33** is easily prepared from chlorodiphenylphosphine and deuterated methanol in a single step (Figure 4A) [17]. With this reagent in hand, the selective trideuteromethylation of thymidine **34** (Figure 4B) led to the trideutero‐analogue of 3‐meT **35**, which is recognized by the fat mass and obesity‐associated (FTO) gene [33]. Inspired by the observed high selectivity of one specific methylation site throughout our study, we also probed difficult selectivity challenges [34]. While classic methylation agents such as methyl triflate only moderately distinguish (3.4 [**36**]: 1 [**37**]) between *N*‐ and *O*‐alkylation of quinolone, the reaction was both more efficient and more selective (6.9 [**36**]: 1 [**37**]) with our flavin‐mediated method (Figure 4C). The observation that phenolic positions are not alkylated (c.f., Figure 3D) encouraged us to test dipeptides containing tyrosine and cysteine. With methyl triflate, we observed cysteine methylation (**38**) and overalkylation at both positions (**39**). In contrast, the flavin‐mediated strategy did not lead to overalkylation (see Supporting Information for details). Besides selectivity, the recyclability of the flavin mediator was identified as a key prerequisite for an efficient protocol. Building on our initial observation that adduct **11** is unstable under acidic conditions, we tested the addition of bistriflimidic acid (HNTf_2_) for selective hydrolysis (Figure 4D). Indeed, flavinium salt **12** was isolated in 67% under these conditions, which implies that no ion exchange is necessary before application in the next reaction. In methanolic solution, acidic treatment leads to flavin recovery and the corresponding methyl diphenylphosphinate **40**, a useful compound *inter alia* as a sensitizer [35].

## 3 Conclusion

Our study shows that flavinium salts derived from naturally occurring (–)‐riboflavin are competent mediators in redox condensation methylations with organic substrates. We observed high chemoselectivity in several cases, typically avoiding any reaction at the alcohol and phenol positions. These characteristics are expected to serve as beneficial properties for achieving selective functionalization of complex molecules, including biologically active compounds. In this context, the facile incorporation of trideuteromethyl groups likely adds to the synthetic usefulness. Contrary to several existing strategies, the simple recovery of the flavin mediator through acidic workup and oxidation by air also adds to the applicability of our protocol. In addition to methylation, we envision the flavin‐mediated strategy to be equally effective in other group transfer reactions if different phosphinites are applied. With other phosphorous nucleophiles, we expect similar flavin‐mediated reactions to unlock completely new transformations.

## Conflicts of Interest

The authors declare no conflict of interest.

## Supporting information



The data that support the findings of this study are available in the supplementary material of this article. Deposition number 2496823 (for **11**) contains the supplementary crystallographic data for this paper. This data is provided free of charge by the joint Cambridge Crystallographic Data Centre and Fachinformationszentrum Karlsruhe Access Structures service. The authors have cited additional references within the Supporting Information [36, 37, 38, 39, 40, 41, 42, 43, 44, 45, 46, 47, 48, 49, 50, 51, 52, 53, 54, 55, 56, 57, 58, 59].

**Supporting file 2**: chem70586‐sup‐0002‐DataFile‐ee.zip

**Supporting file 3**: chem70586‐sup‐0003‐DataFile‐ff.zip

## Data Availability

The data that support the findings of this study are available in the supplementary material of this article.

## References

[1] H. Schönherr and T. Cernak , “Profound Methyl Effects in Drug Discovery and a Call for New C–H Methylation Reactions” Angewandte Chemie International Edition 52 (2013): 12256–12267, 10.1002/anie.201303207. 24151256

[2] E. J. Barreiro , A. E. Kümmerle , and C. A. M. Fraga , “The Methylation Effect in Medicinal Chemistry,” Chemical Reviews 111 (2011): 5215–5246, 10.1021/cr200060g. 21631125

[3] A. A. Boezio , J. Pytkowicz , A. Côté , and A. B. Charette , “Asymmetric, Catalytic Synthesis of α‐Chiral Amines Using a Novel Bis(phosphine) Monoxide Chiral Ligand,” Journal of the American Chemical Society 125 (2003): 14260–14261, 10.1021/ja038291+. 14624558

[4] T. T. Dang , B. Ramalingam , and A. M. Seayad , “Efficient Ruthenium‐Catalyzed N‐Methylation of Amines Using Methanol,” ACS Catalysis 5 (2015): 4082–4088, 10.1021/acscatal.5b00606.

[5] M. Giedyk , K. Goliszewskaab , and D. Gryko , “Vitamin B_12_ Catalysed Reactions” Chemical Society Reviews 44 (2015): 3391–3404, 10.1039/C5CS00165J. 25945462

[6] V. N. Tsarev , Y. Morioka , J. Caner , et al., “N‐Methylation of Amines With Methanol at Room Temperature,” Organic Letters 17 (2015): 2530–2533, 10.1021/acs.orglett.5b01063. 25915546

[7] M. R. Bennett , S. A. Shepherd , V. A. Cronin , and J. Micklefield , “Recent Advances in Methyltransferase Biocatalysis,” Current Opinion in Chemical Biology 37 (2017): 97–106, 10.1016/j.cbpa.2017.01.020. 28259085

[8] E. Abdelraheem , B. Thair , R. F. Varela , et al., “Methyltransferases: Functions and Applications” ChemBioChem 23 (2022): e202200212, 10.1002/cbic.202200212. 35691829 PMC9539859

[9] C. Bou‐Nader , D. Cornu , V. Guerineau , T. Fogeron , M. Fontecave , and D. Hamdane , “Enzyme Activation with a Synthetic Catalytic Co‐enzyme Intermediate: Nucleotide Methylation by Flavoenzymes,” Angewandte Chemie International Edition 56 (2017): 12523–12527, 10.1002/anie.201706219. 28796306

[10] C. Bou‐Nader , F. W. Stull , L. Pecqueur , et al., “An Enzymatic Activation of Formaldehyde for Nucleotide Methylation,” Nature Communications 12 (2021): 4542, 10.1038/s41467-021-24756-8. PMC8316439 34315871

[11] D. Hamdane , M. Argentini , D. Cornu , B. Golinelli‐Pimpaneau , and M. Fontecave , “FAD/Folate‐Dependent tRNA Methyltransferase: Flavin as a New Methyl‐Transfer Agent,” Journal of the American Chemical Society 134 (2012): 19739–19745, 10.1021/ja308145p. 23157377

[12] T. V. Mishanina , L. Yu , K. Karunaratne , et al., “An Unprecedented Mechanism Of Nucleotide Methylation in Organisms Containing *thyX* ,” Science 351 (2016): 507–510, 10.1126/science.aad0300. 26823429 PMC4744818

[13] T. Mukaiyama , “Explorations into New Reaction Chemistry,” Angewandte Chemie International Edition 43 (2004): 5590–5614, 10.1002/anie.200300641. 15470679

[14] P.‐H. Chen and S. Bloom , “Divergent Synthesis of ΔAA‐Peptides Using a Bioorthogonal Pro‐Amino Acid and Aqueous Flavin Photocatalyst: Green Light Enhances Catalyst Performance and Product Selectivity,” A Sequence of Radical Addition and Reduction was Reported with Flavin Photocatalysis 64 (2025): e202511832, 10.1002/anie.202511832. PMC13092294 40842206

[15] T. Mukaiyama , T. Shintou , and K. Fukumoto , “A Convenient Method for the Preparation of Inverted *Tert*‐Alkyl Carboxylates from Chiral *Tert*‐Alcohols by a New Type of Oxidation−Reduction Condensation Using 2,6‐Dimethyl‐1,4‐benzoquinone” Journal of the American Chemical Society 125 (2003): 10538–10539, 10.1021/ja0303844. 12940734

[16] T. Shintou and T. Mukaiyama , “Efficient Methods for the Preparation of Alkyl−Aryl and Symmetrical or Unsymmetrical Dialkyl Ethers Between Alcohols and Phenols or Two Alcohols by Oxidation−Reduction Condensation” Journal of the American Chemical Society 126 (2004): 7359–7367, 10.1021/ja0487877. 15186175

[17] J.‐X. Wang , M.‐Q. Chen , Y. Zhang , et al., “A Modified Arbuzov‐Michalis Reaction for Selective Alkylation of Nucleophiles,” Angewandte Chemie International Edition 64 (2024): e202409931, 10.1002/anie.202409931. 38957113

[18] G. B. Michaels , J. T. Davidson , and H. D. Peck , “A Flavin‐sulfite Adduct as an Intermediate in the Reaction Catalyzed by Adenylyl Sulfate Reductase from Desulfovibriovulgaris,” Biochemical and Biophysical Research Communications 39 (1970): 321–328, 10.1016/0006-291X(70)90579-6. 5421934

[19] P. F. Fitzpatrick , “Nitroalkane Oxidase: Structure and Mechanism,” Archives of Biochemistry and Biophysics 632 (2017): 41–46, 10.1016/j.abb.2017.05.012. 28529198 PMC5650508

[20] M. Soltero‐Higgin , E. E. Carlson , T. D. Gruber , and L. L. Kiessling , “A Unique Catalytic Mechanism for UDP‐galactopyranose Mutase,” Nature Structural & Molecular Biology 11 (2004): 539–543, 10.1038/nsmb772. 15133501

[21] R. Teufel , A. Miyanaga , Q. Michaudel , et al., “Flavin‐mediated Dual Oxidation Controls an Enzymatic Favorskii‐type Rearrangement,” Nature 503 (2013): 552–556, 10.1038/nature12643. 24162851 PMC3844076

[22] M. März , M. Babor , and R. Cibulka , “Flavin Catalysis Employing an N(5)‐Adduct: An Application in the Aerobic Organocatalytic Mitsunobu Reaction,” European Journal of Organic Chemistry 2019 (2019): 3264–3268, 10.1002/ejoc.201900397.

[23] E. Zubova , A. Pokluda , H. Dvořáková , M. Krupička , and R. Cibulka , “Exploring the Reactivity of Flavins with Nucleophiles Using a Theoretical and Experimental Approach,” ChemPlusChem 89 (2024): e202300547, 10.1002/cplu.202300547. 38064649

[24] T. Sakai , T. Kumoi , T. Ishikawa , T. Nitta , and H. Iida , “Comparison of Riboflavin‐derived Flavinium Salts Applied to Catalytic H_2_O_2_ Oxidations” Organic & Biomolecular Chemistry 16 (2018): 3999–4007, 10.1039/C8OB00856F. 29766194

[25] S. Murahashi , D. Zhang , H. Iida , et al., “Flavin‐catalyzed Aerobic Oxidation of Sulfides and Thiols with Formic Acid/Triethylamine,” Chemical Communications 50 (2014): 10295–10298, 10.1039/C4CC05216A. 25056359

[26] M. Bürchner , A. M. T. Erle , H. Scherer , and I. Krossing , “Synthesis and Characterization of Boranate Ionic Liquids (BILs),” Chemistry—A European Journal 18 (2012): 2254–2262, 10.1002/chem.201102460. 22267056

[27] R. J. Mayer , A. R. Ofial , H. Mayr , and C. Y. Legault , “Lewis Acidity Scale of Diaryliodonium Ions Toward Oxygen, Nitrogen, and Halogen Lewis Bases,” Journal of the American Chemical Society 142 (2020): 5221–5233, 10.1021/jacs.9b12998. 32125154

[28] R. J. Mayer , M. Breugst , N. Hampel , A. R. Ofial , and H. Mayr , “Ambident Reactivity of Phenolate Anions Revisited: A Quantitative Approach to Phenolate Reactivities,” The Journal of Organic Chemistry 84 (2019): 8837–8858, 10.1021/acs.joc.9b01485. 31241938

[29] J. Steverlynck , R. Sitdikov , and M. Rueping , “The Deuterated “Magic Methyl” Group: A Guide to Site‐Selective Trideuteromethyl Incorporation and Labeling by Using CD_3_ Reagents” Chemistry: A European Journal 27 (2021): 11751–11772, 10.1002/chem.202101179. 34076925 PMC8457246

[30] J. Atzrodt , V. Derdau , W. J. Kerr , and M. Reid , “Deuterium‐ and Tritium‐Labelled Compounds: Applications in the Life Sciences,” Angewandte Chemie International Edition 57 (2017): 1758–1784, 10.1002/anie.201704146. 28815899

[31] R. M. C. Di Martino , B. D. Maxwell , and T. Pirali , “Deuterium in Drug Discovery: Progress, Opportunities and Challenges,” Nature Reviews Drug Discovery 22 (2023): 562–584, 10.1038/s41573-023-00703-8. 37277503 PMC10241557

[32] A. Mullard , “First De Novo Deuterated Drug Poised for Approval,” Nature Reviews Drug Discovery 21 (2022): 623–625, 10.1038/d41573-022-00139-6. 35974147

[33] Z. Han , T. Niu , J. Chang , et al., “Crystal Structure of the FTO Protein Reveals Basis for Its Substrate Specificity,” Nature 464 (2010): 1205–1209, 10.1038/nature08921. 20376003

[34] L. L. Bengel , B. Aberle , A.‐N. Egler‐Kemmerer , S. Kienzle , B. Hauer , and S. C. Hammer , “Engineered Enzymes Enable Selective *N*‐Alkylation of Pyrazoles with Simple Haloalkanes” Angewandte Chemie International Edition 60 (2021): 5554–5560, 10.1002/anie.202014239. 33300646 PMC7986378

[35] G. Heinrich , M. Kondratiuk , L. J. Goossen , and M. P. Wiesenfeldt , “Rapid Reaction Optimization by Robust and Economical Quantitative Benchtop ^19^F NMR Spectroscopy” Nature Protocols 19 (2024): 1529–1556, 10.1038/s41596-023-00951-3. 38409535

[36] A. Walter , W. Eisenreich , and G. Storch , “Photochemical Desaturation and Epoxidation with Oxygen by Sequential Flavin Catalysis,” Angewandte Chemie International Edition 62 (2023): e202310634, 10.1002/anie.202310634. 37635656

[37] M. Shi and Y. Inoue , “Geometrical Photoisomerization of (Z )‐Cyclooctene Sensitized by Aromatic Phosphate, Phosphonate, Phosphinate, Phosphine Oxide and Chiral Phosphoryl Esters,” Journal of the Chemical Society, Perkin Transactions 2 (1998): 2421–2428, 10.1039/a805371e.

[38] X. Ma , X. Yan , J. Yu , et al., “Metal‐free Catalytic Nucleophilic Substitution of Primary Alcohols with Secondary Phosphine Oxides,” Green Chemistry 27 (2025): 102–108, 10.1039/D4GC04409F.

[39] K. S. Colle and E. S. Lewis , “Methoxyphosphonium Ions; Intermediates in the Arbuzov Reaction,” Journal of Organic Chemistry 43 (1978): 571–574, 10.1021/jo00398a010.

[40] A. L. Roch , M. Hébert , and A. Gagnon , “Copper‐Promoted O‐Arylation of the Phenol Side Chain of Tyrosine Using Triarylbismuthines,” European Journal of Organic Chemistry 2020 (2020): 5363–5367, 10.1002/ejoc.202000790.

[41] I. Borthakur , S. Srivastava , S. Kumari , and S. Kundu , “Tandem Synthesis of N ‐methylated Tertiary Amines Via Three‐component Coupling of Carbonyl Compounds, Amines, and Methanol, Amines, and Methanol,” Chemical Communications 58 (2022): 9822–9825, 10.1039/D2CC03115A. 35975637

[42] A. Biswas , S. Kolb , S. H. Rottger , et al., “A BOIMPY Dye Enables Multi‐Photoinduced Electron Transfer Catalysis: Reaching Super‐Reducing Properties,” Angewandte Chemie International Edition 64 (2025): e202416472, 10.1002/anie.202416472. 39655963

[43] J. García‐Méndez , A. López‐Torres , and M. A. Fernández‐Herrera , “Improved Synthesis and Characterization of Bile Acid Esters: Organogelation and Supramolecular Properties,” Steroids 214 (2025): 109560, 10.1016/j.steroids.2025.109560. 39793913

[44] L. F. Toneto Novaes , C. Martins Avila , K. J. Pelizzaro‐Rocha , et al., “(−)‐*Tarchonanthuslactone: Design of New Analogues*, Evaluation of their Antiproliferative Activity on Cancer Cell Lines, and Preliminary Mechanistic Studies,” ChemMedChem 10 (2015): 1687–1699, 10.1002/cmdc.201500246. 26305900

[45] S. Malik , U. K. Nadir , and P. S. Pandey , “Microwave‐Assisted Efficient Methylation of Alkyl and Arenesulfonamides with Trimethylsulfoxonium Iodide and KOH,” Synthetic Communications 38 (2008): 3074–3081, 10.1080/00397910802045600.

[46] X.‐Y. Jin , Y. M. He , T. H. Hui , L. Liu , and L. Cheng , “Selective Methylation of Nucleosides via an *In Situ* Generated Methyl Oxonium” Journal of Organic Chemistry 89 (2024): 3597–3604, 10.1021/acs.joc.3c02578. 38356389

[47] F. Ishikawa , N. Tsukumo , E. Morishita , et al., “Biosynthetic Diversification of Non‐ribosomal Peptides Through Activity‐based Protein Profiling of Adenylation Domains,” Chemical Communications 59 (2023): 9473–9476, 10.1039/D3CC02633G. 37477345

[48] J. Sitkowski , L. Stefaniak , L. Nicol , M. L. Martin , G. J. Martin , and G. A. Webb , “Complete Assignments of the ^1^H, ^13^C and ^15^N NMR Spectra of Caffeine” Spectrochimica Acta, Part A: Molecular and Biomolecular Spectroscopy 51 (1995): 839–841, 10.1016/0584-8539(94)00192-E.

[49] Z. Sun , F. He , Y. Xu , et al., “Intramolecular Palladium(II)‐Catalyzed Regioselective 6‐*endo* or 6‐*exo* C–H Benzannulation: An Approach for the Diversity‐Oriented Synthesis of Quinolinone Derivatives from Pyridones” Journal of Organic Chemistry 89 (2024): 7058–7064, 10.1021/acs.joc.4c00449. 38682741

[50] Y. Naganawa , K. Sakamoto , A. Fujita , et al., “One‐Step Esterification of Phosphoric, Phosphonic and Phosphinic Acids with Organosilicates: Phosphorus Chemical Recycling of Sewage Waste,” Angewandte Chemie International Edition 64 (2025): e202416487, 10.1002/anie.202416487. 39541227

[51] APEX4 Suite of Crystallographic Software, Version 2021‐10.0, Bruker AXS Inc, Madison, Wisconsin, USA, 2021.

[52] Bruker, SAINT, V8.40B, Bruker AXS Inc, Madison, Wisconsin, USA.

[53] L. Krause , R. Herbst‐Irmer , G. M. Sheldrick , and D. Stalke , “Comparison of Silver and Molybdenum Microfocus X‐ray Sources for Single‐crystal Structure Determination,” Journal of Applied Crystallography 48 (2015): 3–10, 10.1107/S1600576714022985. 26089746 PMC4453166

[54] G. M. Sheldrick , “SHELXT—Integrated Space‐group and Crystal‐structure Determination” Acta Crystallographica A71 (2015): 3–8, 10.1107/S2053273314026370. PMC4283466 25537383

[55] G. M. Sheldrick , “Crystal Structure Refinement with *SHELXL* ” Acta Crystallographica C71 (2015): 3–8, 10.1107/S2053229614024218. PMC4294323 25567568

[56] C. B. Hübschle , G. M. Sheldrick , and B. Dittrich , “ *ShelXle*: A Qt Graphical User Interface for *SHELXL* ,” Journal of Applied Crystallography 44 (2011): 1281–1284, 10.1107/S0021889811043202. 22477785 PMC3246833

[57] E. Prince , International Tables for Crystallography Volume C, Mathematical, Physical and Chemical Tables, International Union of Crystallography, Chester, England 500–502; 219–222 (2006): 193–199, 10.1107/97809553602060000103.

[58] C. R. Groom , I. J. Bruno , M. P. Lightfoot , and S. C. Ward , “The Cambridge Structural Database,” Acta Crystallographica B72 (2016): 171–179, 10.1107/S2052520616003954. PMC4822653 27048719

[59] D. Kratzert , FinalCif, V144, https://dkratzert.de/finalcif.html.

