# Supplementary material for: A Riboflavin‐Derived Flavinium Salt Mediates Chemoselective Methylation Reactions
Source: Chemistry. 2025 Dec 21;32(5):e03590. doi: 10.1002/chem.202503590 (PMC12865148; doi:10.1002/chem.202503590)
Supplement: Supplementary file 1 — The data that support the findings of this study are available in the supplementary material of this article. Deposition number 2496823 (for 11) contains the supplementary crystallographic data for this paper. This data is provided free of charge by the joint Cambridge Crystallographic Data Centre and Fachinformationszentrum Karlsruhe Access Structures service. The authors have cited additional references within the Supporting Information [36, 37, 38, 39, 40, 41, 42, 43, 44, 45, 46, 47, 48, 49, 50, 51, 52, 53, 54, 55, 56, 57, 58, 59]. [file CHEM-32-e03590-s002.pdf]

A Riboflavin-derived Flavinium Salt Mediates Chemoselective Methylation Reactions

Supporting Information

Tim Langschwager, Ekrem Suylu, Julian Zuber, Prof. Dr. Golo Storch

Correspondence to: golo.storch@tum.de

Department of Chemistry, TUM School of Natural Sciences, Technical University of Munich, Lichtenbergstr. 4, 85747 Garching, Germany

Table of Contents

1. General Information 3
2. Flavin Synthesis 5
3. Mechanistic Experiments 14
4. Substrate Synthesis 24
5. Screening 28
6. Methylation Experiments 30
7. Comparative Reactions with Methyl triflate 63
8. Cleavage of Flavin Adduct 11 70
9. Crystallographic Data 73
10. NMR Spectra 88
11. References 119

1. General Information

1.1 General Remarks

Room temperature is defined as 21–23°C. All reactions with air sensitive reactants were carried out under an argon atmosphere (Ar 4.8) applying standard *Schlenk* technique.

1.2 Solvents and Reagents

Unless otherwise noted, all chemicals were obtained from Sigma-Aldrich, Acros, TCI, abcr, Fisher Scientific, BLD Pharm, or Alfa Aesar and used without further purification. Dichloromethane (2×MB-KOLA type 2, aluminium oxide), was obtained from an MBSPS 800 MBraun solvent purification system.

1.3 Analytics and Devices

1.3.1 Thin Layer Chromatography (TLC) and Column Chromatography

Thin layer chromatography was carried out with TLC glass plates purchased from Merck (0.25 mm, silica 60, F_{254}). Spots were detected by UV light (λ = 254 nm), color and standard stains, i.e. $KMnO_4$ stain, I_2 stain (developing by iodine vapour).

Preparative flash column chromatography was performed on silica 60 (Merck, 230-400 mesh). Automated flash column chromatography was performed on a Biotage Selekt device using Biotage® Sfär C18 Duo columns (12 g) and UV based detection.

1.3.2 High-Performance Liquid Chromatography (HPLC)

Analytical and preparative HPLC measurements were performed on an Agilent Technologies 1290 Infinity II HPLC (Agilent Technologies, Palo Alto, California, USA), equipped with a binary solvent pump, an autosampler, membrane solvent degasser, and DAD/VWD detectors. All operations were controlled by the Agilent ChemStation software (Agilent Technologies, Palo Alto, California, USA). The solvents used were obtained from Sigma-Aldrich (HPLCgrade quality). For preparative HPLC a MACHEREY-NAGEL Kromasil 100-5-SIL (21.2 x 250 mm) column was used. Flow rate: 19 mL/min

1.3.3 Nuclear Magnetic Resonance (NMR) Spectroscopy

NMR spectra were recorded on Bruker AVHD500-, AV400-, AV300- and AV500cryo-devices at 298 K. Chemical shifts are given in the delta-scale (ppm) and ^1H NMR-spectra are referenced to the residual proton signals of the deuterated solvents ($CDCl_3$ = 7.26 ppm, DMSO–d_6 = 2.50 ppm, CD_2Cl_2 = 5.32 ppm, MeCN–d_3 = 1.94 ppm, acetone–d_6 = 2.05 ppm). ^{13}C NMR-spectra are referenced to the deuterium coupling of the solvents ($CDCl_3$ = 77.2 ppm, DMSO–d_6 = 39.5 ppm, CD_2Cl_2 = 53.8 ppm, MeCN–d_3 = 1.32 ppm, acetone–d_6 = 29.84 ppm).

Solvents were supplied by Sigma Aldrich or Deutero. The following abbreviations were used for the assignments of the multiplets: s-singlet, d-doublet, t-triplet. q-quartet, p-pentett, sex-sextet, hept-heptet, m-multiplet, br-broad, *virt.*-virtual. Coupling constants *J* [Hz] were calculated as the average of the coupling and back-coupling. For signal assignments the following experiments were used: ^{1}H-^{13}C-HSQC-ME, ^{1}H-^{13}C-HMBC, ^{1}H-^{1}H-COSY and ^{1}H-^{1}H-NOESY. In cases where an unambiguous assignment was not possible, it is indicated by "/", while "," is used in cases where resonances of two or more atoms overlap. Apparent multiplets which occur as a result of accidental equality of coupling constants to those of magnetically non-equivalent protons are marked as virtual (*virt.*). Note that small deviations in chemical shifts may be observed depending on the concentration of NMR samples.

1.3.4 High Resolution Mass Spectrometry (HR-MS)

High-resolution mass spectrometry was performed on a Thermo Scientific Q Executive Plus (HRESI) equipped with an orbitrap mass analyzer and a Thermo Scientific LTQ-Orbitrap XL using an electron spray ionization (ESI) mode.

1.3.5 Ultra Performance Liquid Chromatography Mass Spectrometry (UPLC-MS)

Ultra Performance Liquid Chromatography Mass Spectrometry was performed on a Dionex UltiMate 3000 UHPLC device with a Rs variable wavelength detector (200 nm) and a C_{18} reversed phase column coupled to a Thermo Scientific LCQ Fleet mass spectrometer (electrospray ionization, positive mode). A water/acetonitrile (containing 0.1 v/v% formic acid) gradient (90/10→10/90 over 20 min) and a flow rate of 0.7 mL/min was applied.

1.3.6 Infrared (IR) Spectroscopy

Infrared spectra were recorded on a Perkin Elmer Frontier ATR/FT-IR spectrometer, and $\tilde{\nu}_{max}$ are reported in cm^{-1}. The signal intensity is assigned using the following abbreviations: vs-very strong, s-strong, m-medium, w-weak, br-broad.

1.3.7 Elemental Analysis

CHNS-Analysis was simultaneously performed with an EuroVector Elemental Analyzer. The fluorine content was separately determined via titration with a Metrohm Titrando 904 device.

2. Flavin Synthesis

2.1 2-(7,8-Dimethyl-2,4-dioxo-3,4-dihydrobenzo[*g*]pteridin-10(2*H*)-yl)acetaldehyde (SI-1)

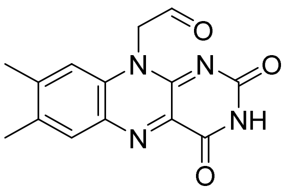

SI-1

According to a modified literature procedure,[24] (–)-riboflavin (3.80 g, 10.1 mmol, 1.00 equiv.) is dissolved in water (55 mL, 185 mM) and sodium periodate (5.94 g, 27.8 mmol, 2.75 equiv.) is slowly added. The reaction is stirred for 24 h at r.t. and the resulting suspension is filtered. The residual solid is washed with water (200 mL), methanol (100 mL), and diethyl ether (150 mL). The obtained flavin **SI-1** is used without further purification and characterization.

Orange solid; 2.43 g (8.55 mmol, 85%).

2.2 10-(2-Hydroxyethyl)-7,8-dimethylbenzo[*g*]pteridine-2,4(3*H*,10*H*)-dione (SI-2)

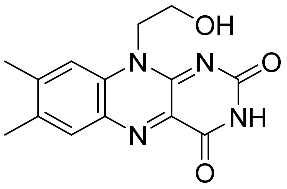

SI-2

According to a modified literature procedure,[25] flavin **SI-1** (4.84 g, 17.0 mmol, 1.00 equiv.) is dissolved in technical grade methanol (500 mL, 34 mM) and sodium borohydride (1.03 g, 27.3 mmol, 1.60 equiv.) is slowly added. The reaction is stirred for 4 h at r.t. and another portion of sodium borohydride (1.03 g, 27.3 mmol, 1.60 equiv.) is slowly added. The reaction is stirred for 13 h at r.t., is filtered and the residual solid is washed with water (100 mL), methanol (100 mL), and diethyl ether (100 mL). The obtained flavin **SI-2** is used without further purification and characterization.

Orange solid; 3.74 g (13.1 mmol, 77%).

2.3 3-Butyl-10-(2-hydroxyethyl)-7,8-dimethylbenzo[*g*]pteridine-2,4(3*H*,10*H*)-dione (SI-3)

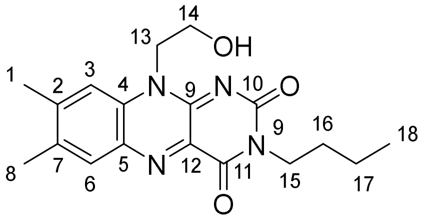

SI-3

Flavin **SI-2** (3.74 g, 13.1 mmol, 1.00 equiv.) and potassium carbonate (10.3 g, 102 mmol, 7.80 equiv.) are dissolved in dimethyl sulfoxide (50 mL, 26 mM) and *n*-butyl iodide (11.6 mL, 18.7 g, 102 mmol, 7.80 equiv.) is slowly added. The reaction is stirred for 6 d at r.t. and diethyl ether (250 mL) is added. The precipitated solid is washed with diethyl ether (7×250 mL) until most of the dimethyl sulfoxide is removed. The solid is then washed with water (500 mL) and diethyl ether (100 mL) to remove residual dimethyl sulfoxide and potassium salts and is dried *in vacuo* to obtain flavin **SI-3**.

Orange solid; 3.29 g (9.61 mmol, 74%); **^{1}H NMR** (500 MHz, DMSO–d_6, 298 K): δ = 7.93 (s, 1H, H^{6}), 7.91 (s, 1H, H^{3}), 4.93 (t, $^{3}J_{H\text{-}H}$ = 6.0 Hz, 1H, OH), 4.71 (t, $^{3}J_{H\text{-}H}$ = 6.0 Hz, 2H, H^{13}), 3.88 (t, $^{3}J_{H\text{-}H}$ = 7.4 Hz, 2H, H^{15}), 3.82 (t, $^{3}J_{H\text{-}H}$ = 6.0 Hz, 2H, H^{14}), 2.50 (s, 3H, H^{1}), 2.41 (s, 3H, H^{8}), 1.56 (*virt.* p, $^{3}J_{H\text{-}H} \approx {}^{3}J_{H\text{-}H}$ = 7.4 Hz, 2H, H^{16}), 1.56 (*virt.* sex, $^{3}J_{H\text{-}H} \approx {}^{3}J_{H\text{-}H}$ = 7.4 Hz, 2H, H^{17}), 0.91 (t, $^{3}J_{H\text{-}H}$ = 7.4 Hz, 3H, H^{18}); H^{1} overlaps with the DMSO-d_6 signal as confirmed by ^{1}H-^{13}C-HSQC; **^{13}C{^{1}H} NMR** (126 MHz, DMSO–d_6, 298 K): δ = 159.5 ($C^{10/11}$), 154.8 ($C^{10/11}$), 148.9 (C^{9}), 146.4 (C^{7}), 136.2 (C^{5}), 135.9 (C^{2}), 134.2 (C^{12}), 131.8 (C^{4}), 130.8 (C^{6}), 116.9 (C^{3}), 57.4 (C^{14}), 46.4 (C^{13}), 40.6 (C^{15}), 29.6 (C^{16}), 20.8 (C^{7}), 19.8 (C^{17}), 18.8 (C^{8}), 13.8 (C^{18}); **HR-MS** (ESI^{+}): *m/z* = calc. for $[C_{18}H_{23}N_4O_3]^{+}$: 343.1765 ($[M+H]^{+}$), found: 343.1768; **IR** (ATR): $\tilde{\nu}_{max}$ [cm^{-1}] = 3416 (w, O–H), 2956 (w, C–H_{Ar}), 2931 (w, C–H_{Ar}), 2871 (w), 1712 (w, C=O), 1639 (s, C=O), 1579 (s, C–C_{Ar}), 1545 (s, C–C_{Ar}), 1510 (s, C–C_{Ar}), 1462 (s, C–C_{Ar}), 1444 (s, C–C_{Ar}), 1432 (s, C–C_{Ar}), 1404 (w), 1386 (w), 1368 (w), 1350 (w), 1341 (w), 1309 (w), 1270 (w), 1240 (w), 1209 (w), 1193 (w), 1168 (w), 1151 (w), 1071 (w), 1056 (w), 1022 (w), 997 (w), 983 (w), 928 (w), 897 (w), 854 (w), 806 (w), 778 (w), 760 (w), 733 (w), 694 (w).

2.4 5-Butyl-9,10-dimethyl-4,6-dioxo-1,2,5,6-tetrahydro-4*H*-benzo[*g*]imidazo[1,2,3-*ij*] pteridin-3-ium chloride (10)

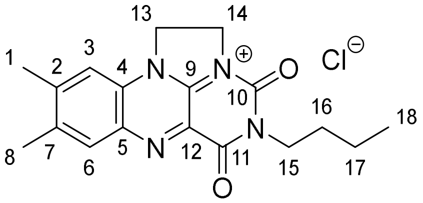

10

Flavin **SI-3** (1.00 g, 2.94 mmol, 1.00 equiv.) is dissolved in thionyl chloride (5.30 mL, 8.69 g, 73.0 mmol, 25.0 equiv.) and the reaction is stirred for 20 h at r.t. in the dark. The product is precipitated with diethyl ether (75 mL), the residual solid is washed with diethyl ether (3×75 mL) and dissolved in acetonitrile (4 mL). The product is precipitated and washed with diethyl ether (3×50 mL) to afford flavin reagent **10**.

Yellow-orange solid; 868 mg (2.46 mmol, 84%); **^{1}H NMR** (500 MHz, DMSO–d_6, 298 K): δ = 8.42 (s, 1H, H^6), 8.16 (s, 1H, H^3), 5.33 (t, $^3J_{H\text{-}H}$ = 9.4 Hz, 2H, H^{13}), 4.71 (t, $^3J_{H\text{-}H}$ = 9.4 Hz, 2H, H^{14}), 4.01 (t, $^3J_{H\text{-}H}$ = 7.3 Hz, 2H, H^{15}), 2.66 (s, 3H, H^8), 2.57 (s, 3H, H^1), 1.61 (*virt.* p, $^3J_{H\text{-}H} \approx {}^3J_{H\text{-}H}$ = 7.7 Hz, 2H, H^{16}), 1.56 (*virt.* sex, $^3J_{H\text{-}H} \approx {}^3J_{H\text{-}H}$ = 7.4 Hz, 2H, H^{17}), 0.93 (t, $^3J_{H\text{-}H}$ = 7.4 Hz, 3H, H^{18}); **^{13}C{^{1}H} NMR** (126 MHz, DMSO–d_6, 298 K): δ = 157.5 (C$^{10/11}$), 152.1 (C^7), 146.9 (C$^{10/11}$), 142.5 (C^9), 142.3 (C^2), 138.8 (C^4), 131.9 (C^{12}), 131.0 (C^6), 127.5 (C^5), 117.1 (C^3), 50.3 (C^{13}), 45.6 (C^{14}), 41.7 (C^{15}), 29.3 (C^{16}), 21.1 (C^1), 19.6 (C^8), 19.5 (C^{17}), 13.7 (C^{18}); **HR-MS** (ESI$^+$): *m/z* = calc. for $[C_{18}H_{22}N_4O_2]^+$: 325.1659 ([M]$^+$), found: 325.1660; **IR** (ATR): $\tilde{\nu}_{max}$ [cm^{-1}] = 3371 (w), 2964 (w, C–H$_{Ar}$), 1744 (m), 1694 (s, C=O), 1635 (m, C=O), 1612 (m, C–C$_{Ar}$), 1476 (w, C–C$_{Ar}$), 1393 (w), 1371 (w), 1344 (w), 1244 (m), 1097 (w), 734 (w).

2.5 5-Butyl-9,10-dimethyl-4,6-dioxo-1,2,5,6-tetrahydro-4*H*-benzo[*g*]imidazo[1,2,3-*ij*] pteridin-3-ium bistriflimide (12)

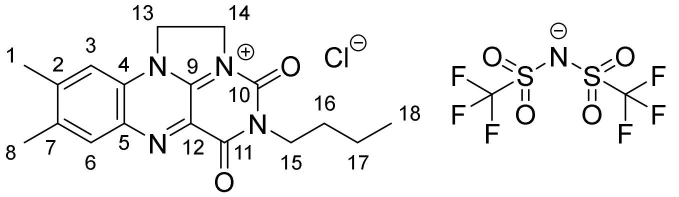

12

Flavin **10** (456 mg, 1.26 mmol, 1.00 equiv.) is dissolved in dichloromethane (42 mL, 30 mM) and acetone (11 mL, 120 mM) and silver(I) bistriflimide (735 mg, 1.90 mmol, 1.50 equiv.) is added. The reaction is stirred for 5.5 h at r.t. and stopped by adding brine (20 mL). The phases are separated, and the organic phase is extracted with brine (3×20 mL). All volatiles are removed *in vacuo* to afford flavin reagent **12**.

Yellow solid; 670 mg (1.10 mmol, 88%); **^{1}H NMR** (500 MHz, DMSO–d_6, 298 K): δ = 8.43 (s, 1H, H^{6}), 8.15 (s, 1H, H^{3}), 5.33 (t, $^{3}J_{H\text{-}H}$ = 9.3 Hz, 2H, H^{13}), 4.71 (t, $^{3}J_{H\text{-}H}$ = 9.3 Hz, 2H, H^{14}), 4.01 (t, $^{3}J_{H\text{-}H}$ = 7.3 Hz, 2H, H^{15}), 2.66 (s, 3H, H^{8}), 2.57 (s, 3H, H^{1}), 1.62 (*virt.* p, $^{3}J_{H\text{-}H} \approx {}^{3}J_{H\text{-}H}$ = 7.5 Hz, 2H, H^{16}), 1.36 (*virt.* sex, $^{3}J_{H\text{-}H} \approx {}^{3}J_{H\text{-}H}$ = 7.4 Hz, 2H, H^{17}), 0.93 (t, $^{3}J_{H\text{-}H}$ = 7.4 Hz, 3H, H^{18}); **^{19}F{^{1}H} NMR** (376 MHz, DMSO–d_6, 298 K): δ = -78.70; **^{13}C{^{1}H} NMR** (126 MHz, DMSO–d_6, 298 K): δ = 157.5 ($C^{10/11}$), 152.2 (C^{7}), 146.8 ($C^{10/11}$), 142.5 (C^{9}), 142.3 (C^{2}), 138.8 (C^{4}), 131.8 (C^{12}), 131.0 (C^{6}), 127.5 (C^{5}), 119.5 (q, $^{1}J_{C\text{-}F}$ = 322.0 Hz, 2C, CF_3), 117.1 (C^{3}), 50.3 (C^{13}), 45.6 (C^{14}), 41.7 (C^{15}), 29.3 (C^{16}), 21.1 (C^{1}), 19.6 (C^{8}), 19.5 (C^{17}), 13.7 (C^{18}); **HR-MS** (ESI^{+}): *m/z* = calc. for $[C_{18}H_{22}N_4O_2]^{+}$: 325.1659 ($[M]^{+}$), found: 325.1645; **IR** (ATR): $\tilde{\nu}_{max}$ [cm^{-1}] = 3063 (w, C–H_{Ar}), 2962 (w, C–H_{Ar}), 2875 (w), 1747 (m, C=O), 1699 (s, C=O), 1635 (m), 1612 (m), 1518 (w, C–C_{Ar}), 1476 (m, C–C_{Ar}), 1444 (m, C–C_{Ar}), 1412 (w, C–C_{Ar}), 1392 (m, C–C_{Ar}), 1355 (s), 1270 (w), 1239 (m), 1188 (vs), 1136 (m), 1048 (m), 1007 (w), 939 (w), 905 (w), 853 (w), 791 (m), 739 (m), 654 (w), 614 (s); **Elemental Analysis** (%) calc. for $C_{20}H_{21}F_6N_5O_6S_2$: C 39.67, H 3.50, N 11.57, S 10.59, F 18.82, O 15.85; found C 39.71, H 3.28, N 11.17, S 10.75, F 17.71, O n.d.

To proof and simplify the quantification of the ion exchange reaction, a method using phenyl trifluoromethanesulfonate as an internal standard was developed. Therefore, a small quantity of flavin reagent **12** and internal standard are dissolved in DMSO–d_6 and ^{1}H and ^{19}F{^{1}H} NMR spectra are recorded (note that the ^{19}F{^{1}H} -NMR offset must be set to the mean of the chemical shifts of the analyte and internal standard to allow quantification[36]). The fractions $f(^{19}F)$ and

$f(^1H)$ of the integrals of the $^{19}F\{^1H\}$ NMR and ^{1}HNMR, weighted by the respective number of nuclei n that the integral corresponds to, are determined. The coefficient of these two fractions q represents the quantity of the ion exchange in percent.

$$f(^{19}F) = \frac{I(xxx)/n}{I(PhOTf)/n} = \frac{1.00/6}{2.70/3} = 0.1852$$

$$f(^{1}H) = \frac{I(xxx)/n}{I(PhOTf)/n} = \frac{1.00/1}{26.22/5} = 0.1906$$

$$q = \frac{f(^{19}F)}{f(^{1}H)} * 100\% = \frac{0.1852}{0.1906} * 100\% = 97\%$$

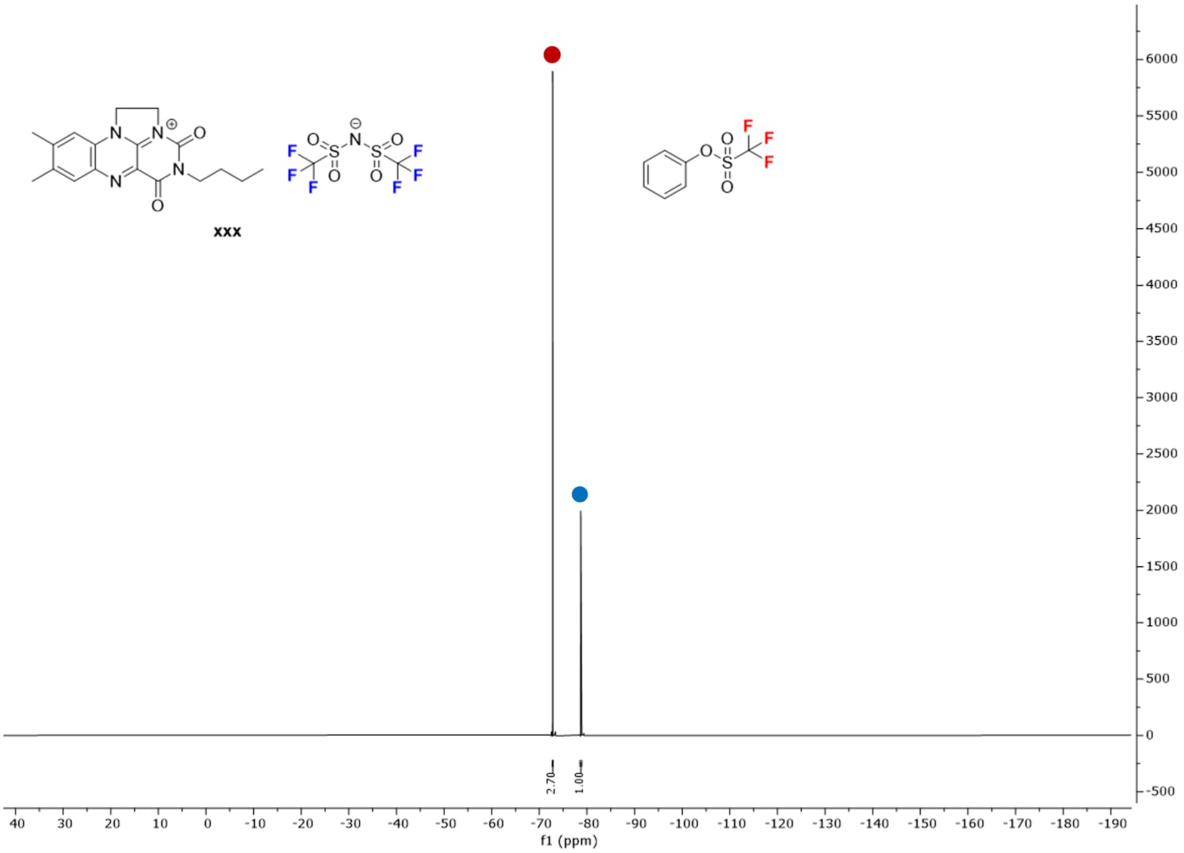

Figure S 1: $^{19}F\{^1H\}$ NMR spectrum of flavin **12** with internal standard.

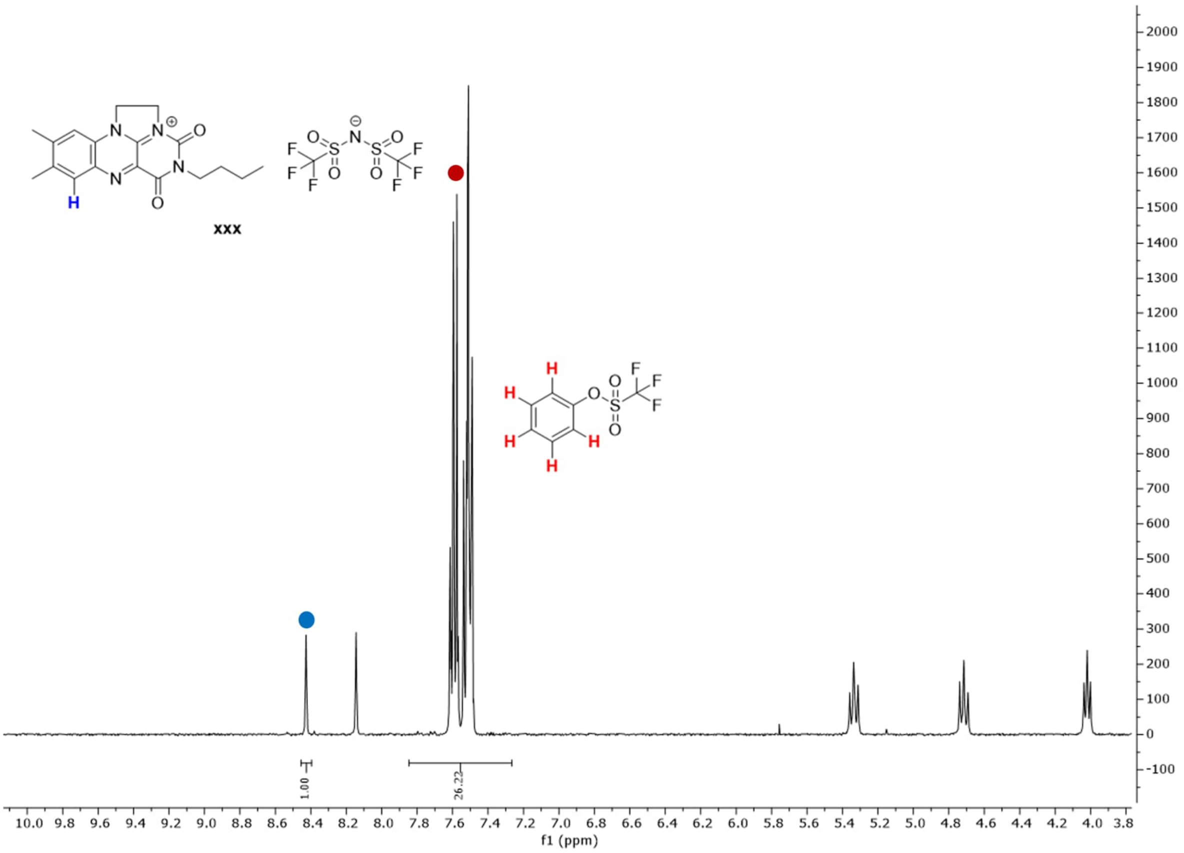

Figure S 2: ^{1}H-NMR spectrum of flavin **12** with internal standard.

2.6 3-Butyl-10-methylbenzo[*g*]pteridine-2,4(3*H*,10*H*)-dione (SI-4)

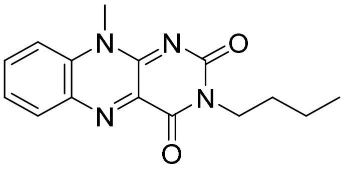

SI-4

The synthesis of flavin **SI-4** was previously reported by our group.[37]

2.7 5-Butyl-7-(diphenylphosphoryl)-9,10-dimethyl-1,2-dihydro-4*H*,7*H*-benzo[*g*]imidazo [1,2,3-*ij*]pteridine-4,6(5*H*)-dione (11)

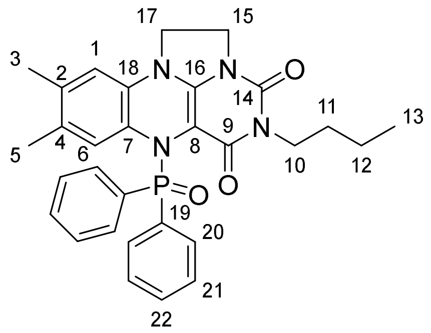

11

Flavin **10** (9.0 mg, 25.0 µmol, 1.00 equiv.) is dissolved in MeCN-d_3 (500 µL, 50 mM) in a *J Young* NMR tube under Ar atmosphere and methyl diphenylphosphinite (25.1 µL, 27.0 mg, 125 µmol, 5.00 equiv.) is added. The tube is left for 1.3 h at r.t. and the solvent is removed *in vacuo*. The remaining oil is dissolved in CH_2Cl_2 (20 mL), and the solvent is again removed *in vacuo*. The flavin adduct **11** is precipitated with Et_2O (5 mL) and the remaining solid is washed with Et_2O (2×5 mL). Crystallization is achieved from dissolving the substance in a few 100 µL of an acetonitrile/water mixture and slow evaporation of the acetonitrile content at ambient temperature and atmosphere.

Pale orange solid; 13.0 mg (24.7 mmol, 99%); **^{1}H NMR** (500 MHz, CD_2Cl_2, 298 K): δ = 8.11 (br. s, 2H, H^{20a}/H^{21a}), 7.72 (br. s, 2H, H^{20b}/H^{21b}), 7.62–7.23 (m, 6H, $H^{20a}/H^{21a}/H^{20b}/H^{21b}$, H^{22}), 6.64 (s, 1H, H^6), 6.50 (s, 1H, H^1), 4.26–4.17 (m, 3H, H^{17a}, H^{15}), 3.69 (br. s, 1H, H^{17b}), 3.54 (br. s, 1H, H^{10a}), 3.47 (br. s, 1H, H^{10b}), 2.14 (s, 3H, H^3), 1.94 (s, 3H, H^5), 1.10–1.02 (m, 4H, H^{11}, H^{12}), 0.83 (t, $^3J_{H\text{-}H}$ = 6.7 Hz, 3H, H^{13}); **^{13}C{^{1}H} NMR** (126 MHz, CD_2Cl_2, 298 K): δ = 158.5 ($C^9/C^{14}/C^{16}/C^{19}$), 149.7 ($C^9/C^{14}/C^{16}/C^{19}$), 148.4 ($C^9/C^{14}/C^{16}/C^{19}$), 136.5 (C^2/C^{18}), 134.4 (C^2/C^{18}), 133.3 (d, $J_{C\text{-}P}$ = 72.1 Hz, 4C, C^{20}/C^{21}), 132.1 (d, $J_{C\text{-}P}$ = 25.6 Hz, 2C, C^{22}), 132.1 (C^4/C^7), 131.5 ($C^9/C^{14}/C^{16}/C^{19}$), 128.3 (d, $J_{C\text{-}P}$ = 20.4 Hz, 4C, C^{20}/C^{21}), 127.2 (C^4/C^7), 124.9 (C^6), 114.7 (C^1), 94.5 (d, $^2J_{C\text{-}P}$ = 4.8 Hz, C^8), 47.4 (C^{17}), 44.2 (C^{15}), 40.9 (C^{10}), 30.3 (C^{11}), 20.3 (C^{12}), 19.5 (C^3), 19.1 (C^5), 14.0 (C^{13}); **^{31}P{^{1}H} NMR** (162 MHz, CD_2Cl_2, 298 K): 30.7; **HR-MS** (ESI$^+$): *m/z* = calc. for $[C_{30}H_{32}N_4O_3P]^+$: 527.2207 ($[M+H]^+$), found: 527.2204; **IR** (ATR): $\tilde{\nu}_{max}$ [cm^{-1}] = 3059 (w, C_{Ar}–H), 2956 (w, C_{Ar}–H), 2928 (w, C_{Alk}–H), 2871 (w, C–H_{Alk}), 1710 (w), 1677 (m), 1644 (s, C=O), 1591 (m, C=C), 1516 (m), 1495 (m, C=C), 1466 (m), 1457 (m), 1438 (m, P–C), 1407 (m), 1374 (m), 1320 (m), 1277 (m), 1262 (m), 1215 (s, P=O), 1195 (m), 1114 (m), 1103 (m), 1074 (m), 1023 (m), 999 (m), 934 (m), 899 (m), 884 (m), 844 (m), 803 (m), 780 (m), 752 (s), 722 (s), 696 (vs, C_{Ar}–H).

3. Mechanistic Experiments

3.1 Reaction of Flavin 10 with Methyl Diphenylphosphinite

In a *J Young* NMR tube, flavin reagent **10** (1.8 mg, 5.00 µmol, 1.00 equiv.) is placed and the atmosphere is exchanged with Ar (3×). Subsequently, methyl diphenylphosphinite (1.5 µL, 1.6 mg, 7.50 µmol, 1.50 equiv.) and MeCN-d_3 (500 µL, 10 mM) are added and the tube is handled at r.t.

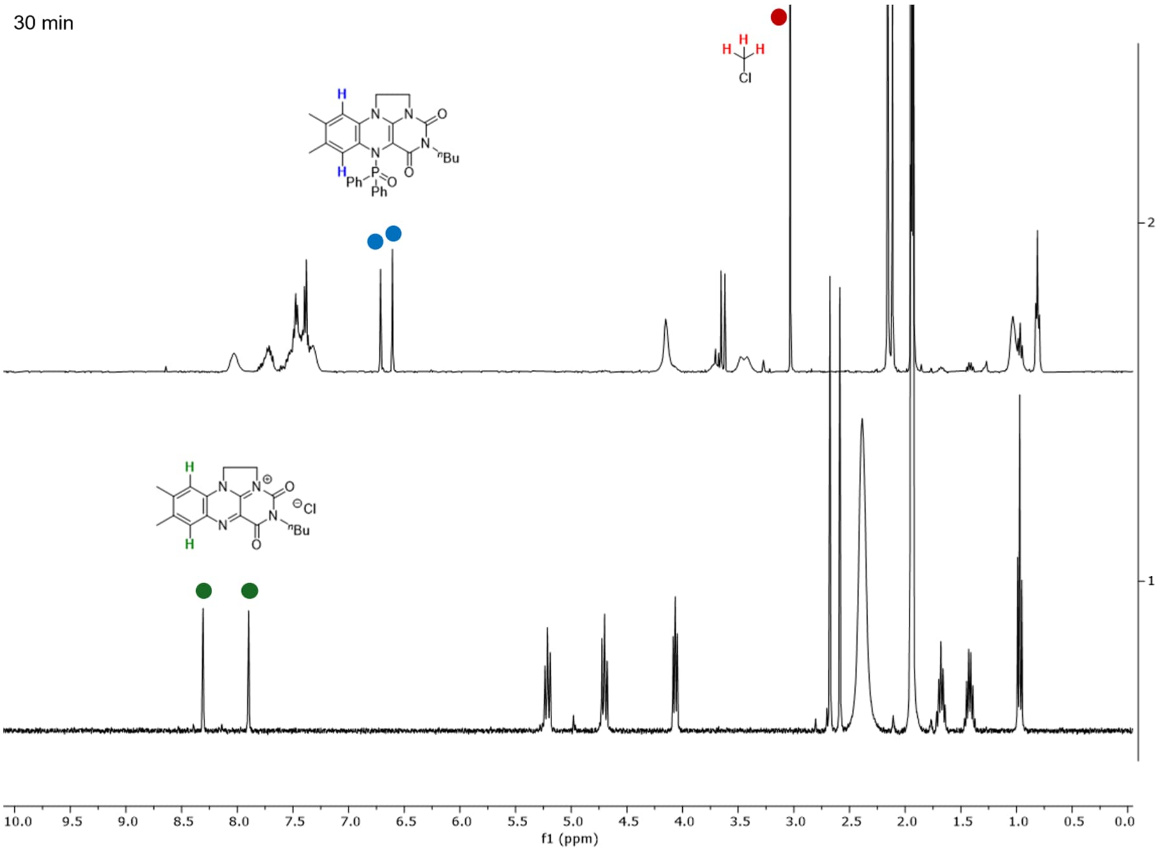

Figure S 3: ^{1}H NMR spectra of the reaction of flavinium reagent **10** with methyl diphenyl phosphinite after 30 min.

Chloromethane was observed by ^{1}H-NMR and its ^{1}H-^{13}C-HSQC-contact from the crude mixture. **^{1}H NMR** (400 MHz, MeCN-d_3, 298 K): δ = 3.03 (s 3H); **^{13}C{^{1}H} NMR** (101 MHz, DMSO-d$_6$, 298 K): δ = 25.9.

The analytical data are in accordance with the literature.[26]

^{1}H-^{13}C-HSQC-contact for the identification of chloromethane

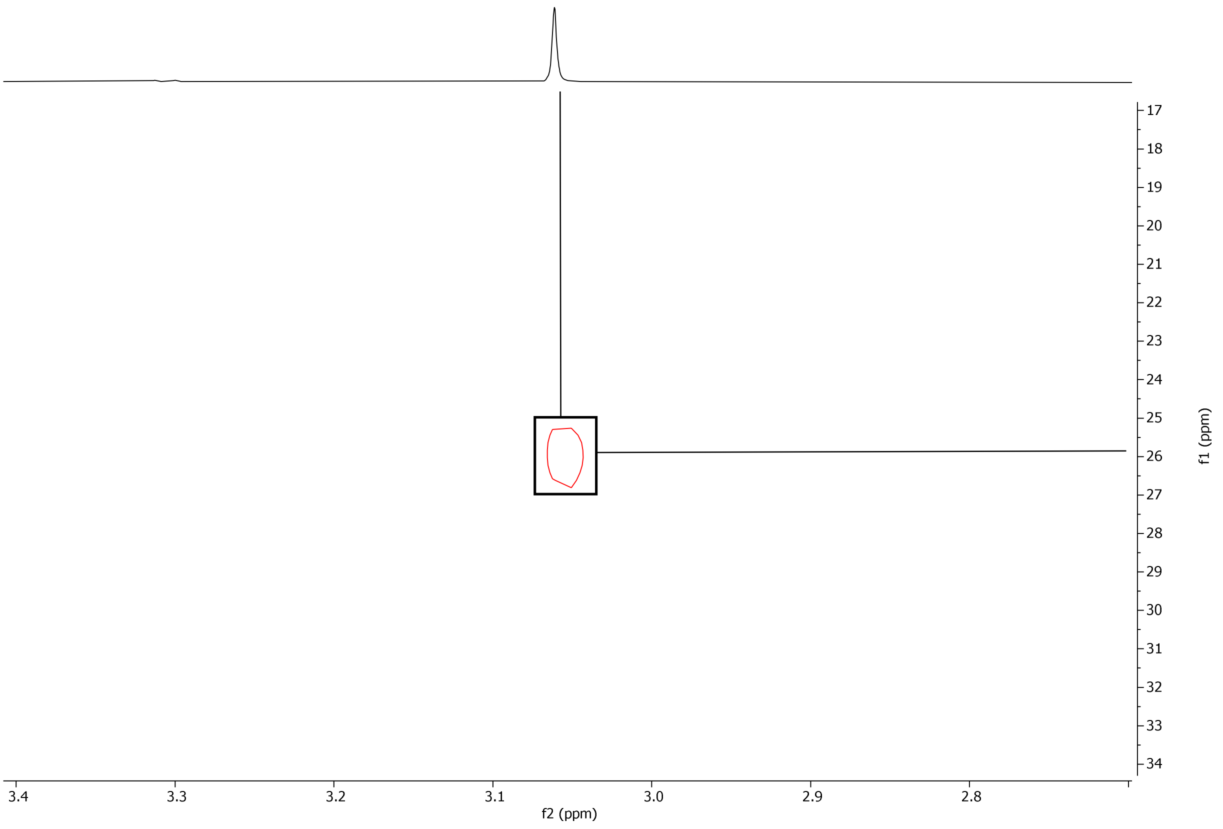

3.2 Reaction of Flavin 12 with Methyl Diphenylphosphinite

In a *J Young* NMR tube, flavin reagent **12** (3.0 mg, 5.00 µmol, 1.00 equiv.) is placed and the atmosphere is exchanged with Ar (3×). Subsequently, methyl diphenylphosphinite (1.5 µL, 1.6 mg, 7.50 µmol, 1.50 equiv.) and MeCN-d_3 (500 µL, 10 mM) are added and the the reaction is monitored at r.t.

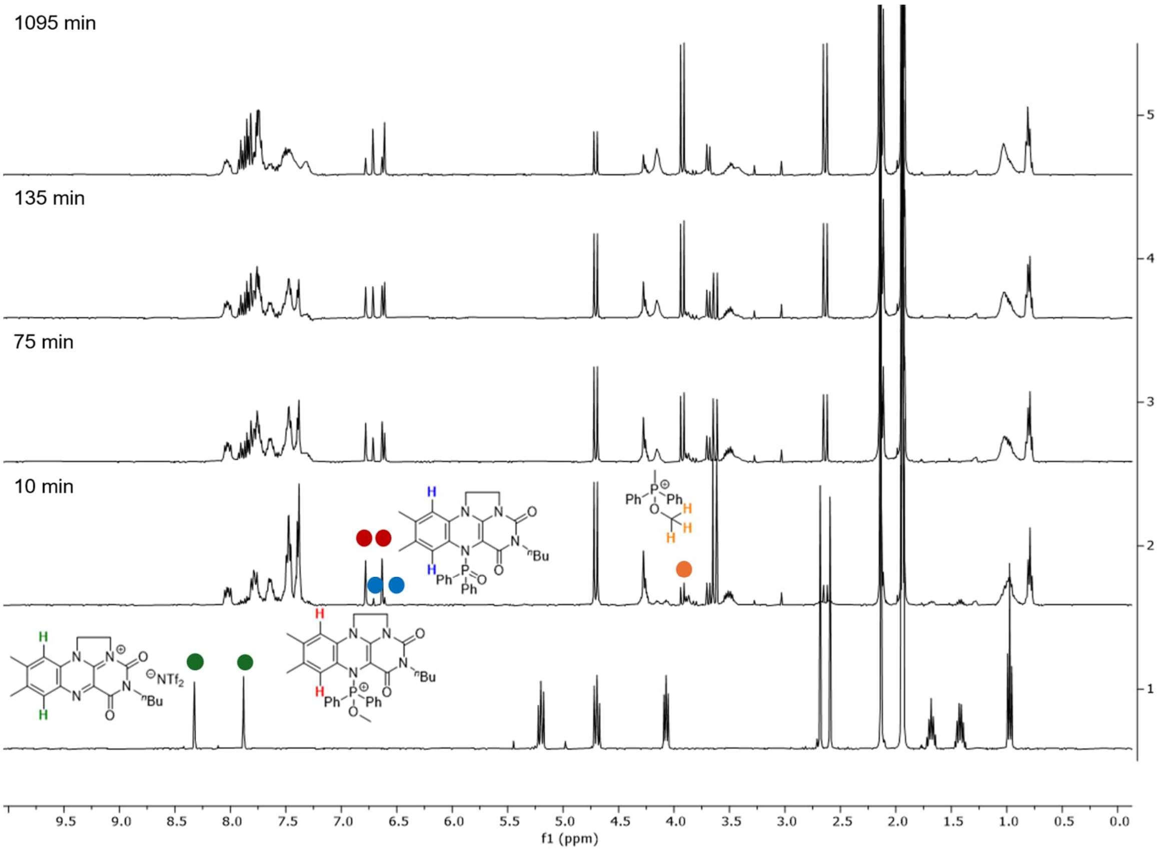

Figure S 4: ^{1}H NMR spectra of the reaction of flavinium reagent **12** with methyl diphenyl phosphinite at different time points.

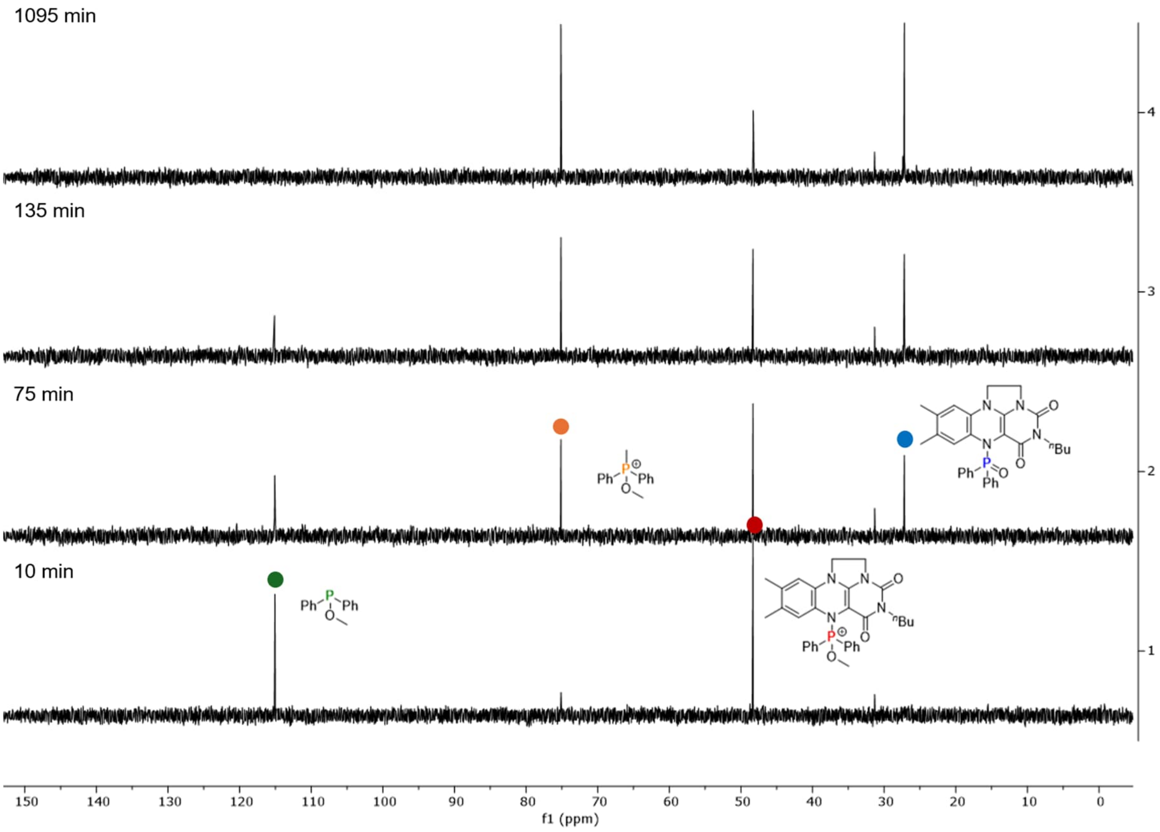

Figure S 5: $^{31}P\{^{1}H\}$ NMR spectra of the reaction of flavinium reagent **12** with methyl diphenyl phosphinite at different time points.

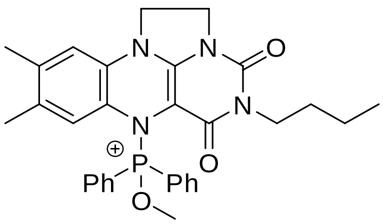

13

Intermediate **13** was observed in HR-MS from the crude mixture. **HR-MS** (ESI^+): *m/z* = calc. for $[C_{31}H_{34}N_4O_3P]^+$: 541.2363 ($[M]^+$), found: 541.2362.

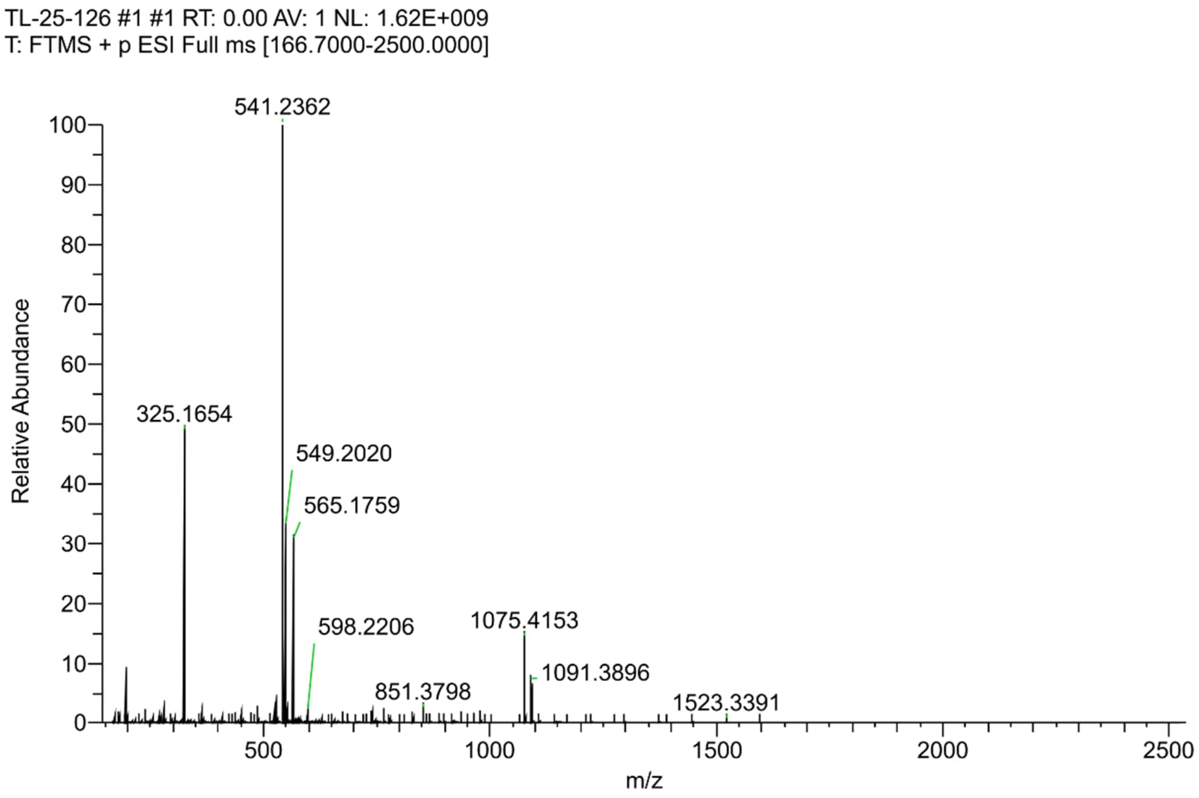

3.3 Reaction of Flavin Adduct 11 with Acid

In an open NMR tube under ambient atmosphere, flavin adduct **11** (2.6 mg, 5.00 µmol, 1.00 equiv.) is dissolved in MeCN-d_3 (500 µL, 10 mM) and a few µL of trifluoracetic acid and D_2O are added. The tube is left at r.t. and after 19 h (1140 min), another portion of trifluoracetic acid and D_2O is added.

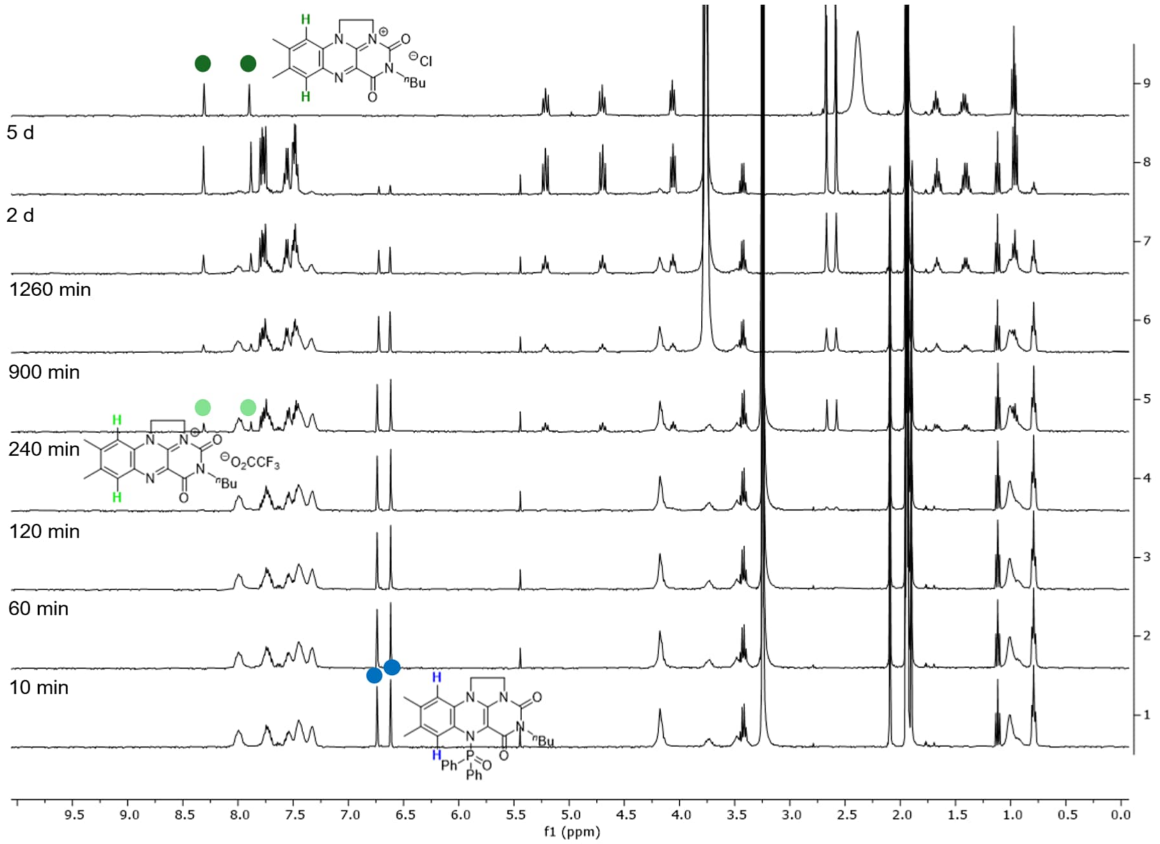

Figure S 6: ^{1}H NMR spectra of the cleavage of flavin adduct **11** at different time points.

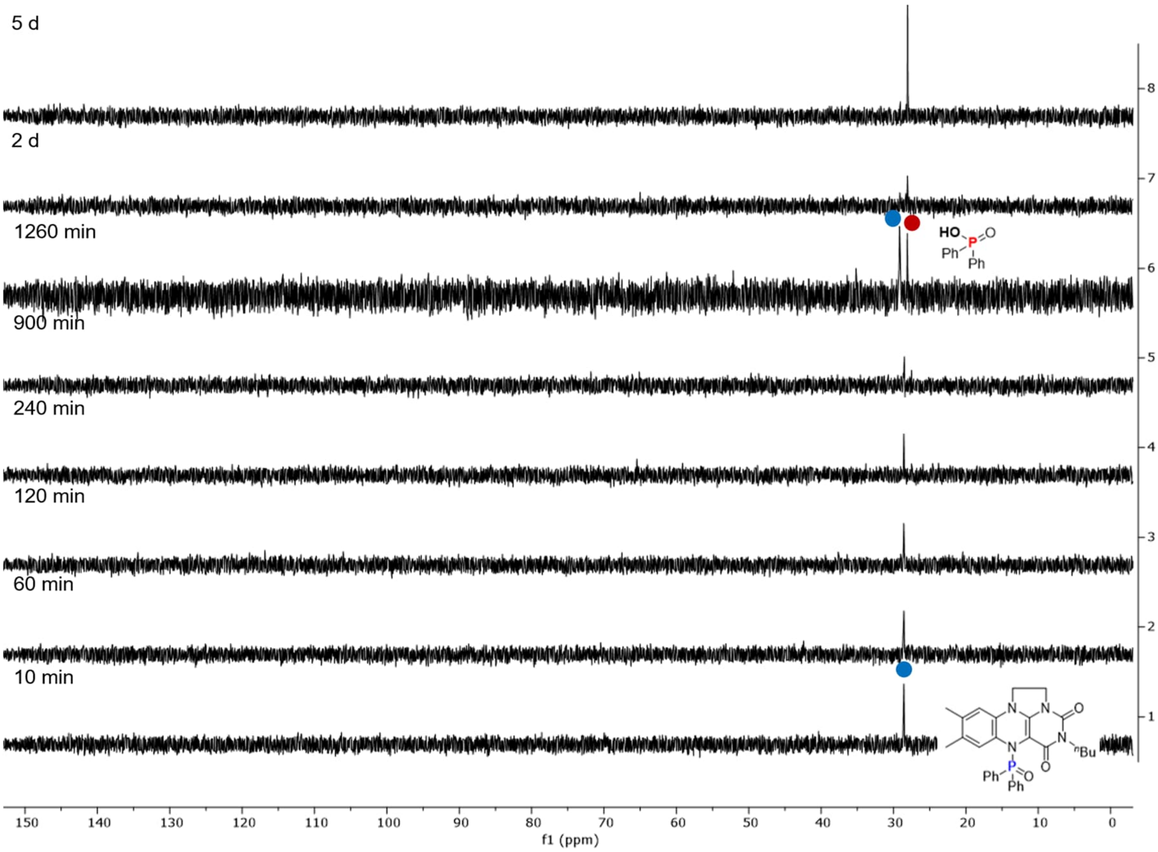

Figure S 7: $^{31}P\{^{1}H\}$ NMR spectra of the cleavage of flavin adduct **11** at different time points.

3.4 Control Experiment with Methoxy(methyl)diphenylphosphonium triflate (23)

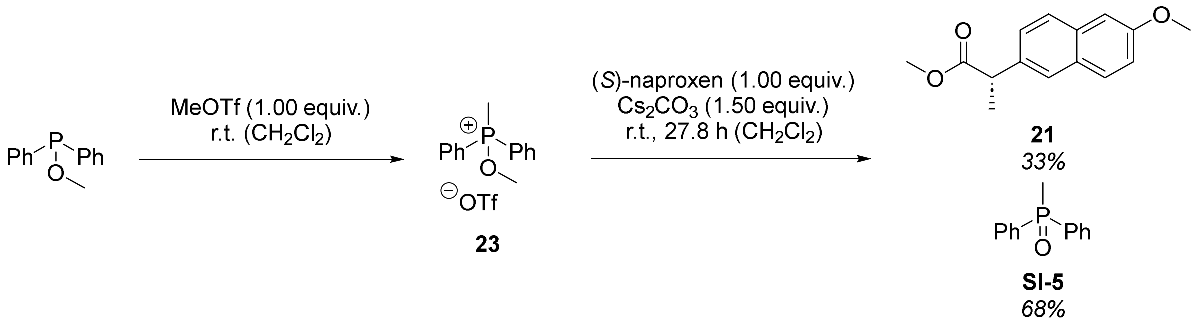

In a crimp-cap vial equipped with a magnetic stirring bar under Ar atmosphere, methyl diphenylphosphinite (10.0 µL, 10.8 mg, 50.0 µmol, 1.00 equiv.) is dissolved in CH_2Cl_2 (2.50 mL, 20 mM) and methyl triflate (5.5 µL, 8.2 mg, 50.0 µmol, 1.00 equiv.) is added (solution **A**). In a second crimp-cap vial equipped with a magnetic stirring bar, (*S*)-naproxen (11.5 mg, 50.0 µmol, 1.00 equiv.) and cesium carbonate (24.4 mg, 75.0 µmol, 1.50 equiv.) are placed. The vial is sealed with a septum safety cap, the atmosphere is exchanged with Ar (3×) and CH_2Cl_2 (2.00 mL, 25 mM) is added (solution **B**). Solution **A** is transferred to solution **B**, the vial of solution **A** is rinsed with CH_2Cl_2 (2×250 µL), and the reaction is stirred at r.t. After 27.8 h, the vial is opened, the solution is diluted with dichloromethane, transferred to a round bottom flask and all volatiles are removed *in vacuo*. The crude product is transferred to an NMR tube and a stock solution of the internal standard trimethyl 1,3,5-benzenetricarboxylate (8.33 µmol in 350 µL $CDCl_3$, relaxation time of the ^1H spectrum is set to $\tau = 10$ s, the integral of the signal at $\delta = 8.82$ ppm is set to 0.50) and DMSO (few µL) and AcOH (few µL) are added.

The NMR yields of product **21** and side product **SI-5** are determined as:

21: 33%

SI-5: 68%

The recorded NMR data for phosphinoxide **SI-5** are in accordance with the literature.[38]

Crude NMR with Internal Standard

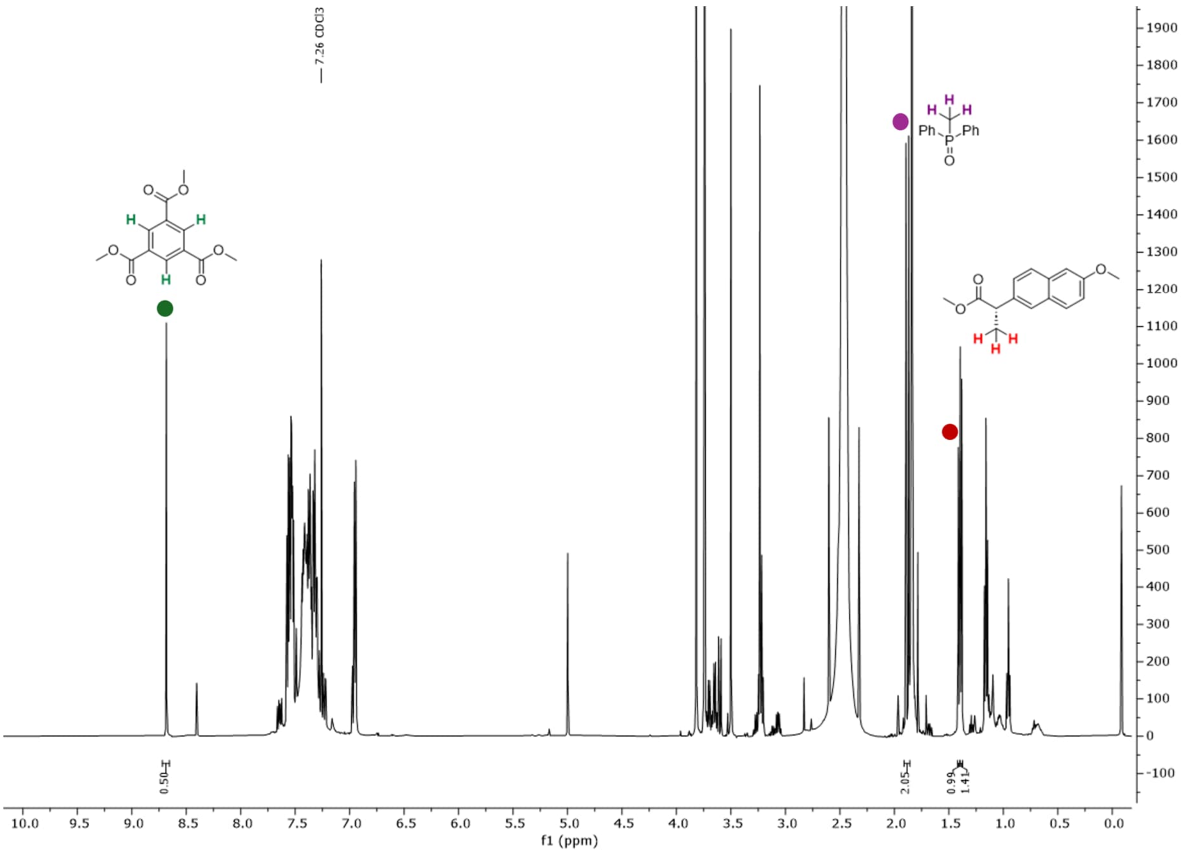

In a separate experiment, the formation of reactive intermediate **23** was proven under the same conditions.

In a *J Young* NMR tube under Ar atmosphere, methyl diphenylphosphinite (4.0 µL, 4.3 mg, 20.0 µmol, 1.00 equiv.) and trimethyl trimesate (1.7 mg, 6.67 µmol, 0.33 equiv.) are dissolved in CD_2Cl_2 (500 µL, 40 mM) and methyl triflate (2.2 µL, 3.3 mg, 20.0 µmol, 1.00 equiv.) is added. An ^{1}H NMR spectrum (τ = 10 s) is recorded and intermediate **23** is detected with 88% NMR yield.

The recorded NMR data are in accordance with the literature and figure S 4 and S 5.[39]

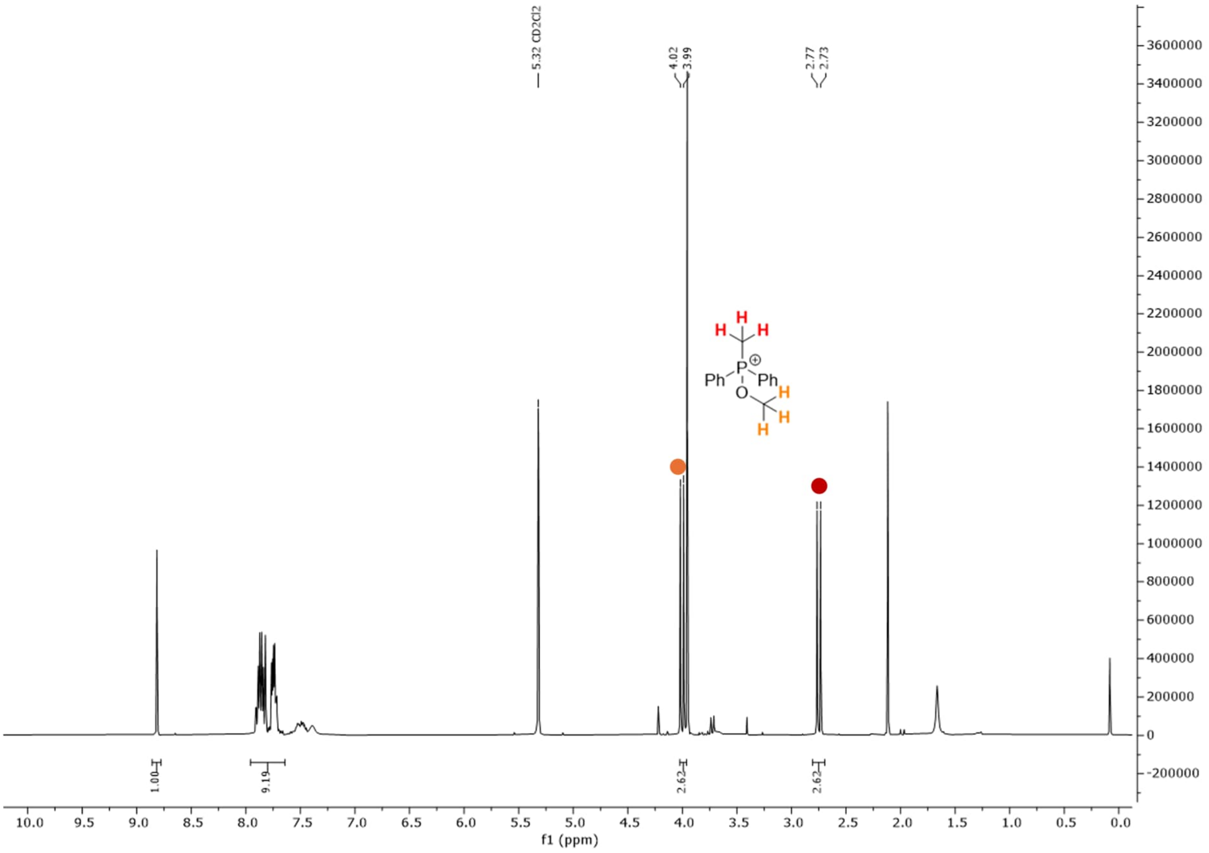

Figure S 8: ^{1}H NMR spectrum of intermediate **23**.

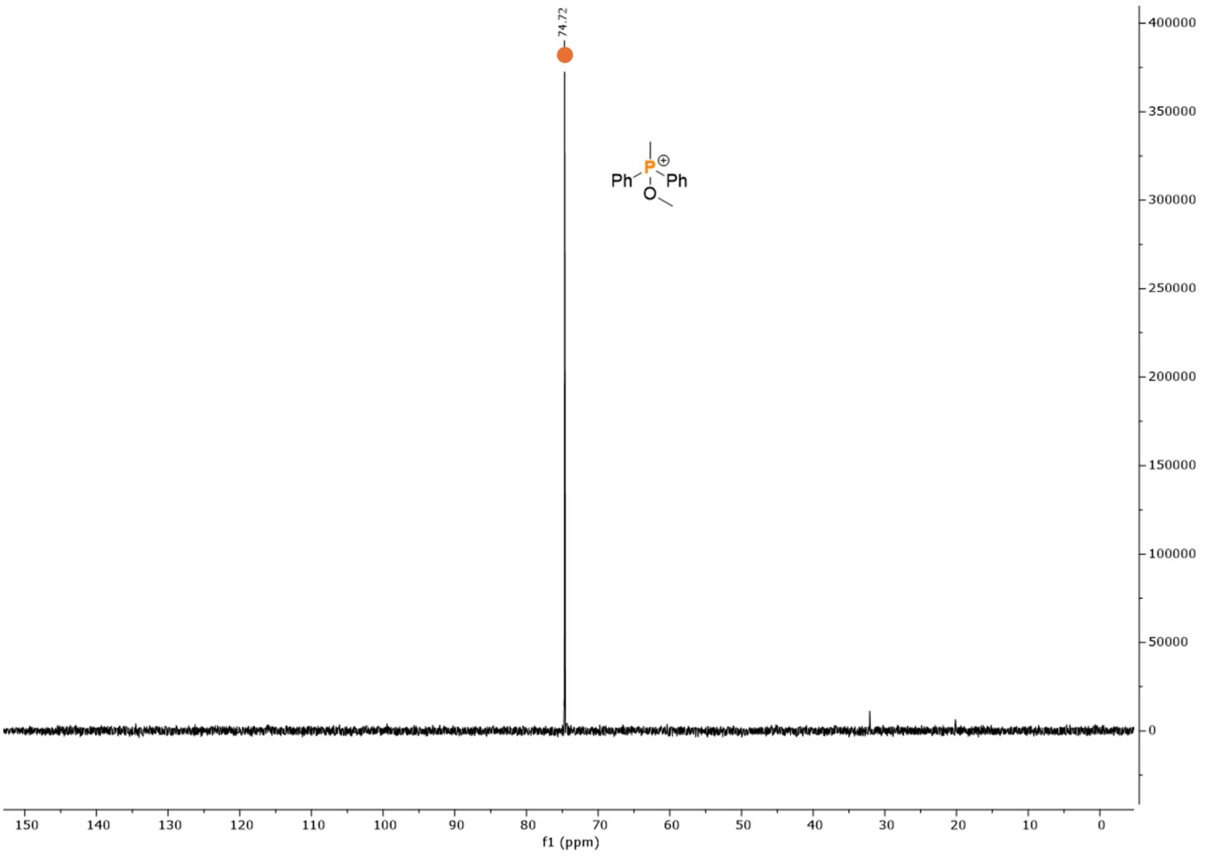

Figure S 9: $^{31}P\{^{1}H\}$ NMR spectrum of intermediate **23**.

4. Substrate Synthesis

4.1 Dimethyl 2,2'-(((2*R*,2'*R*)-3,3'-disulfanediylbis(2-((*tert*-butoxycarbonyl)amino)-propanoyl))bis(azanediyl))(2*S*,2'*S*)-bis(3-(4-hydroxyphenyl)propanoate) (SI-6)

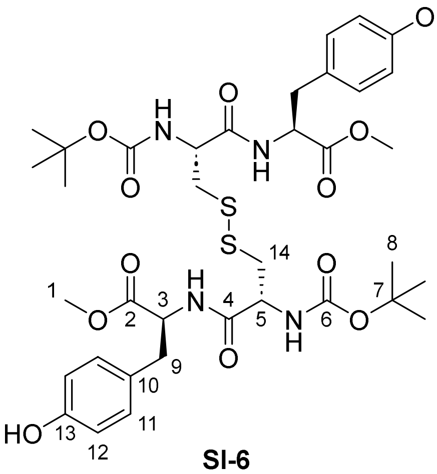

SI-6

According to a modified literature procedure,[40] (L-Boc-Cys-OH)$_2$ (713 mg, 1.62 mmol, 1.00 equiv.) is dissolved in anhydrous dichloromethane (32 mL, 100 mM). The solution is cooled to 0°C and DIPEA (1.65 mL, 1.26 mg, 9.71 mmol, 6.00 equiv.), HOBt•H_2O (545 mg, 3.56 mmol, 2.20 equiv.), EDC•HCl (683 mg, 3.56 mmol, 2.20 equiv.) and L-H-Tyr-OMe•HCl (750 mg, 3.24 mmol, 2.00 equiv.) are added. The solution is warmed to r.t. and stirred for 16 h. The reaction is quenched by adding NH_4Cl solution (aqueous, sat., 20 mL), the phases are separated, and the aqueous phase is extracted with CH_2Cl_2 (3×25 mL). The combined organic phases are washed with HCl (aqueous, 1 M, 20 mL) and brine (20 mL). Subsequently, the solution is dried over Na_2SO_4 and the volatiles are removed *in vacuo*. The crude product is purified by column chromatography (silica, CH_2Cl_2/MeOH = 98/2) to afford the product **SI-6**.

Colorless solid; 671 mg (844 µmol, 52%); **TLC**: R_f = 0.19 (CH_2Cl_2/MeOH = 96/4) [UV]; **^{1}H NMR** (500 MHz, $CDCl_3$, 298 K): δ = 7.51 (s, 2H, C^{3}NH), 7.02 (d$_{AB}$, $^3J_{H\text{-}H}$ = 8.4 Hz, 4H, H^{11}), 6.72 (d$_{AB}$, $^3J_{H\text{-}H}$ = 8.4 Hz, 4H, H^{12}), 5.38 (d, $^3J_{H\text{-}H}$ = 8.9 Hz, 2H, C^{5}NH), 4.77 (s, 2H, H^3), 4.57 (s, 2H, H^5), 3.74 (s, 6H, H^1), 3.20 (dd, $^2J_{H\text{-}H}$ = 14.0 Hz, $^3J_{H\text{-}H}$ = 4.0 Hz, 2H, H^{9a}), 2.94–2.84 (m, 6H, H^{9b}, H^{14}), 1.45 (s, 18H, H^8); **^{13}C{^{1}H} NMR** (126 MHz, $CDCl_3$, 298 K): δ = 171.9 (2C, C^2), 170.7 (2C, C^4), 155.8 (2C, C^6), 155.1 (2C, C^{13}), 130.5 (4C, C^{11}), 128.0 (2C, C^{10}), 116.0 (4C, C^{12}), 80.7 (2C, C^7), 54.2 (2C, C^3/C^5), 54.0 (2C, C^3/C^5), 52.7 (2C, C^1), 45.4 (2C, C^{14}), 36.8 (2C, C^9), 28.5 (6C, C^8).

The analytical data are in accordance with the literature.[40]

4.2 Di-*tert*-butyl ((4*S*,7*R*,12*R*,15*S*)-4,15-bis(4-hydroxybenzyl)-3,6,13,16-tetraoxo-9,10-dithia-2,5,14,17-tetraazaoctadecane-7,12-diyl)dicarbamate (SI-7)

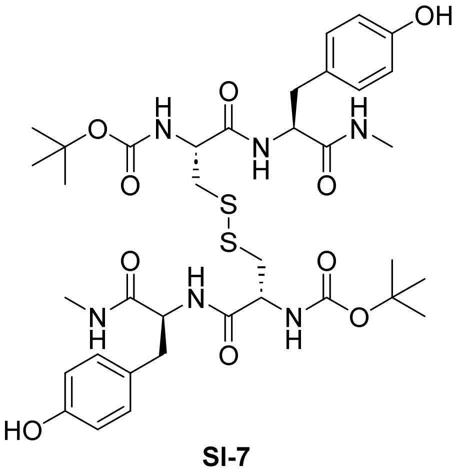

SI-7

Peptide **SI-6** (100 mg, 126 µmol, 1.00 equiv.) and cerium(III) chloride (1.55 mg, 6.29 µmol, 5 mol%) are dissolved in a solution of methylamine in THF (2 M, 0.63 mL, 39.1 mg, 1.26 mmol, 10.0 equiv.) and the solution is stirred for 20 h at r.t. The solvent is removed *in vacuo*. Peptide **SI-7** is obtained as a colorless solid without further purification and characterization.

4.3 *tert*-Butyl ((*R*)-1-(((*S*)-3-(4-hydroxyphenyl)-1-(methylamino)-1-oxopropan-2-yl)-amino)-3-mercapto-1-oxopropan-2-yl)carbamate (SI-8)

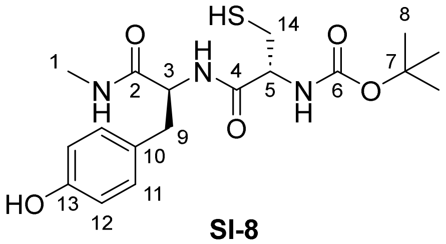

SI-8

According to a modified literature procedure,[40] peptide **SI-7** (99.8 mg, 126 µmol, 1.00 equiv.) and TCEP•HCl (72.1 mg, 252 µmol, 2.00 equiv.) are diluted in a mixture of MeOH/H_2O (4/1, 786 µL) at r.t. and the solution is stirred for 19 h, before all volatiles are removed *in vacuo*. The crude product is purified by column chromatography (silica, CH_2Cl_2/MeOH = 97/3) to afford the product **SI-8**.

Colorless solid; 85 mg (214 µmol, 85%); **TLC**: R_f = 0.68 (CH_2Cl_2/MeOH = 95/5) [UV]; **^{1}H NMR** (500 MHz, acetone–d_6, 298 K): δ = 8.22 (s, 1H, OH), 7.33 (d, $^3J_{H\text{-}H}$ = 8.2 Hz, 1H, C^3NH), 7.12 (br s, 1H, C^1NH), 7.03 (d_{AB}, $^3J_{H\text{-}H}$ = 8.4 Hz, 2H, H^{11}), 6.72 (d_{AB}, $^3J_{H\text{-}H}$ = 8.4 Hz, 2H, H^{12}), 6.32 (d, $^3J_{H\text{-}H}$ = 7.2 Hz, 1H, C^5NH), 4.55–4.50 (m, 1H, H^3), 4.18 (*virt.* q, $^3J_{H\text{-}H} \approx {}^3J_{H\text{-}H}$ = 6.2 Hz, 1H, H^5), 3.01–2.98 (m, 1H, H^{9a}), 2.91–2.82 (m, 3H, H^{9b}, H^{14}), 2.66 (d, $^3J_{H\text{-}H}$ = 4.7 Hz, 3H, H^1), 1.85 (*virt.* t, $^3J_{H\text{-}H} \approx {}^3J_{H\text{-}H}$ = 8.8 Hz, 1H, SH), 1.40 (s, 9H, H^8); **^{13}C{^{1}H} NMR** (126 MHz, acetone–d_6, 298 K): δ = 171.8 (C^2), 170.5 (C^4), 157.0 (C^6), 156.7 (C^{13}), 131.2 (2C, C^{11}), 128.9 (C^{10}), 115.9 (2C, C^{12}), 80.0 (C^7), 57.9 (C^5), 55.3 (C^3), 37.7 (C^9), 28.5 (3C, C^8), 27.0 (C^{14}), 26.0 (C^1); **HR-MS** (ESI^+): *m/z* = calc. for $[C_{18}H_{27}N_3NaO_5S]^+$: 420.1564 ($[M+Na]^+$), found: 420.1562; **IR** (ATR): $\tilde{\nu}_{max}$ [cm^{-1}] = 3330 (w, O–H), 3160 (w, C–H_{Ar}), 3025 (w, C–H_{Ar}), 2984 (w, C–H_{Ar}), 1681 (w, C=O), 1646 (s, C=O), 1618 (s, C–C_{Ar}), 1597 (s, C–C_{Ar}), 1575 (s, C–C_{Ar}), 1536 (s, C–C_{Ar}), 1518 (s, C–C_{Ar}), 1459 (s, C–C_{Ar}), 1415 (w), 1390 (w), 1367 (w), 1318 (w, C–N), 1284 (w, C–N), 1274 (w, C–N), 1239 (w), 1209 (w), 1193 (w), 1163 (w), 1110 (w), 1085 (w), 1046 (w), 1010 (w), 925 (w), 893 (w), 856 (w), 832 (w), 803 (w), 779 (w), 735 (w), 708 (w).

4.4 (Methoxy-d_3)diphenylphosphane (33)

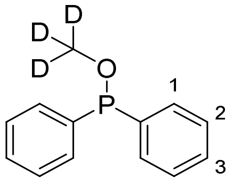

33

According to a modified literature procedure,[17] methanol-d_4 (406 µL, 361 mg, 10.0 mmol, 2.00 equiv.), pyridine (443 µL, 435 mg, 5.50 mmol, 1.10 equiv.) and triethylamine (836 µL, 607 mg, 6.00 mmol, 1.20 equiv.) are dissolved in anhydrous THF (10 mL, 500 mM) and chlorodiphenylphosphine (1.10 g, 5.00 mmol, 1.00 equiv) is added. The reaction mixture is stirred at r.t. for 1 h. The solvent is removed *in vacuo*. Subsequently, the reaction mixture is suspended in a mixture of P/EtOAc (9/1, 50 mL) and filtered under Ar atmosphere. The solvent is removed *in vacuo*. The product **33** is obtained as a colorless liquid in 91% purity without further purification and stored under Ar atmosphere. A yield was not determined due to the air sensitivity of the compound.

Colorless liquid; **^{1}H NMR** (400 MHz, $CDCl_3$, 298 K): δ = 7.53–7.47 (m, 4H, H^1), 7.41–7.34 (m, 6H, H^2, H^3); **^{31}P{^{1}H} NMR** (162 MHz, $CDCl_3$, 298 K): δ = 116.7 (hept, $^3J_{P\text{-}D}$ = 2.0 Hz).

5. Screening

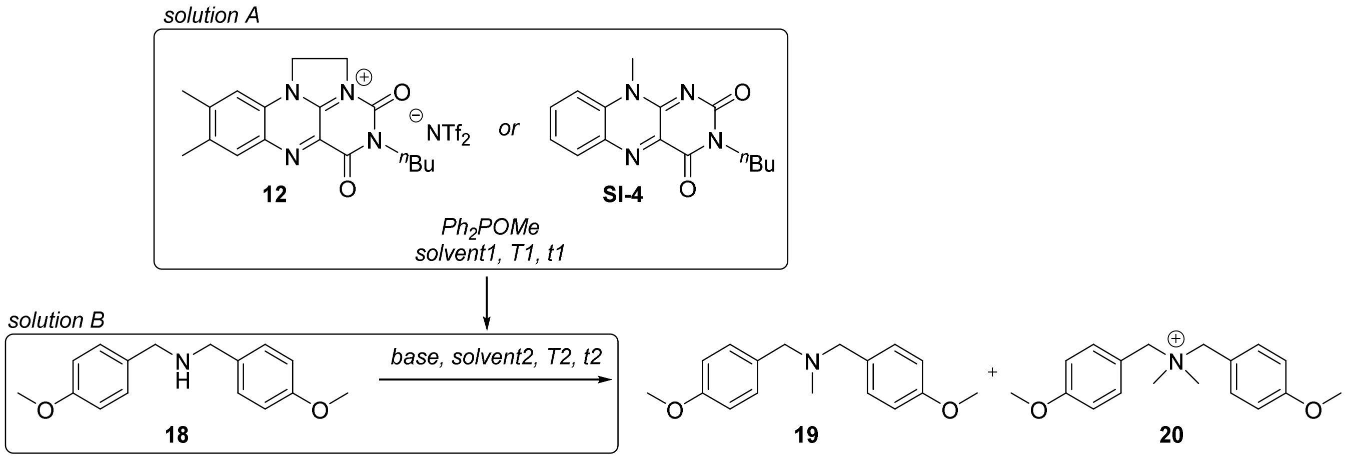

#	Fl (1.00 equiv.)	solv1 [mM]	T1 [°C]	t1 [min]	Base (equiv)	solv2 [mM]	T2 [°C]	t2 [h]	A→B[a]	18 [%]	19 [%]	20 [%]	11 [%]
**1**	**12**	MeCN (20)	r.t	10	DIPEA (1.00)	MeCN (20)	r.t.	o.n.	B→A (5min)	30	50	14	75
**2**	**12**	MeCN (20)	r.t.	10	DIPEA (1.00)	MeCN (20)	-30	o.n.	B→A (3min)	30	59	11	89
**3**	**12**	MeCN (20)	r.t	10	DIPEA (1.00) +3Å MS	MeCN (20)	r.t.	o.n.	B→A (5min)	53	19	13	14
**4**	**12**	MeCN (20)	r.t	10	--	MeCN (20)	-30→r.t.	o.n.	B→A (5min)	37	42	19	85
**5**	**12**	MeCN (20)	r.t	10	DBU (1.00)	MeCN (20)	r.t.	o.n.	B→A (5min)	38	33	13	77
**6**	**12**	MeCN (20)	r.t	10	Barton's Base (1.00)	MeCN (20)	r.t.	o.n.	B→A (10min)	41	40	8	67
**7**	**12**	MeCN (20)	r.t	10	2,6-Lutidine (1.00)	MeCN (20)	r.t.	o.n.	B→A (10min)	43	38	15	37
**8**	**12**	MeCN (20)	r.t	10	KO^{t}Bu (1.00)	MeCN (20)	r.t.	o.n.	B→A (10min)	29	51	15	85
**9**	**12**	CH_2Cl_2 (20)	r.t	10	DIPEA (1.00)	CH_2Cl_2 (20)	r.t.	o.n.	B→A (10min)	32	51	15	84
**10**	**12**	DMF (20)	r.t	10	DIPEA (1.00)	DMF (20)	r.t.	o.n.	B→A (10min)	59	31	9	56

[a] refers to which solution is added in which order and over which period of time [b] 50.0 µmol [c] 100 µmol ; blue entries depict the varied parameters, grey entries represent blank experiments

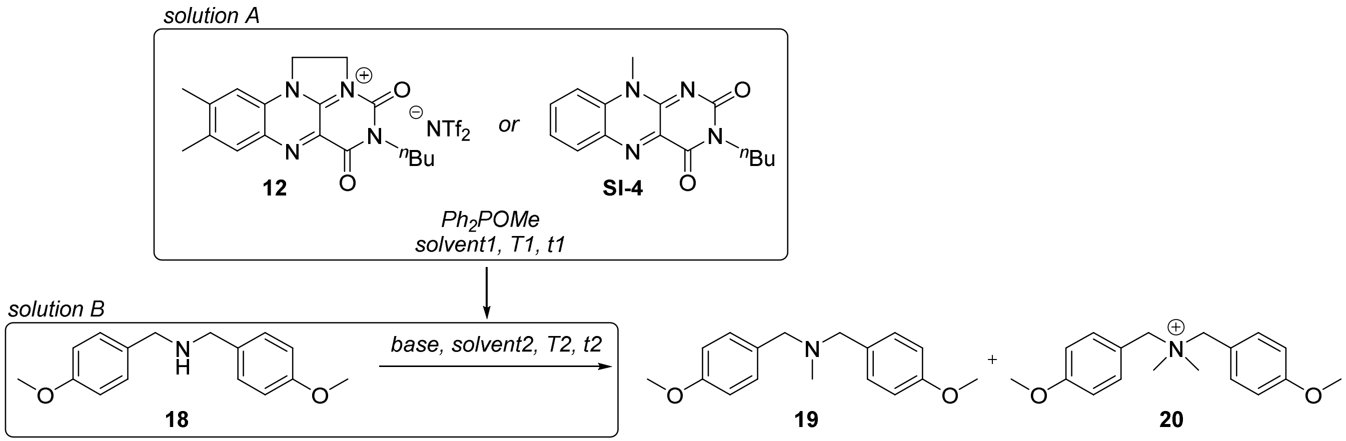

#	Fl (1.00 equiv.)	solv1 [mM]	T1 [°C]	t1 [min]	base	solv2 [mM]	T2 [°C]	t2 [h]	A→B[a]	18 [%]	19 [%]	20 [%]	11 [%]
**11**	**12**	MeCN (20)	r.t	10	DIPEA (1.00)	MeCN (20)	r.t.	4.75h	B→A (10min)	30	45	15	75
**12**	**12**	CH_2Cl_2 (20)	r.t	10	DIPEA (1.00)	CH_2Cl_2 (20)	r.t.	4.75h	B→A (10min)	30	60	15	89
**13**	**12**	MeCN (20)	r.t	10	DIPEA (1.00)	MeCN (20)	r.t.	o.n.	A→B (1h)	42	56	8	88
**14**	**12**	MeCN (20)	r.t	10	DIPEA (1.00)	MeCN (20)	r.t.	o.n.	A→B (30min)	29	56	7	79
**15**	**SI-4**	MeCN (20)	r.t	10	DIPEA (1.00)	MeCN (20)	r.t.	o.n.	A→B (30min)	88	0	0	0
**16**	**---**	MeCN (20)	r.t	10	DIPEA (1.00)	MeCN (20)	r.t.	4.5h	---	96	0	0	0

blue entries depict the varied parameters, grey entries represent blank experiments

6. Methylation Experiments

6.1 General Procedure A: Stoichiometric Methylation Reactions using Flavin reagent 12

Flavinium Reagent **12** (30.3 mg, 50.0 µmol, 1.00 equiv.) is placed in a crimp-cap vial equipped with a magnetic stirring bar, the vial is sealed with a septum safety cap and the atmosphere is exchanged with Ar (3×). Dichloromethane (2.50 mL, 20 mM) is added and the vial is placed in an ultrasonic bath to achieve homogenization. Subsequently, diphenyl phosphinite (10.0 µL, 10.8 mg, 50.0 µmol, 1.00 equiv.) is added and the solution is vigorously stirred for 30 min (solution **A**). In a second crimp-cap vial, the substrate (50.0 µmol, 1.00 equiv.) and cesium carbonate (24.4 mg, 75.0 µmol, 1.50 equiv.) are placed in a crimp-cap vial equipped with a magnetic stirring bar, the vial is sealed with a septum safety cap, the atmosphere is exchanged with Ar (3×) and dichloromethane (2.00 mL, 25 mM) or a mixture of dichloromethane and dimethyl sulfoxide (Σ = 2.00 mL, Σ = 25 mM) is added (solution **B**). Solution **A** is transferred to solution **B**, the vial of solution **A** is rinsed with CH_2Cl_2 (2×250 µL), and the reaction is stirred at r.t. After the completion of the reaction, the vial is opened, the solution is diluted with dichloromethane, transferred to a round bottom flask and all volatiles are removed *in vacuo*. The crude product is transferred to an NMR tube and a stock solution of the internal standard trimethyl 1,3,5-benzenetricarboxylate (8.33 µmol in 350 µL $CDCl_3$, relaxation time of the ^{1}H-spectrum is set to τ = 10 s, the integral of the signal at δ = 8.82 ppm is set to 0.50) is added. Yields and selectivities (*S*) are determined by comparing the obtained integrals.

6.2 *N*-(4-Methoxybenzyl)-1-(4-methoxyphenyl)-*N*-methylmethanamine (19)

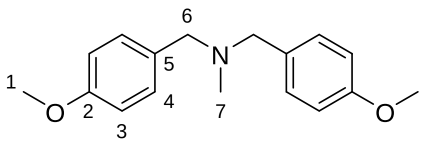

19

The reaction is performed according to general procedure **A** (reaction time 16.2 h; 62% NMR yield, 8% of side product **20** were detected) using bis(4-methoxybenzyl)amine (**18**) (12.9 mg, 50.0 µmol) as a substrate. In deviation from general procedure **A**, MeCN was used as the solvent instead of CH_2Cl_2, DIPEA (8.5 µL, 6.5 mg, 50.0 µmol, 1.00 equiv.) was used instead of cesium carbonate and solution **A** (reaction time 10 min) was added to solution **B** over the course of 30 min. The crude product is purified by column chromatography (silica, P/EtOAc = 70/30) to yield methylated product **19**.

Crude NMR with Internal Standard

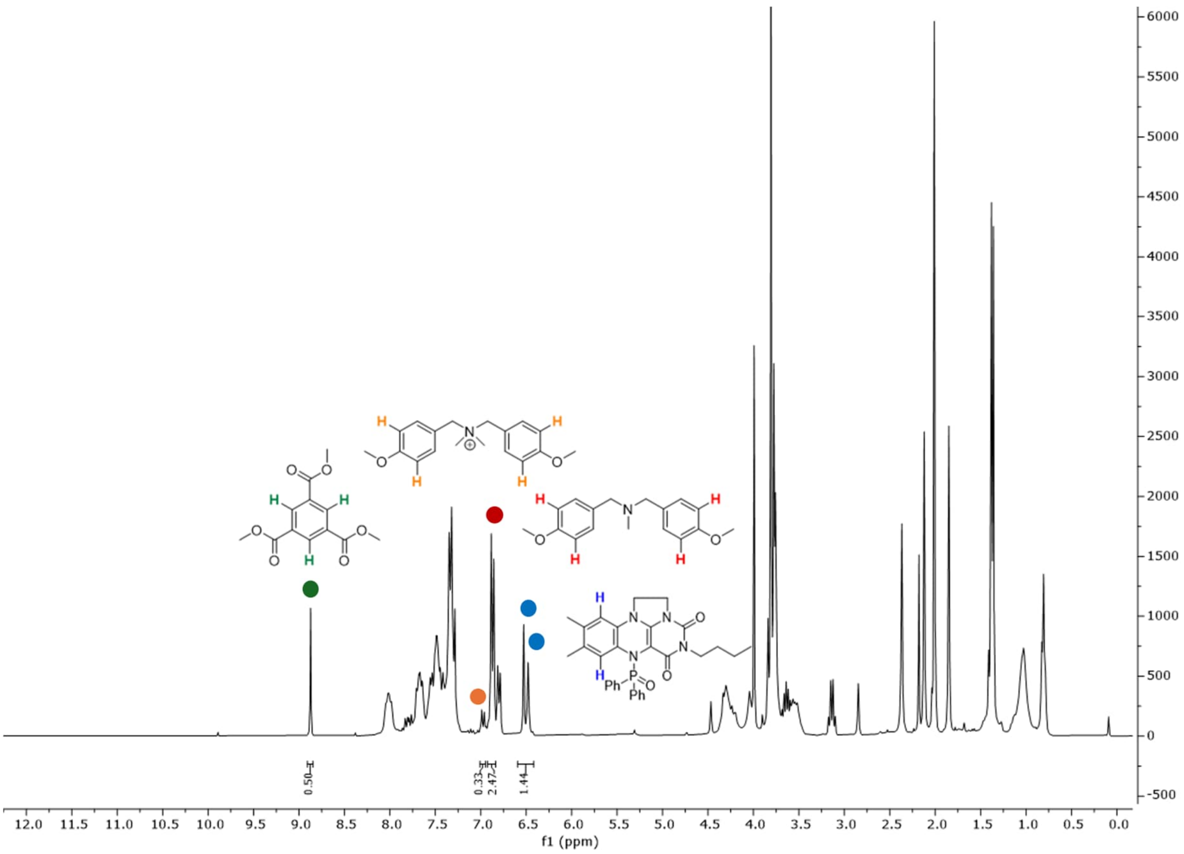

Colorless oil; 6.3 mg (23.2 µmol, 46%); **TLC**: R_f = 0.28 (P/EtOAc = 70/30) [UV]; **^{1}H NMR** (400 MHz, $CDCl_3$, 298 K): δ = 7.28–7.23 (m, 4H, H^4), 6.88–6.84 (m, 4H, H^3), 3.80 (s, 6H, H^1), 3.45 (s, 4H, H^6), 2.15 (s, 3H, H^7); **^{13}C{^{1}H} NMR** (101 MHz, $CDCl_3$, 298 K): δ = 158.8 (2C, C^2), 131.4 (2C, C^5), 130.3 (4C, C^4), 113.7 (4C, C^3), 61.2 (2C, C^6), 55.4 (2C, C^1), 42.0 (C^7).

The analytical data are in accordance with the literature.[41]

6.3 Methyl (*S*)-2-(6-methoxynaphthalen-2-yl)propanoate (22)

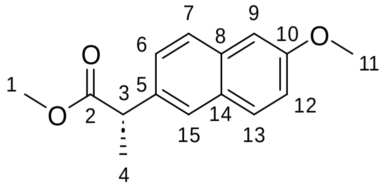

22

The reaction is performed according to general procedure **A** (reaction time 20 h; 75% NMR yield) using (*S*)-naproxen (11.5 mg, 50.0 µmol) as a substrate. The crude product is purified by column chromatography (silica, P/EtOAc = 95/5) to yield methylated product **22**.

Crude NMR with Internal Standard

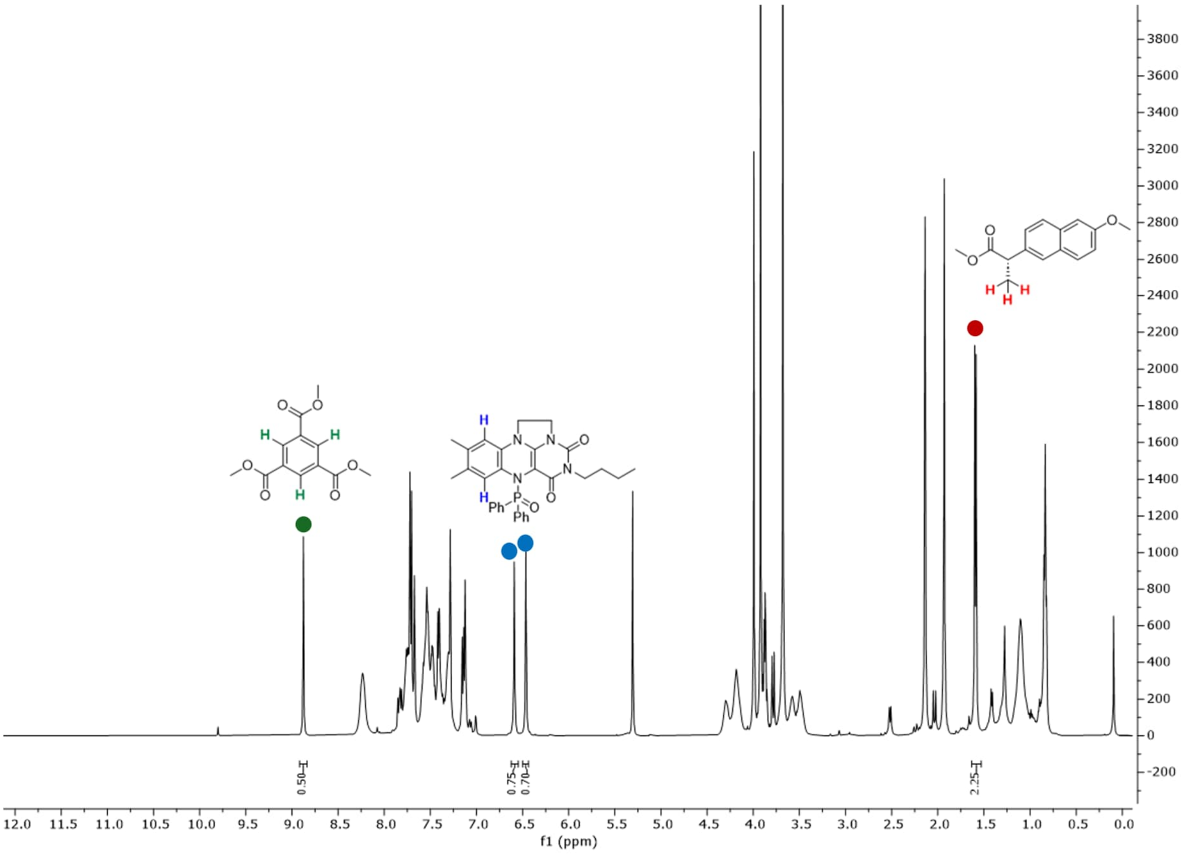

Colorless solid; 7.9 mg (32.4 µmol, 65%); **TLC**: R_f = 0.57 (P/EtOAc = 90/10) [UV]; **^{1}H NMR** (400 MHz, $CDCl_3$, 298 K): δ = 7.70 (2d, $^3J_{H\text{-}H}$ = 8.5 Hz, 2H, H^7, H^{13}), 7.66 (d, $^4J_{H\text{-}H}$ = 1.9 Hz, 1H, H^{15}), 7.40 (dd, $^3J_{H\text{-}H}$ = 8.5 Hz, $^4J_{H\text{-}H}$ = 1.9 Hz, 1H, H^6), 7.14 (dd, $^3J_{H\text{-}H}$ = 8.8 Hz, $^4J_{H\text{-}H}$ = 2.5 Hz, 1H, H^{12}), 7.11 (d, $^4J_{H\text{-}H}$ = 2.5 Hz, 1H, H^9), 3.91 (s, 3H, H^{11}), 3.86 (q, $^3J_{H\text{-}H}$ = 7.1 Hz, 1H, H^3), 3.67 (s, 3H, H^1), 1.58 (d, $^3J_{H\text{-}H}$ = 7.1 Hz, 3H, H^4); **^{13}C{^{1}H} NMR** (101 MHz, $CDCl_3$, 298 K): δ = 175.3 (C^2), 157.8 (C^{10}), 135.8 (C^5), 133.9 (C^8), 129.4 (C^7/C^{13}),

129.1 (C^{14}), 127.3 (C^{7}/C^{13}), 126.3 (C^{6}), 126.1 (C^{15}), 119.1 (C^{12}), 105.8 (C^{9}), 55.5 (C^{11}), 52.2 (C^{1}), 45.5 (C^{3}), 18.7 (C^{4}).

The analytical data are in accordance with the literature.[42]

The same reaction was performed with an increased amount of flavinium reagent **12** (42.4 mg, 70.0 µmol, 1.40 equiv.) and methyl diphenylphosphinite (14.0 µL, 15.1 mg, 70.0 µmol, 1.40 equiv.) and an NMR yield of product **22** was determined to be 91%.

Crude NMR with Internal Standard

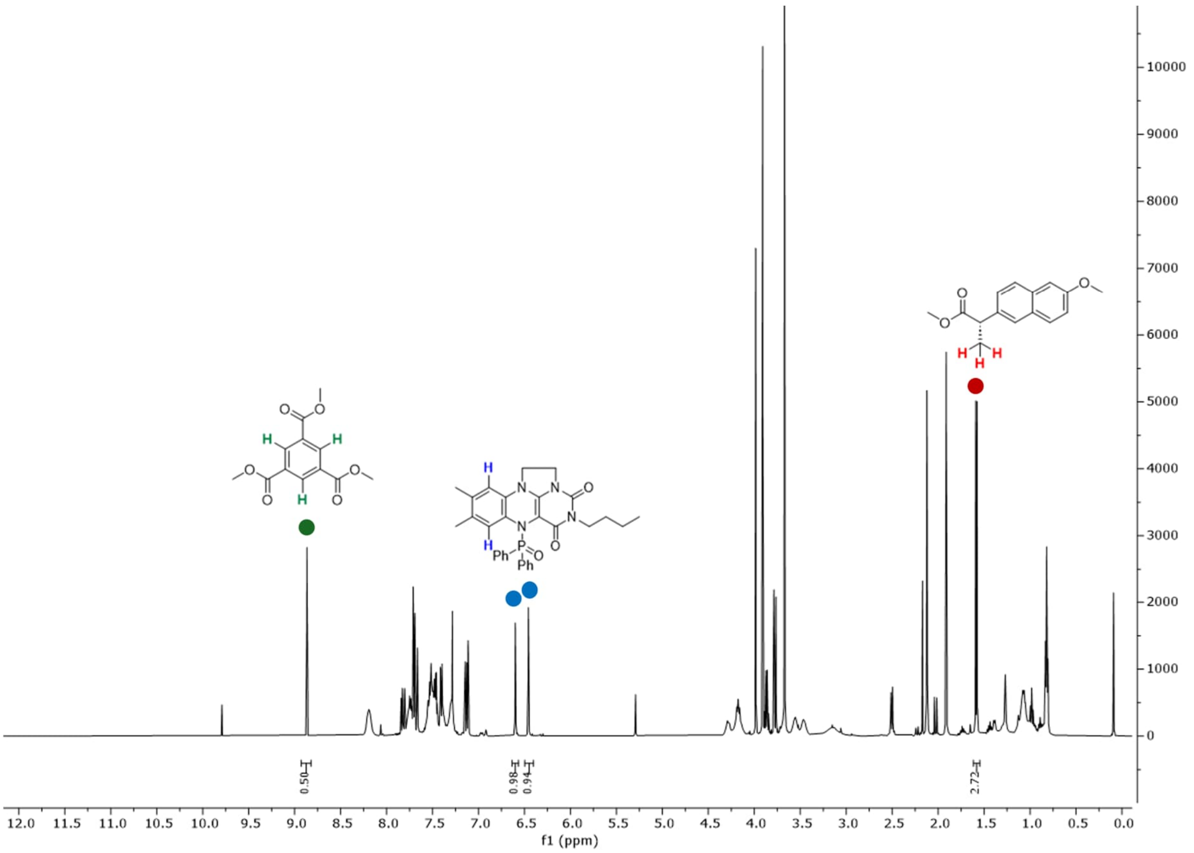

6.4 Methyl lithocholate (24)

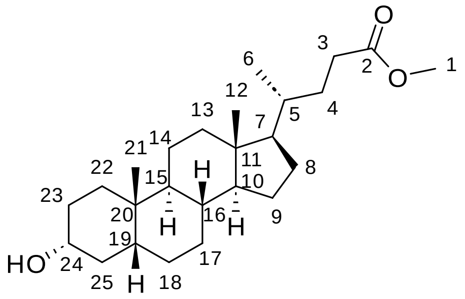

24

The reaction is performed according to general procedure **A** (reaction time 15.8 h; 42% NMR yield) using lithocholic acid (18.8 mg, 50.0 µmol) as a substrate in a solvent mixture of CH_2Cl_2 (1.90 mL) and DMSO (100 µL). The crude product is purified by column chromatography (silica, P/EtOAc = 85/15) to yield methylated product **24**.

Crude NMR with Internal Standard

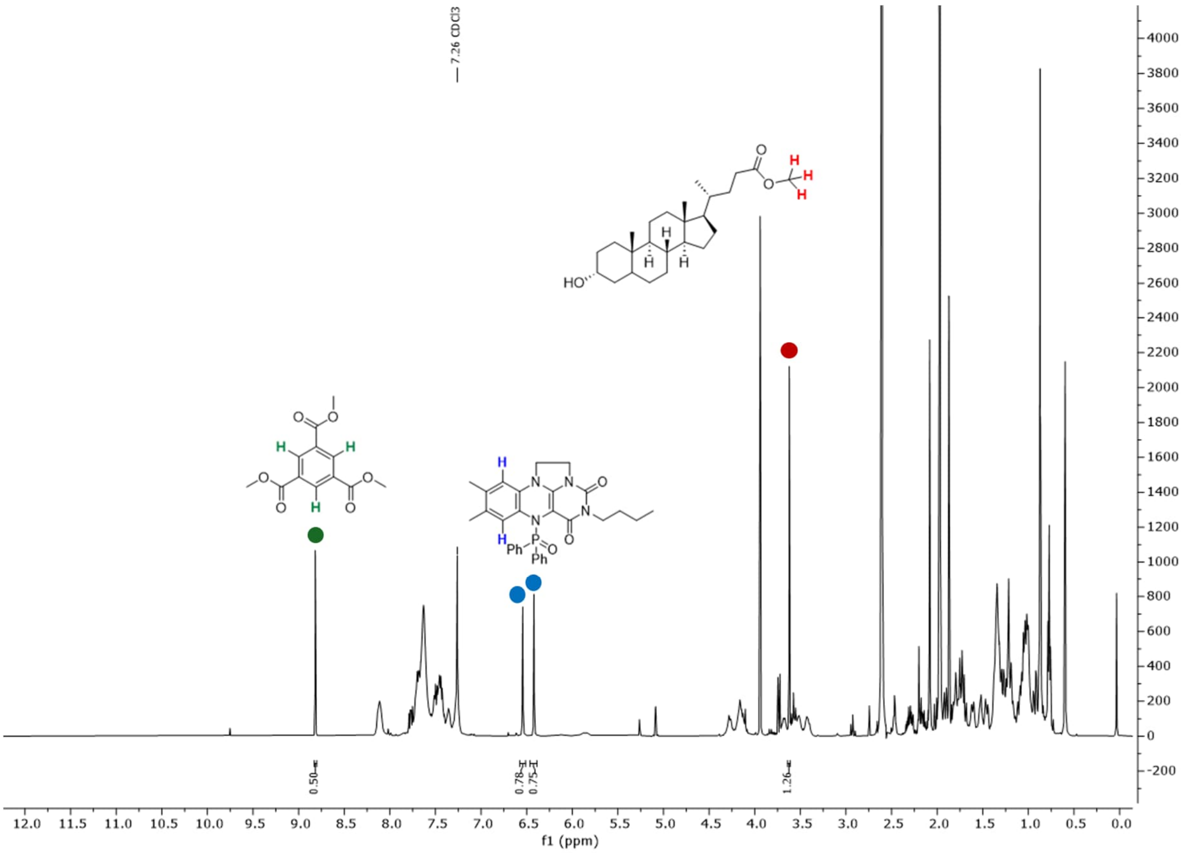

*crude NMR contains 100µL AcOH in order to solubilize the remaining starting material.

Colorless oil; 7.8 mg (20.0 µmol, 40%); **TLC**: R_f = 0.13 (P/EtOAc = 85/15) [$KMnO_4$]; **^{1}H NMR** (500 MHz, $CDCl_3$, 298 K): δ = 3.66 (s, 3H, H^1), 3.62 (*virt.* tt, $^3J_{H\text{-}H} \approx {}^3J_{H\text{-}H}$ = 11.0 Hz, $^3J_{H\text{-}H} \approx {}^3J_{H\text{-}H}$ = 5.0 Hz, 1H, H^{24}), 2.35 (ddt, $^2J_{H\text{-}H}$ = 15.4 Hz, $^3J_{H\text{-}H}$ = 10.2 Hz, $^3J_{H\text{-}H}$ = 5.1 Hz,

1H, H^{3a}), 2.25–2.17 (m, 1H, H^{3b}), 1.95 (dt, $^2J_{H\text{-}H}$ = 12.3 Hz, $^3J_{H\text{-}H}$ = 3.1Hz, 1H, $H^4/H^5/H^7/H^8/H^9/H^{13}/H^{14}/H^{15}/H^{16}/H^{17}/H^{18}/H^{19}/H^{22}$), 1.89–1.71 (m, 5H, H^{23a}), 1.66 (ddd, $^2J_{H\text{-}H}$ = 12.4 Hz, $^3J_{H\text{-}H}$ = 4.6 Hz, $^3J_{H\text{-}H}$ = 2.6 Hz, 1H, H^{25a}), 1.60–1.54 (m, 1H, $H^4/H^5/H^7/H^8/H^9/H^{13}/H^{14}/H^{15}/H^{16}/H^{17}/H^{18}/H^{19}/H^{22}$), 1.50 (dtd, $^2J_{H\text{-}H}$ = 12.6 Hz, $^3J_{H\text{-}H}$ = 4.4 Hz, $^3J_{H\text{-}H}$ = 2.3 Hz, 1H, H^{23b}), 1.47–1.01 (m, 17H, $H^4/H^5/H^7/H^8/H^9/H^{13}/H^{14}/H^{15}/H^{16}/H^{17}/H^{18}/H^{19}/H^{22}$, H^{25b}), 0.96 (td, $^2J_{H\text{-}H}$ = 14.1 Hz, $^3J_{H\text{-}H} \approx {}^3J_{H\text{-}H}$ = 3.3 Hz, 1H, $H^4/H^5/H^7/H^8/H^9/H^{13}/H^{14}/H^{15}/H^{16}/H^{17}/H^{18}/H^{19}/H^{22}$), 0.91 (s, d, $^3J_{H\text{-}H}$ = 6.7Hz, 6H, H^{12}/H^{21}, H^6), 0.64 (s, 3H, H^{12}/H^{21}); **^{13}C{^{1}H} NMR** (101 MHz, $CDCl_3$, 298 K): δ = 174.9 (C^2), 72.0 (C^{24}), 56.7 ($C^4/C^5/C^7/C^8/C^9/C^{11}/C^{12}/C^{13}/C^{14}/C^{15}/C^{16}/C^{17}/C^{18}/C^{19}/C^{20}/C^{22}$), 56.1 ($C^4/C^5/C^7/C^8/C^9/C^{11}/C^{12}/C^{13}/C^{14}/C^{15}/C^{16}/C^{17}/C^{18}/C^{19}/C^{20}/C^{22}$), 51.6 ($C^1$), 42.9 ($C^4/C^5/C^7/C^8/C^9/C^{11}/C^{12}/C^{13}/C^{14}/C^{15}/C^{16}/C^{17}/C^{18}/C^{19}/C^{20}/C^{22}$), 42.9 ($C^4/C^5/C^7/C^8/C^9/C^{11}/C^{12}/C^{13}/C^{14}/C^{15}/C^{16}/C^{17}/C^{18}/C^{19}/C^{20}/C^{22}$), 42.3 ($C^4/C^5/C^7/C^8/C^9/C^{11}/C^{12}/C^{13}/C^{14}/C^{15}/C^{16}/C^{17}/C^{18}/C^{19}/C^{20}/C^{22}$), 40.6 ($C^4/C^5/C^7/C^8/C^9/C^{11}/C^{12}/C^{13}/C^{14}/C^{15}/C^{16}/C^{17}/C^{18}/C^{19}/C^{20}/C^{22}$), 40.3 ($C^4/C^5/C^7/C^8/C^9/C^{11}/C^{12}/C^{13}/C^{14}/C^{15}/C^{16}/C^{17}/C^{18}/C^{19}/C^{20}/C^{22}$), 36.6 ($C^{23}$), 36.0 ($C^4/C^5/C^7/C^8/C^9/C^{11}/C^{12}/C^{13}/C^{14}/C^{15}/C^{16}/C^{17}/C^{18}/C^{19}/C^{20}/C^{22}$), 35.5 ($C^4/C^5/C^7/C^8/C^9/C^{11}/C^{12}/C^{13}/C^{14}/C^{15}/C^{16}/C^{17}/C^{18}/C^{19}/C^{20}/C^{22}$), 35.5 ($C^4/C^5/C^7/C^8/C^9/C^{11}/C^{12}/C^{13}/C^{14}/C^{15}/C^{16}/C^{17}/C^{18}/C^{19}/C^{20}/C^{22}$), 34.7 ($C^4/C^5/C^7/C^8/C^9/C^{11}/C^{12}/C^{13}/C^{14}/C^{15}/C^{16}/C^{17}/C^{18}/C^{19}/C^{20}/C^{22}$), 31.2 ($C^3$), 31.2 ($C^4/C^5/C^7/C^8/C^9/C^{11}/C^{12}/C^{13}/C^{14}/C^{15}/C^{16}/C^{17}/C^{18}/C^{19}/C^{20}/C^{22}$), 30.7 ($C^{25}$), 28.3 ($C^4/C^5/C^7/C^8/C^9/C^{11}/C^{12}/C^{13}/C^{14}/C^{15}/C^{16}/C^{17}/C^{18}/C^{19}/C^{20}/C^{22}$), 27.3 ($C^4/C^5/C^7/C^8/C^9/C^{11}/C^{12}/C^{13}/C^{14}/C^{15}/C^{16}/C^{17}/C^{18}/C^{19}/C^{20}/C^{22}$), 26.6 ($C^4/C^5/C^7/C^8/C^9/C^{11}/C^{12}/C^{13}/C^{14}/C^{15}/C^{16}/C^{17}/C^{18}/C^{19}/C^{20}/C^{22}$), 24.4 ($C^4/C^5/C^7/C^8/C^9/C^{11}/C^{12}/C^{13}/C^{14}/C^{15}/C^{16}/C^{17}/C^{18}/C^{19}/C^{20}/C^{22}$), 23.5 ($C^{12}/C^{21}$), 21.0 ($C^4/C^5/C^7/C^8/C^9/C^{11}/C^{12}/C^{13}/C^{14}/C^{15}/C^{16}/C^{17}/C^{18}/C^{19}/C^{20}/C^{22}$), 18.4 ($C^6$), 12.2 ($C^{12}/C^{21}$).

The analytical data are in accordance with the literature.[43]

Indicative HMBC-contacts for determination of the chemoselectivity

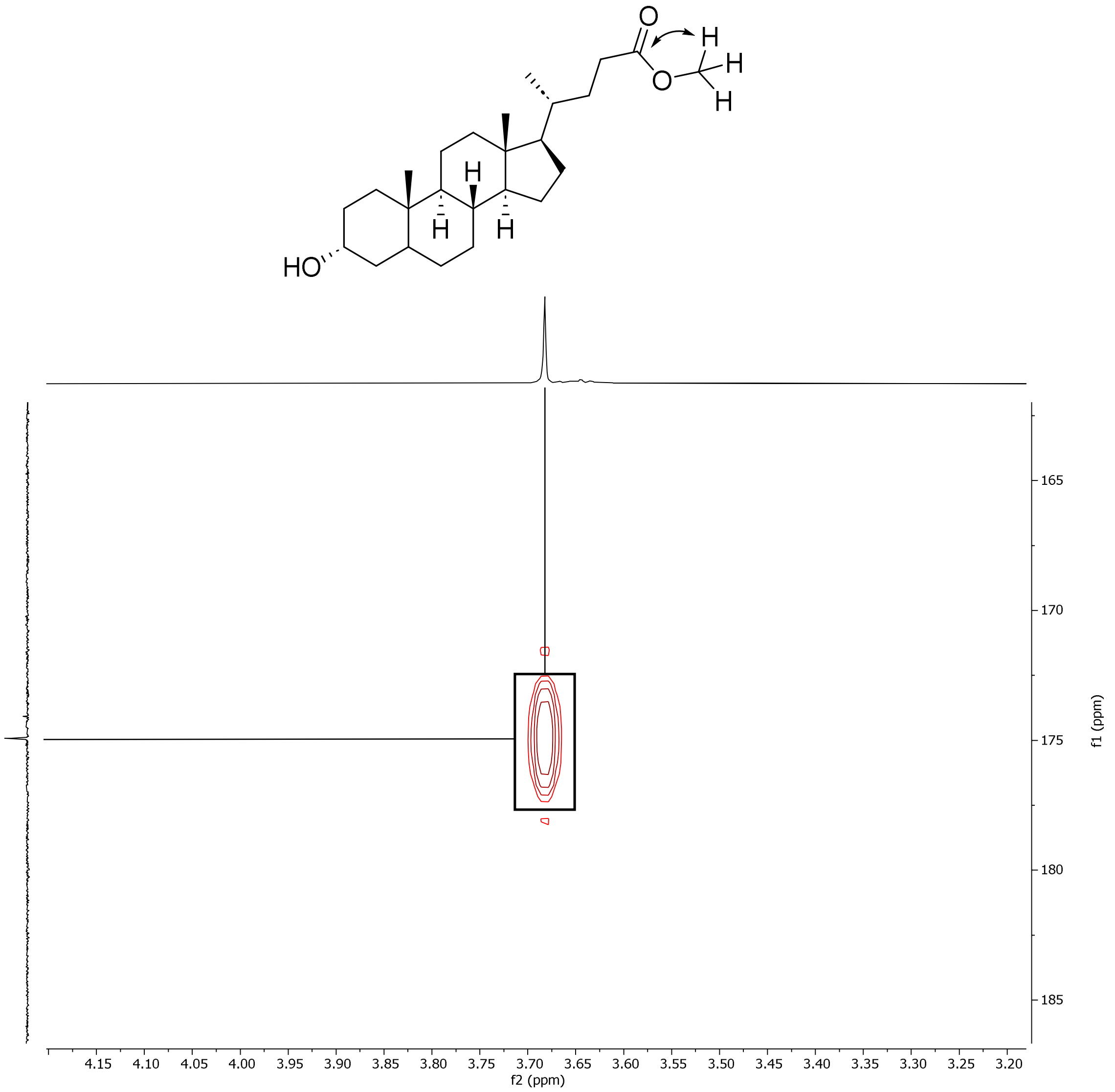

6.5 Methyl (*E*)-ferulate (25)

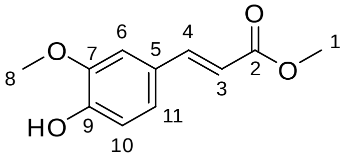

25

The reaction is performed according to general procedure **A** (reaction time 19.2 h; 49% NMR yield) using (*E*)-ferulic acid (9.7 mg, 50.0 µmol) as a substrate in a solvent mixture of CH_2Cl_2 (1.50 mL) and DMSO (500 µL). The crude product is purified by column chromatography (silica, P/EtOAc = 80/20) to yield methylated product **25**.

Crude NMR with Internal Standard

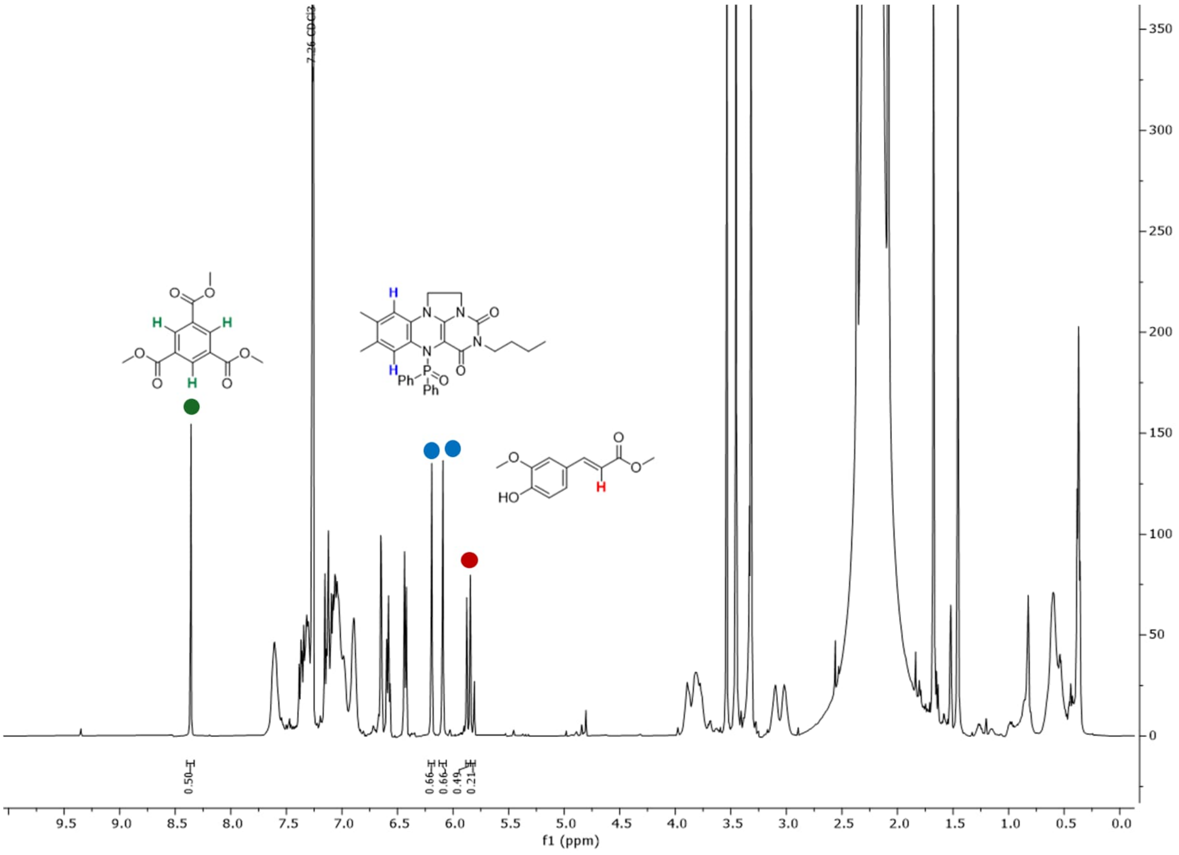

*crude NMR contains 100µL AcOH in order to solubilize the remaining starting material.

Colorless solid; 4.4 mg (21.1 µmol, 42%); **TLC**: R_f = 0.21 (P/EtOAc = 80/20) [UV]; **^{1}H NMR** (400 MHz, $CDCl_3$, 298 K): δ = 7.62 (d, $^3J_{H\text{-}H}$ = 15.9 Hz, 1H, H^4), 7.08 (dd, $^3J_{H\text{-}H}$ = 8.2 Hz, $^4J_{H\text{-}H}$ = 1.9 Hz, 1H, H^{11}), 7.03 (d, $^4J_{H\text{-}H}$ = 1.9 Hz, 1H, H^6), 6.92 (d, $^3J_{H\text{-}H}$ = 8.2 Hz, 1H, H^{10}), 6.29 (d, $^3J_{H\text{-}H}$ = 15.9 Hz, 1H, H^3), 5.86 (s, 1H, OH), 3.93 (s, 3H, H^8), 3.80 (s, 3H, H^1); **^{13}C{^{1}H} NMR** (101 MHz, $CDCl_3$, 298 K): δ = 167.9 (C^2), 148.1 (C^9), 146.9

(C[7]), 145.1 (C[4]), 127.1 (C[5]), 123.2 (C[11]), 115.4 (C[3]), 114.9 (C[10]), 109.5 (C[6]), 56.1 (C[8]), 51.8 (C[1]).

The analytical data are in accordance with the literature.[44]

Indicative HMBC-contacts for determination of the chemoselectivity

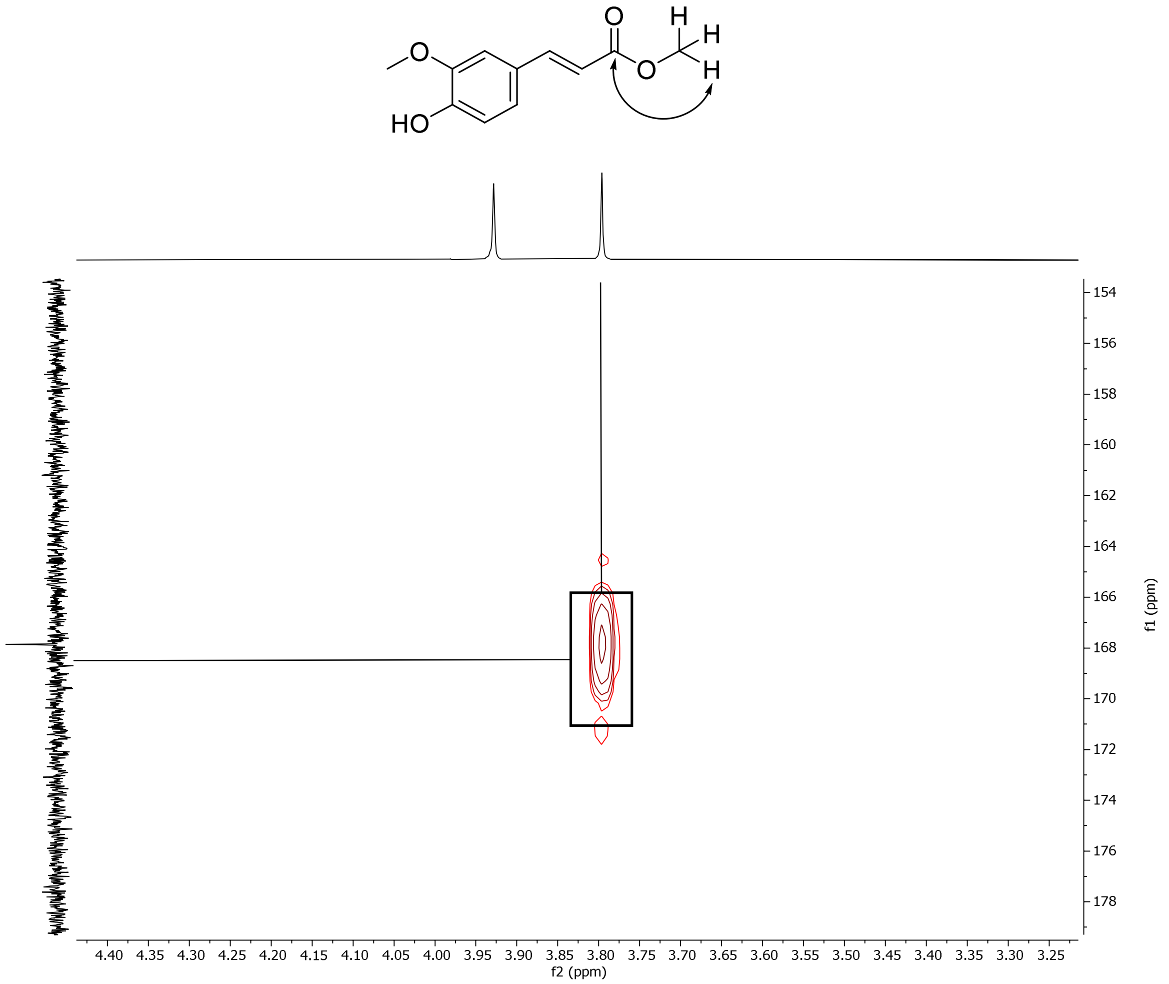

6.6 Comparison with the Methylation methodology of Wang *et al.*[17]

Methyl (*E*)-3-(3,4-dimethoxyphenyl)acrylate (SI-9)

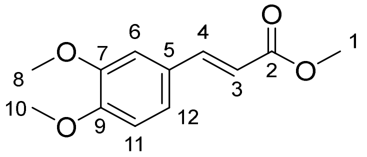

SI-9

The reaction was performed according to the literature procedure reported by Wang *et al.*, using either the reported 2.50 equiv. of the methylating reagents or 1.00 equiv.[17] (*E*)-ferulic acid (38.8 mg, 200 µmol) is placed in a screw cap *Schlenk* tube under Ar atmosphere and a solvent mixture of CH_2Cl_2/$PhCF_3$ (1/1, 1.00 mL, 800 mM) is added. Ethyl acrylate (51.0 µL, 50.1 mg, 500 µmol, 2.50 equiv or 20.4 µL, 20.0 mg, 200 µmol, 1.00 equiv.) and methyl diphenlyphosphinite (100 µL, 108 mg, 500 µmol, 2.50 equiv. or 40.0 µL, 43.2 mg, 200 µmol, 1.00 equiv.) are added, the tube is closed and the reaction is stirred at 80°C (18.5 hor 16 h). After the completion of the reaction, the tube is opened, the solution is diluted with dichloromethane, transferred to a round bottom flask and all volatiles are removed *in vacuo*. The crude product is transferred to an NMR tube and a stock solution of the internal standard trimethyl 1,3,5-benzenetricarboxylate (8.33 µmol in 350 µL $CDCl_3$, relaxation time of the ^{1}H-spectrum is set to τ = 10 s, the integral of the signal at δ = 8.82 ppm is set to 0.125) is added (83% NMR yield for dimethylated product **SI-9** in the reaction using 2.50 equiv. methylating reagents). The dimethylated product **SI-9** was isolated by column chromatography (silica, P/EtOAc = 90/10) in a separate experiment using 2.50 equiv. of the methylating reagents.

Crude NMR with Internal Standard using 2.50 equiv. of Methylating Reagents

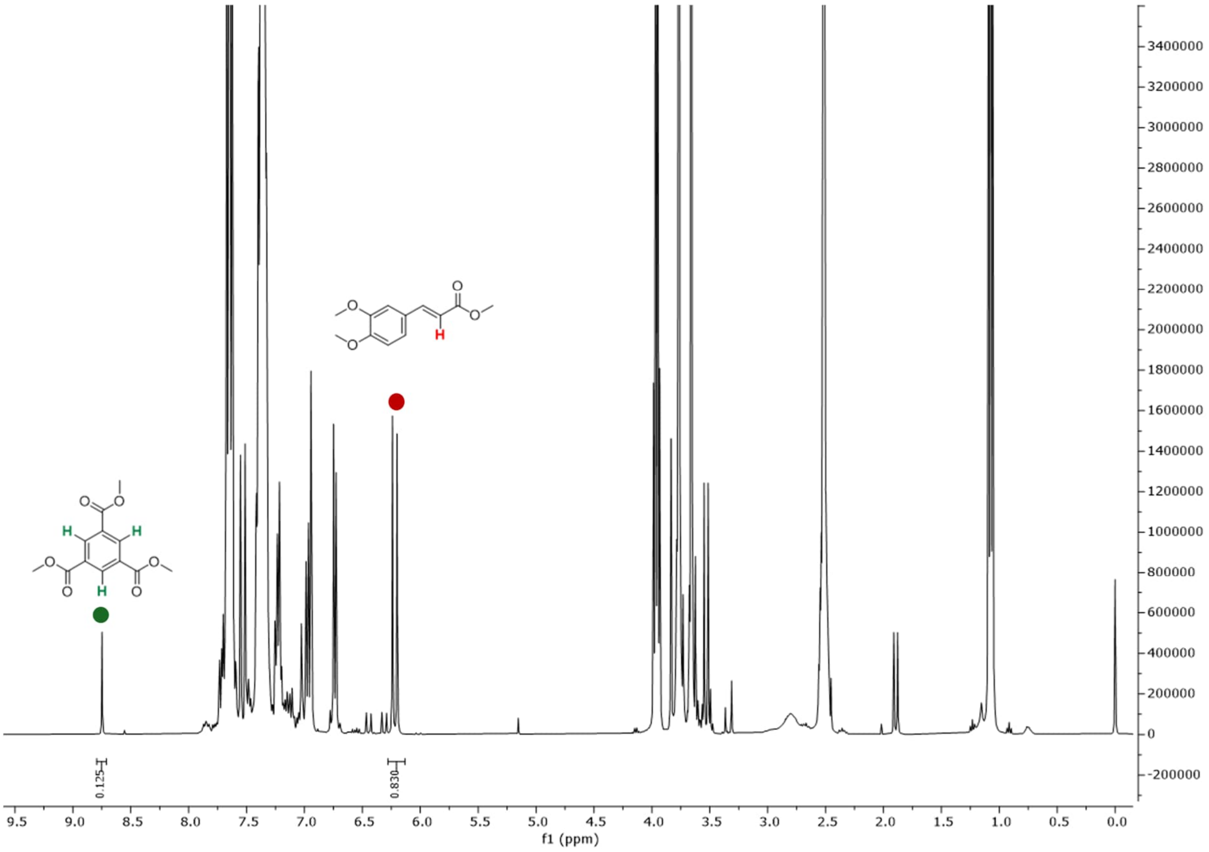

Colorless, crystalline solid; 37.0 mg (166 µmol, 83%); **TLC**: R_f = 0.25 (P/EtOAc = 90/10) [UV]; **^{1}H NMR** (400 MHz, $CDCl_3$, 298 K): δ = 7.64 (d, $^3J_{H-H}$ = 16.0 Hz, 1H, H^4), 7.11 (dd, $^3J_{H-H}$ = 8.3 Hz, $^4J_{H-H}$ = 2.0 Hz, 1H, H^{12}), 7.05 (d, $^4J_{H-H}$ = 2.0 Hz, 1H, H^6), 6.87 (d, $^3J_{H-H}$ = 8.3 Hz, 1H, H^{11}), 6.31 (d, $^3J_{H-H}$ = 16.0 Hz, 1H, H^3), 3.91 (2s, 6H, H^8, H^{10}), 3.80 (s, 3H, H^1); **^{13}C{^{1}H} NMR** (101 MHz, $CDCl_3$, 298 K): δ = 167.8 (C^2), 151.3 (C^9), 149.4 (C^7), 144.9 (C^4), 127.5 (C^5), 122.7 (C^{12}), 115.7 (C^3), 111.2 (C^{11}), 109.8 (C^6), 56.1 (C^8/C^{10}), 56.0 (C^8/C^{10}), 51.8 (C^1).

The analytical data are in accordance with the literature.[44]

Crude NMR with Internal Standard using 1.00 equiv. of Methylating Reagents

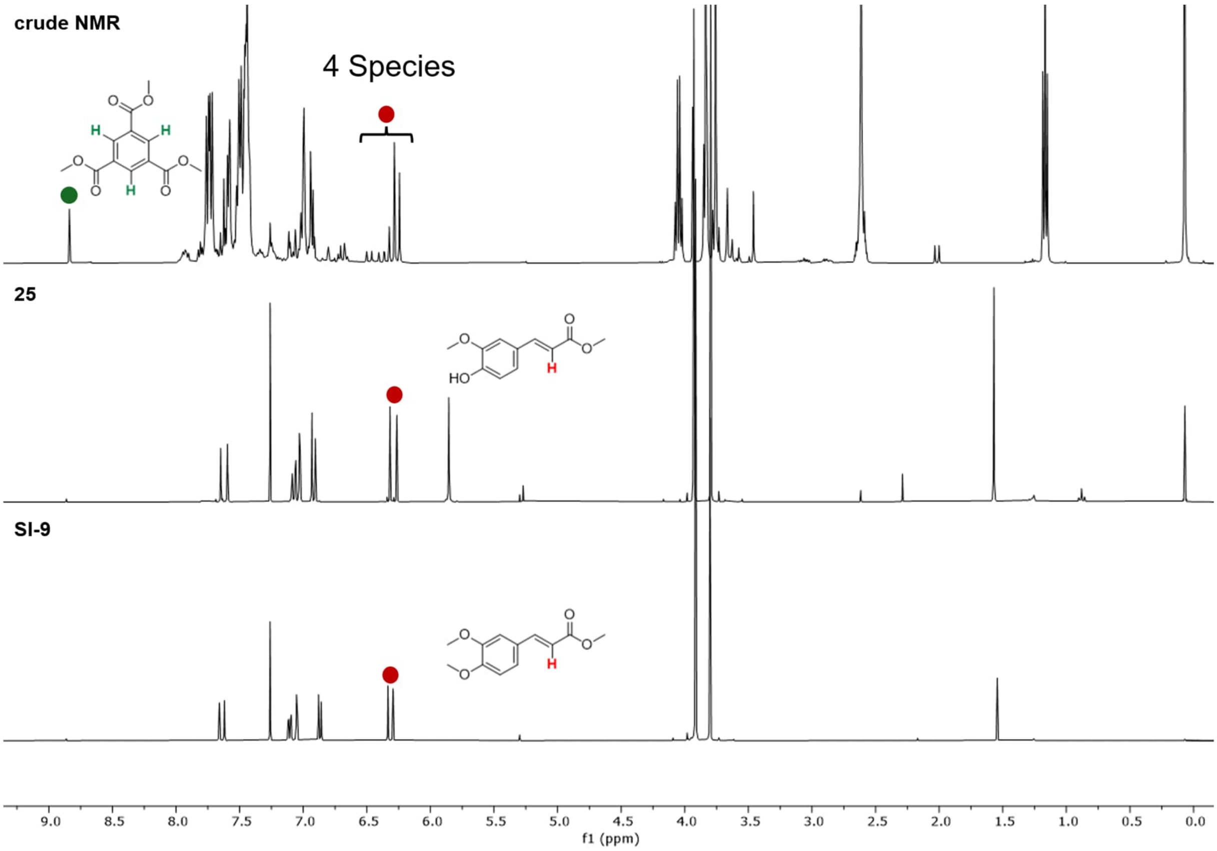

The crude NMR using 1.00 equiv. of methylating reagents shows four distinct species (9%, 10%, 22%, 43%), presumably corresponding to the four possible not-, mono-, or dimethylated species listed below. A clear assignment is not possible according to the crude NMR.

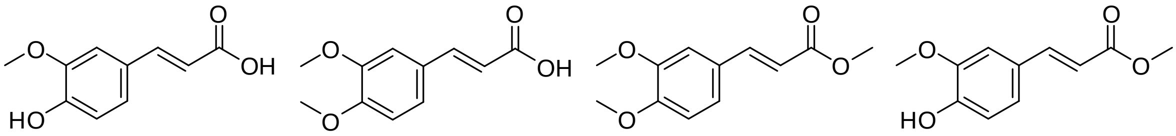

(*E*)-ferulic acid **SI-9** **25**

6.7 4-Amino-*N*-methyl-*N*-(5-methylisoxazol-3-yl)benzenesulfonamide (26)

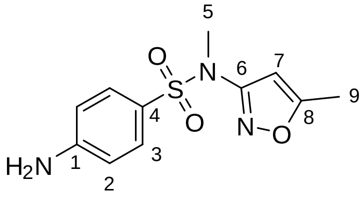

26

The reaction is performed according to general procedure **A** (reaction time 15.8 h; 46% NMR yield) using sulfamethoxazole (12.7 mg, 50.0 µmol) as a substrate. The crude product is purified by column chromatography (silica, P/EtOAc = 70/30) to yield methylated product **26**.

Crude NMR with Internal Standard

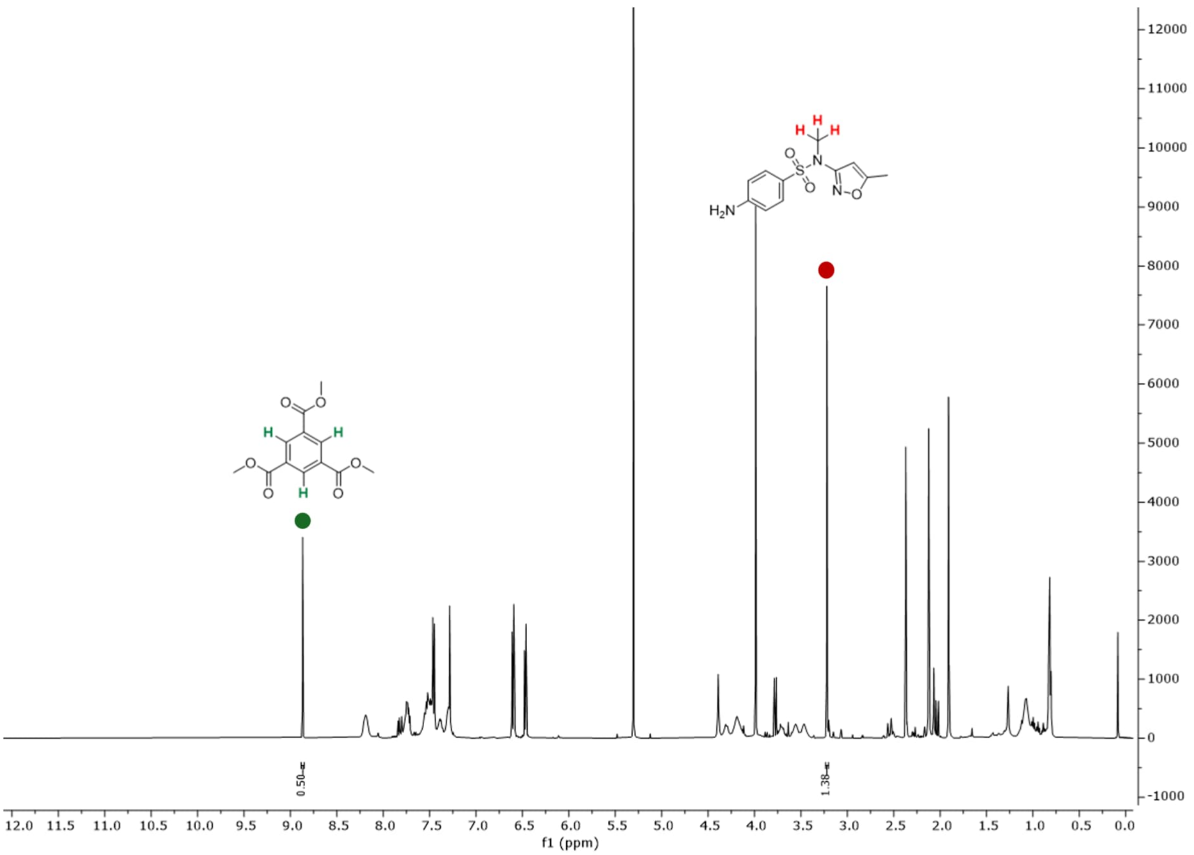

Yellow solid; 6.1 mg (22.8 µmol, 46%); **TLC**: R_f = 0.15 (P/EtOAc = 70/30) [UV]; **^{1}H NMR** (400 MHz, $CDCl_3$, 298 K): δ = 7.54–7.43 (m, 2H, H^3), 6.66–6.56 (m, 2H, H^2), 6.48 (q, $^3J_{H\text{-}H}$ = 0.7 Hz, 1H, H^7), 3.22 (s, 3H, H^5), 2.37 (d, 3H, $^3J_{H\text{-}H}$ = 0.7 Hz, H^9); **^{13}C{^{1}H} NMR** (101 MHz, $CDCl_3$, 298 K): δ = 170.2 (C^8), 161.3 (C^6), 151.4 (C^1), 129.5 (2C, C^3), 125.1 (C^4), 114.2 (2C, C^2), 97.8 (C^7), 35.1 (C^5), 12.8 (C^9).

The analytical data are in accordance with the literature.[45]

Indicative HMBC-contacts for determination of the chemoselectivity

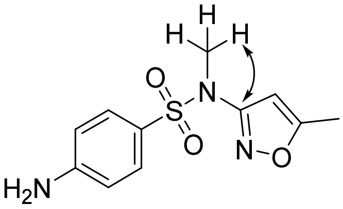

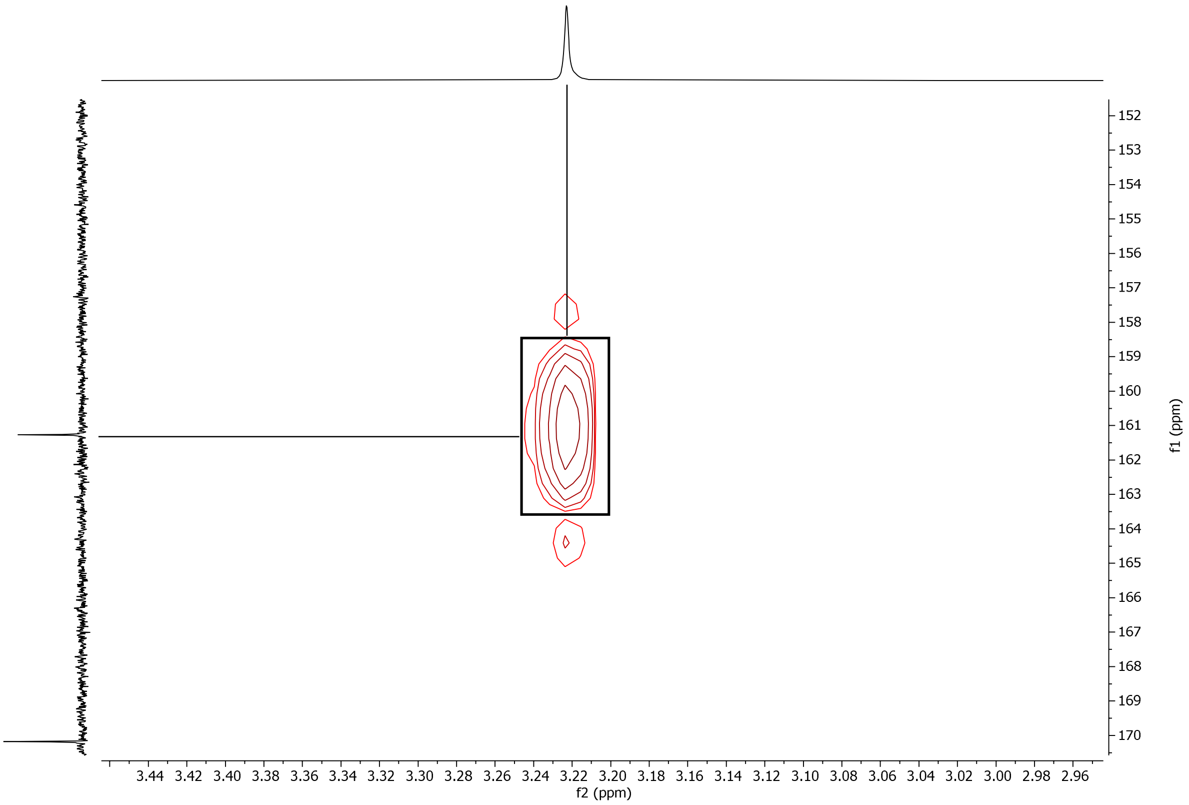

6.8 1-((2*R*,4*S*,5*R*)-4-Hydroxy-5-(hydroxymethyl)tetrahydrofuran-2-yl)-3-methyl-pyrimidine-2,4(1*H*,3*H*)-dione (27)

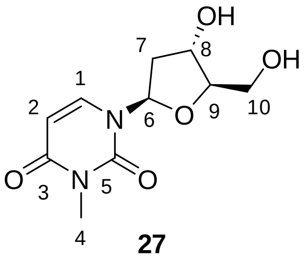

27

The reaction is performed according to general procedure **A** (reaction time 23 h; 45% NMR yield) using 2’-deoxyuridine (11.4 mg, 50.0 µmol) as a substrate in a solvent mixture of CH_2Cl_2 (1.50 mL) and DMSO (500 µL). The crude product is purified by column chromatography (silica, CH_2Cl_2/MeOH = 95/5) to yield methylated product **27**.

Crude NMR with Internal Standard

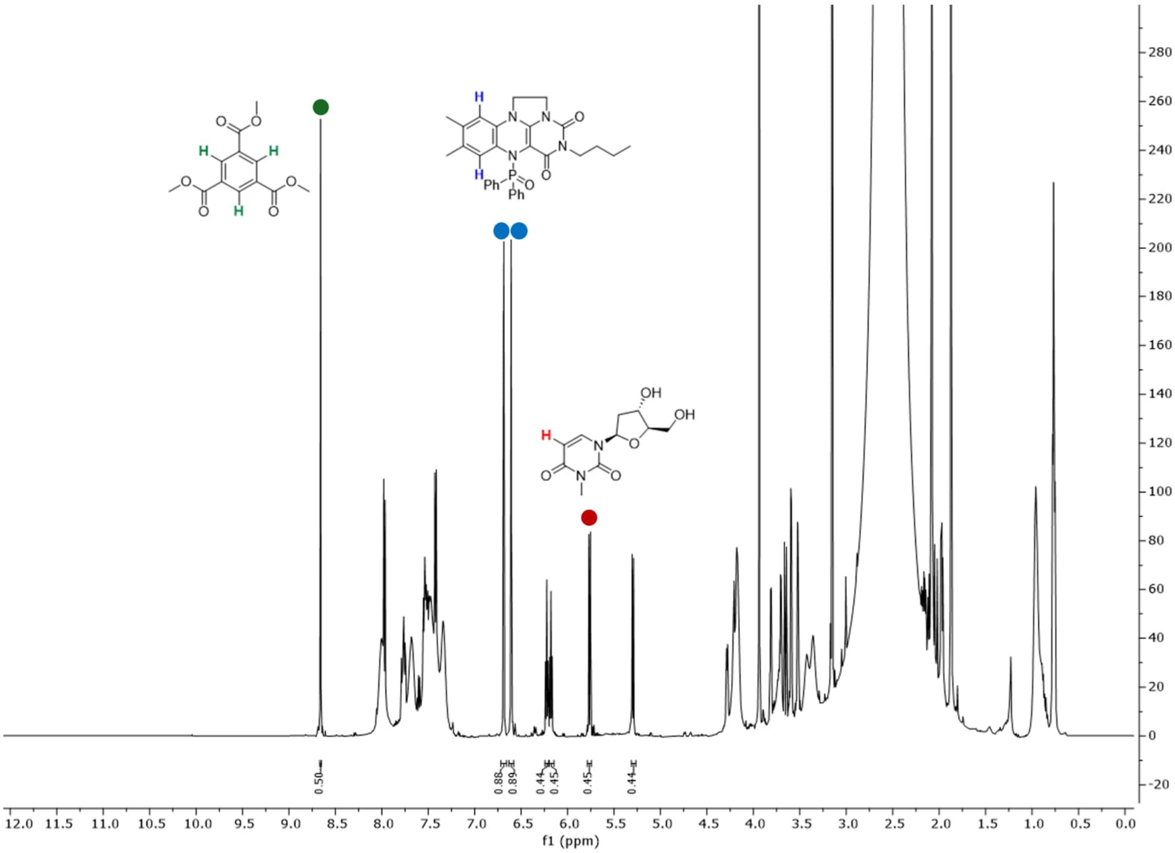

Off-white solid; 5.1 mg (21.1 µmol, 42%); **TLC**: R_f = 0.32 (CH_2Cl_2/MeOH = 90/10) [UV]; **^{1}H NMR** (400 MHz, DMSO-d$_6$, 298 K): δ = 7.91 (d, $^3J_{H-H}$ = 8.1 Hz, 1H, H^1), 6.18 (dd, $^3J_{H-H}$ = 7.3 Hz, $^3J_{H-H}$ = 6.1 Hz, 1H, H^6), 5.76 (d, $^3J_{H-H}$ = 8.1 Hz, 1H, H^2), 5.25 (d, $^3J_{H-H}$ = 4.5 Hz, 1H, C^{8}OH), 5.02 (*virt.* t, $^3J_{H-H} \approx {}^3J_{H-H}$ = 5.1 Hz, 1H, C^{10}OH), 4.23 (ddd, $^3J_{H-H}$ = 7.5 Hz, $^3J_{H-H}$ = 4.5 Hz, $^3J_{H-H}$ = 3.0 Hz, 1H, H^8), 3.80 (*virt.* q, $^3J_{H-H} \approx {}^3J_{H-H} \approx {}^3J_{H-H}$ = 3.6 Hz, 1H, H^9),

3.65–3.48 (m, 2H, H[10]), 3.15 (s, 3H, H[4]), 2.21–1.99 (m, 2H, H[7]); **^{13}C{^{1}H} NMR** (101 MHz, DMSO-d$_6$, 298 K): δ = 162.1 (C[3]/C[5]), 150.7 (C[3]/C[5]), 138.7 (C[1]), 100.7 (C[2]), 87.5 (C[9]), 85.3 (C[6]), 70.3 (C[8]), 61.2 (C[10]), 27.2 (C[4]).
The signal of C[7] overlaps with the solvent signal of DMSO-d$_6$.

The analytical data are in accordance with the literature.[46]

Indicative HMBC-contacts for determination of the chemoselectivity

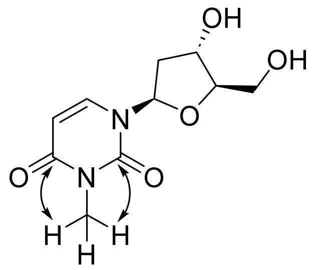

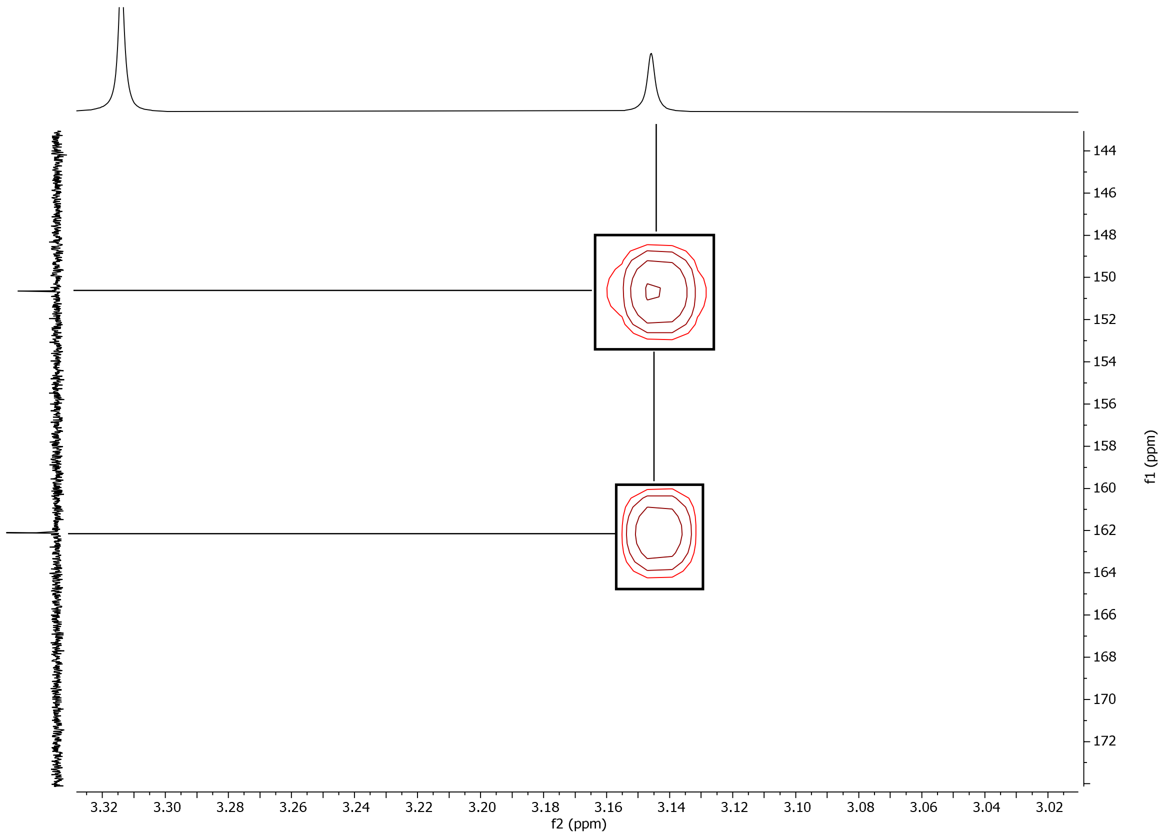

6.9 Methyl *N*-(*tert*-butoxycarbonyl)-*S*-methyl-L-cysteinate (28)

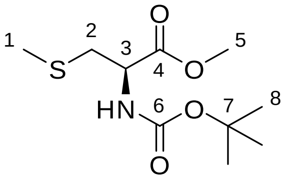

28

The reaction is performed according to general procedure **A** (reaction time 15.5 h; 66% NMR yield) using Boc-Cys-OMe (11.8 mg, 50.0 µmol) as a substrate. In deviation from general procedure **A**, another portion of methyl diphenylphosphinite (5.0 µL, 5.4 mg, 25.0 µmol, 0.50 equiv.) is added to solution **A** after 30 min and the solution is stirred for another 10 min. The crude product is purified by column chromatography (silica, P/EtOAc = 97/3→95/5) to yield methylated product **28**.

Crude NMR with Internal Standard

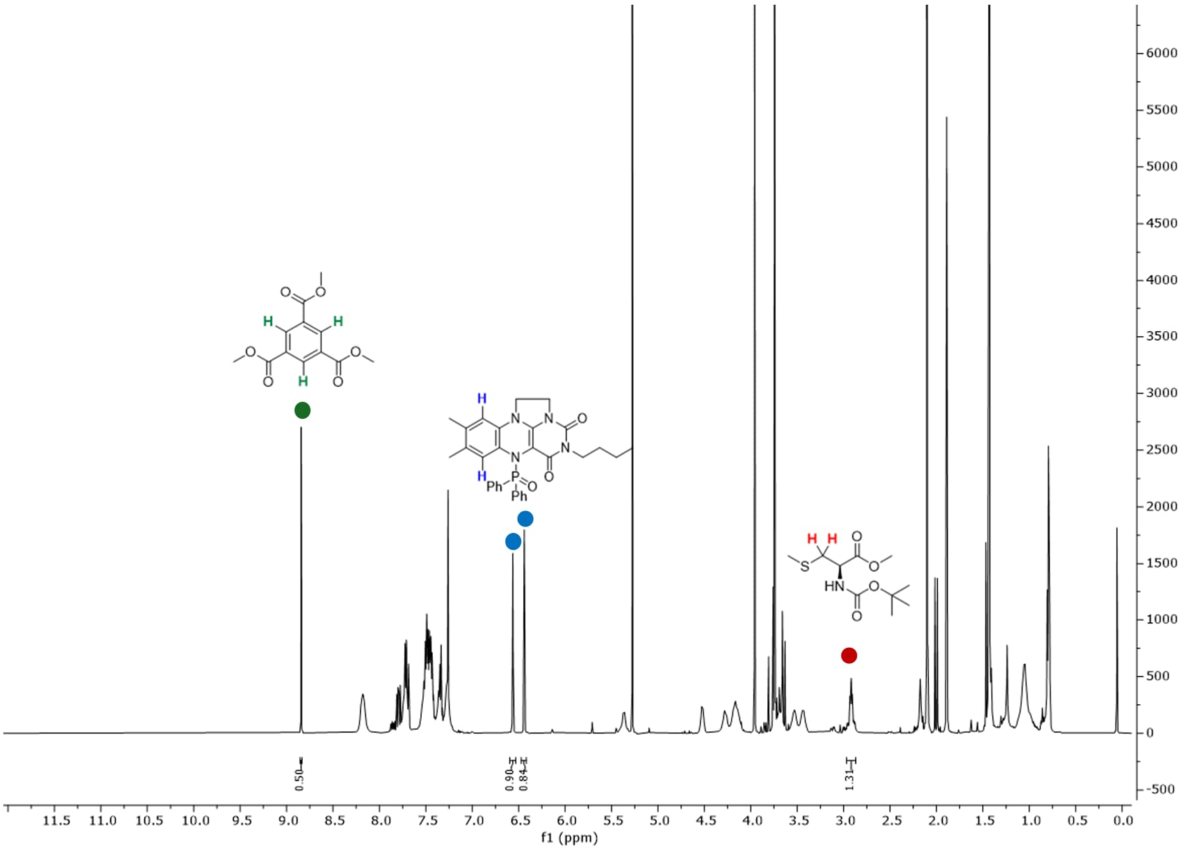

Colorless oil; 7.6 mg (30.3 µmol, 61%, contains 10% trimethyl trimesate, the yield was corrected accordingly); **TLC**: R_f = 0.19 (P/EtOAc = 95/5) [$KMnO_4$]; **^{1}H NMR** (400 MHz, $CDCl_3$, 298 K): δ = 5.33 (br. s, 1H, NH), 4.54 (dd, $^3J_{H\text{-}H}$ = 12.2 Hz, $^3J_{H\text{-}H}$ = 5.2 Hz, 1H, H^3), 3.76 (s, 3H, H^5), 2.98–2.89 (m, 2H, H^2), 2.12 (s, 3H, H^1), 1.45 (s, 9H,

H[8]); **^{13}C{^{1}H} NMR** (101 MHz, $CDCl_3$, 298 K): δ = 171.8 (C^4), 155.3 (C^6), 80.3 (C^7), 53.2 (C^3), 52.7 (C^5), 36.9 (C^2), 28.5 (C^8), 16.4 (C^1).

The analytical data are in accordance with the literature.[47]

Indicative HMBC-contacts for determination of the chemoselectivity

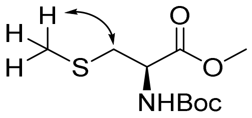

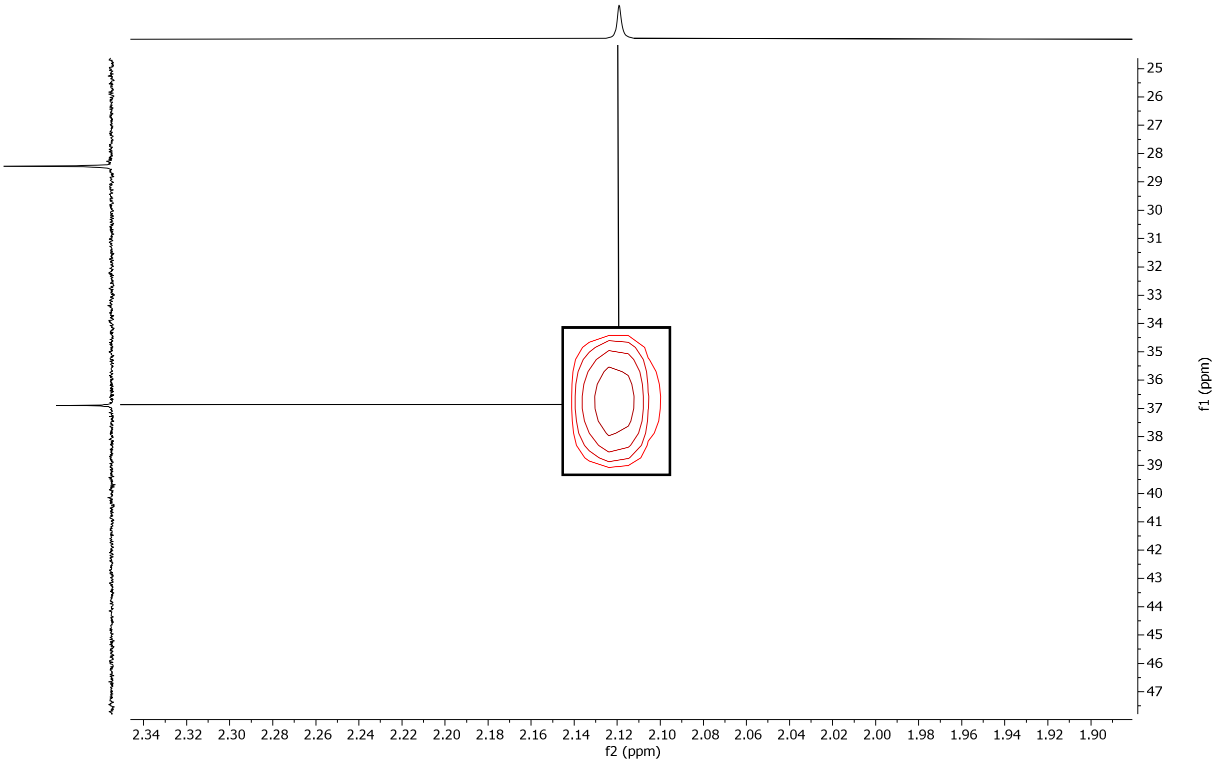

6.10 Enisamium bistriflimide (29)

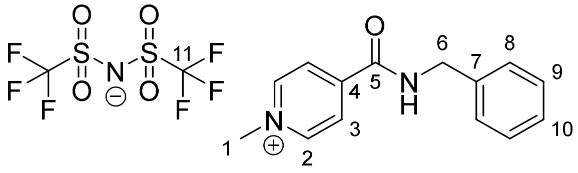

29

The reaction is performed according to general procedure **A** (reaction time 19.8 h; NMR yield was not determined) using benzyl isonicotinamide (10.6 mg, 50.0 µmol) as a substrate and omitting the base. The crude product is purified by column chromatography (silica, CH_2Cl_2/MeOH = 90/10) to yield methylated product **29**.

Yellow solid; 20.1 mg (39.6 µmol, 79%); **TLC**: $R_f = 0.04$ (CH_2Cl_2/MeOH = 90/10) [UV]; **^{1}H NMR** (400 MHz, DMSO–d_6, 298 K): δ = 9.75 (t, $^3J_{H\text{-}H}$ = 5.8 Hz, 1H, NH), 9.19–9.09 (d_{AB}, $^3J_{H\text{-}H}$ = 6.7 Hz, 2H, H^2), 8.51–8.39 (d_{AB}, $^3J_{H\text{-}H}$ = 6.7 Hz, 2H, H^3), 7.40–7.33 (m, 4H, H^8, H^9), 7.32–7.27 (m, 1H, H^{10}), 4.55 (d, $^3J_{H\text{-}H}$ = 5.8 Hz, 2H, H^6), 4.39 (s, 3H, H^1); **^{19}F{^{1}H} NMR** (376 MHz, DMSO-d_6, 298 K): δ = -78.69 (s, 6F); **^{13}C{^{1}H} NMR** (101 MHz, DMSO–d_6, 298 K): δ = 161.9 (C^5), 147.7 (C^4), 146.5 (2C, C^2), 138.3 (C^7), 128.4 (2C, C^8), 127.5 (2C, C^9), 127.2 (C^{10}), 125.4 (2C, C^3), 119.5 (2C, $^1J_{C\text{-}F}$ = 325.7 Hz, C^{11}), 48.0 (C^1), 43.2 (C^6). **HR-MS** (ESI$^+$): m/z = calc. for $[C_{14}H_{15}N_2O]^+$: 227.1179 ($[M]^+$), found: 227.1177; **IR** (ATR): $\tilde{\nu}_{max}$ [cm^{-1}] = 3567 (w, N–H), 3383 (w), 3068 (w, C_{Ar}–H), 2927 (w, C_{Alk}–H), 2855 (w), 1673 (m), 1647 (m, C=O), 1553 (m), 1513 (m), 1457 (m), 1349 (s, SO_2), 1331 (s), 1192 (vs, SO_2, CF_3), 1135 (s, CF_3), 1059 (s, S–N–S), 863 (w), 793 (m), 764 (m), 743 (m, C_{Ar}–H), 702 (m), 655 (m).

Indicative HMBC-contacts for determination of the chemoselectivity

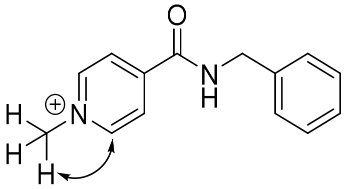

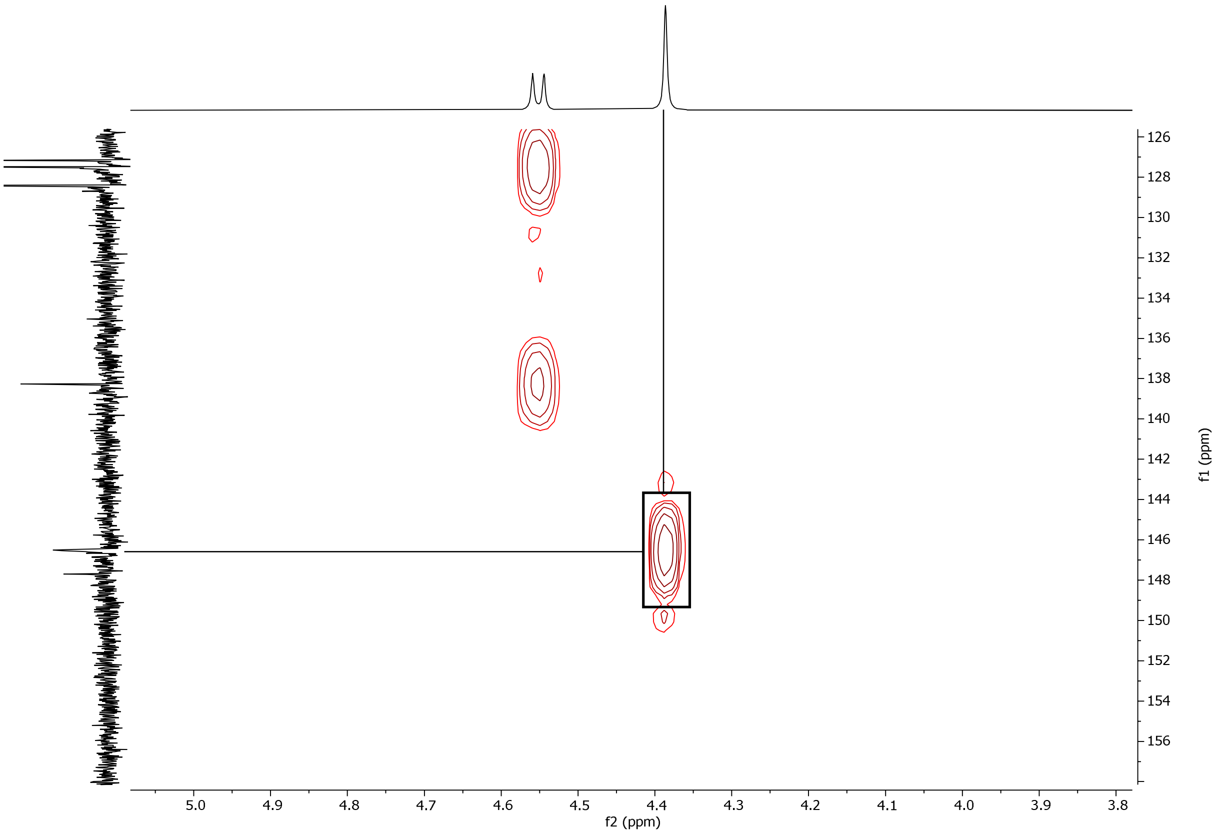

6.11 Caffeine (30)

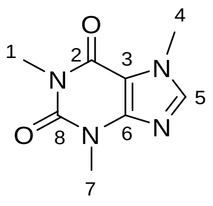

30

The reaction is performed according to general procedure **A** (reaction time 16 h; 50% NMR yield) using theobromine (9.0 mg, 50.0 µmol) as a substrate in a solvent mixture of CH_2Cl_2 (1.50 mL) and DMSO (500 µL). The crude product is purified by column chromatography (silica, EtOAc) to yield methylated product **30**.

Crude NMR with Internal Standard

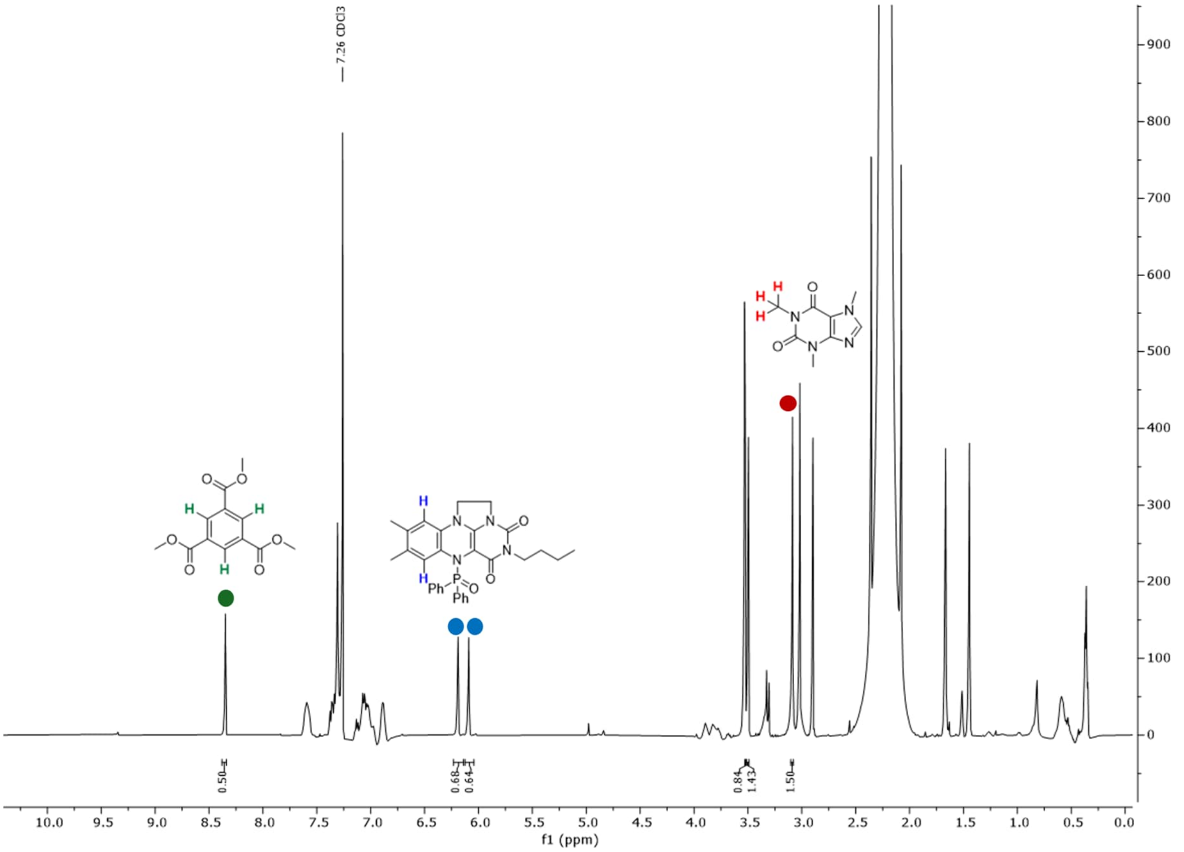

*crude NMR contains 50µL AcOH–d_4 in order to solubilize the remaining starting material.

Yellowish, crystalline solid; 4.1 mg (21.1 µmol, 42%); **TLC**: R_f = 0.15 (EtOAc) [UV]; **^{1}H NMR** (500 MHz, $CDCl_3$, 298 K): δ = 7.54 (s, 1H, H^5), 4.00 (s, 3H, H^4), 3.59 (s, 3H, H^7), 3.41 (s, 3H, H^1); **^{13}C{^{1}H} NMR** (126 MHz, $CDCl_3$, 298 K): δ = 155.5 (C^2), 151.8 (C^8), 148.6 (C^6), 141.4 (C^5), 107.7 (C^3), 33.9 (C^4), 30.0 (C^7), 28.1 (C^1).

The analytical data are in accordance with the literature.[48]

Indicative HMBC-contacts for determination of the chemoselectivity

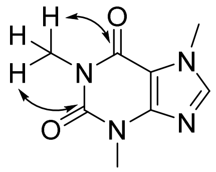

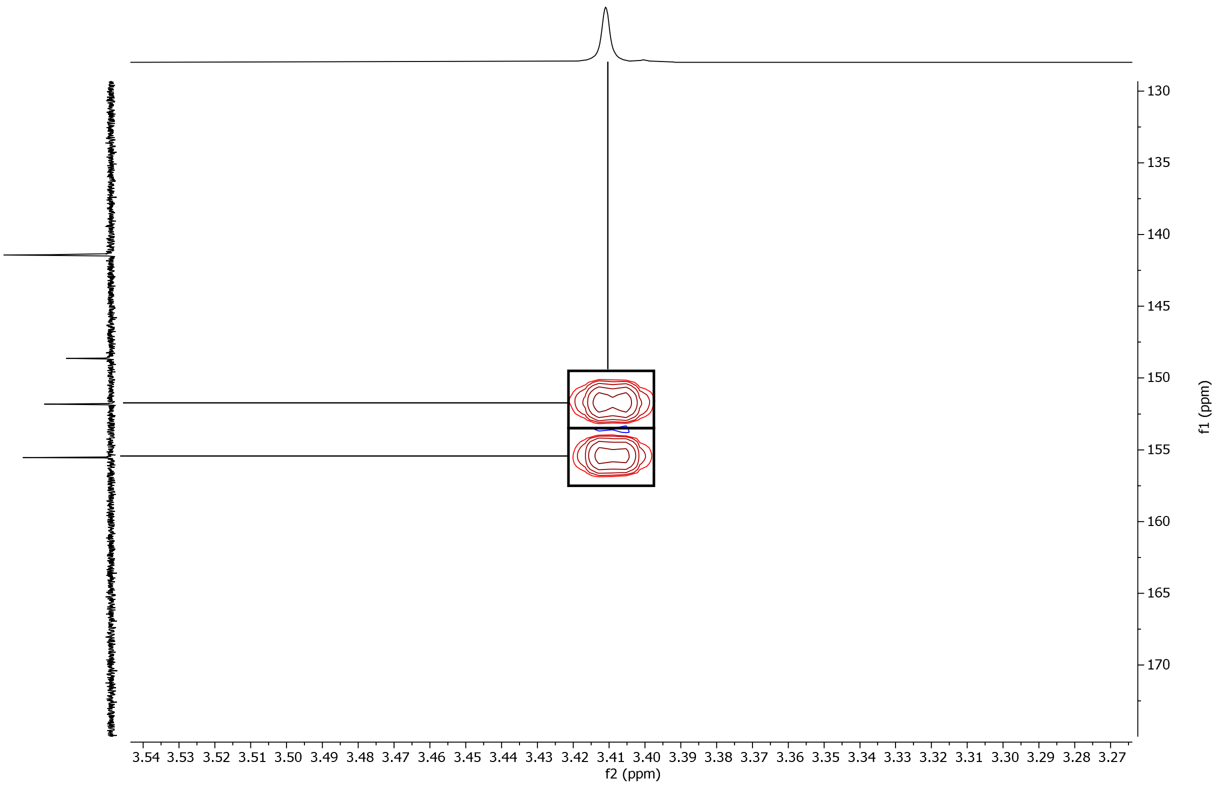

6.12 11-Cyclopropyl-4,5-dimethyl-5,11-dihydro-6*H*-dipyrido[3,2-*b*:2',3'-*e*][1,4] diazepin-6-one (31)

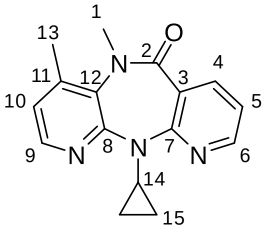

31

The reaction is performed according to general procedure **A** (reaction time 18.5 h; 45% NMR yield; side product **SI-9** was observed in 5% NMR yield; S = 9/1) using nevirapine (13.3 mg, 50.0 µmol) as a substrate. The crude product is purified by column chromatography (silica, P/EtOAc = 60/40) and preparative normale phase preparative HPLC (Kromasil 100-5-SIL, 19 mL/min, *n*-heptane/^{i}PrOH = 85/15→60/40 for 30 min) to yield methylated product **31**.

Crude NMR with Internal Standard

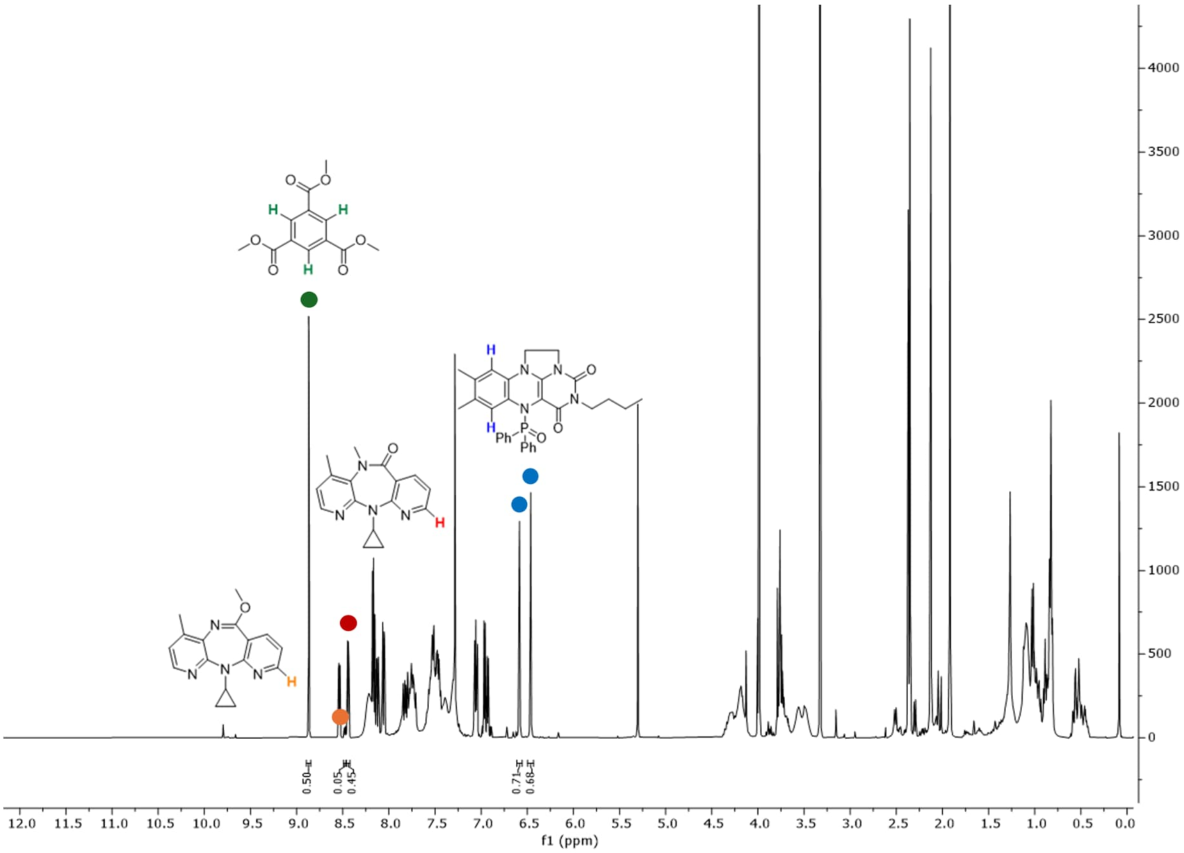

Colorless solid; 4.9 mg (17.5 µmol, 35%); **TLC**: R_f = 0.16 (P/EtOAc = 60/40) [UV]; ^{1}**H NMR** (400 MHz, $CDCl_3$, 298 K): δ = 8.44 (dd, $^{3}J_{H\text{-}H}$ = 4.8 Hz, $^{3}J_{H\text{-}H}$ = 2.0 Hz, 1H, H^{6}), 8.17 (d, $^{3}J_{H\text{-}H}$ = 4.8 Hz, 1H, H^{9}), 8.05 (dd, $^{3}J_{H\text{-}H}$ = 7.6 Hz, $^{4}J_{H\text{-}H}$ = 2.0 Hz, 1H, H^{4}), 7.05 (dd, $^{3}J_{H\text{-}H}$ = 7.6 Hz, $^{3}J_{H\text{-}H}$ = 4.8 Hz, 1H, H^{5}), 6.95 (dd, $^{3}J_{H\text{-}H}$ = 4.8 Hz, $^{4}J_{H\text{-}H}$ = 0.7 Hz, 1H, H^{10}), 3.73

(tt, $^3J_{H\text{-}H}$ = 6.7 Hz, $^3J_{H\text{-}H}$ = 3.8 Hz, 1H, H^{14}), 3.32 (s, 3H, H^1), 2.35 (s, 3H, H^{13}), 1.06–0.95 (m, 2H, H^{15a}), 0.61–0.45 (m, 2H, H^{15b}); **^{13}C{^{1}H} NMR** (101 MHz, $CDCl_3$, 298 K): δ = 168.7 (C^2), 161.2 (C^7), 158.4 (C^8), 150.9 (C^6), 145.1 (C^9), 143.6 (C^{11}), 140.2 (C^4), 129.6 (C^{12}), 122.9 (C^{10}), 121.8 (C^3), 119.3 (C^5), 38.4 (C^1), 28.8 (C^{13}), 19.2 (C^{14}), 8.7 (C^{15a}), 8.7 (C^{15b}). **HR-MS** (ESI^+): *m/z* = calc. for $[C_{16}H_{17}N_4O]^+$: 281.1397 ($[M+H]^+$), found: 281.1395; **IR** (ATR): $\tilde{\nu}_{max}$ [cm^{-1}] = 3081 (w, C_{Ar}–H), 3031 (w), 3010 (w), 2921 (w, C_{Alk}–H), 2858 (w, C_{Alk}–H), 1645 (s, C=O/C=N), 1584 (m, C=C), 1569 (m), 1551 (m), 1471 (m), 1458 (m), 1436 (m), 1407 (vs), 1373 (m), 1353 (m), 1308 (m), 1299 (m), 1290 (m), 1263 (m), 1237 (m), 1206 (m), 1188 (m), 1130 (m),1104 (m), 1078 (m), 1045 (m), 1026 (m), 997 (m), 985 (m), 959 (m), 952 (m), 940 (m), 909 (m), 891 (m), 874 (m), 843 (m), 835 (m), 814 (m), 799 (m), 787 (m), 773 (s, C_{Ar}–H), 732 (m), 678 (m).

Indicative HMBC-contacts for determination of the chemoselectivity

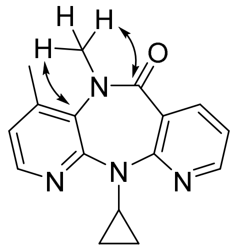

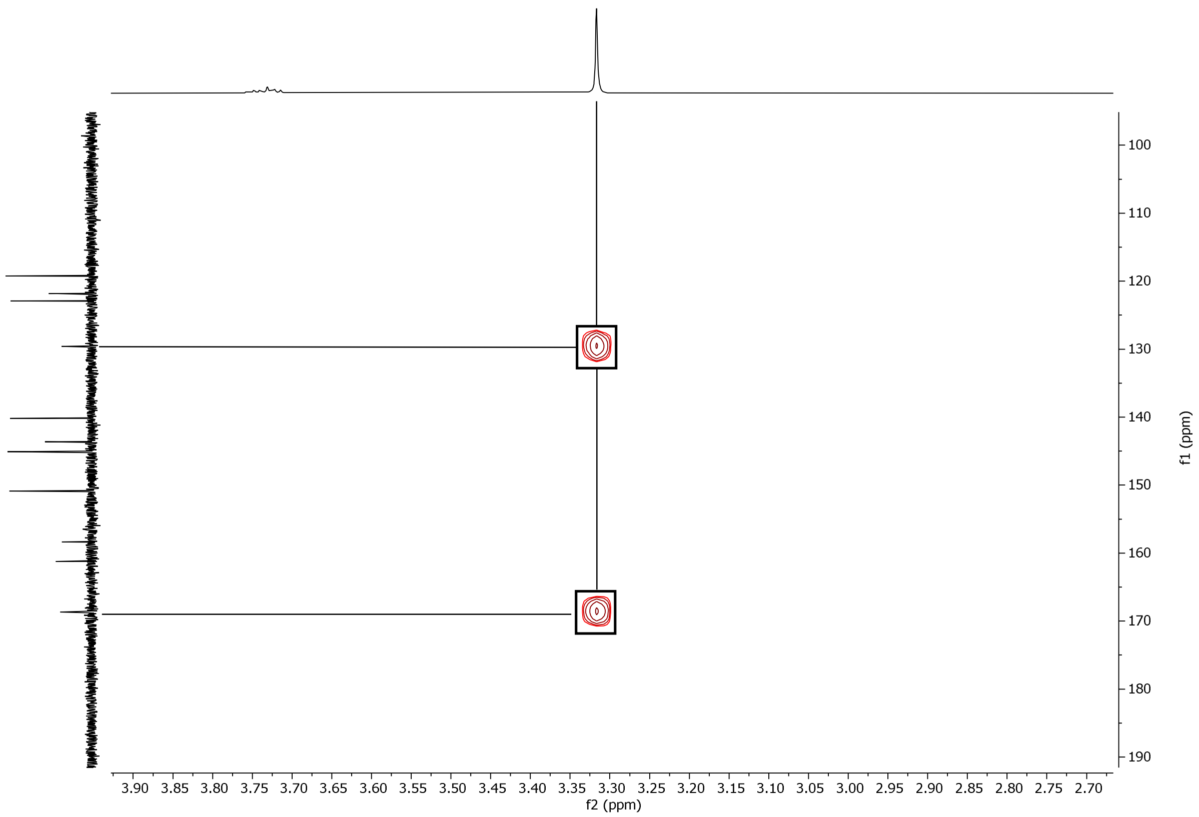

In a separate experiment, traces of the side product **SI-10** were isolated which we assigned to *O*-methylation based on the chemical shifts of the methyl group and indicative NOE-contacts (*vide infra*).

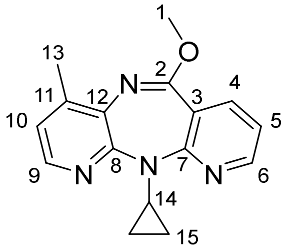

SI-10

^{1}H NMR (400 MHz, $CDCl_3$, 298 K): δ = 8.47 (dd, $^3J_{H\text{-}H}$ = 4.9 Hz, $^3J_{H\text{-}H}$ = 2.0 Hz, 1H, H^6), 8.07 (d, $^3J_{H\text{-}H}$ = 4.9 Hz, 1H, H^9), 7.76 (dd, $^3J_{H\text{-}H}$ = 7.6 Hz, $^4J_{H\text{-}H}$ = 1.9 Hz, 1H, H^4), 6.97 (dd, $^3J_{H\text{-}H}$ = 7.6 Hz, $^3J_{H\text{-}H}$ = 4.9 Hz, 1H, H^5), 6.89 (dd, $^3J_{H\text{-}H}$ = 4.9 Hz, $^4J_{H\text{-}H}$ = 0.7 Hz, 1H, H^{10}), 4.00 (s, 3H, H^1), 3.66 (tt, $^3J_{H\text{-}H}$ = 6.9 Hz, $^3J_{H\text{-}H}$ = 3.8 Hz, 1H, H^{14}), 2.34 (s, 3H, H^{13}), 0.84 (br. s, 2H, H^{15a}), 0.52–0.42 (m, 2H, H^{15b}).

Indicative NOE-contacts for determination of the chemoselectivity

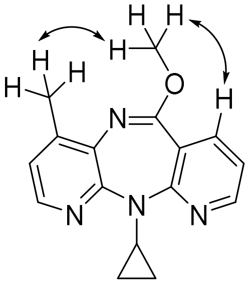

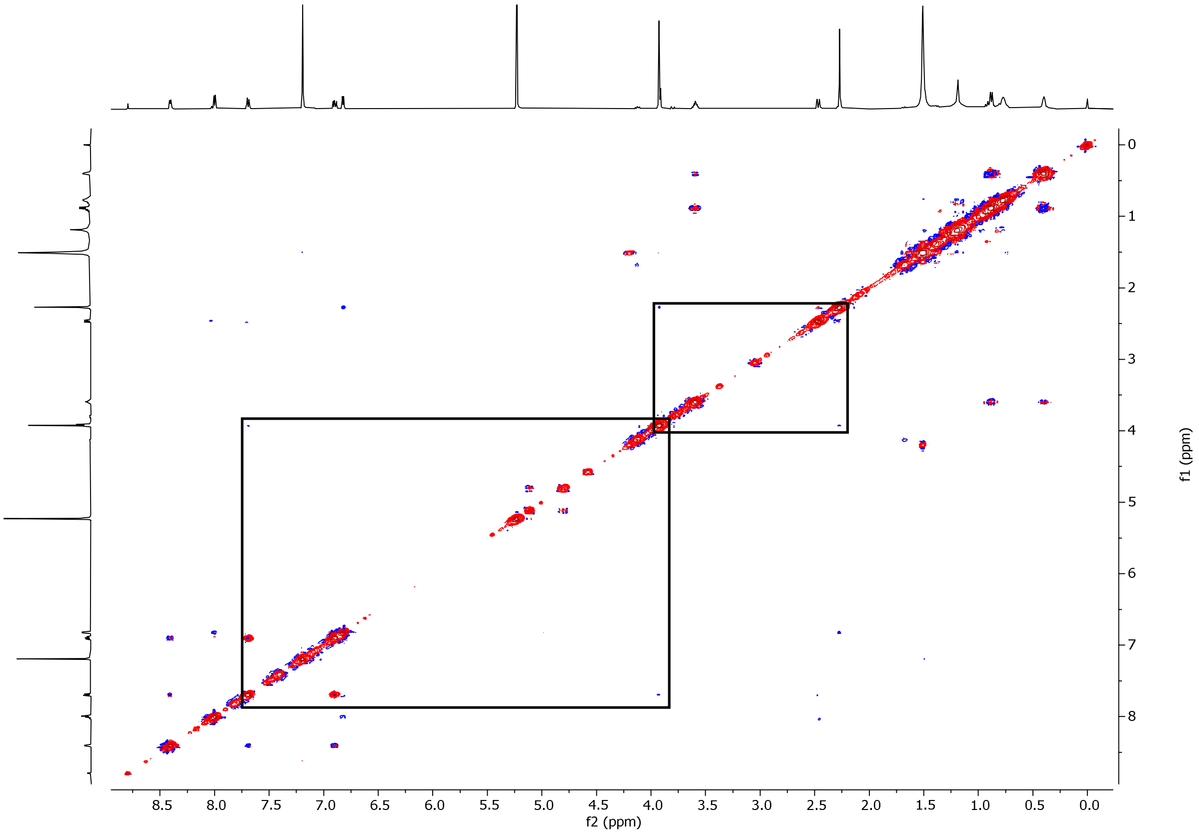

6.13 5-(5-(4-((4'-chloro-5,5-dimethyl-3,4,5,6-tetrahydro-[1,1'-biphenyl]-2-yl)methyl) piperazin-1-yl)-2-(((3-nitro-4-(((tetrahydro-2H-pyran-4-yl)methyl)amino)phenyl) sulfonyl)carbamoyl)phenoxy)-7-methyl-1H-pyrrolo[2,3-b]pyridin-7-ium bis((trifluoromethyl)sulfonyl)amide (32)

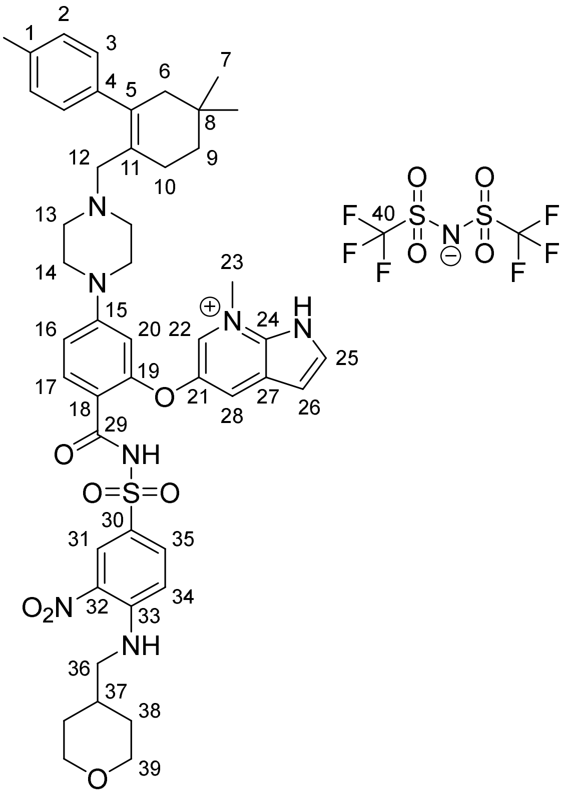

32

The reaction is performed according to general procedure **A** (reaction time 19 h) using venetoclax (43.4 mg, 50.0 µmol) as a substrate. The crude product is purified by column chromatography (silica, CH_2Cl_2/MeOH = 96/4) to yield methylated product **32**.

The crude NMR did not allow for assigning the position of methylation. Our structural analysis is based on 2D-NMR of the purified product (*vide infra*).

Yellow solid; 10.2 mg (8.50 µmol, 17%); **TLC**: R_f = 0.10 (CH_2Cl_2/MeOH = 96/4) [UV]; **^{1}H NMR** (500 MHz, DMSO-d_6, 298 K): δ = 8.49 (d, $^4J_{H\text{-}H}$ = 2.3 Hz, 1H, H^{22}), 8.35 (t, $^3J_{H\text{-}H}$ = 5.5 Hz, 1H, C^{36}NH), 8.24 (s, 1H, H^{31}), 7.97 (s, 1H, H^{28}), 7.82 (d, $^3J_{H\text{-}H}$ = 3.4 Hz, 1H, H^{25}), 7.68–7.63 (m, 2H, H^{17}, H^{35}), 7.41–7.35 (m, 2H, H^2), 7.11–7.05 (m, 2H, H^3), 6.91 (d, $^3J_{H\text{-}H}$ = 9.2 Hz, 1H, H^{34}), 6.76 (dd, $^3J_{H\text{-}H}$ = 8.9 Hz, $^3J_{H\text{-}H}$ = 2.5 Hz, 1H, H^{16}), 6.70 (d, $^3J_{H\text{-}H}$ = 3.4 Hz, 1H, H^{26}), 6.54 (br. s, 1H, H^{20}), 4.32 (s, 3H, H^{23}), 3.85 (ddd, $^2J_{H\text{-}H}$ = 11.5 Hz, $^3J_{H\text{-}H}$ = 4.4 Hz, $^3J_{H\text{-}H}$ = 1.5 Hz, 2H, H^{39a}), 3.31–3.23 (m, 6H, H^{36}, H^{39b}), 3.15 (br. s, 4H, H^{14}), 2.78 (s, 2H, H^{12}), 2.25 (br. s, 4H, H^{13}), 2.19 (br. s, 2H, H^{10}), 1.98 (br. s, 2H, H^6), 1.88 (ddd, $^3J_{H\text{-}H}$ = 11.4 Hz, $^3J_{H\text{-}H}$ = 7.5 Hz, $^3J_{H\text{-}H}$ = 3.9 Hz, 1H, H^{37}), 1.65–1.57 (m, 2H, H^{38a}), 1.41 (t,

$^{3}J_{H\text{-}H}$ = 6.4 Hz, 2H, H^{9}), 1.26 (virt. qd, $^{3}J_{H\text{-}H} \approx {}^{4}J_{H\text{-}H}$ = 11.4 Hz, $^{3}J_{H\text{-}H}$ = 4.4 Hz, 2H, H^{38b}), 0.95 (s, 6H, H^{7}); **^{13}C{^{1}H} NMR** (126 MHz, DMSO-d_6, 298 K): δ = 168.4 (C^{29}), 154.3 (C^{21}), 153.1 (C^{19}), 145.8 (C^{33}), 145.8 (C^{15}), 142.1 (C^{4}), 136.8 (C^{24}), 134.7 (C^{35}), 134.2 (C^{5}), 132.8 (C^{17}), 130.9 (C^{1}), 130.1 (2C, C^{3}), 129.1 ($C^{11}/C^{30}/C^{32}$), 129.1 ($C^{11}/C^{30}/C^{32}$), 129.1 ($C^{11}/C^{30}/C^{32}$), 128.7 (C^{22}), 128.2 (2C, C^{2}), 125.8 (C^{27}), 125.4 (C^{31}), 124.3 (C^{28}), 113.5 (C^{34}), 110.6 (C^{16}), 107.2 (C^{18}), 103.2 (C^{26}), 66.7 (2C, C^{39}), 59.9 (C^{12}), 52.4 (2C, C^{13}), 47.9 (C^{36}), 47.2 (2C, C^{14}), 46.3 (C^{6}), 42.3 (C^{23}), 34.9 (C^{9}), 33.9 (C^{37}), 30.3 (2C, C^{38}), 29.0 (C^{8}), 28.0 (2C, C^{7}), 25.3 (C^{10}); The signals of C^{20},C^{25},C^{40} either overlap with other signals or display low intensity; **^{19}F{^{1}H} NMR** (376 MHz, DMSO-d_6, 298 K): δ = -78.70 (s, 6F); **HR-MS** (ESI^{+}): *m/z* = calc. for $[C_{46}H_{54}ClN_{7}O_{7}S]^{2+}$: 441.6742 ($[M+H]^{2+}$), found: 441.6741, calc. for $[C_{46}H_{53}ClN_{7}O_{7}S]^{+}$: 882.3410 ($[M]^{+}$), found: 882.3400; **IR** (ATR): $\tilde{\nu}_{max}$ [cm^{-1}] = 3370 (w, N–H), 3099 (w, C_{Ar}–H), 3025 (w), 2911 (w, C_{Alk}–H), 2840 (w, C_{Alk}–H), 1681 (m), 1611 (s, C=O), 1571 (m), 1514 (m, NO_2), 1494 (m), 1469 (m), 1453 (m), 1427 (m), 1387 (m), 1355 (m), 1330 (s), 1269 (vs, C–O), 1235 (vs, C–O), 1191 (m), 1173 (m), 1128 (vs, C–F), 1088 (vs, C–F), 1053 (m), 1014 (m), 992 (m), 964 (m), 902 (m), 843 (m), 826 (m), 810 (m, C_{Ar}–H), 779 (m, C_{Ar}–H), 765 (m, C_{Ar}–H), 725 (m), 704 (m), 678 (m), 662 (m).

Indicative HMBC-contacts for determination of the chemoselectivity

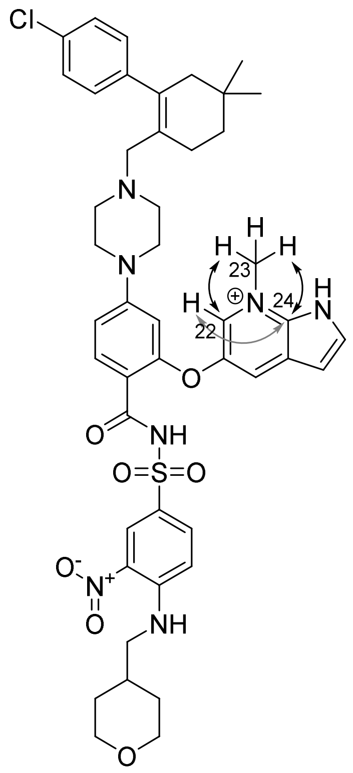

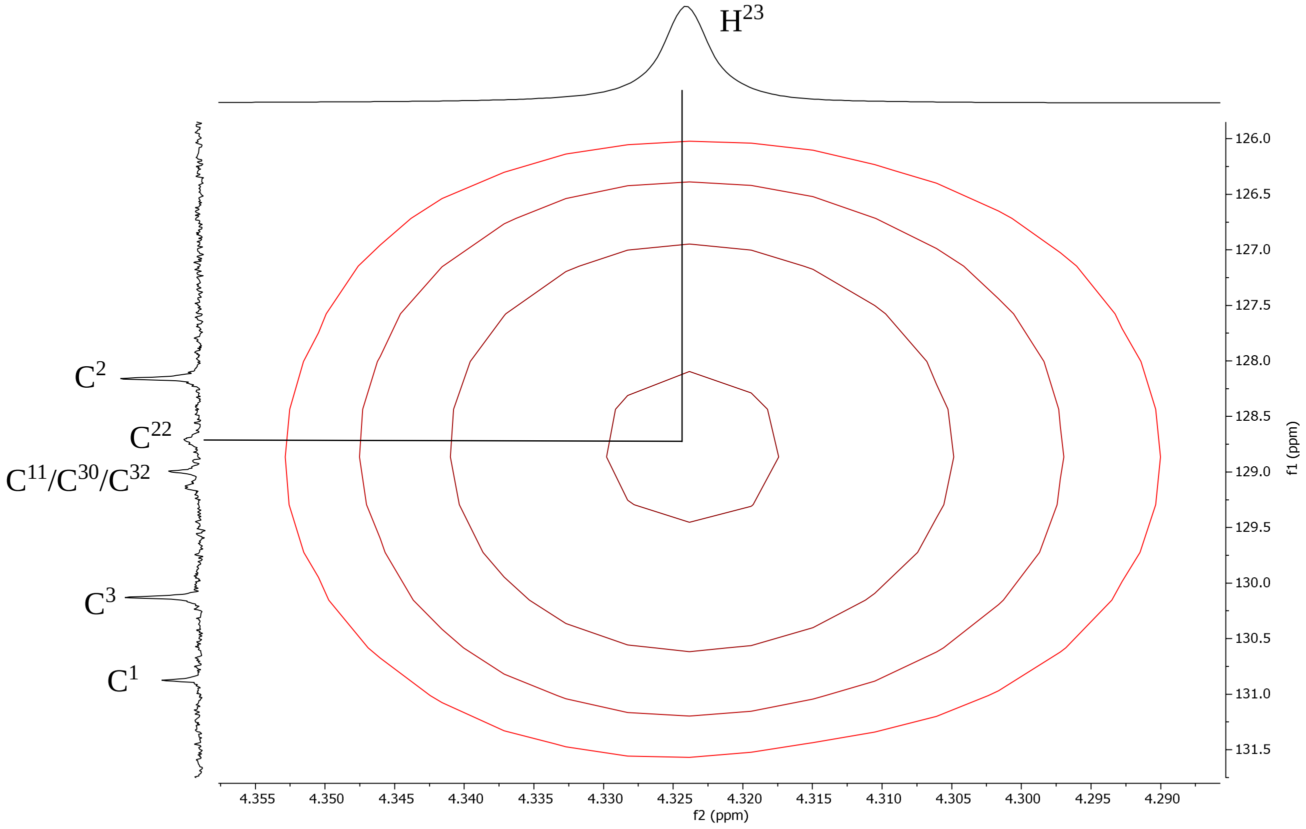

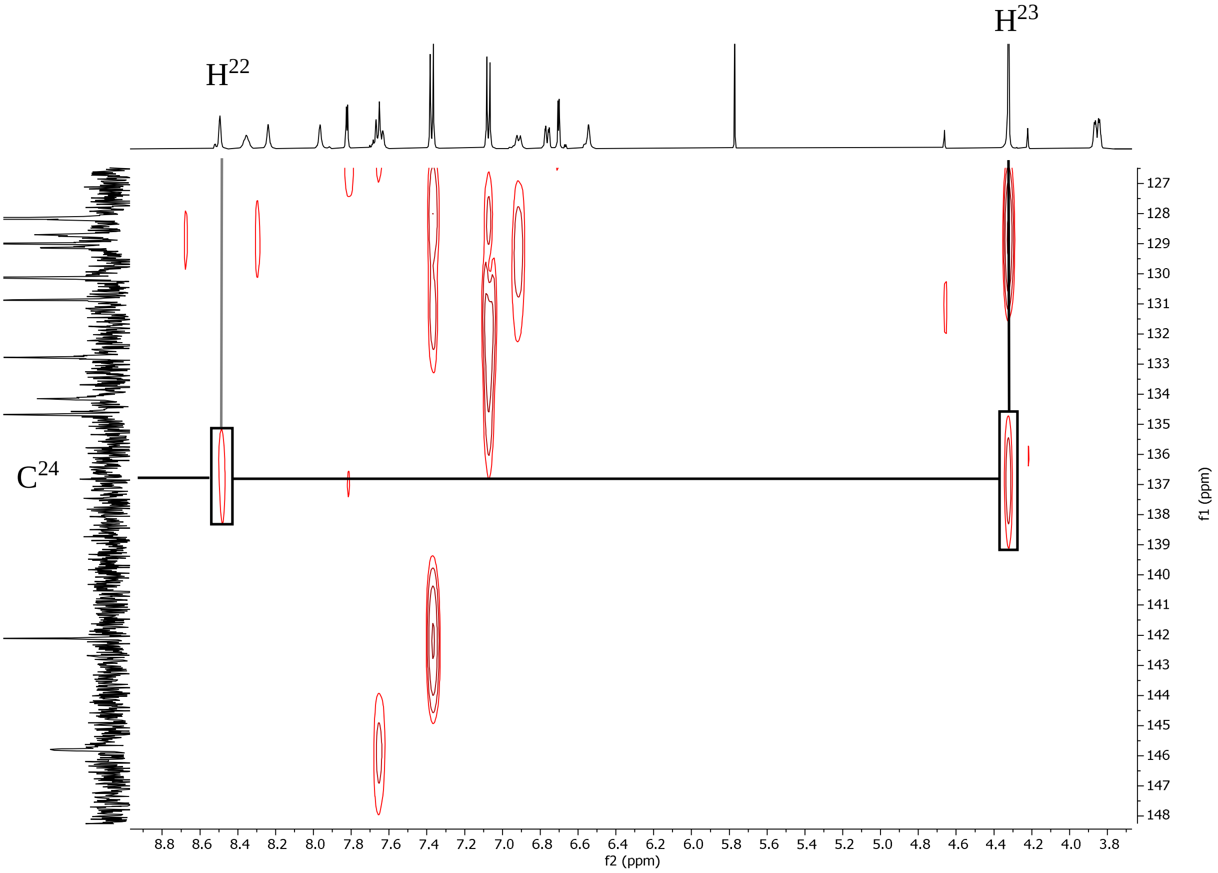

H22
H23
C24
f1 (ppm)
f2 (ppm)

6.14 1-((2*R*,4*S*,5*R*)-4-Hydroxy-5-(hydroxymethyl)tetrahydrofuran-2-yl)-5-methyl-3-(methyl-*d*3)pyrimidine-2,4(1*H*,3*H*)-dione (35)

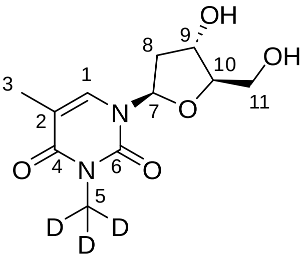

35

The reaction is performed according to general procedure **A** (reaction time 19.7 h; 27% NMR yield) using thymidine (**34**) (11.5 mg, 50.0 µmol) as a substrate in a solvent mixture of CH_2Cl_2 (1.50 mL) and DMSO (500 µL) and methyl diphenylphosphinite-*d*3 (91% purity, 11.2 µL, 12.1 mg, 50.0 µmol, 1.00 equiv.) as a reagent. The crude product is purified by column chromatography (silica, CH_2Cl_2/MeOH = 95/5) to yield methylated product **35**.

Crude NMR with Internal Standard

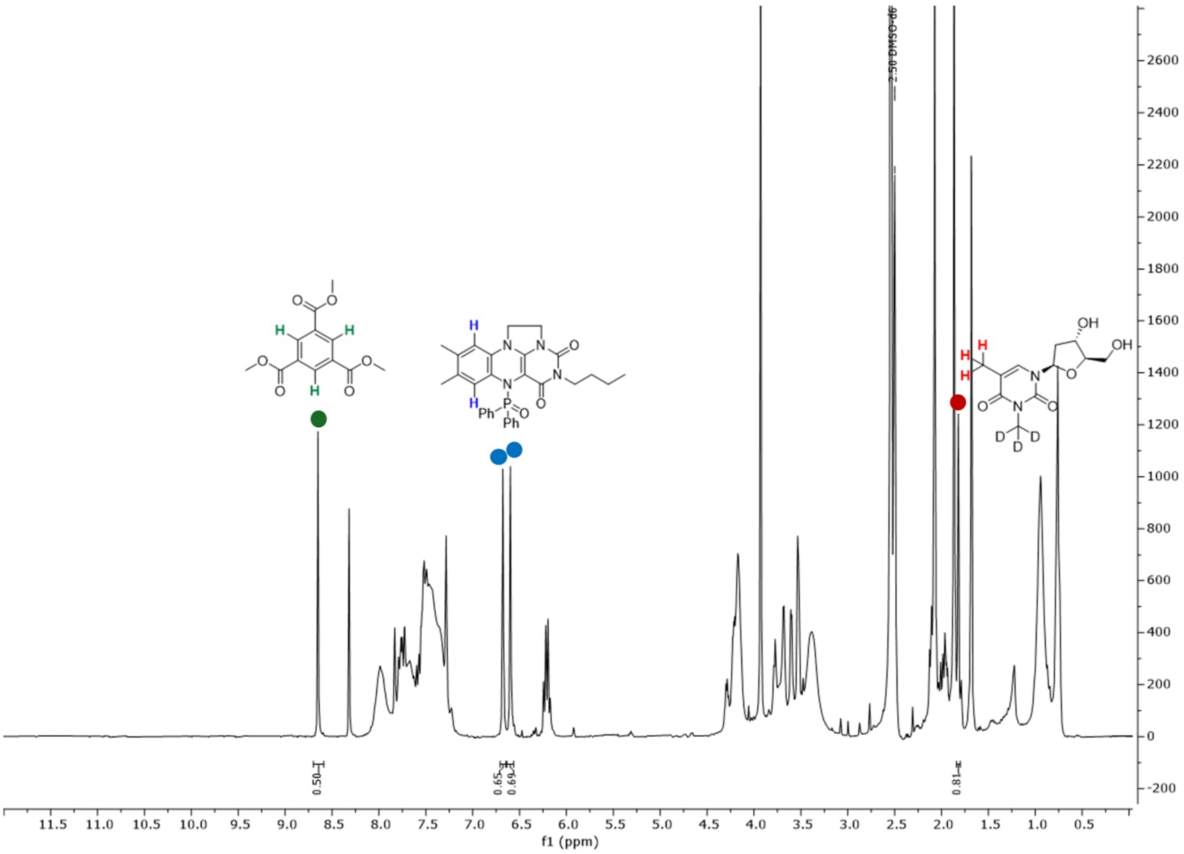

Colorless solid; 2.5 mg (9.64 µmol, 19%); **TLC**: R_f = 0.10 (CH_2Cl_2/MeOH = 95/5) [UV]; **^{1}H NMR** (500 MHz, DMSO-*d*6, 298 K): δ = 7.78 (d, $^4J_{H\text{-}H}$ = 1.2 Hz, 1H, H^1), 6.21 (*virt.* t, $^3J_{H\text{-}H} \approx {}^3J_{H\text{-}H}$ = 6.8 Hz, 1H), 5.27 (d, $^3J_{H\text{-}H}$ = 4.2 Hz, 1H, C^{9}OH), 5.07 (*virt.* t,

$^3J_{H\text{-}H} \approx {}^3J_{H\text{-}H}$ = 5.2 Hz, 1H, C^{11}OH), 4.23 (*virt.* dt, $^3J_{H\text{-}H}$ = 7.8 Hz, $^3J_{H\text{-}H} \approx {}^3J_{H\text{-}H}$ = 4.3 Hz, 1H, H^9), 3.77 (*virt.* q, $^3J_{H\text{-}H} \approx {}^3J_{H\text{-}H} \approx {}^3J_{H\text{-}H}$ = 3.6 Hz, 1H, H^{10}), 3.60 (ddd, $^2J_{H\text{-}H}$ = 11.8 Hz, $^3J_{H\text{-}H}$ = 5.4 Hz, $^3J_{H\text{-}H}$ = 3.7 Hz, 1H, H^{11a}), 3.55 (ddd, $^2J_{H\text{-}H}$ = 11.8 Hz, $^3J_{H\text{-}H}$ = 5.1 Hz, $^3J_{H\text{-}H}$ = 3.9 Hz, 1H, H^{11b}), 2.13–2.06 (m, 2H, H^8), 1.82 (d, $^4J_{H\text{-}H}$ = 1.2 Hz, 3H, H^3); **^{13}C{^{1}H} NMR** (126 MHz, DMSO-d_6, 298 K): δ = 163.0 (C^4), 150.6 (C^6), 134.6 (C^1), 108.3 (C^2), 87.4 (C^{10}), 84.9 (C^7), 70.3 (C^9), 61.3 (C^{11}), 26.9 (hept, $^2J_{C\text{-}D}$ = 21.5 Hz, C^5), 13.1 (C^3); The signal of C^8 overlaps with the solvent signal of DMSO-d_6; **HR-MS** (ESI$^+$): *m/z* = calc. for $[C_{11}H_{13}D_3N_2NaO_5]^+$: 282.1140 ([M+Na]$^+$), found: 282.1139; **IR** (ATR): $\tilde{\nu}_{max}$ [cm^{-1}] = 3407 (m, O–H), 2926 (w, O–H), 1693 (s), 1667 (vs, C=O), 1632 (vs, C=O), 1472 (m), 1291 (m), 1194 (w), 1099 (m), 1060 (m, C–O), 763 (m), 676 (m, C–H).

7. Comparative Reactions with Methyl triflate

7.1 General Procedure B: Stoichiometric Methylation Reactions using Methyl Triflate

Methyl triflate (8.20 mg, 50.0 µmol, 1.00 equiv.) is placed in a crimp-cap vial equipped with a magnetic stirring bar, the vial is sealed with a septum safety cap and the atmosphere is exchanged with Ar (3×). Dichloromethane (2.50 mL, 20 mM) is added and the solution is vigorously stirred for 3 min (solution **A**). In a second crimp-cap vial, the substrate (50.0 µmol, 1.00 equiv.) and cesium carbonate (24.4 mg, 75.0 µmol, 1.50 equiv.) are placed in a crimp-cap vial equipped with a magnetic stirring bar, the vial is sealed with a septum safety cap, the atmosphere is exchanged with Ar (3×) and dichloromethane (2.00 mL, 25 mM) is added (solution **B**). Solution **A** is transferred to solution **B**, the vial of solution **A** is rinsed with CH_2Cl_2 (2×250 µL), and the reaction is stirred at r.t. The reaction is quenched by adding 500 µL triethylamine and the vial is opened, the solution is diluted with dichloromethane, transferred to a round bottom flask and all volatiles are removed *in vacuo*. The crude product is transferred to an NMR tube and a stock solution of the internal standard trimethyl 1,3,5-benzenetricarboxylate (8.33 µmol in 350 µL $CDCl_3$, relaxation time of the ^{1}H-spectrum is set to $\tau = 10$ s, the integral of the signal at $\delta = 8.82$ ppm is set to 0.50) is added. Yields are determined by comparing the obtained integrals.

7.2 *N*-Methyl quinolone (36) via flavin methodology

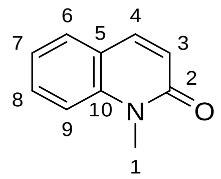

36

The reaction is performed according to general procedure **A** (reaction time 16 h; 56% NMR yield; side product **37** was observed in 8% NMR yield; $S = 6.9/1$) using 2-quinolone (7.3 mg, 50.0 µmol) as a substrate. The crude product is purified by column chromatography (silica, P/EtOAc = 70/30→60/40) to yield methylated product **36**.

Comparative crude NMR spectra with Internal Standard

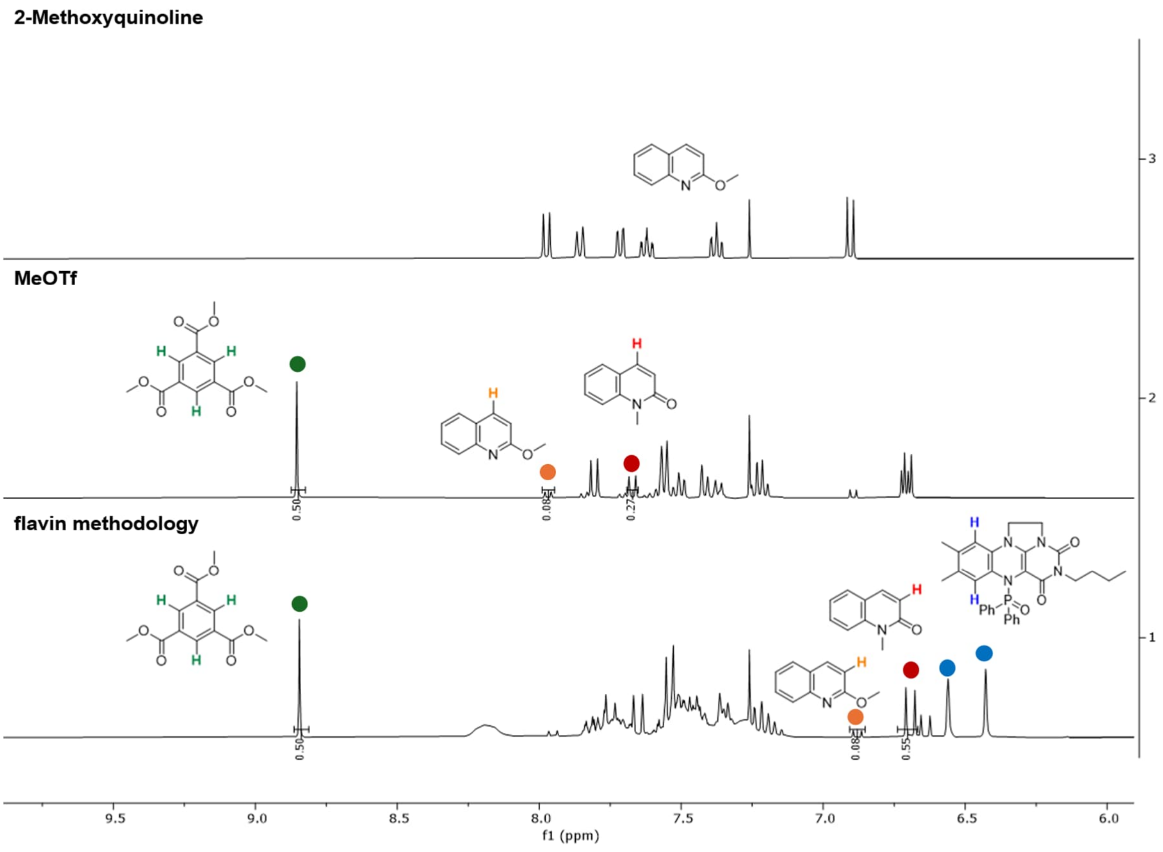

Yellowish solid; 4.0 mg (25.1 µmol, 50%); **TLC**: $R_f = 0.45$ (P/EtOAc = 50/50) [UV]; **^{1}H NMR** (400 MHz, $CDCl_3$, 298 K): $\delta = 7.67$ (d, $^3J_{H\text{-}H} = 9.4$ Hz, 1H, H^4), 7.59–7.55 (m, 2H, H^6, H^8), 7.37 (dd, $^3J_{H\text{-}H} = 8.3$ Hz, $^4J_{H\text{-}H} = 1.0$ Hz, 1H, H^9), 7.24 (ddd, $^3J_{H\text{-}H} = 8.0$ Hz, $^3J_{H\text{-}H} = 7.2$ Hz, $^4J_{H\text{-}H} = 1.0$ Hz, 1H, H^7), 6.71 (d, $^3J_{H\text{-}H} = 9.4$ Hz, 1H, H^3), 3.73 (s, 3H, H^1). **^{13}C{^{1}H} NMR** (101 MHz, $CDCl_3$, 298 K): $\delta = 162.5$ (C^2), 140.2 (C^{10}), 139.1 (C^4), 130.8 (C^8), 128.9 (C^6), 122.2 (C^7), 121.9 (C^3), 120.9 (C^5), 114.3 (C^9), 29.6 (C^1).

The analytical data are in accordance with the literature.[49]

7.3 *N*-Methyl quinolone (36) via methyl triflate

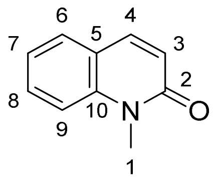

36

The reaction is performed according to general procedure **B** (reaction time 16 h) using quinoline-2-ol (7.3 mg, 50.0 µmol) yielding product **36** (27% NMR yield, side product **37** was observed in 8% NMR yield; *S* = 3.4/1).

7.4 *tert*-Butyl ((*R*)-1-(((*S*)-3-(4-hydroxyphenyl)-1-(methylamino)-1-oxopropan-2-yl)-amino)-3-(methylthio)-1-oxopropan-2-yl)carbamate (38) via flavin methodology

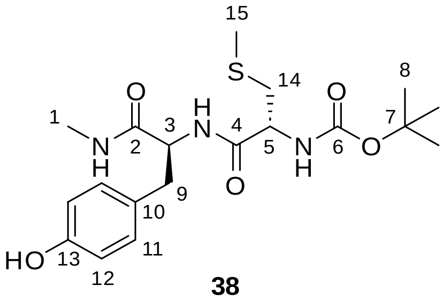

38

The reaction is performed according to general procedure **A** (reaction time 17.5 h) using peptide **SI-8** (19.9 mg, 50.0 µmol) and 2.00 equiv. of Cs_2CO_3 (32.6 mg, 100 µmol). The crude material is purified by column chromatography (silica, CH_2Cl_2/MeOH = 98/2 and C_{18}-silica, H_2O/MeCN = 98/2→0/100) to yield a mixture of desired product **38**, starting material **SI-8**, and dehydroalanine side product **SI-11** (ratio determined by NMR spectroscopy 62/19/19). Additionally, adduct **11** was isolated. Due to the complexity of the crude NMR, UPLC-MS was considered and indicative for the formation of the side product **39** only in the case of the MeOTf methodology. No overalkylation was observed in the case of the flavin methodology.

Comparative crude UPLC-MS traces of MeOTf and flavin methodology

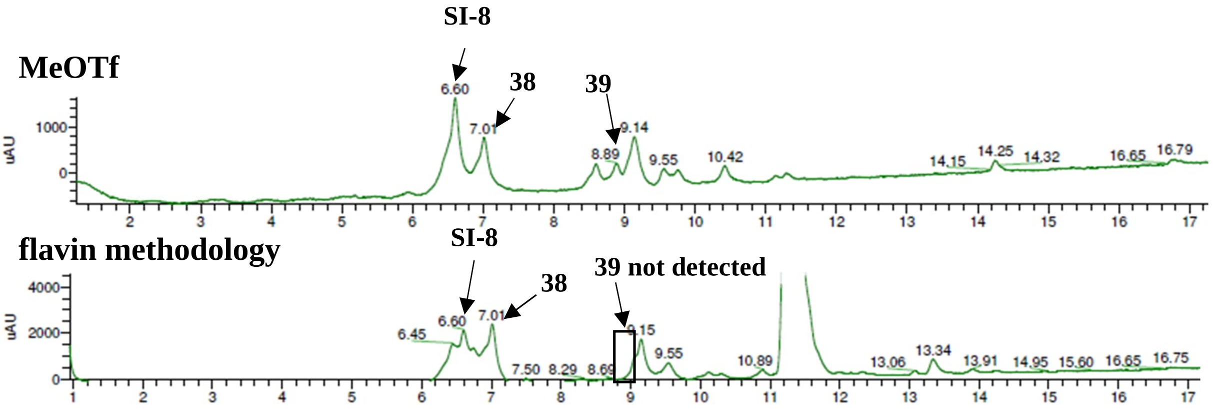

Colorless solid; 4.0 mg (9.79 µmol, 20%); **TLC**: R_f = 0.19 (CH_2Cl_2/MeOH = 95/5) [UV]; **^{1}H NMR** (400 MHz, acetone–d_6, 298 K): δ = 7.38–7.33 (m, 1H, C^3NH), 7.15–7.13 (m, 1H, C^1NH), 7.03 (d$_{AB}$, $^3J_{H\text{-}H}$ = 8.4 Hz, 2H, H^{11}), 6.72 (d, $^3J_{H\text{-}H}$ = 8.4 Hz, 2H, H^{12}), 6.29–6.27 (m, 1H, C^5NH), 4.53–4.48 (m, 1H, H^3), 4.23–4.17 (m, 1H, H^5), 3.02–2.97 (m, 1H, H^{9a}), 2.88–2.82 (m, 2H, H^{9b}, H^{14a}), 2.74–2.71 (m, 1H, H^{14b}), 2.66 (d, $^3J_{H\text{-}H}$ = 4.7 Hz, 3H, H^1), 2.07 (s, 3H, H^{15}), 1.40 (s, 9H, H^8); **^{13}C{^{1}H} NMR** (126 MHz, acetone–d_6, 298 K): δ = 171.6 (C^2), 171.1 (C^4), 156.9 (C^6), 156.7 (C^{13}), 131.2 (2C, C^{11}), 128.8 (C^{10}), 115.8 (2C, C^{12}), 79.8 (C^7), 55.2 (C^3), 54.9

(C[5]), 37.7 (C[9]), 36.7 (C[14]), 28.5 (3C, C[8]), 25.9 (C[1]), 15.5 (C[15]); **HR-MS** (ESI$^+$): *m/z* = calc. for $[C_{19}H_{29}N_3NaO_5S]^+$: 434.1720 ($[M+Na]^+$), found: 434.1717.

Indicative HMBC-contacts for determination of the chemoselectivity

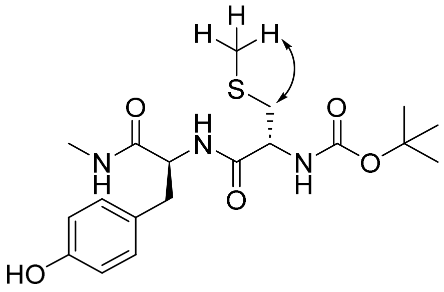

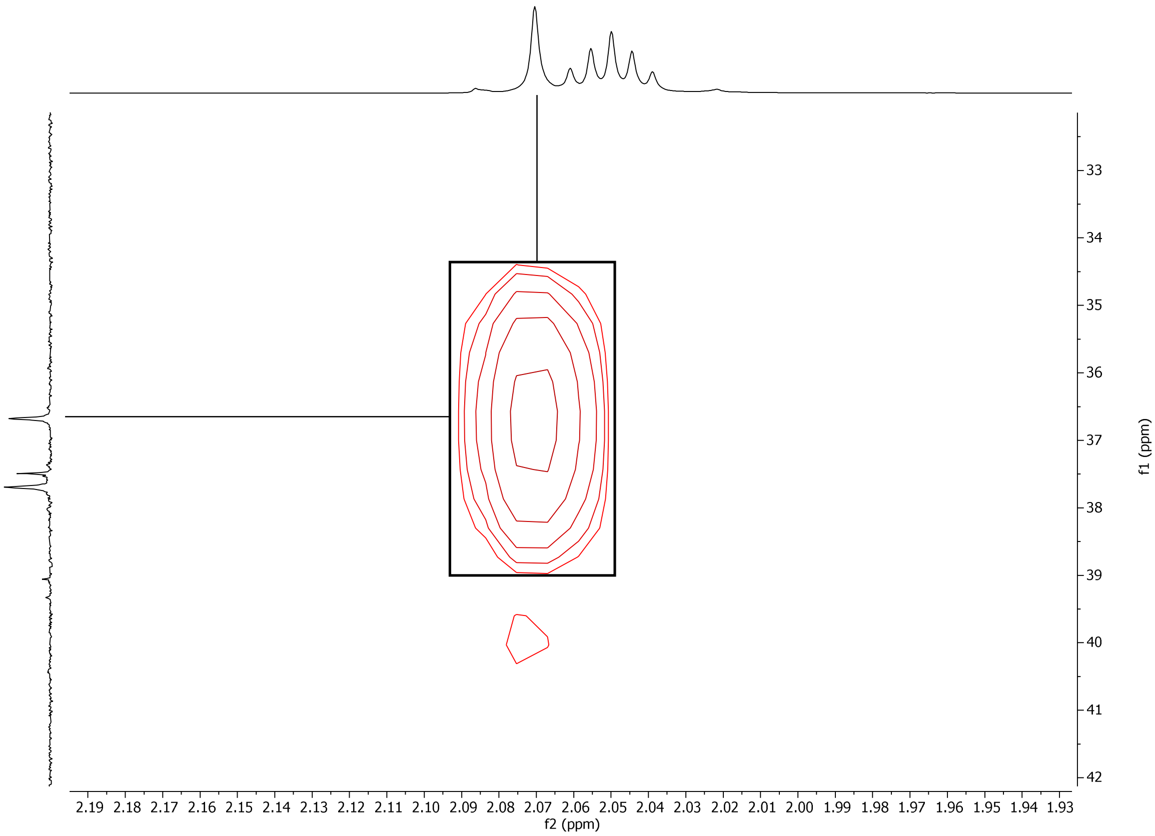

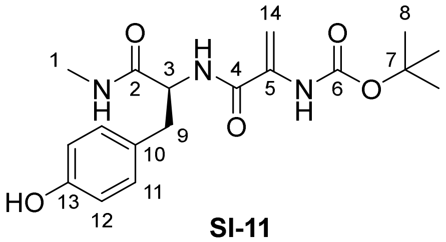

SI-11

Colorless solid; 1.2 mg (3.40 µmol, 7%); **TLC**: $R_f = 0.19$ (CH_2Cl_2/MeOH = 95/5) [UV]; **^{1}H NMR** (400 MHz, acetone–d_6, 298 K): δ = 7.28–7.25 (m, 1H, C^{3}NH), 7.15–7.13 (m, 1H, C^{1}NH), 7.07 (d, $^3J_{H\text{-}H}$ = 8.4 Hz, 2H, H^{11}), 5.80 (d, $^2J_{H\text{-}H}$ = 1.1 Hz, 1H, H^{14a}), 5.29 (d, $^2J_{H\text{-}H}$ = 1.1 Hz, 1H, H^{14b}), 4.60–4.54 (m, 1H, H^3), 3.10 (dd, $^2J_{H\text{-}H}$ = 13.9 Hz, $^3J_{H\text{-}H}$ = 5.5 Hz, 1H, H^{9a}), 2.88–2.82 (m, 1H, H^{9b}), 2.69 (d, $^3J_{H\text{-}H}$ = 4.7 Hz, 3H, H^1), 1.44 (s, 9H, H^8); **^{13}C{^{1}H} NMR** (126 MHz, acetone–d_6, 298 K): δ = 171.7 (C^2), 164.3 (C^4), 156.9 (C^{13}), 153.3 (C^6), 136.0 (C^5), 131.0 (2C, C^{11}), 129.2 (C^{10}), 115.9 (2C, C^{12}), 99.3 (C^{14}), 80.6 (C^7), 56.2 (C^3), 37.5 (C^9), 28.3 (3C, C^8), 26.0 (C^1); **HR-MS** (ESI$^+$): m/z = calc. for $[C_{18}H_{25}N_3NaO_5]^+$: 386.1686 ([M+Na]$^+$), found: 386.1682.

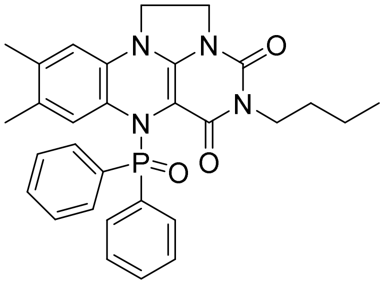

11

Pale orange solid; 19.7 mg (37.4 mmol, 75%). For a full characterization, see above.

7.5 *tert*-Butyl ((*R*)-1-(((*S*)-3-(4-hydroxyphenyl)-1-(methylamino)-1-oxopropan-2-yl)-amino)-3-(methylthio)-1-oxopropan-2-yl)carbamate (38) via methyl triflate

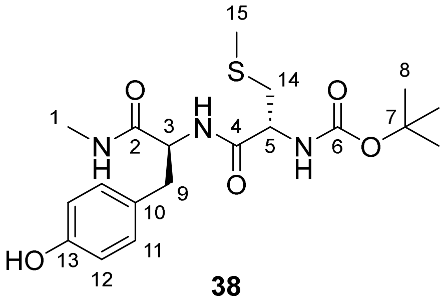

38

The reaction is performed according to general procedure **B** (reaction time 16.5 h) using peptide **SI-8** (19.9 mg, 50.0 µmol) and 2.00 equiv. of Cs_2CO_3 (32.6 mg, 100 µmol). The crude material

is purified by column chromatography (silica, CH_2Cl_2/MeOH = 98/2) to yield a mixture of product **38** and starting material **SI-8** (ratio determined by NMR spectroscopy: 55/45). Additionally, overalkylation product **39** was isolated.

Colorless solid; 3.3 mg (7.64 µmol, 15%); **TLC**: R_f = 0.19 (CH_2Cl_2/MeOH = 95/5) [UV]; **^{1}H NMR** (500 MHz, acetone–d_6, 298 K): δ = 8.25 (s, 1H, OH), 7.40 (d, $^3J_{H\text{-}H}$ = 8.2 Hz, 1H, C^{3}NH), 7.15–7.13 (m, 1H, C^{1}NH), 7.03 (d, $^3J_{H\text{-}H}$ = 8.4 Hz, 2H, H^{11}), 6.72 (d, $^3J_{H\text{-}H}$ = 8.4 Hz, 2H, H^{12}), 6.33 (d, $^3J_{H\text{-}H}$ = 7.0 Hz, 1H, C^{5}NH), 4.55–4.49 (m, 1H, H^3), 4.22–4.17 (m, 1H, H^5), 3.02–2.97 (m, 1H, H^{9a}), 2.88–2.82 (m, 2H, H^{9b}, H^{14a}), 2.73–2.69 (m, 1H, H^{14b}), 2.67–2.65 (m, 3H, H^1), 2.07 (s, 3H, H^{15}), 1.40 (s, 9H, H^8); **^{13}C{^{1}H} NMR** (126 MHz, acetone–d_6, 298 K): δ = 171.7 (C^2), 171.2 (C^4), 157.0 (C^6), 156.7 (C^{13}), 131.2 (2C, C^{11}), 128.8 (C^{10}), 115.9 (2C, C^{12}), 79.8 (C^7), 55.3 (C^3), 55.0 (C^5), 37.7 (C^9), 36.7 (C^{14}), 28.5 (3C, C^8), 26.1 (C^1), 15.5 (C^{15}); **HR-MS** (ESI$^+$): *m/z* = calc. for $[C_{19}H_{29}N_3NaO_5S]^+$: 434.1720 ($[M+Na]^+$), found: 434.1716; **IR** (ATR): $\tilde{\nu}_{max}$ [cm^{-1}] = 3329 (w, O–H), 2981 (w, C–H$_{Ar}$), 1681 (w, C=O), 1646 (s, C=O), 1597 (s, C–C$_{Ar}$), 1516 (s, C–C$_{Ar}$), 1457 (s, C–C$_{Ar}$), 1415 (w), 1367 (w), 1318 (w, C-N), 1284 (w, C–N), 1162 (w), 1111 (w), 1046 (w), 856 (w), 832 (w), 802 (w), 779 (w), 735 (w), 707 (w).

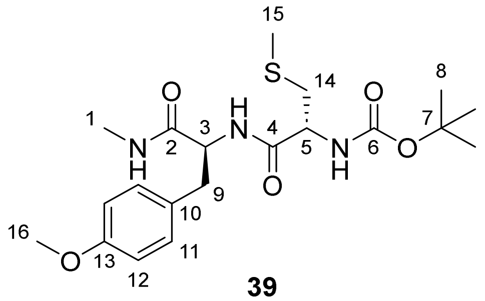

39

Colorless solid; 1.6 mg (3.64 µmol, 7%); **TLC**: R_f = 0.33 (CH_2Cl_2/MeOH = 95/5) [UV]; **^{1}H NMR** (500 MHz, acetone-d_6, 298 K): δ = 7.40 (d, $^3J_{H\text{-}H}$ = 8.2 Hz, 1H, C^{3}NH), 7.17–7.11 (m, 3H, C^{1}NH, H^{11}), 6.81 (d, $^3J_{H\text{-}H}$ = 8.5 Hz, 2H, H^{12}), 6.26 (d, $^3J_{H\text{-}H}$ = 7.3 Hz, 1H, C^{5}NH), 4.54–4.50 (m, 1H, H^3), 4.21–4.17 (m, 1H, H^5), 3.15 (s, 3H, H^{16}), 3.05–3.02 (m, 1H, H^{9a}), 2.93–2.88 (m, 2H, H^{9b}, H^{14a}), 2.72–2.68 (m, 1H, H^{14b}), 2.66 (d, $^3J_{H\text{-}H}$ = 4.7 Hz, 3H, H^1), 2.07 (s, 3H, H^{15}), 1.40 (s, 9H, H^8); **^{13}C{^{1}H} NMR** (126 MHz, acetone-*d6*, 298 K): δ = 171.6 (C^2), 171.2 (C^4), 159.4 (C^6), 156.6 (C^{13}), 131.1 (2C, C^{11}), 130.2 (C^{10}), 114.4 (2C, C^{12}), 79.8 (C^7), 55.4 (C^3), 55.2 (C^{16}), 55.0 (C^5), 37.7 (C^9), 36.7 (C^{14}), 28.5 (3C, C^8), 26.0 (C^1), 15.5 (C^{15}); **HR-MS** (ESI$^+$): *m/z* = calc. for $[C_{20}H_{31}N_3NaO_5S]^+$: 448.1877 ($[M+Na]^+$), found: 448.1874; **IR** (ATR): $\tilde{\nu}_{max}$ [cm^{-1}] = 3307 (w, NH), 2923 (w, C–H$_{Ar}$), 2324 (w), 2108 (w), 1691 (w, C=O), 1646 (s, C=O), 1515 (s, C–C$_{Ar}$), 1412 (w), 1391 (w), 1367 (w), 1250 (w, C–N), 1169 (w), 1036 (w), 824 (w), 779 (w), 693 (w).

8. Cleavage of Flavin Adduct 11

8.1 Methyl diphenylphosphinate (40)

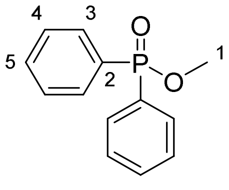

40

Adduct **11** (26.3 mg, 50.0 µmol, 1.00 equiv.) is dissolved in a methanolic hydrogen chloride solution (3 M, 1.67 mL, 100 equiv.) and the solution is stirred at r.t. for 14 h. The HCl is removed by an Ar stream and all volatiles are removed *in vacuo*. The crude product is purified by automated reversed phase flash column chromatography (C_{18}-silica, H_2O/MeCN = 98/2→0/100).

Yellowish oil; 9.0 mg (38.8 µmol, 78%); ^{1}**H NMR** (300 MHz, $CDCl_3$, 298 K): δ = 7.88–7.74 (m, 4H, H^3), 7.58–7.40 (m, 6H, H^4, H^5), 3.77 (d, $^{3}J_{H\text{-}P}$ = 11.2 Hz, 3H, H^1); ^{13}**C{^{1}H} NMR** (101 MHz, $CDCl_3$, 298 K): δ = 132.4 (d, $^{4}J_{C\text{-}P}$ = 2.9 Hz, 2C, C^5), 131.8 (d, $^{3}J_{C\text{-}P}$ = 10.2 Hz, 4C, C^3), 131.2 (d, $^{1}J_{C\text{-}P}$ = 137.2 Hz, 2C, C^2), 128.72 (d, $^{2}J_{C\text{-}P}$ = 13.1 Hz, 4C, C^4), 51.7 (d, $^{3}J_{C\text{-}P}$ = 6.2 Hz, C^1); ^{31}**P{^{1}H} NMR** (163 MHz, $CDCl_3$, 298 K): δ = 33.18.

The analytical data are in accordance with the literature.[50]

8.2 5-Butyl-9,10-dimethyl-4,6-dioxo-1,2,5,6-tetrahydro-4*H*-benzo[*g*]imidazo[1,2,3-*ij*] pteridin-3-ium bistriflimide (12)

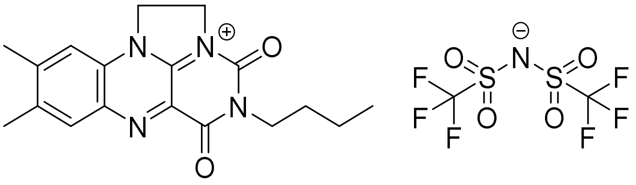

12

Adduct **11** (26.3 mg, 50.0 µmol, 1.00 equiv.) is dissolved in a mixture of acetonitrile and water (1/1, 2.00 mL, 25 mM), two tips of a spatula of bistriflimidic acid are added and the solution is stirred at r.t. for 24.5 h. The solution is diluted with brine (5 mL) and CH_2Cl_2 (15 mL), the phases are separated, and the organic phase is extracted with brine (3×5 mL). All volatiles are removed in vacuo and the crude product is purified by automated reversed phase flash column chromatography (C_{18}-silica, H_2O/MeCN = 98/2→0/100).

Yellow solid; 20.0 mg (33.0 µmol, 67%).

The analytical data (^{1}H and ^{19}F{^{1}H} NMR) of this reaction (*vide infra*)) are in accordance with the data previously acquired for flavin **12**.

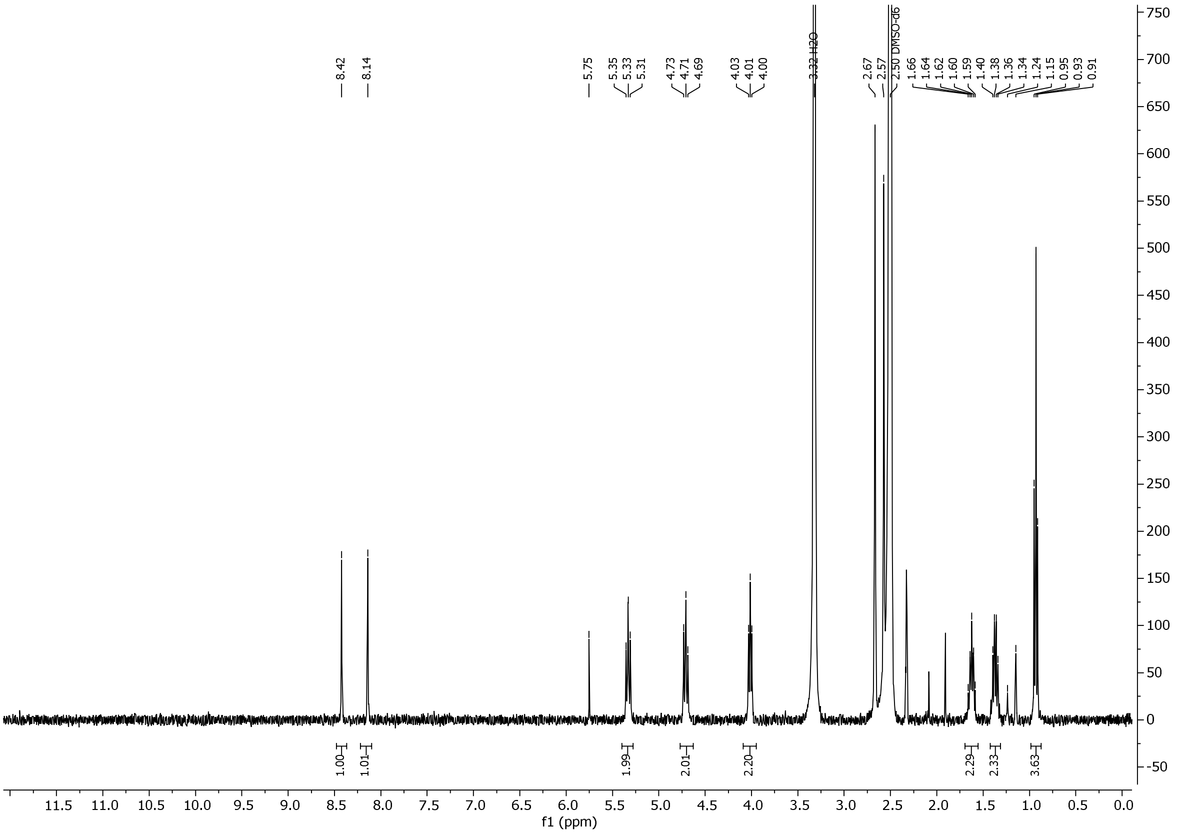

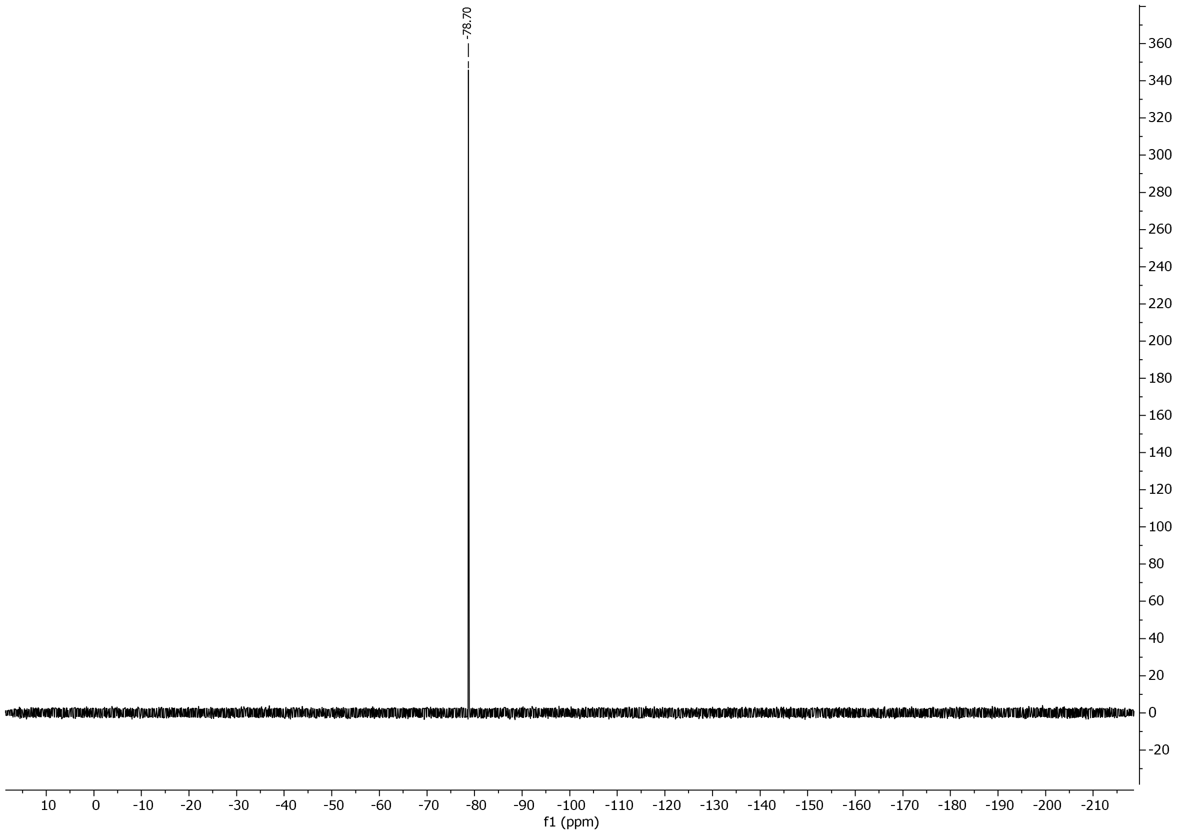
-78.70
f1 (ppm)

9. Crystallographic Data

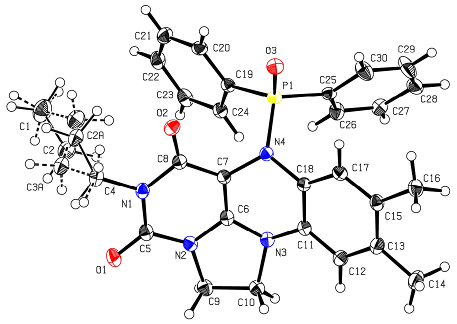

Figure S 10: ORTEP representation of the solid-state structure of compound **11** (C = black, N = blue, O = red and P = yellow) shown with 50 % probability displacement ellipsoids.

A orange, needle-shaped crystal of $C_{30}H_{31}N_4O_3P$ coated with perfluorinated ether and fixed on top of a Kapton micro sampler was used for X-ray crystallographic analysis. The X-ray intensity data were collected at 100(2) K on a Bruker D8 VENTURE three-angle diffractometer with a TXS rotating anode with MoK_α radiation (λ=0.71073 Å) using APEX4.[51] The diffractometer was equipped with a Helios optic monochromator, a Bruker PHOTON III detector, and an Oxford Cryostreamlow temperature device.

A matrix scan was used to determine the initial lattice parameters. All data were integrated with the Bruker SAINT V8.40B software package using a narrow-frame algorithm and the reflections were corrected for Lorentz and polarisation effects, scan speed, and background.[52] The integration of the data using a monoclinic unit cell yielded a total of 136157 reflections within a 2θ range [°] of 4.02 to 52.87 (0.80 Å), of which 5345 were independent. Data were corrected for absorption effects including odd and even ordered spherical harmonics by the multi-scan method (SADABS 2016/2).[53] Space group assignment was based upon systematic absences, E statistics, and successful refinement of the structure.

The structure was solved by direct methods using SHELXT and refined by full-matrix least-squares methods against F^2 by minimizing $\Sigma w(F_o^2 - F_c^2)^2$ using SHELXL in conjunction with SHELXLE.[54-56] All non-hydrogen atoms were refined with anisotropic displacement parameters. Hydrogen atoms were refined isotropically on calculated positions using a riding model with their U_{iso} values constrained to 1.5 times the U_{eq} of their pivot atoms for terminal sp^3 carbon atoms and a C–H distance of 0.98 Å. Non-methyl hydrogen atoms were refined using a riding model with methylene, aromatic, and other C–H distances of 0.99 Å, 0.95 Å, and 1.00 Å, respectively, and U_{iso} values constrained to 1.2 times the U_{eq} of their pivot atoms.

Neutral atom scattering factors for all atoms and anomalous dispersion corrections for the non-hydrogen atoms were taken from International Tables for Crystallography.[57] Supplementary crystallographic data reported in this paper have been deposited with the Cambridge Crystallographic Data Centre (CCDC 2496823) and can be obtained free of charge from The Cambridge Crystallographic Data Centre via www.ccdc.cam.ac.uk/structures.[58] This report and the CIF file were generated using FinalCif.[59]

Table S 1 Crystal data and structure refinement for compound 11.

CCDC number	2496823
Empirical formula	$C_{30}H_{31}N_4O_3P$
Formula weight	526.56
Temperature [K]	100(2)
Crystal system	monoclinic
Space group (number)	$P2_1/c$ (14)
a [Å]	12.5433(9)
b [Å]	13.2318(10)
c [Å]	15.9266(10)
α [°]	90
β [°]	99.392(2)
γ [°]	90
Volume [Å^3]	2607.9(3)
Z	4
ρ_{calc} [gcm^{-3}]	1.341
μ [mm^{-1}]	0.146
F(000)	1112
Crystal size [mm^3]	0.043×0.045×0.196
Crystal colour	orange
Crystal shape	needle
Radiation	MoK_α (λ=0.71073 Å)
2θ range [°]	4.02 to 52.87 (0.80 Å)
Index ranges	$-15 \le h \le 15$<br>$-16 \le k \le 16$<br>$-19 \le l \le 19$
Reflections collected	136157
Independent reflections	5345<br>R_{int} = 0.0701<br>R_{sigma} = 0.0209
Completeness to<br>θ = 25.242°	99.9
Data / Restraints / Parameters	5345 / 93 / 353
Goodness-of-fit on F^2	1.040
Final R indexes<br>[$I \geq 2\sigma(I)$]	R_1 = 0.0372<br>wR_2 = 0.0919
Final R indexes<br>[all data]	R_1 = 0.0493<br>wR_2 = 0.1006
Largest peak/hole [eÅ^{-3}]	0.47/−0.33

Atom	*x*	*y*	*z*	U_{eq}
P1	0.53704(3)	0.16940(3)	0.66581(2)	0.02024(11)
O1	0.83511(9)	0.56730(10)	0.80717(8)	0.0325(3)
O2	0.67205(9)	0.26338(9)	0.84698(7)	0.0266(3)
O3	0.49324(9)	0.09964(9)	0.72393(7)	0.0270(3)
N1	0.75749(10)	0.41236(11)	0.82488(8)	0.0242(3)
N2	0.69496(10)	0.51461(10)	0.70900(8)	0.0230(3)
N3	0.54442(10)	0.48219(10)	0.61599(8)	0.0223(3)
N4	0.51934(10)	0.29355(10)	0.68466(8)	0.0219(3)
C1	1.08887(17)	0.27467(18)	0.84240(14)	0.0477(5)
H1A	1.058606	0.207125	0.829320	0.072
H1B	1.133120	0.274330	0.899122	0.072
H1C	1.133800	0.293624	0.800016	0.072
H1D	1.092429	0.228892	0.794565	0.072
H1E	1.136145	0.249475	0.893191	0.072
H1F	1.112569	0.342289	0.828395	0.072
C2	0.99710(17)	0.35088(17)	0.84046(14)	0.0355(5)
H2A	1.027419	0.417927	0.858711	0.043
H2B	0.957697	0.357262	0.781562	0.043
C3	0.91960(16)	0.31780(17)	0.89866(13)	0.0312(4)
H3A	0.961084	0.304688	0.956011	0.037
H3B	0.885243	0.253482	0.877042	0.037
C3A	0.9507(10)	0.3843(11)	0.9002(9)	0.0312(4)
H3AA	0.973669	0.437899	0.863527	0.037
H3AB	0.995616	0.388883	0.957244	0.037
C2A	0.9618(10)	0.2804(11)	0.8613(10)	0.0355(5)
H2AA	0.909897	0.272630	0.807720	0.043
H2AB	0.948182	0.226408	0.901268	0.043
C4	0.83046(14)	0.39562(15)	0.90643(11)	0.0306(4)
H4A	0.787500	0.371791	0.949466	0.037
H4B	0.864459	0.460621	0.926604	0.037
H4C	0.806728	0.333836	0.933197	0.037
H4D	0.822355	0.453033	0.944749	0.037
C5	0.76801(12)	0.50245(13)	0.78247(10)	0.0255(3)
C6	0.61592(12)	0.44610(12)	0.68354(9)	0.0213(3)
C7	0.60645(12)	0.35725(12)	0.72242(10)	0.0213(3)
C8	0.67668(12)	0.33708(12)	0.80137(10)	0.0227(3)
C9	0.67911(14)	0.60651(13)	0.65690(11)	0.0279(4)
H9A	0.733427	0.611571	0.618435	0.034
H9B	0.683074	0.667840	0.692885	0.034
C10	0.56465(13)	0.59152(12)	0.60638(10)	0.0252(3)
H10A	0.511142	0.632736	0.630532	0.030
H10B	0.561873	0.609759	0.545780	0.030
C11	0.43809(12)	0.44375(12)	0.61125(9)	0.0213(3)
C12	0.34858(13)	0.49759(13)	0.57297(10)	0.0252(3)
H12	0.358159	0.562667	0.550131	0.030
C13	0.24481(13)	0.45812(13)	0.56734(10)	0.0267(4)
C14	0.15007(15)	0.51920(16)	0.52373(13)	0.0374(4)
H14A	0.175777	0.584504	0.505983	0.056
H14B	0.098966	0.530328	0.563257	0.056
H14C	0.113937	0.482417	0.473656	0.056
C15	0.23065(13)	0.36346(13)	0.60293(10)	0.0253(3)
C16	0.11950(13)	0.31821(14)	0.59908(12)	0.0328(4)
H16A	0.082643	0.316984	0.539897	0.049
H16B	0.077654	0.359156	0.633352	0.049
H16C	0.126045	0.249125	0.621507	0.049

C17	0.32125(13)	0.31069(13)	0.64328(10)	0.0243(3)
H17	0.311494	0.247222	0.668863	0.029
C18	0.42490(12)	0.34849(12)	0.64693(9)	0.0212(3)
C19	0.68149(12)	0.15941(12)	0.67175(10)	0.0214(3)
C20	0.73484(14)	0.08688(12)	0.72635(10)	0.0259(3)
H20	0.695726	0.047450	0.760836	0.031
C21	0.84498(14)	0.07204(14)	0.73053(11)	0.0319(4)
H21	0.880946	0.021905	0.767440	0.038
C22	0.90287(14)	0.12996(15)	0.68115(12)	0.0327(4)
H22	0.978089	0.118713	0.683385	0.039
C23	0.85076(13)	0.20435(14)	0.62847(11)	0.0295(4)
H23	0.890664	0.245148	0.595545	0.035
C24	0.74060(13)	0.21937(12)	0.62370(10)	0.0239(3)
H24	0.705206	0.270571	0.587652	0.029
C25	0.47118(12)	0.14671(12)	0.55756(10)	0.0216(3)
C26	0.50674(13)	0.18528(14)	0.48599(11)	0.0282(4)
H26	0.569817	0.226151	0.492366	0.034
C27	0.45096(13)	0.16468(13)	0.40507(11)	0.0275(4)
H27	0.476427	0.190982	0.356489	0.033
C28	0.35959(15)	0.10670(13)	0.39512(11)	0.0326(4)
H28	0.320213	0.094572	0.339852	0.039
C29	0.32473(18)	0.06586(17)	0.46555(13)	0.0461(5)
H29	0.261870	0.024678	0.458701	0.055
C30	0.38122(16)	0.08477(15)	0.54635(12)	0.0369(4)
H30	0.357881	0.054864	0.594459	0.044

U_{eq} is defined as 1/3 of the trace of the orthogonalized U_{ij} tensor.

Table S 2: Anisotropic displacement parameters (Å^2) for compound 11. The anisotropic displacement factor exponent takes the form: $-2\pi^2[\, h^2(a^*)^2U_{11} + k^2(b^*)^2U_{22} + \ldots + 2hka^*b^*U_{12}\,]$.

**Atom**	***U*₁₁**	***U*₂₂**	***U*₃₃**	***U*₂₃**	***U*₁₃**	***U*₁₂**
P1	0.0200(2)	0.0202(2)	0.0217(2)	0.00097(15)	0.00698(15)	−0.00015(15)
O1	0.0238(6)	0.0357(7)	0.0370(7)	−0.0041(5)	0.0019(5)	−0.0078(5)
O2	0.0265(6)	0.0284(6)	0.0254(6)	0.0014(5)	0.0059(5)	0.0047(5)
O3	0.0287(6)	0.0276(6)	0.0265(6)	0.0043(5)	0.0100(5)	−0.0024(5)
N1	0.0196(7)	0.0289(7)	0.0237(7)	−0.0026(6)	0.0027(5)	0.0021(5)
N2	0.0196(6)	0.0242(7)	0.0263(7)	−0.0014(5)	0.0066(5)	−0.0017(5)
N3	0.0219(7)	0.0246(7)	0.0208(6)	0.0009(5)	0.0047(5)	−0.0016(5)
N4	0.0189(6)	0.0226(7)	0.0241(7)	−0.0024(5)	0.0032(5)	0.0011(5)
C1	0.0354(9)	0.0598(13)	0.0471(12)	−0.0071(10)	0.0040(8)	0.0139(9)
C2	0.0314(9)	0.0397(10)	0.0358(10)	0.0026(8)	0.0066(7)	0.0046(7)
C3	0.0245(8)	0.0367(10)	0.0312(9)	0.0033(8)	0.0011(7)	0.0027(7)
C3A	0.0245(8)	0.0367(10)	0.0312(9)	0.0033(8)	0.0011(7)	0.0027(7)
C2A	0.0314(9)	0.0397(10)	0.0358(10)	0.0026(8)	0.0066(7)	0.0046(7)
C4	0.0244(8)	0.0414(10)	0.0249(8)	−0.0009(7)	0.0005(6)	0.0025(7)
C5	0.0175(8)	0.0322(9)	0.0274(8)	−0.0050(7)	0.0059(6)	0.0006(7)
C6	0.0176(7)	0.0273(8)	0.0203(7)	−0.0048(6)	0.0070(6)	0.0010(6)
C7	0.0185(7)	0.0222(8)	0.0235(8)	−0.0038(6)	0.0047(6)	0.0012(6)
C8	0.0184(7)	0.0253(8)	0.0256(8)	−0.0049(6)	0.0066(6)	0.0037(6)
C9	0.0278(9)	0.0295(9)	0.0273(8)	0.0017(7)	0.0066(7)	−0.0060(7)
C10	0.0283(9)	0.0256(8)	0.0223(8)	0.0015(6)	0.0061(6)	−0.0041(7)
C11	0.0208(8)	0.0254(8)	0.0184(7)	−0.0023(6)	0.0051(6)	−0.0020(6)
C12	0.0264(8)	0.0267(8)	0.0224(8)	0.0013(6)	0.0042(6)	0.0006(7)
C13	0.0232(8)	0.0312(9)	0.0252(8)	−0.0011(7)	0.0021(6)	0.0031(7)
C14	0.0263(9)	0.0426(11)	0.0406(10)	0.0074(8)	−0.0020(8)	0.0035(8)
C15	0.0208(8)	0.0296(9)	0.0255(8)	−0.0043(7)	0.0042(6)	−0.0009(7)
C16	0.0223(8)	0.0357(10)	0.0396(10)	−0.0021(8)	0.0024(7)	−0.0031(7)
C17	0.0241(8)	0.0236(8)	0.0260(8)	−0.0023(6)	0.0063(6)	−0.0018(6)

C18	0.0204(8)	0.0232(8)	0.0201(7)	−0.0027(6)	0.0038(6)	0.0023(6)
C19	0.0219(8)	0.0211(8)	0.0217(7)	−0.0044(6)	0.0055(6)	0.0007(6)
C20	0.0297(9)	0.0225(8)	0.0257(8)	−0.0027(6)	0.0049(6)	0.0025(7)
C21	0.0306(9)	0.0308(9)	0.0324(9)	−0.0044(7)	−0.0007(7)	0.0085(7)
C22	0.0200(8)	0.0388(10)	0.0386(10)	−0.0129(8)	0.0024(7)	0.0038(7)
C23	0.0231(8)	0.0356(10)	0.0313(9)	−0.0086(7)	0.0092(7)	−0.0048(7)
C24	0.0234(8)	0.0251(8)	0.0235(8)	−0.0030(6)	0.0051(6)	−0.0008(6)
C25	0.0210(8)	0.0189(8)	0.0259(8)	−0.0003(6)	0.0064(6)	0.0013(6)
C26	0.0226(8)	0.0336(9)	0.0286(8)	0.0030(7)	0.0050(6)	−0.0045(7)
C27	0.0265(8)	0.0309(9)	0.0257(8)	0.0036(7)	0.0066(6)	0.0023(7)
C28	0.0419(10)	0.0270(9)	0.0272(9)	−0.0032(7)	0.0003(7)	−0.0045(8)
C29	0.0536(13)	0.0475(12)	0.0354(10)	−0.0013(9)	0.0016(9)	−0.0320(10)
C30	0.0445(11)	0.0373(11)	0.0296(9)	0.0016(8)	0.0079(8)	−0.0183(9)

Table S 3: Bond lengths and angles for compound 11.

**Atom–Atom**	**Length [Å]**
P1–O3	1.4753(11)
P1–N4	1.6911(14)
P1–C19	1.8037(16)
P1–C25	1.8111(16)
O1–C5	1.221(2)
O2–C8	1.223(2)
N1–C5	1.387(2)
N1–C8	1.427(2)
N1–C4	1.478(2)
N2–C6	1.356(2)
N2–C5	1.373(2)
N2–C9	1.467(2)
N3–C6	1.369(2)
N3–C11	1.418(2)
N3–C10	1.481(2)
N4–C7	1.432(2)
N4–C18	1.4354(19)
C1–C2	1.527(3)
C1–C2A	1.671(13)
C1–H1A	0.9800
C1–H1B	0.9800
C1–H1C	0.9800
C1–H1D	0.9800
C1–H1E	0.9800

C1–H1F	0.9800
C2–C3	1.513(3)
C2–H2A	0.9900
C2–H2B	0.9900
C3–C4	1.540(3)
C3–H3A	0.9900
C3–H3B	0.9900
C3A–C2A	1.525(15)
C3A–C4	1.535(12)
C3A–H3AA	0.9900
C3A–H3AB	0.9900
C2A–H2AA	0.9900
C2A–H2AB	0.9900
C4–H4A	0.9900
C4–H4B	0.9900
C4–H4C	0.9900
C4–H4D	0.9900
C6–C7	1.343(2)
C7–C8	1.438(2)
C9–C10	1.540(2)
C9–H9A	0.9900
C9–H9B	0.9900
C10–H10A	0.9900
C10–H10B	0.9900
C11–C12	1.384(2)
C11–C18	1.404(2)
C12–C13	1.392(2)
C12–H12	0.9500
C13–C15	1.398(2)
C13–C14	1.509(2)
C14–H14A	0.9800
C14–H14B	0.9800
C14–H14C	0.9800
C15–C17	1.398(2)
C15–C16	1.509(2)
C16–H16A	0.9800

C16–H16B	0.9800
C16–H16C	0.9800
C17–C18	1.385(2)
C17–H17	0.9500
C19–C20	1.391(2)
C19–C24	1.396(2)
C20–C21	1.386(2)
C20–H20	0.9500
C21–C22	1.386(3)
C21–H21	0.9500
C22–C23	1.386(3)
C22–H22	0.9500
C23–C24	1.386(2)
C23–H23	0.9500
C24–H24	0.9500
C25–C30	1.382(2)
C25–C26	1.387(2)
C26–C27	1.389(2)
C26–H26	0.9500
C27–C28	1.367(2)
C27–H27	0.9500
C28–C29	1.379(3)
C28–H28	0.9500
C29–C30	1.387(3)
C29–H29	0.9500
C30–H30	0.9500

**Atom–Atom–Atom**	**Angle [°]**
O3–P1–N4	115.04(7)
O3–P1–C19	113.26(7)
N4–P1–C19	102.82(7)
O3–P1–C25	109.56(7)
N4–P1–C25	106.10(7)
C19–P1–C25	109.63(7)
C5–N1–C8	126.31(13)
C5–N1–C4	117.52(14)

C8–N1–C4	115.94(14)
C6–N2–C5	122.04(14)
C6–N2–C9	111.10(13)
C5–N2–C9	126.09(14)
C6–N3–C11	114.00(13)
C6–N3–C10	108.70(13)
C11–N3–C10	121.36(13)
C7–N4–C18	113.49(12)
C7–N4–P1	122.20(10)
C18–N4–P1	122.75(10)
C2–C1–H1A	109.5
C2–C1–H1B	109.5
H1A–C1–H1B	109.5
C2–C1–H1C	109.5
H1A–C1–H1C	109.5
H1B–C1–H1C	109.5
C2A–C1–H1D	109.5
C2A–C1–H1E	109.5
H1D–C1–H1E	109.5
C2A–C1–H1F	109.5
H1D–C1–H1F	109.5
H1E–C1–H1F	109.5
C3–C2–C1	110.74(18)
C3–C2–H2A	109.5
C1–C2–H2A	109.5
C3–C2–H2B	109.5
C1–C2–H2B	109.5
H2A–C2–H2B	108.1
C2–C3–C4	113.79(17)
C2–C3–H3A	108.8
C4–C3–H3A	108.8
C2–C3–H3B	108.8
C4–C3–H3B	108.8
H3A–C3–H3B	107.7
C2A–C3A–C4	105.6(10)
C2A–C3A–H3AA	110.6

C4–C3A–H3AA	110.6
C2A–C3A–H3AB	110.6
C4–C3A–H3AB	110.6
H3AA–C3A–H3AB	108.7
C3A–C2A–C1	105.4(9)
C3A–C2A–H2AA	110.7
C1–C2A–H2AA	110.7
C3A–C2A–H2AB	110.7
C1–C2A–H2AB	110.7
H2AA–C2A–H2AB	108.8
N1–C4–C3A	115.6(6)
N1–C4–C3	112.53(14)
N1–C4–H4A	109.1
C3–C4–H4A	109.1
N1–C4–H4B	109.1
C3–C4–H4B	109.1
H4A–C4–H4B	107.8
N1–C4–H4C	108.4
C3A–C4–H4C	108.4
N1–C4–H4D	108.4
C3A–C4–H4D	108.4
H4C–C4–H4D	107.5
O1–C5–N2	121.42(16)
O1–C5–N1	124.22(15)
N2–C5–N1	114.36(14)
C7–C6–N2	124.25(15)
C7–C6–N3	124.87(14)
N2–C6–N3	110.80(14)
C6–C7–N4	116.13(14)
C6–C7–C8	118.56(14)
N4–C7–C8	125.07(14)
O2–C8–N1	120.08(14)
O2–C8–C7	125.86(15)
N1–C8–C7	114.06(14)
N2–C9–C10	102.56(13)
N2–C9–H9A	111.3

C10–C9–H9A	111.3
N2–C9–H9B	111.3
C10–C9–H9B	111.3
H9A–C9–H9B	109.2
N3–C10–C9	103.46(13)
N3–C10–H10A	111.1
C9–C10–H10A	111.1
N3–C10–H10B	111.1
C9–C10–H10B	111.1
H10A–C10–H10B	109.0
C12–C11–C18	119.98(14)
C12–C11–N3	121.82(14)
C18–C11–N3	118.20(14)
C11–C12–C13	121.21(16)
C11–C12–H12	119.4
C13–C12–H12	119.4
C12–C13–C15	119.30(15)
C12–C13–C14	119.12(16)
C15–C13–C14	121.58(15)
C13–C14–H14A	109.5
C13–C14–H14B	109.5
H14A–C14–H14B	109.5
C13–C14–H14C	109.5
H14A–C14–H14C	109.5
H14B–C14–H14C	109.5
C17–C15–C13	119.11(15)
C17–C15–C16	119.79(15)
C13–C15–C16	121.10(15)
C15–C16–H16A	109.5
C15–C16–H16B	109.5
H16A–C16–H16B	109.5
C15–C16–H16C	109.5
H16A–C16–H16C	109.5
H16B–C16–H16C	109.5
C18–C17–C15	121.74(15)
C18–C17–H17	119.1

C15–C17–H17	119.1
C17–C18–C11	118.62(14)
C17–C18–N4	122.65(14)
C11–C18–N4	118.72(13)
C20–C19–C24	119.36(15)
C20–C19–P1	117.39(12)
C24–C19–P1	123.25(12)
C21–C20–C19	120.12(16)
C21–C20–H20	119.9
C19–C20–H20	119.9
C22–C21–C20	120.33(16)
C22–C21–H21	119.8
C20–C21–H21	119.8
C21–C22–C23	119.83(16)
C21–C22–H22	120.1
C23–C22–H22	120.1
C24–C23–C22	120.17(16)
C24–C23–H23	119.9
C22–C23–H23	119.9
C23–C24–C19	120.16(16)
C23–C24–H24	119.9
C19–C24–H24	119.9
C30–C25–C26	118.50(15)
C30–C25–P1	117.20(12)
C26–C25–P1	124.28(12)
C25–C26–C27	120.61(15)
C25–C26–H26	119.7
C27–C26–H26	119.7
C28–C27–C26	120.24(16)
C28–C27–H27	119.9
C26–C27–H27	119.9
C27–C28–C29	119.79(17)
C27–C28–H28	120.1
C29–C28–H28	120.1
C28–C29–C30	120.16(17)
C28–C29–H29	119.9

C30–C29–H29	119.9
C25–C30–C29	120.63(17)
C25–C30–H30	119.7
C29–C30–H30	119.7

Table S 4: Torsion angles for compound 11.

**Atom–Atom–Atom–Atom**	**Torsion Angle [°]**
O3–P1–N4–C7	106.36(12)
C19–P1–N4–C7	−17.24(13)
C25–P1–N4–C7	−132.35(12)
O3–P1–N4–C18	−88.81(13)
C19–P1–N4–C18	147.59(12)
C25–P1–N4–C18	32.48(14)
C1–C2–C3–C4	174.73(17)
C4–C3A–C2A–C1	172.6(8)
C5–N1–C4–C3A	−66.4(6)
C8–N1–C4–C3A	118.7(6)
C5–N1–C4–C3	−106.35(18)
C8–N1–C4–C3	78.79(18)
C2A–C3A–C4–N1	−73.0(11)
C2–C3–C4–N1	64.2(2)
C6–N2–C5–O1	−175.93(14)
C9–N2–C5–O1	−6.9(2)
C6–N2–C5–N1	3.5(2)
C9–N2–C5–N1	172.50(14)
C8–N1–C5–O1	176.08(15)
C4–N1–C5–O1	1.8(2)
C8–N1–C5–N2	−3.3(2)
C4–N1–C5–N2	−177.57(13)
C5–N2–C6–C7	−5.8(2)
C9–N2–C6–C7	−176.33(15)
C5–N2–C6–N3	170.97(13)
C9–N2–C6–N3	0.47(17)
C11–N3–C6–C7	25.8(2)
C10–N3–C6–C7	164.53(15)

C11–N3–C6–N2	−151.01(13)
C10–N3–C6–N2	−12.25(17)
N2–C6–C7–N4	−178.25(13)
N3–C6–C7–N4	5.4(2)
N2–C6–C7–C8	7.1(2)
N3–C6–C7–C8	−169.22(14)
C18–N4–C7–C6	−33.97(18)
P1–N4–C7–C6	132.14(13)
C18–N4–C7–C8	140.25(15)
P1–N4–C7–C8	−53.63(19)
C5–N1–C8–O2	−175.59(14)
C4–N1–C8–O2	−1.2(2)
C5–N1–C8–C7	4.7(2)
C4–N1–C8–C7	179.00(13)
C6–C7–C8–O2	174.14(15)
N4–C7–C8–O2	0.0(2)
C6–C7–C8–N1	−6.1(2)
N4–C7–C8–N1	179.77(13)
C6–N2–C9–C10	10.72(17)
C5–N2–C9–C10	−159.31(14)
C6–N3–C10–C9	18.12(16)
C11–N3–C10–C9	153.27(13)
N2–C9–C10–N3	−16.77(15)
C6–N3–C11–C12	152.93(15)
C10–N3–C11–C12	19.9(2)
C6–N3–C11–C18	−26.88(19)
C10–N3–C11–C18	−159.89(14)
C18–C11–C12–C13	−1.4(2)
N3–C11–C12–C13	178.77(14)
C11–C12–C13–C15	1.6(2)
C11–C12–C13–C14	−179.04(16)
C12–C13–C15–C17	−0.1(2)
C14–C13–C15–C17	−179.37(16)
C12–C13–C15–C16	179.38(15)
C14–C13–C15–C16	0.1(3)
C13–C15–C17–C18	−1.7(2)

C16–C15–C17–C18	178.79(15)
C15–C17–C18–C11	2.0(2)
C15–C17–C18–N4	−177.13(14)
C12–C11–C18–C17	−0.4(2)
N3–C11–C18–C17	179.44(13)
C12–C11–C18–N4	178.76(14)
N3–C11–C18–N4	−1.4(2)
C7–N4–C18–C17	−148.94(15)
P1–N4–C18–C17	45.03(19)
C7–N4–C18–C11	31.96(19)
P1–N4–C18–C11	−134.06(13)
O3–P1–C19–C20	2.21(15)
N4–P1–C19–C20	126.98(12)
C25–P1–C19–C20	−120.49(13)
O3–P1–C19–C24	−178.77(12)
N4–P1–C19–C24	−54.00(14)
C25–P1–C19–C24	58.53(15)
C24–C19–C20–C21	−2.3(2)
P1–C19–C20–C21	176.75(13)
C19–C20–C21–C22	0.7(3)
C20–C21–C22–C23	1.2(3)
C21–C22–C23–C24	−1.4(3)
C22–C23–C24–C19	−0.2(2)
C20–C19–C24–C23	2.1(2)
P1–C19–C24–C23	−176.94(12)
O3–P1–C25–C30	11.02(16)
N4–P1–C25–C30	−113.74(14)
C19–P1–C25–C30	135.89(14)
O3–P1–C25–C26	−167.33(14)
N4–P1–C25–C26	67.91(15)
C19–P1–C25–C26	−42.46(16)
C30–C25–C26–C27	2.1(3)
P1–C25–C26–C27	−179.59(13)
C25–C26–C27–C28	0.6(3)
C26–C27–C28–C29	−2.1(3)
C27–C28–C29–C30	1.0(3)

C26–C25–C30–C29	−3.2(3)
P1–C25–C30–C29	178.33(17)
C28–C29–C30–C25	1.7(3)

10.NMR Spectra

10.1 Compound SI-3

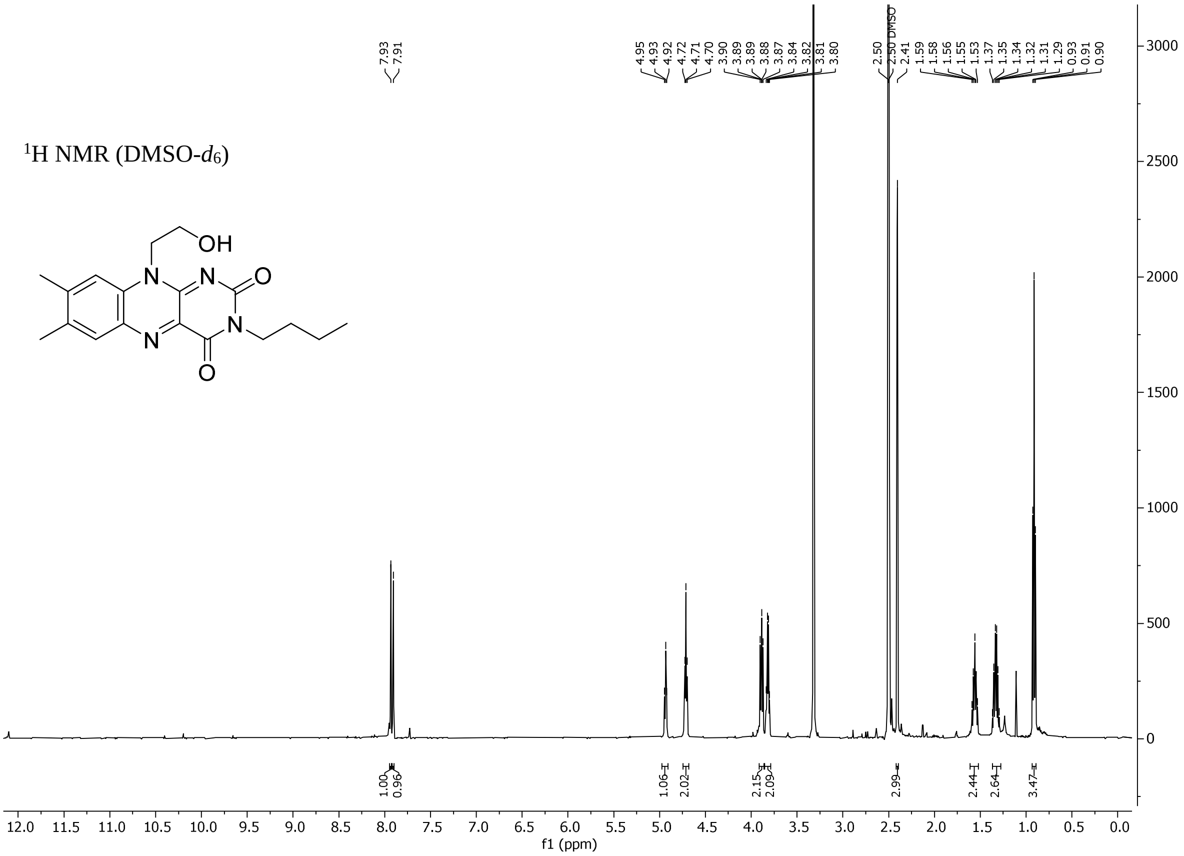

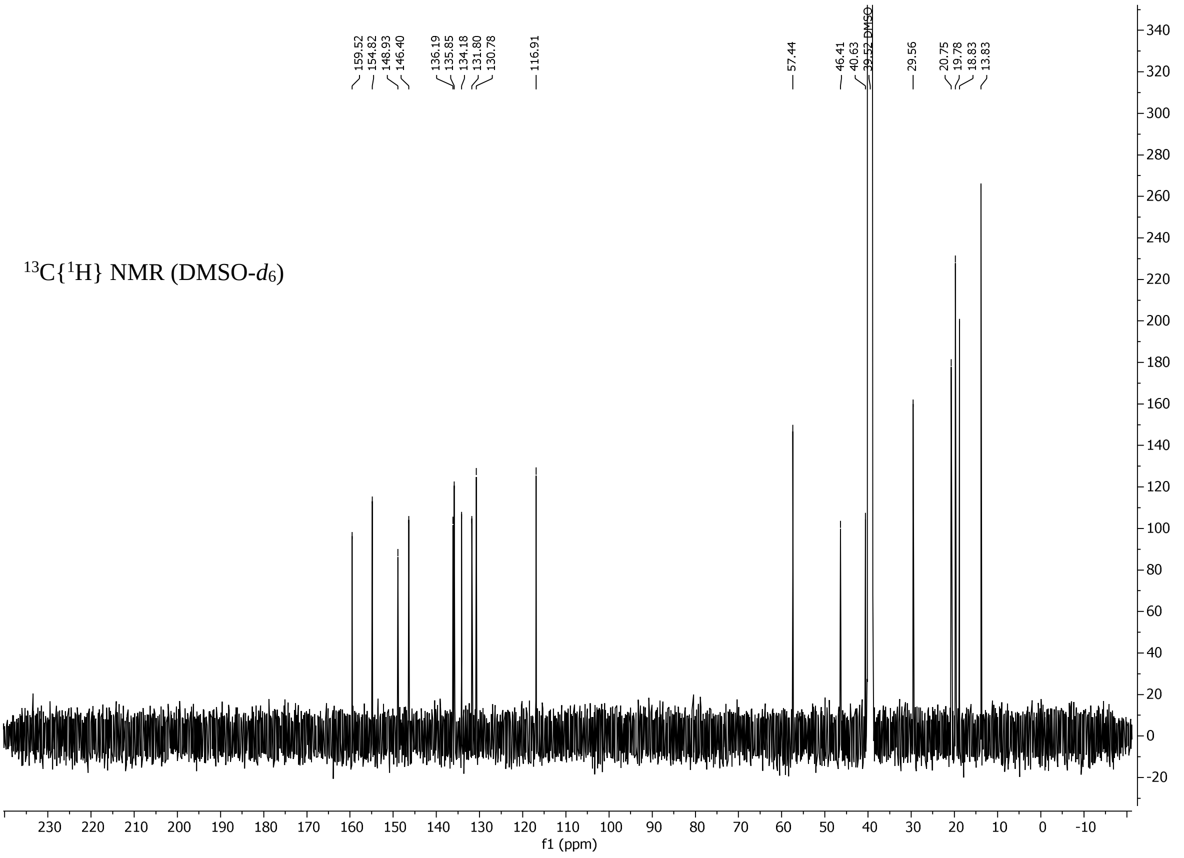

10.2 Compound 10

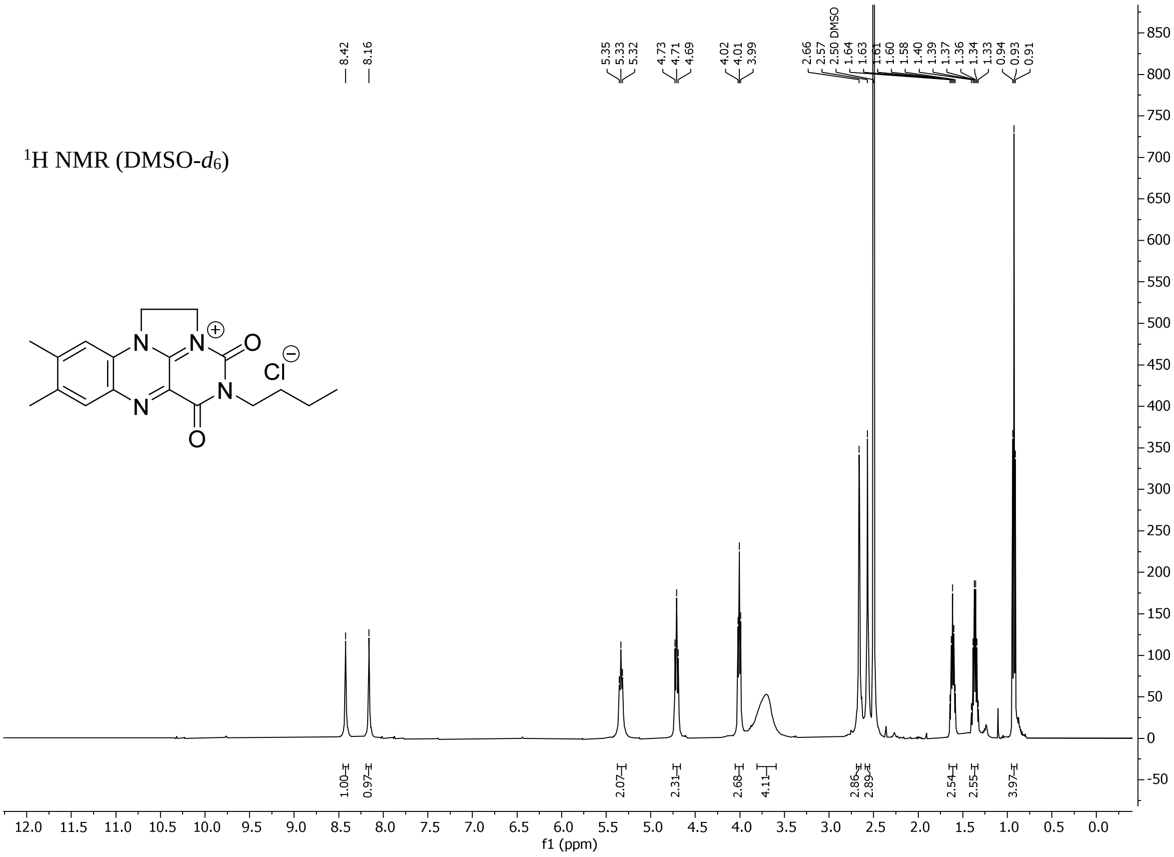

^{1}H NMR (DMSO-d_6)

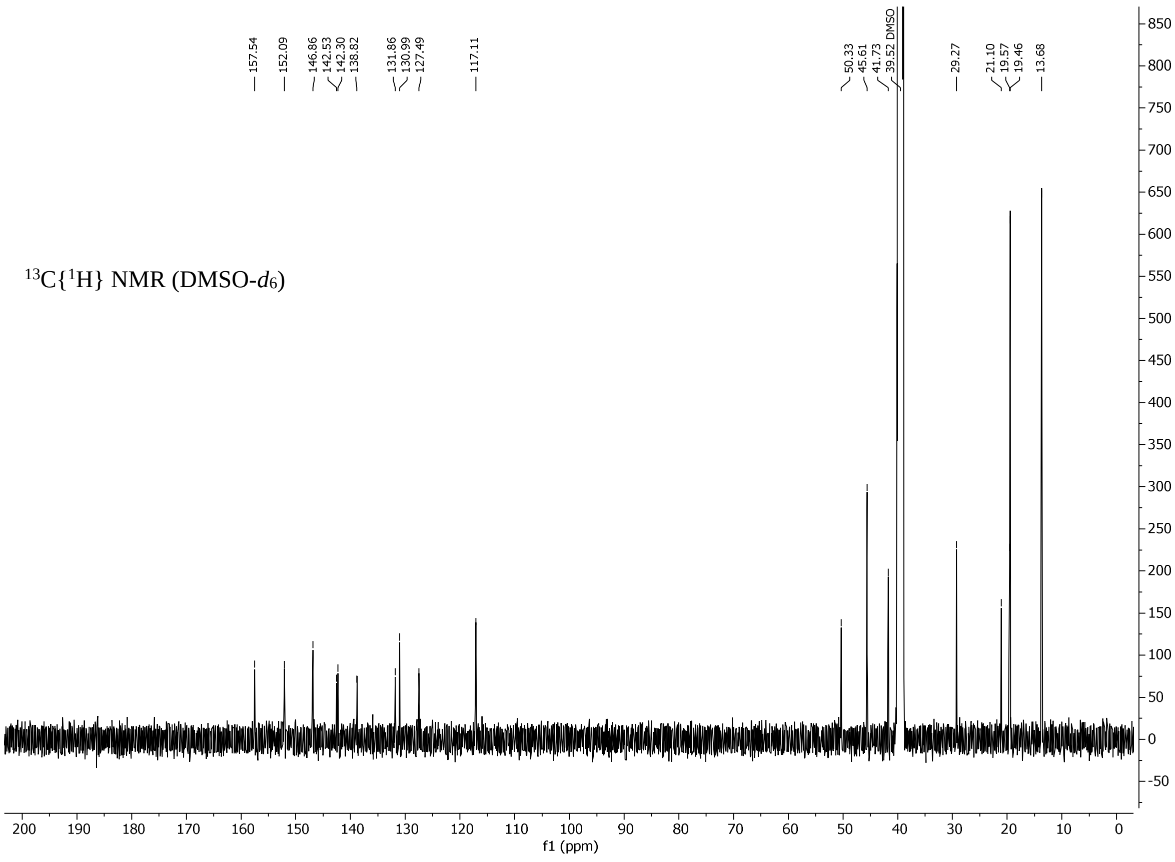

^{13}C{^{1}H} NMR (DMSO-d_6)

10.3 Compound 12

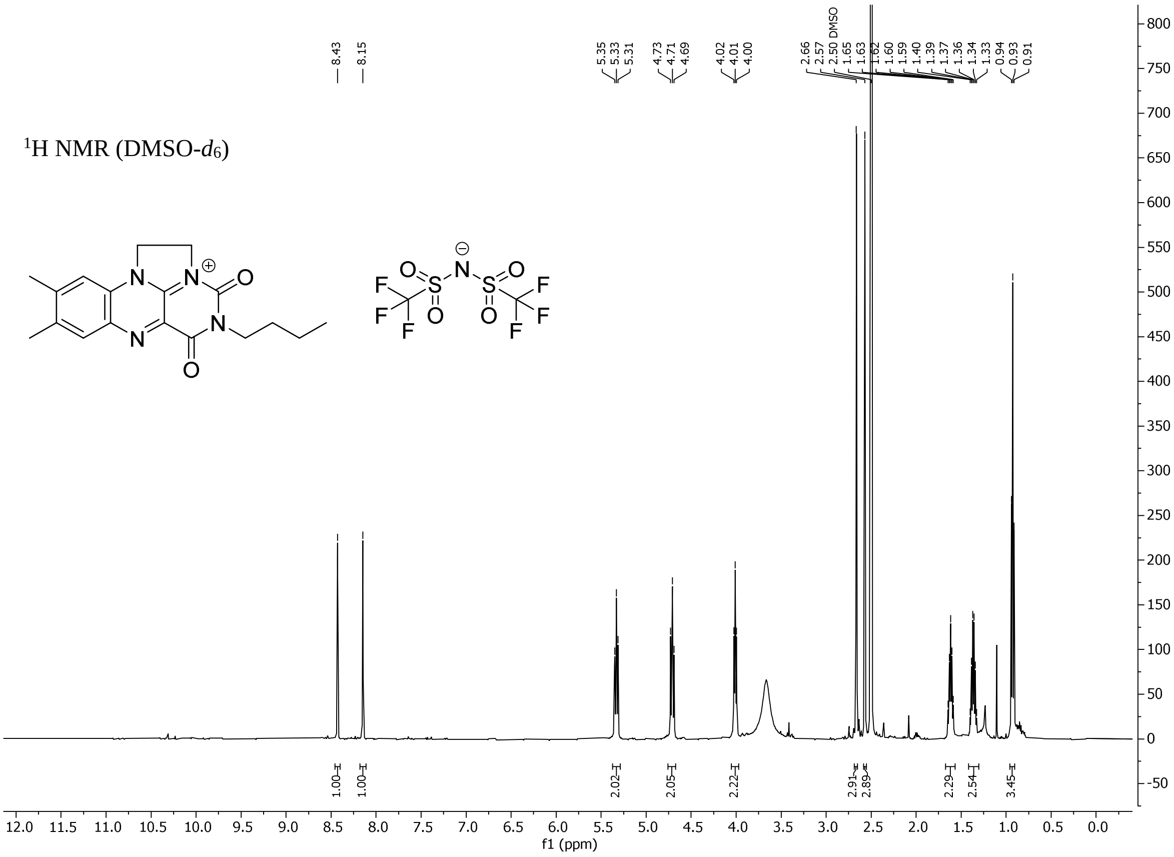

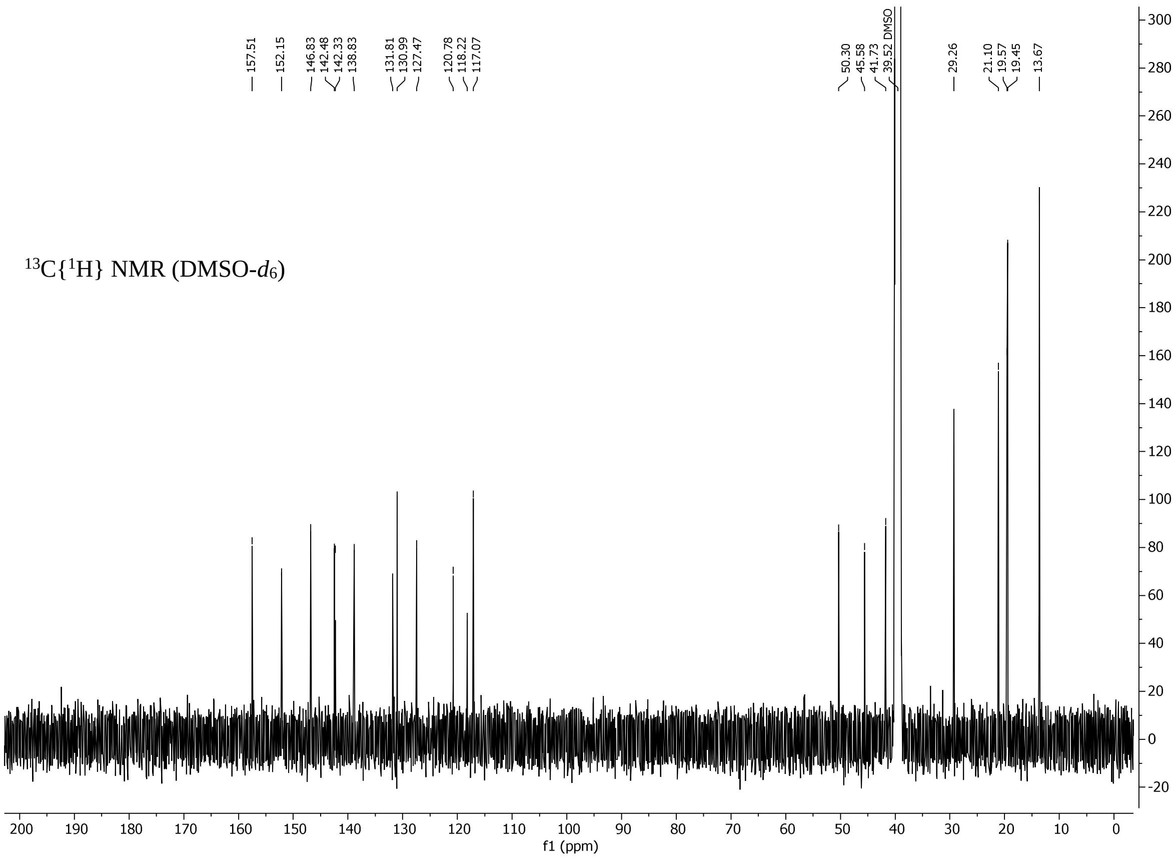

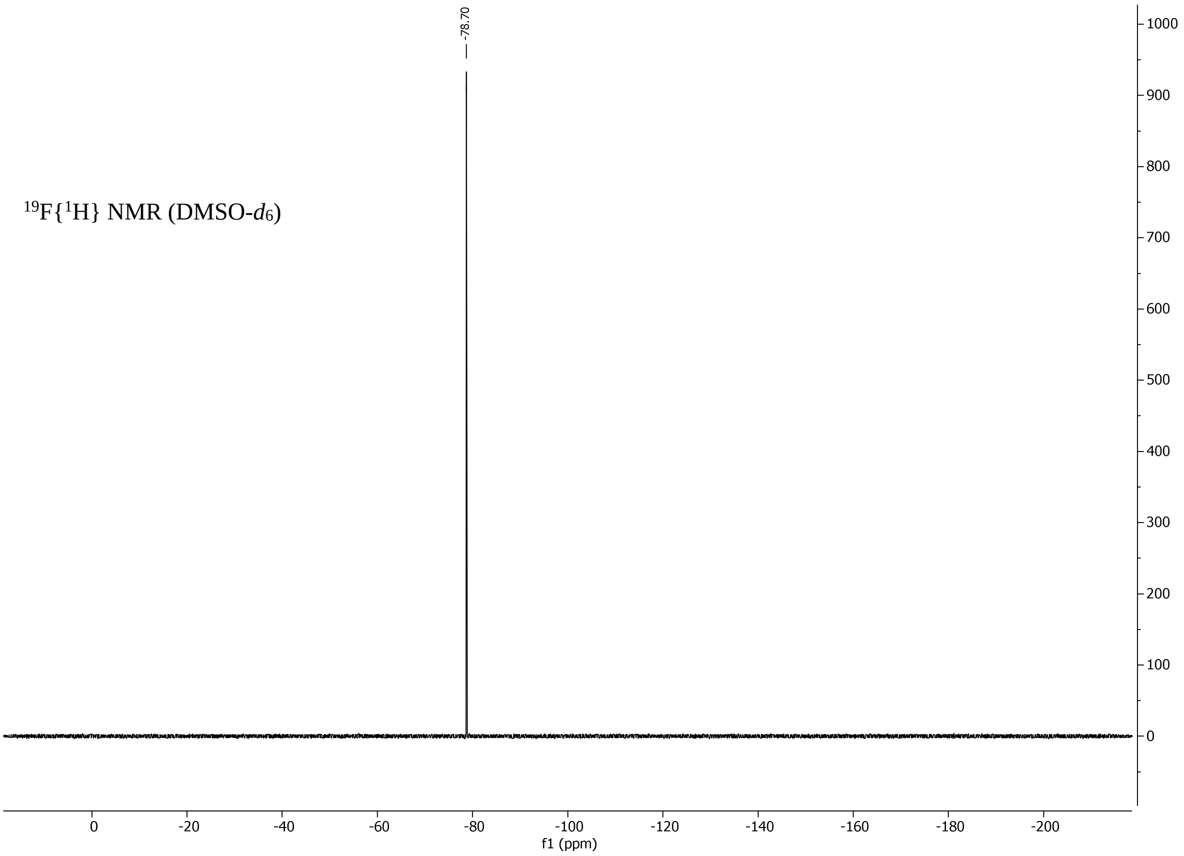
-78.70
19F{1H} NMR (DMSO-d6)
1000
900
800
700
600
500
400
300
200
100
0
0
-20
-40
-60
-80
-100
-120
-140
-160
-180
-200
f1 (ppm)

10.4 Compound 11

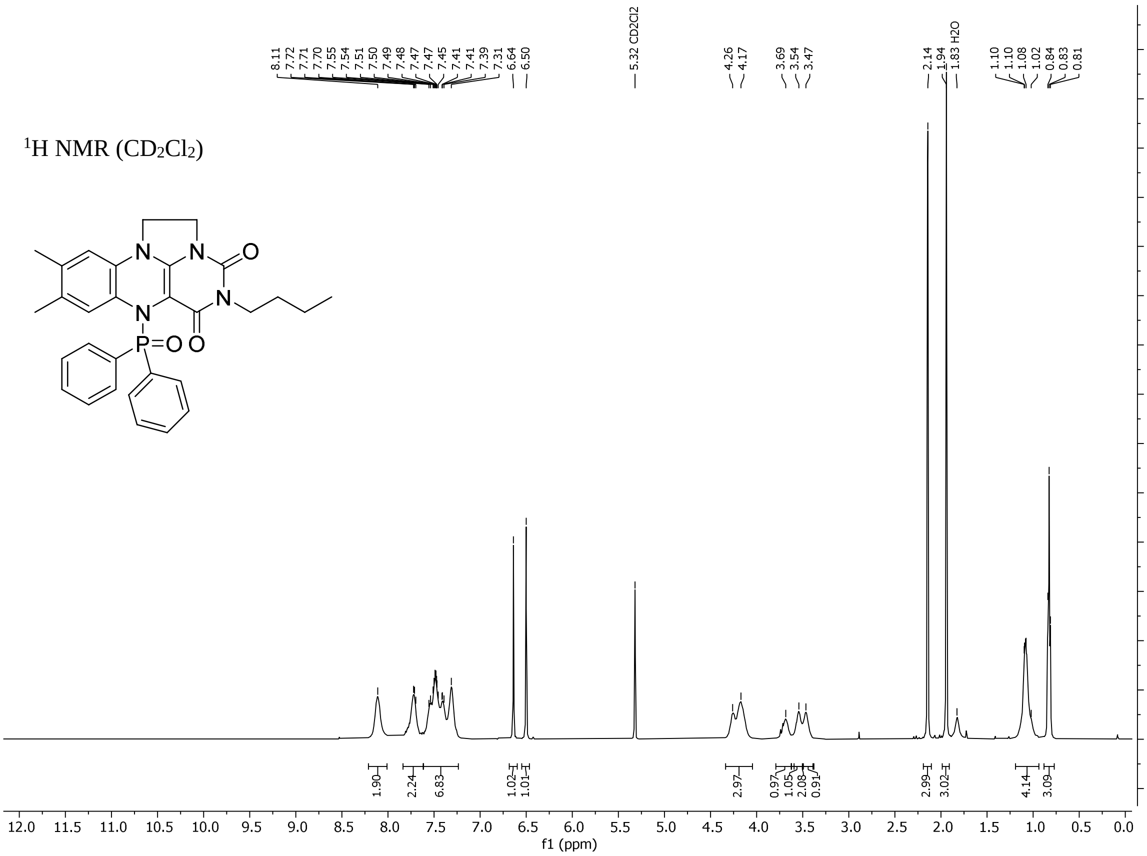

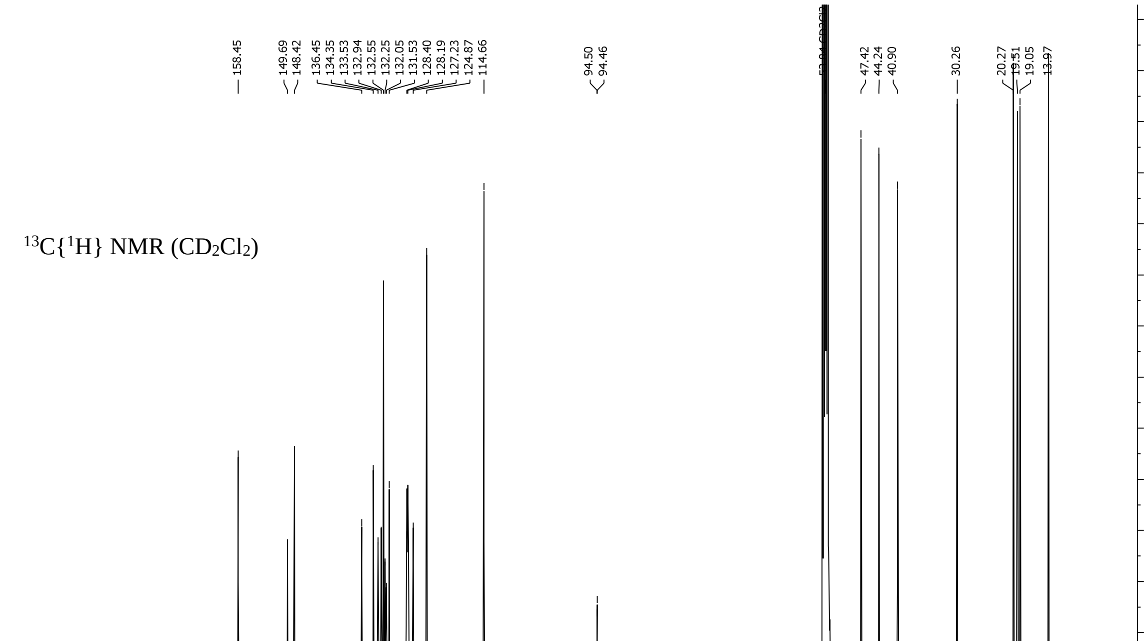

$^{31}P\{^1H\}$ NMR (CD_2Cl_2)

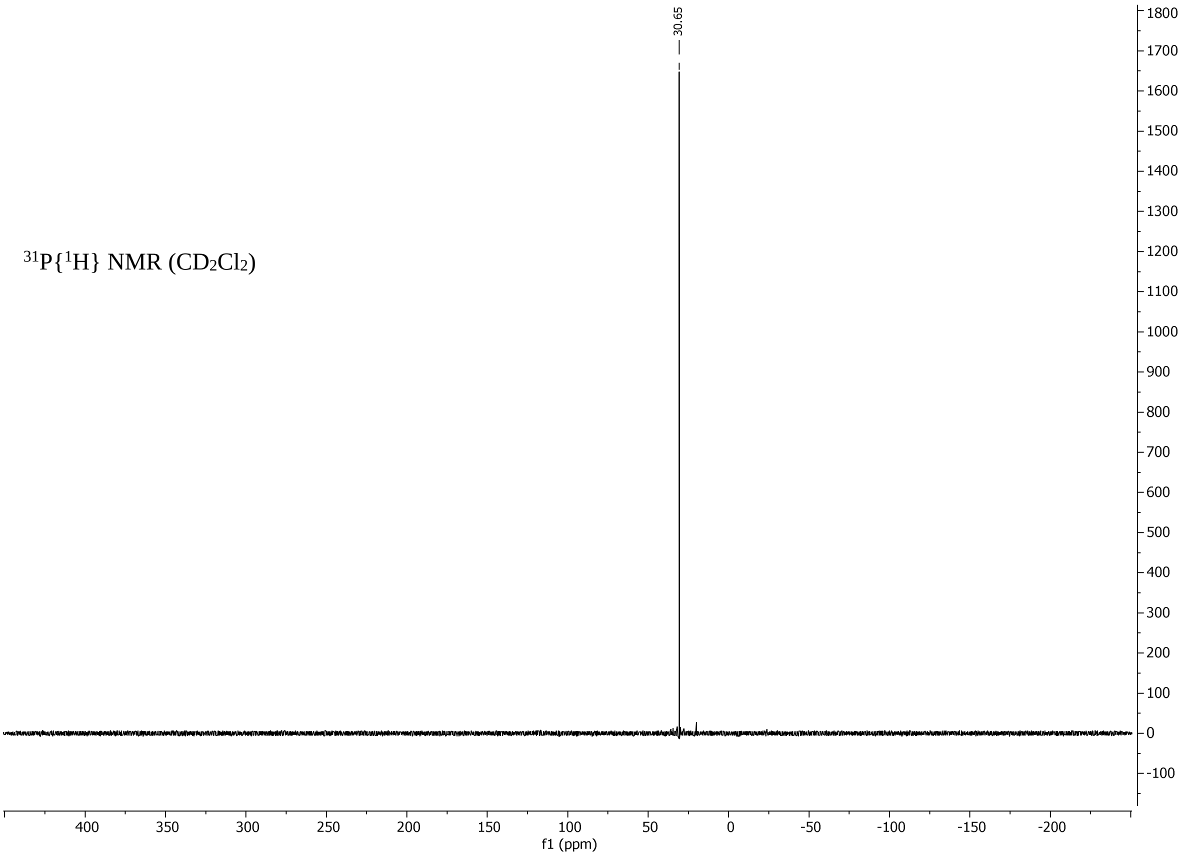

10.5 Compound SI-6

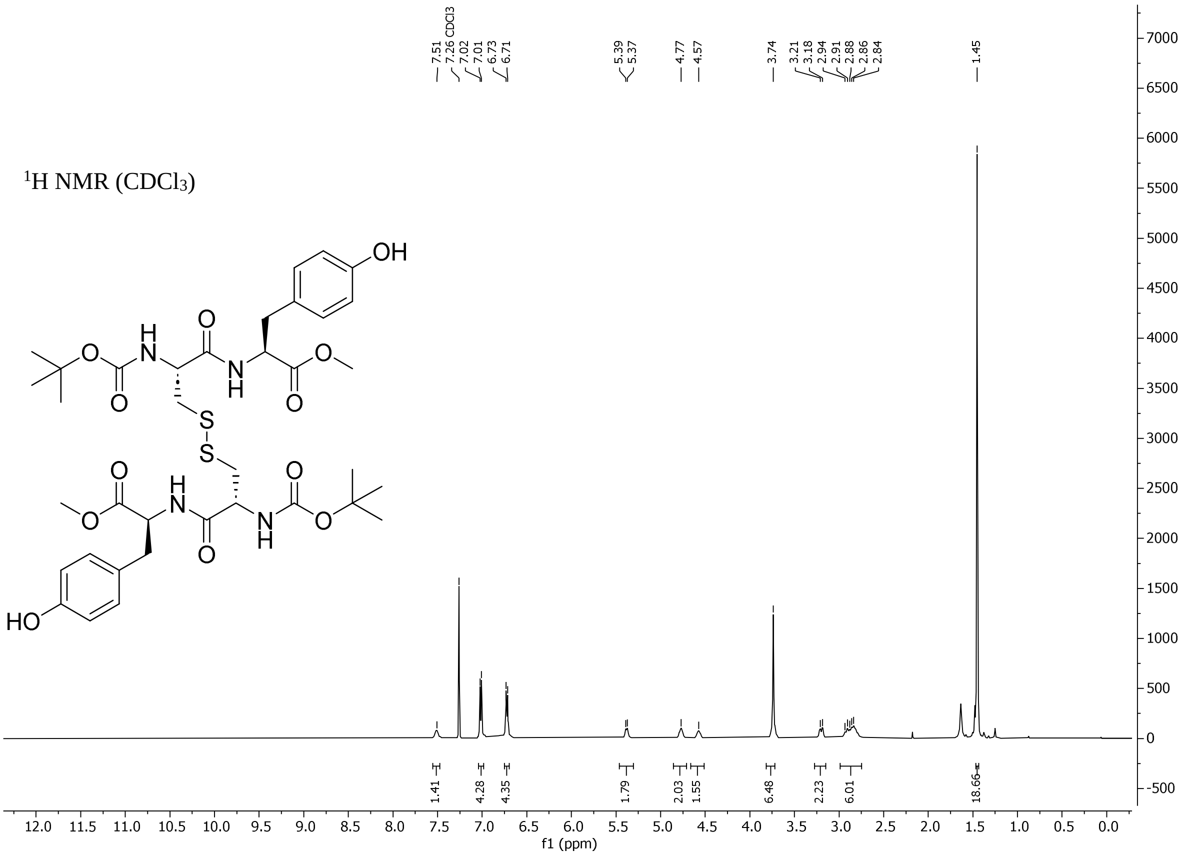

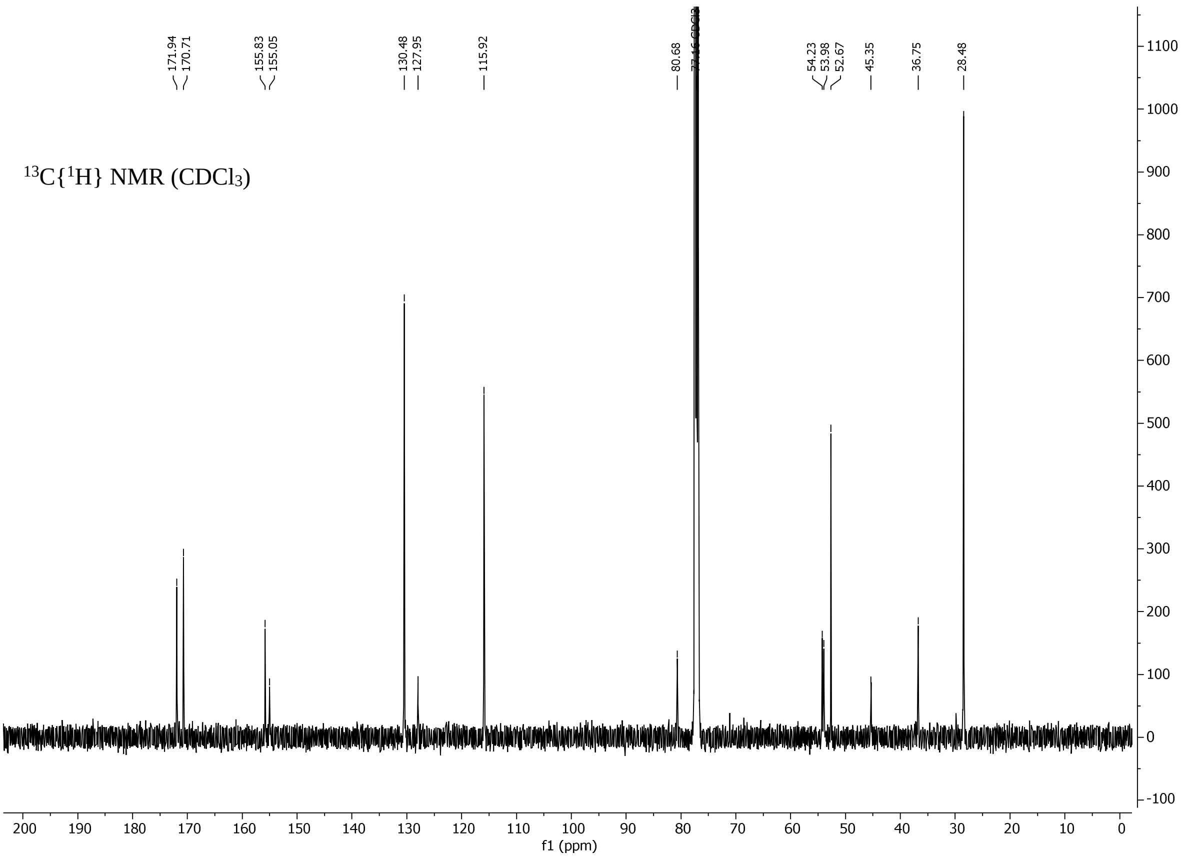

10.6 Compound SI-8

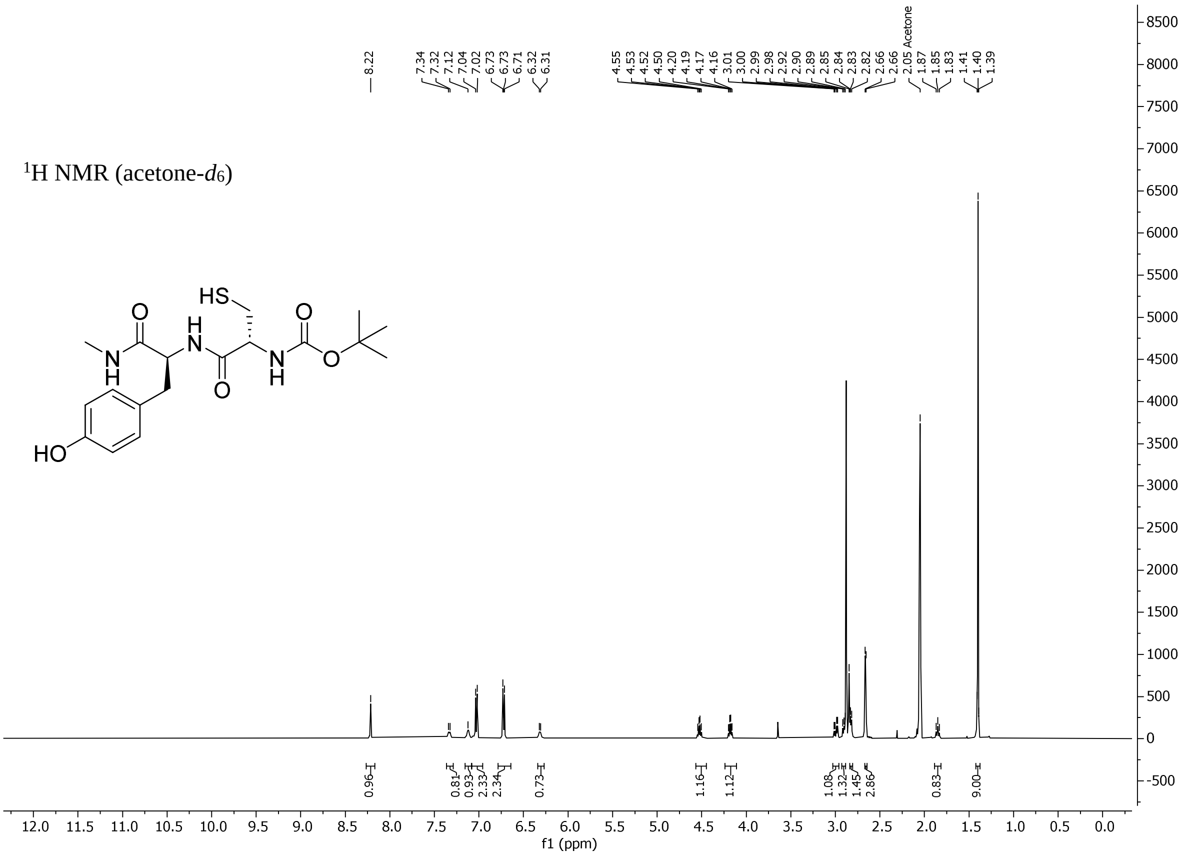

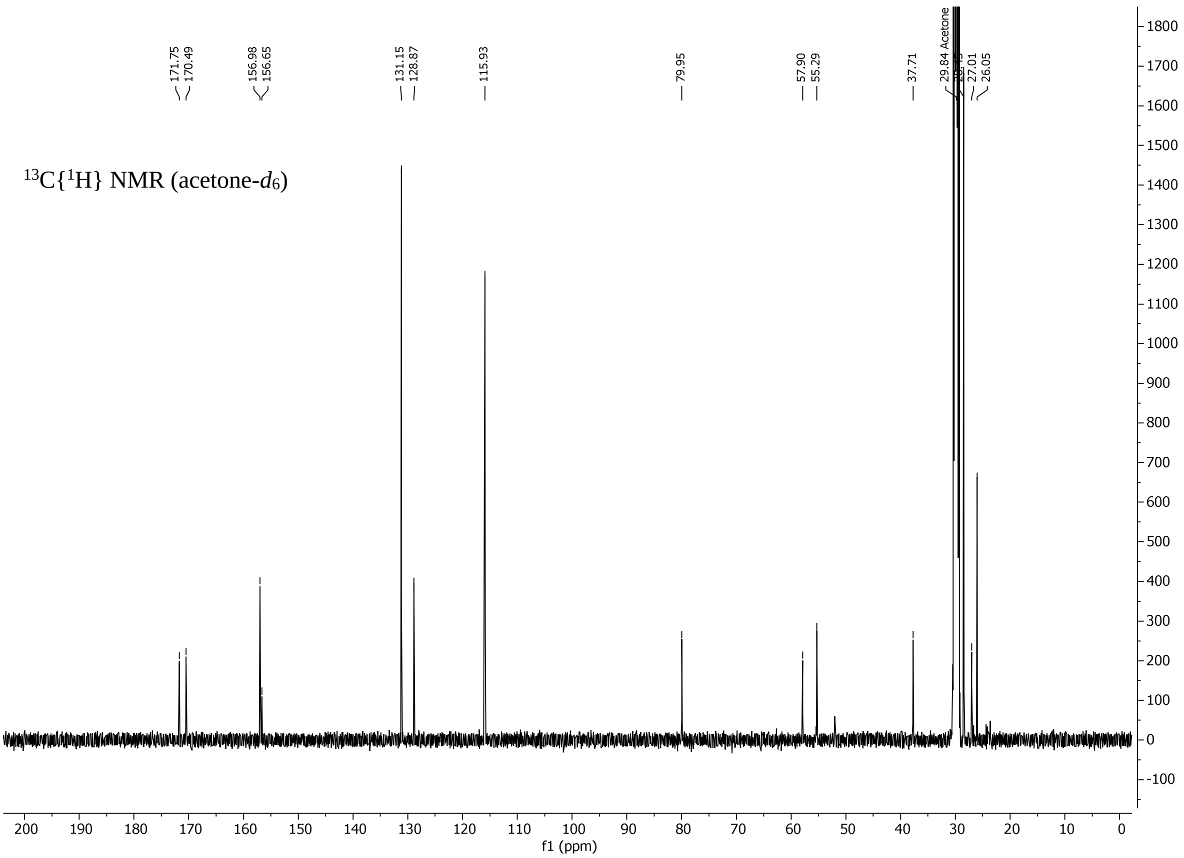

10.7 Compound 33

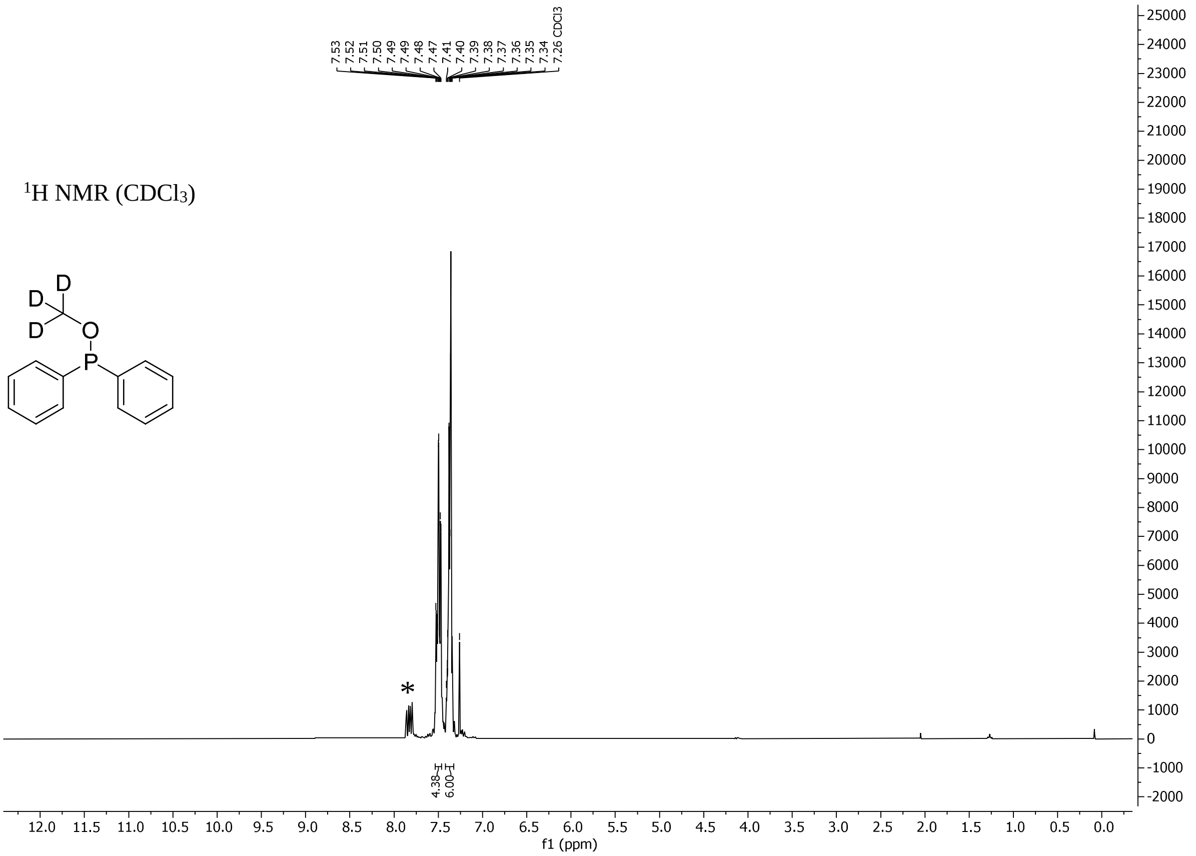

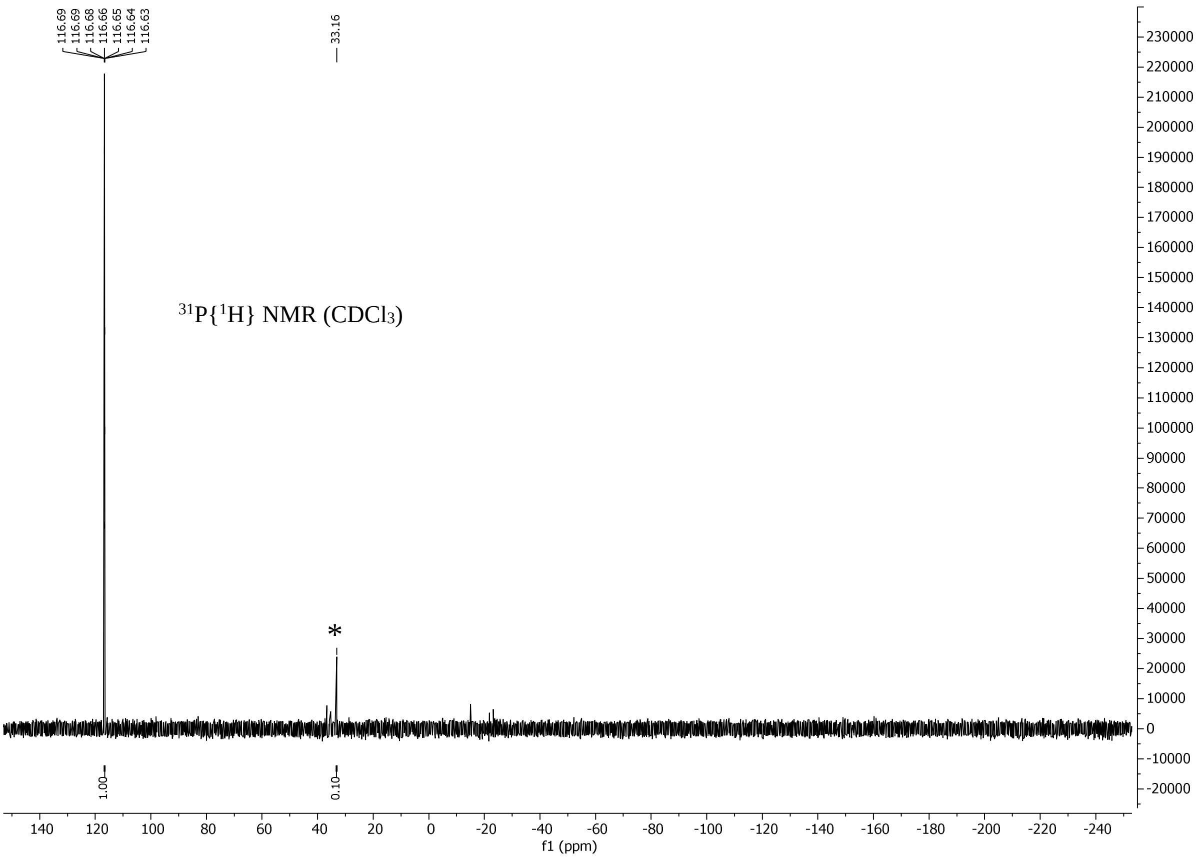

* indicates an oxidation side product (methyl-d_3 diphenylphosphinate).

10.8 Compound 19

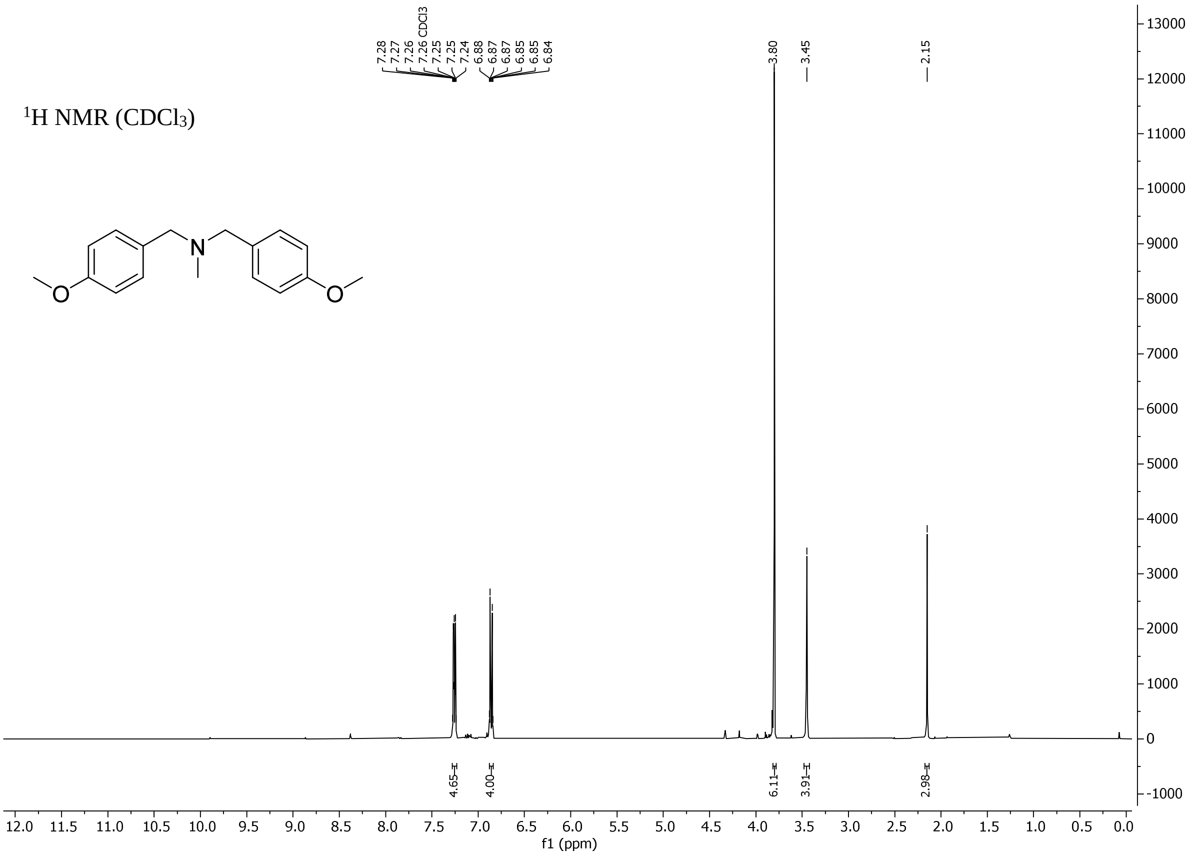

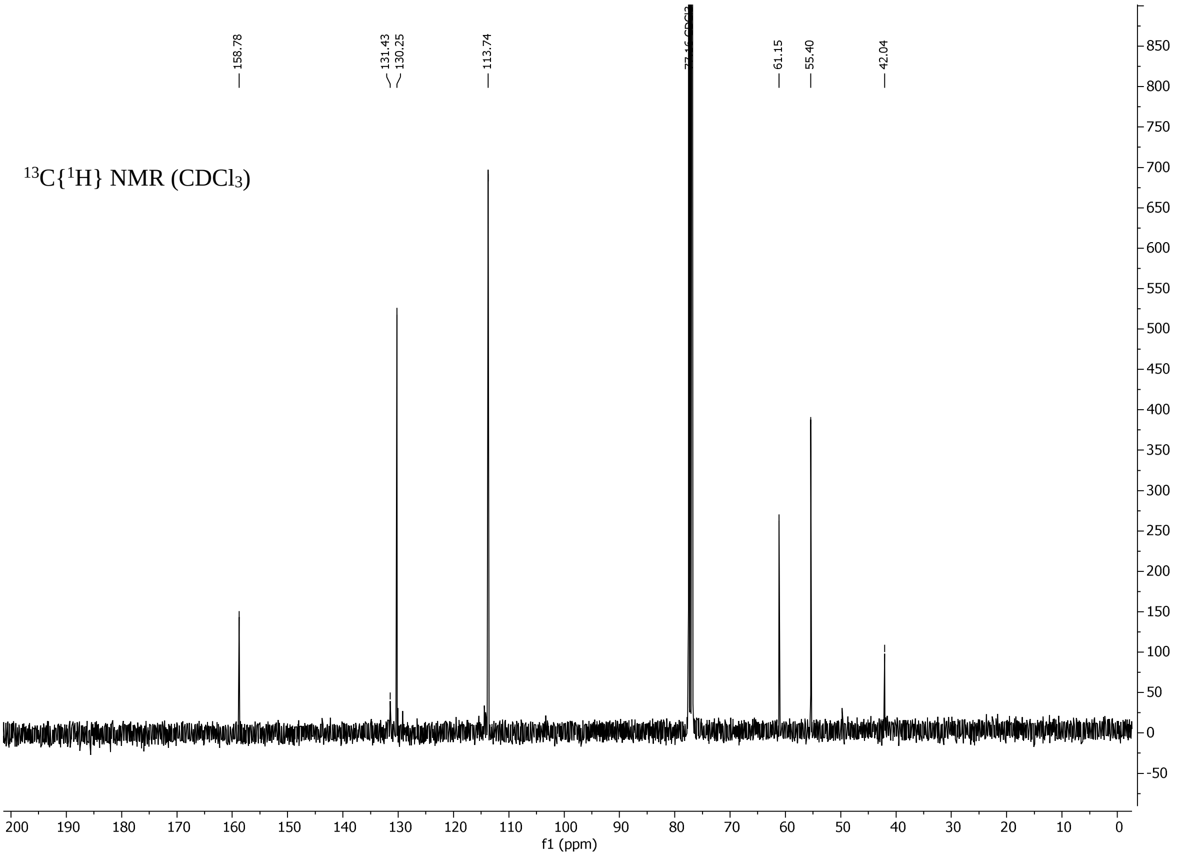

10.9 Compound 22

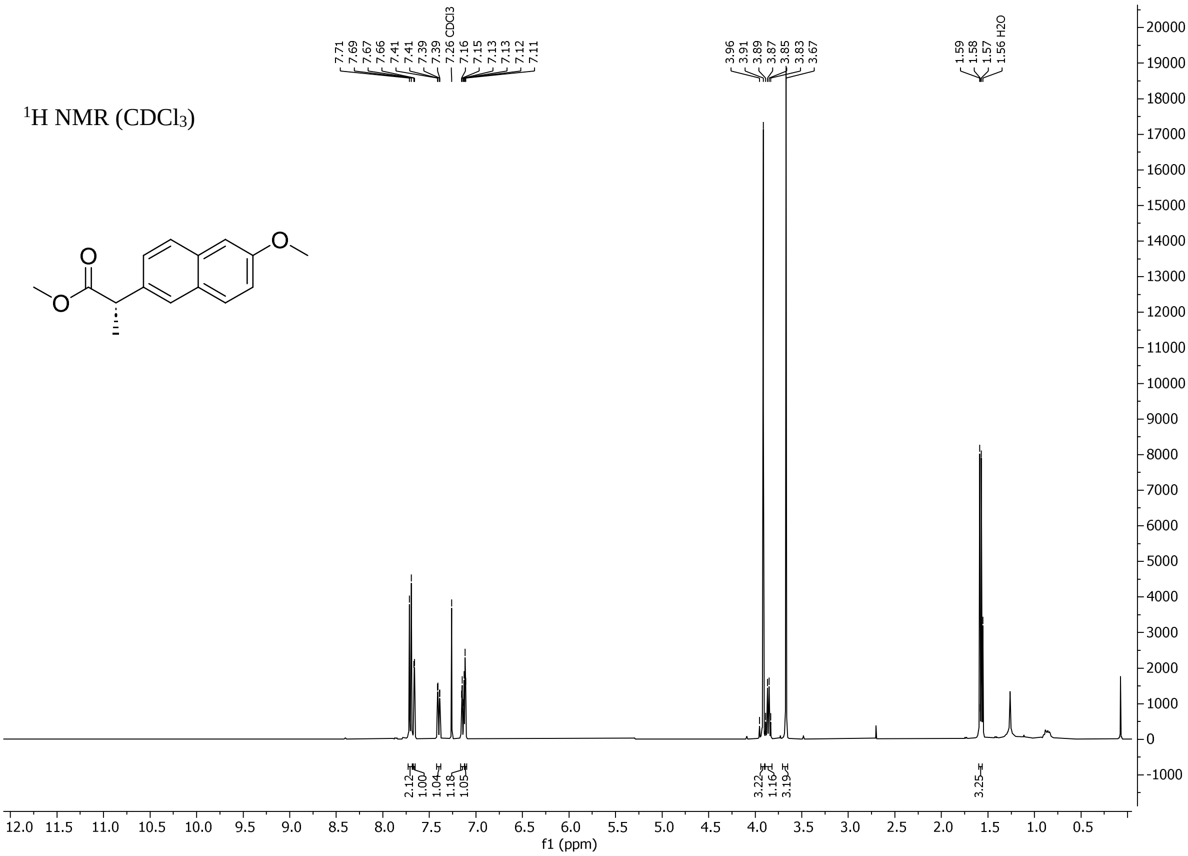

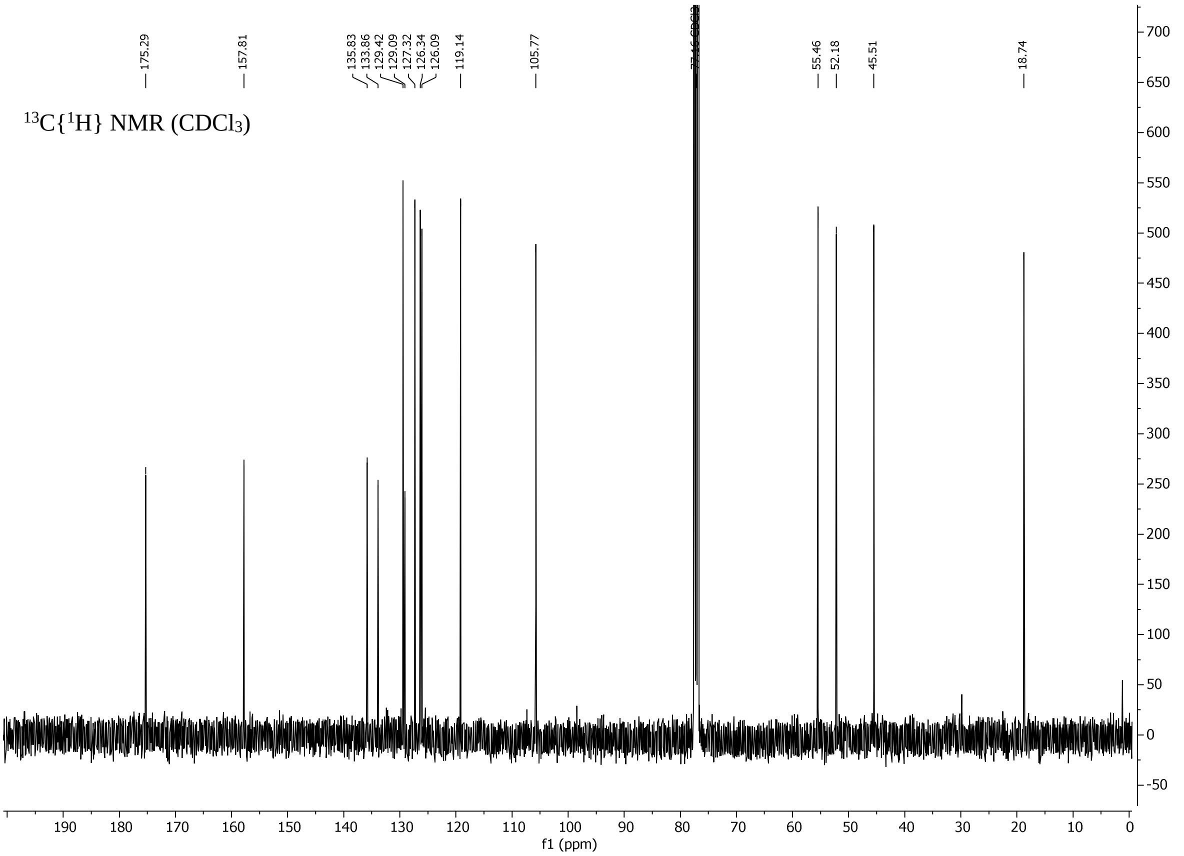

10.10 Compound 24

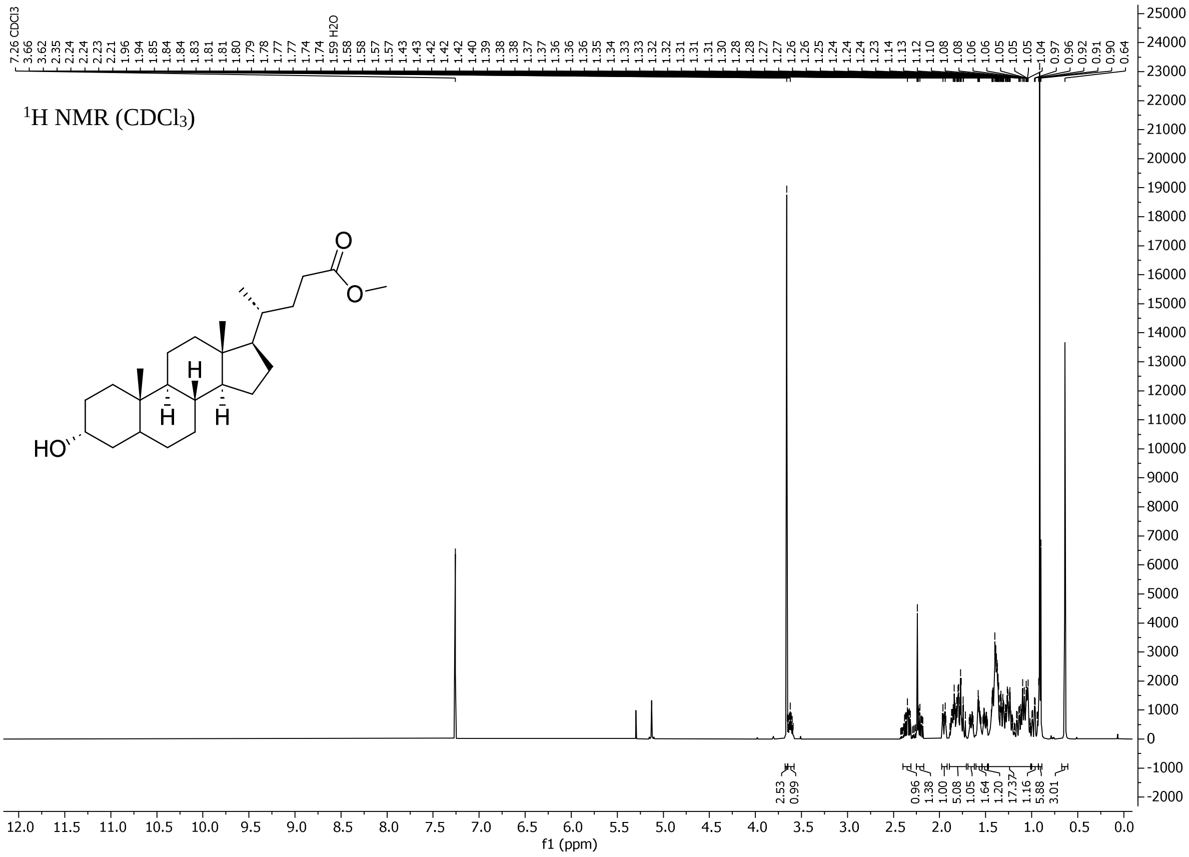

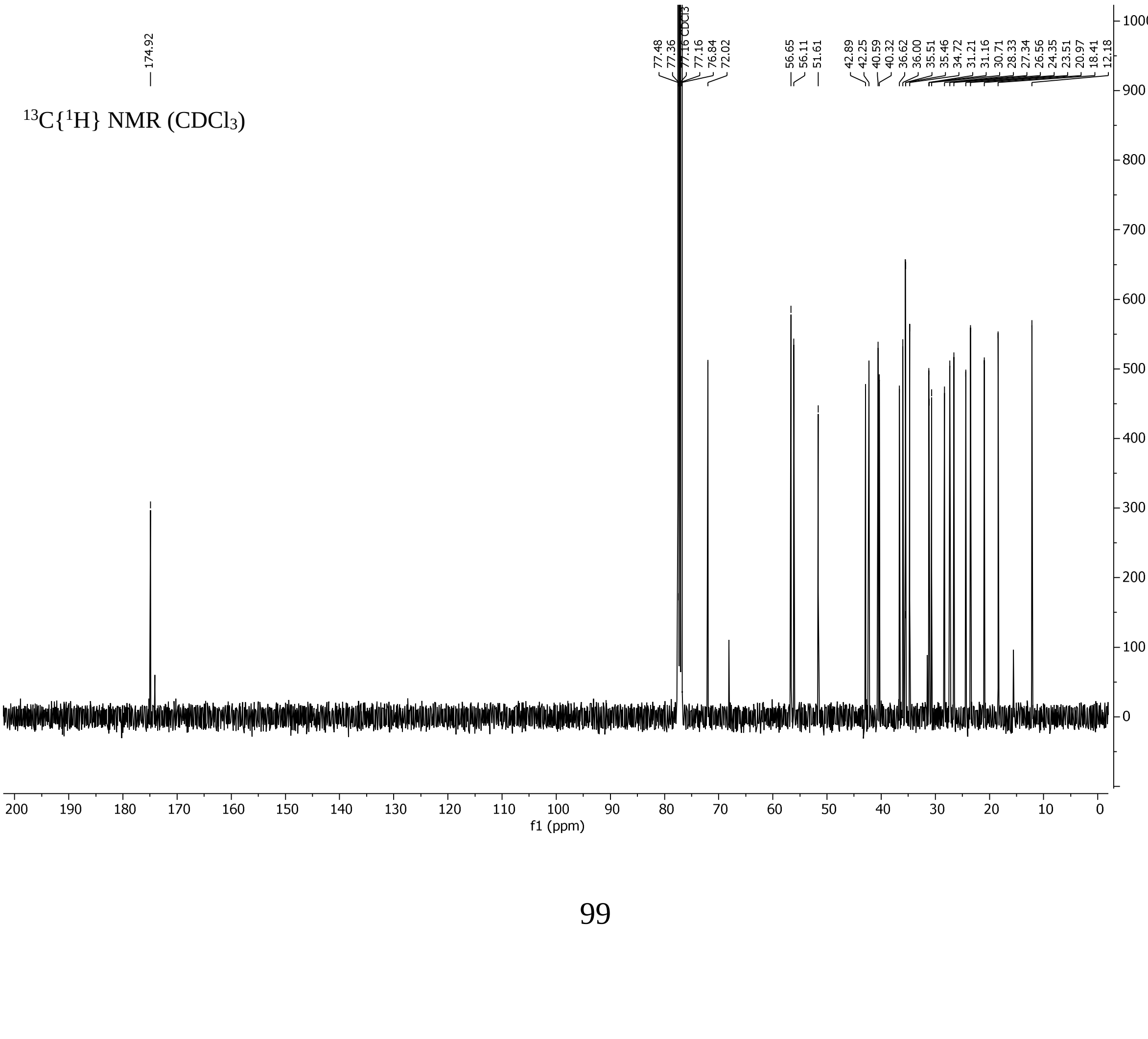

10.11 Compound 25

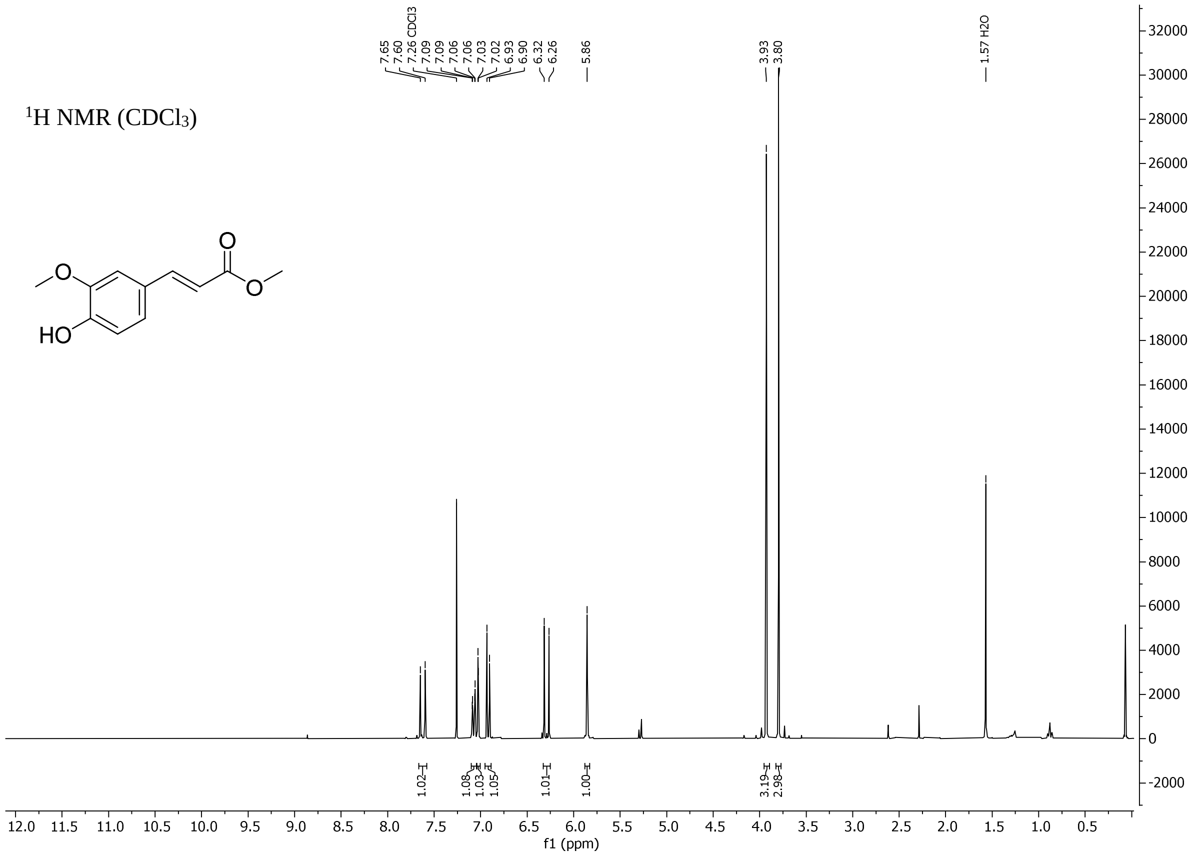

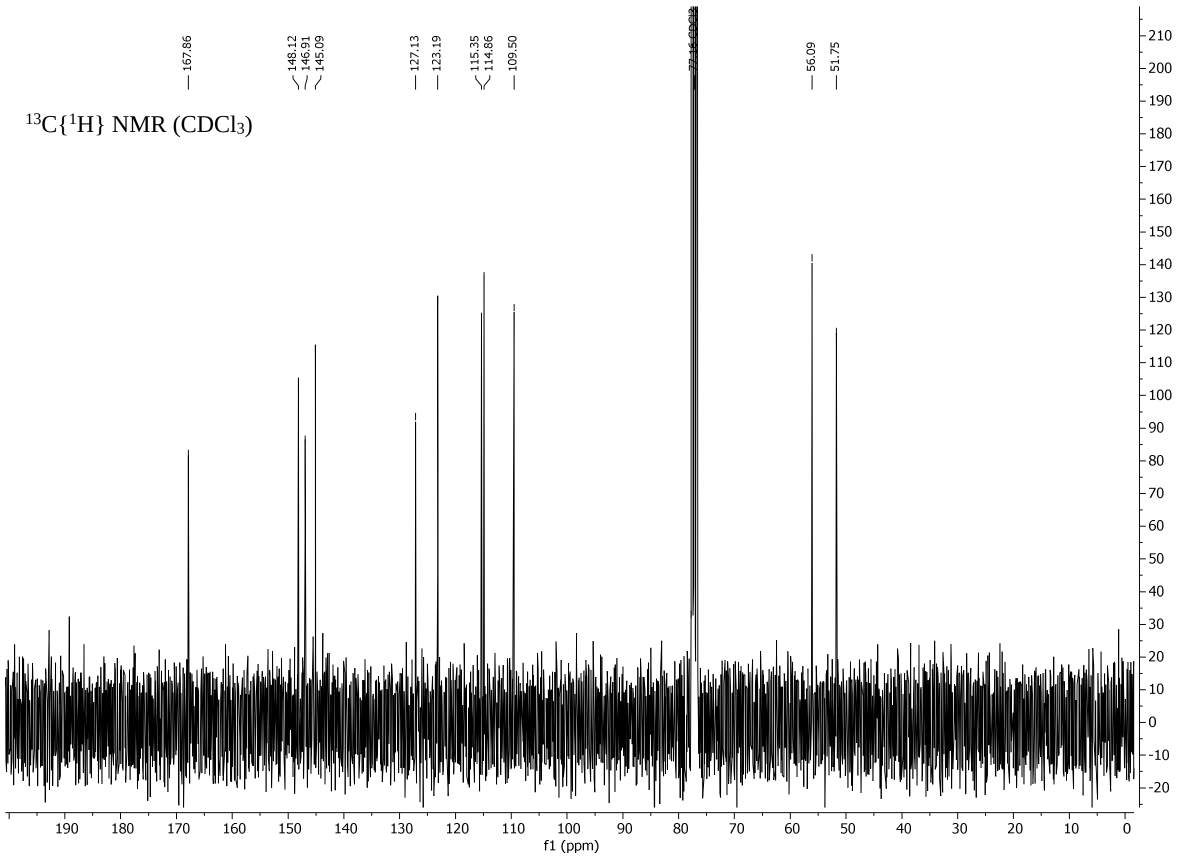

10.12 Compound SI-9

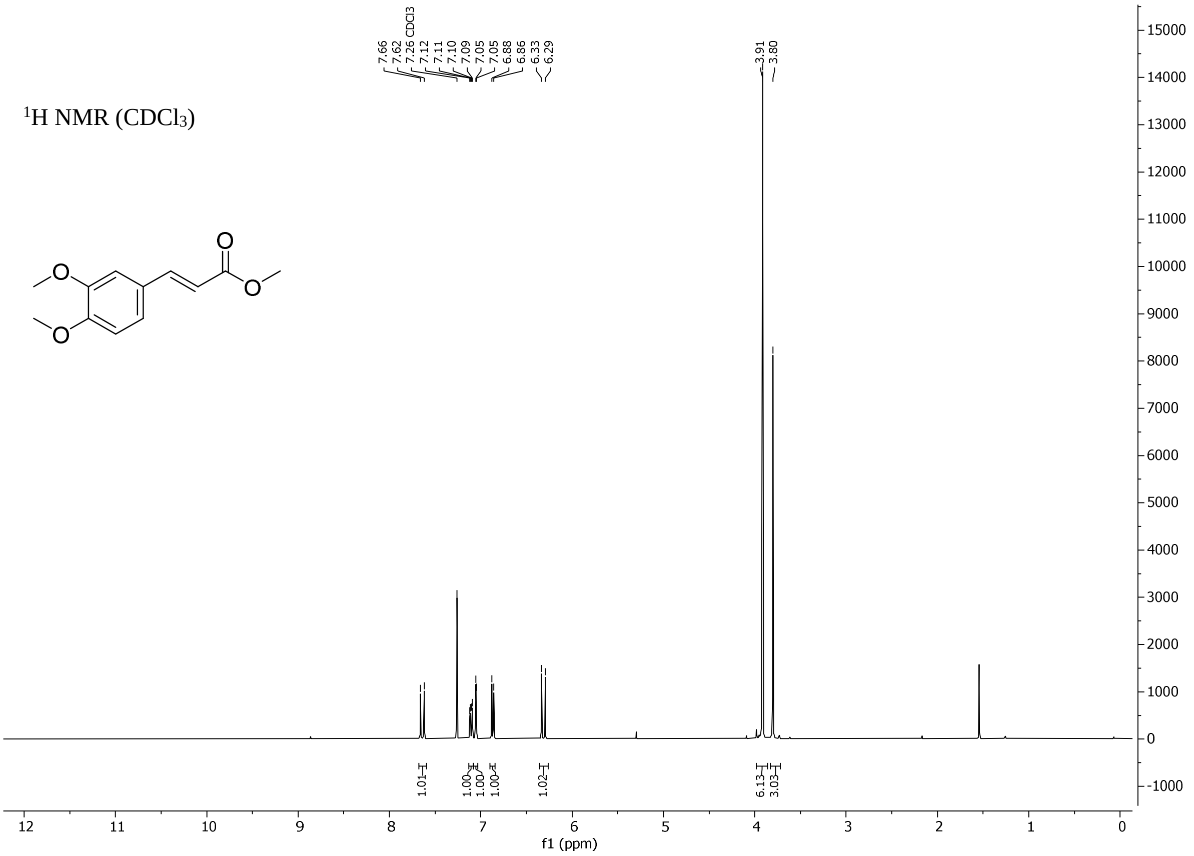

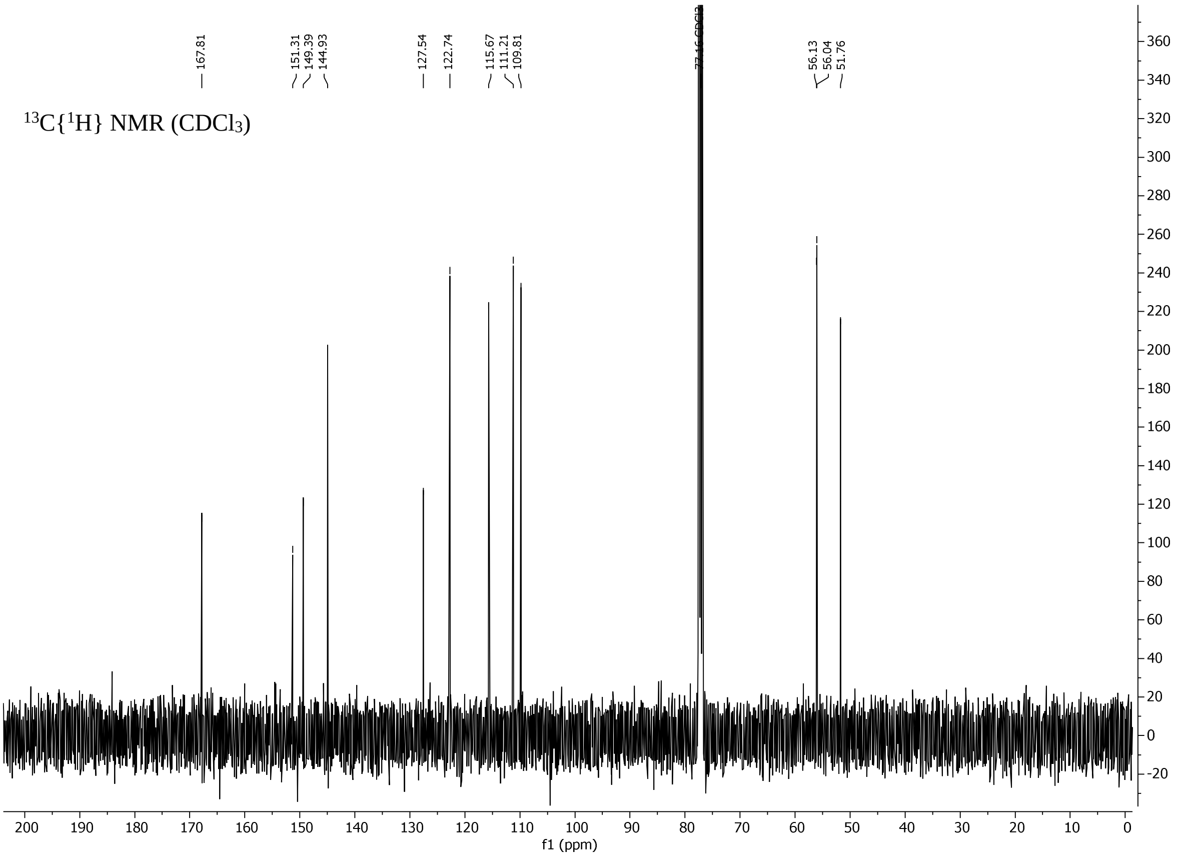

10.13 Compound 26

^{1}H NMR ($CDCl_3$)

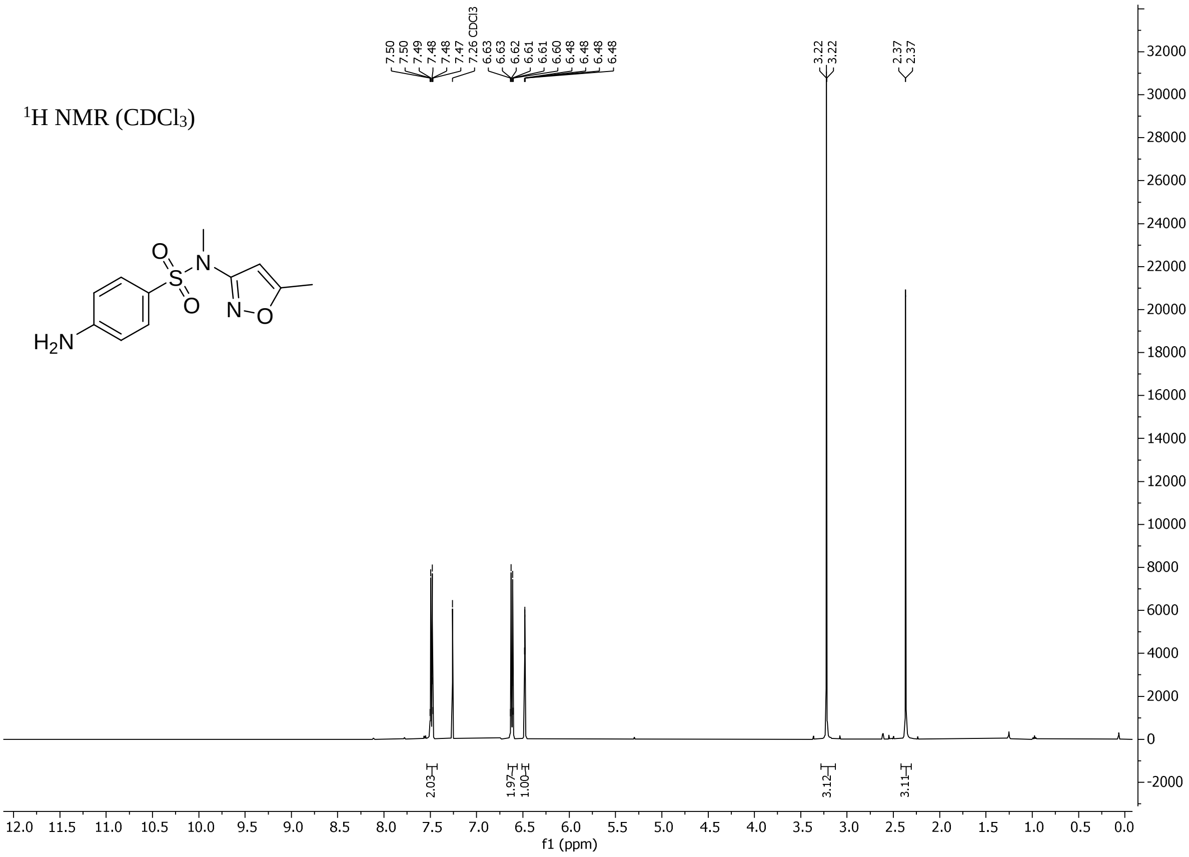

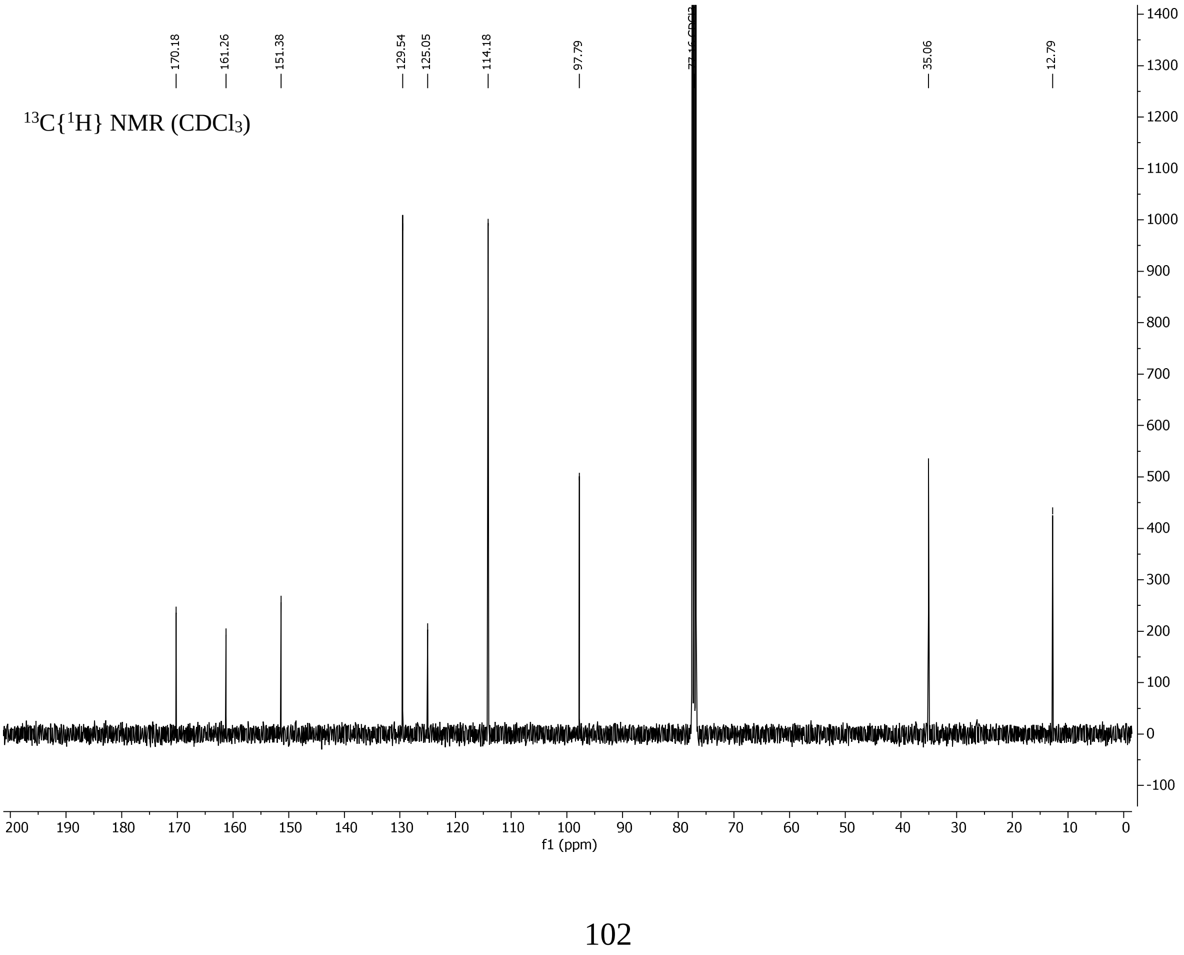

$^{13}C\{^{1}H\}$ NMR ($CDCl_3$)

10.14 Compound 27

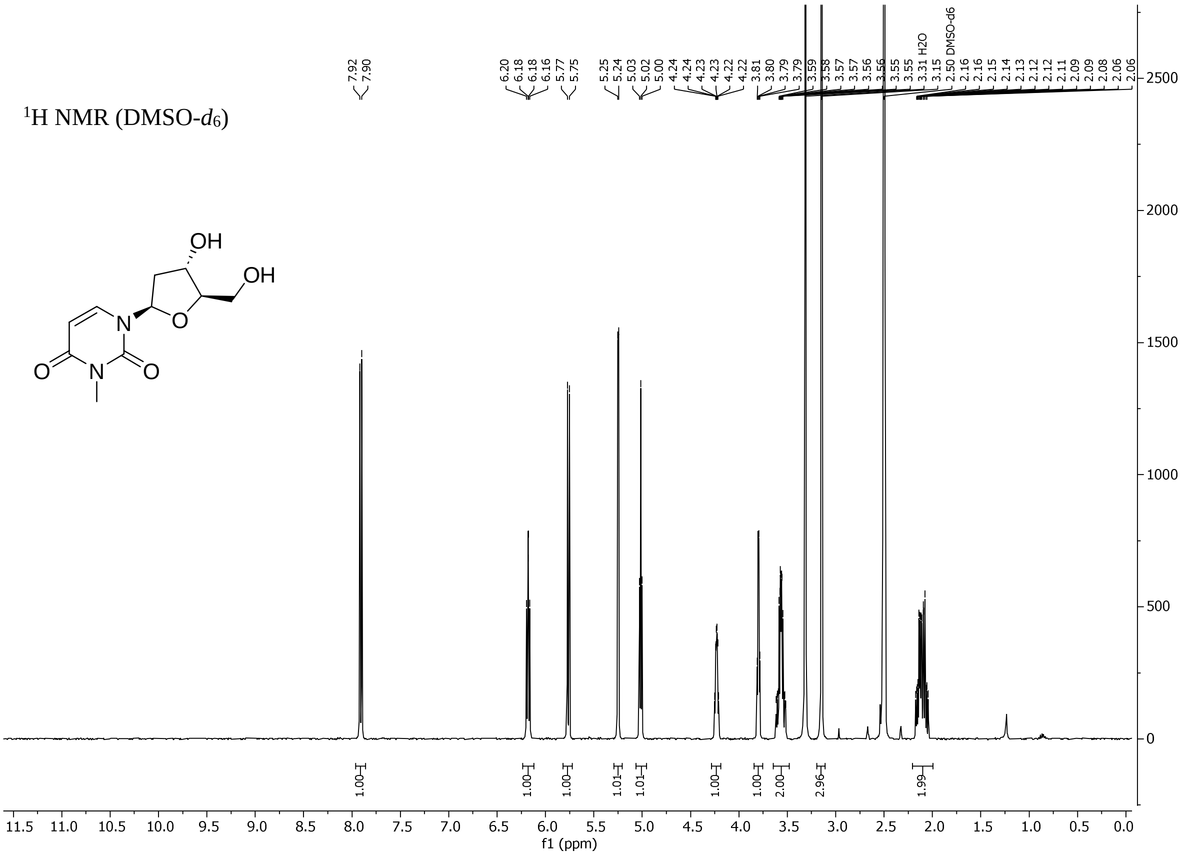

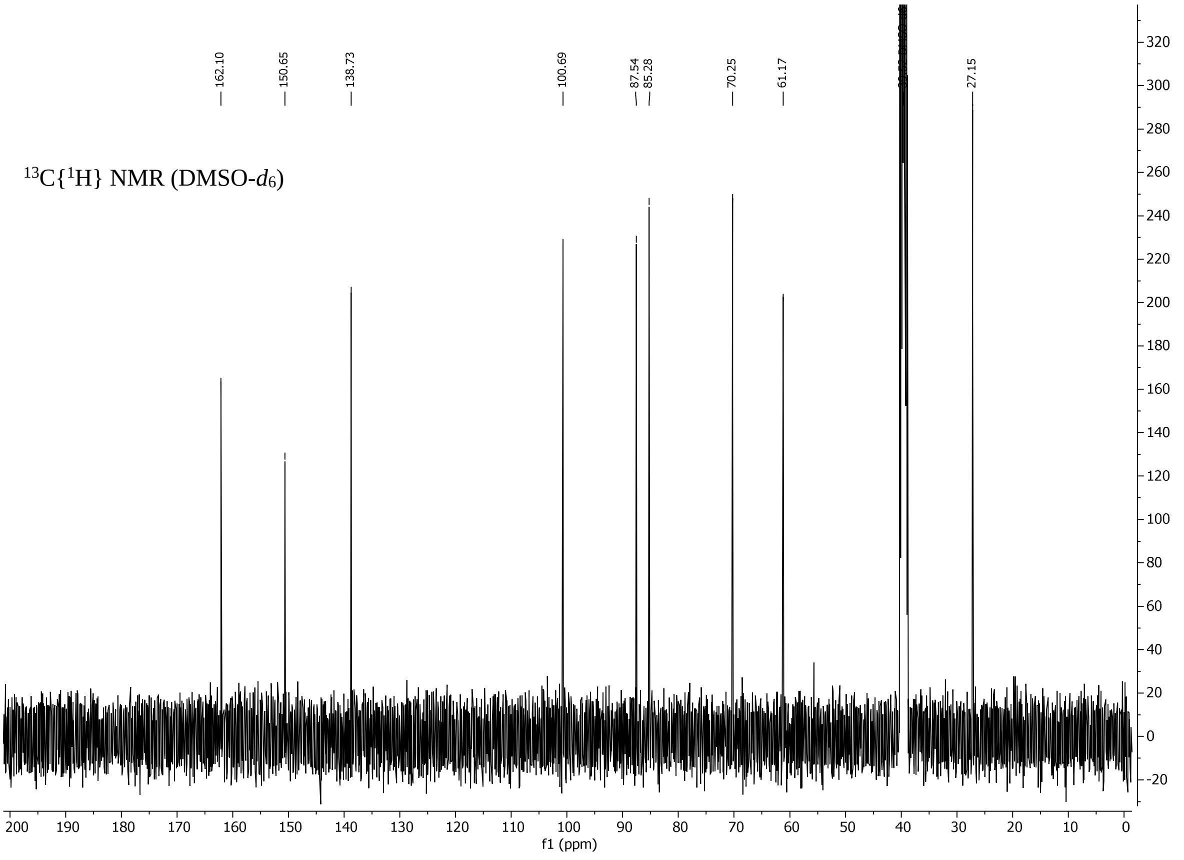

10.15 Compound 28

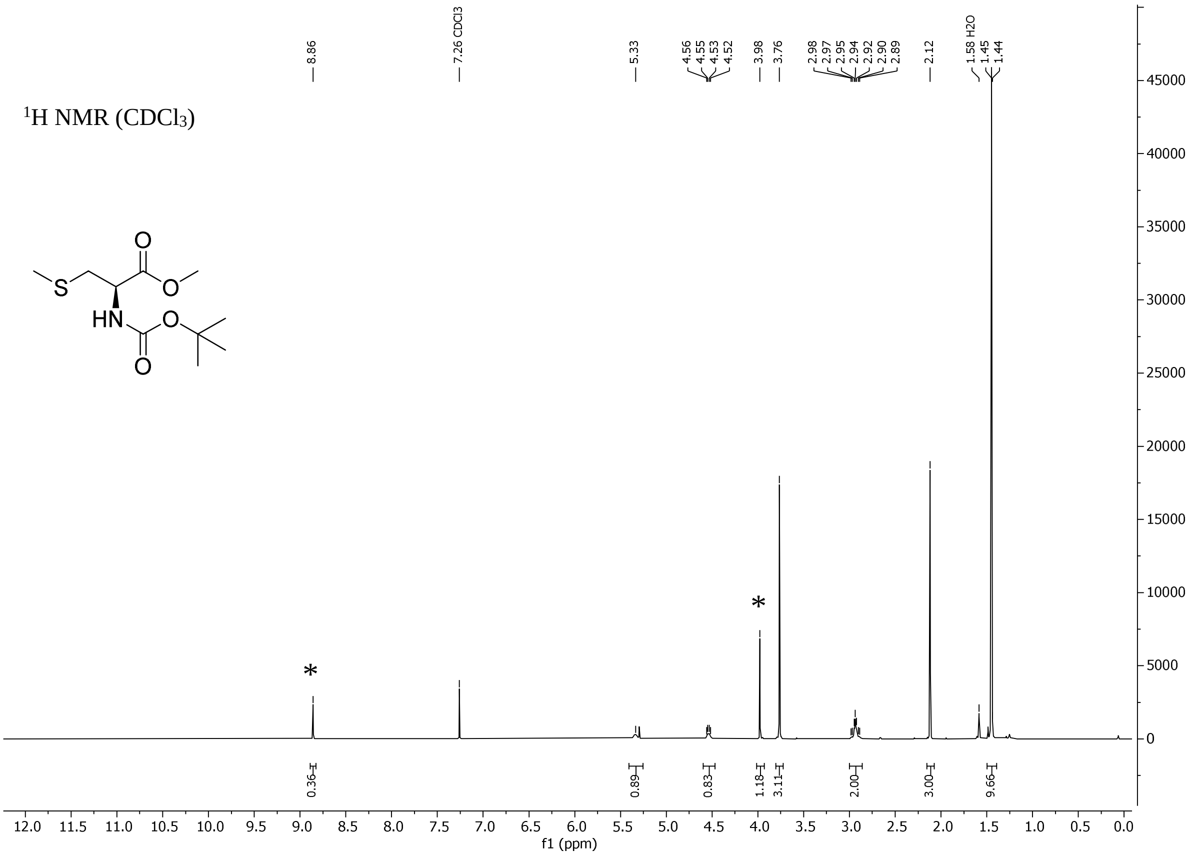

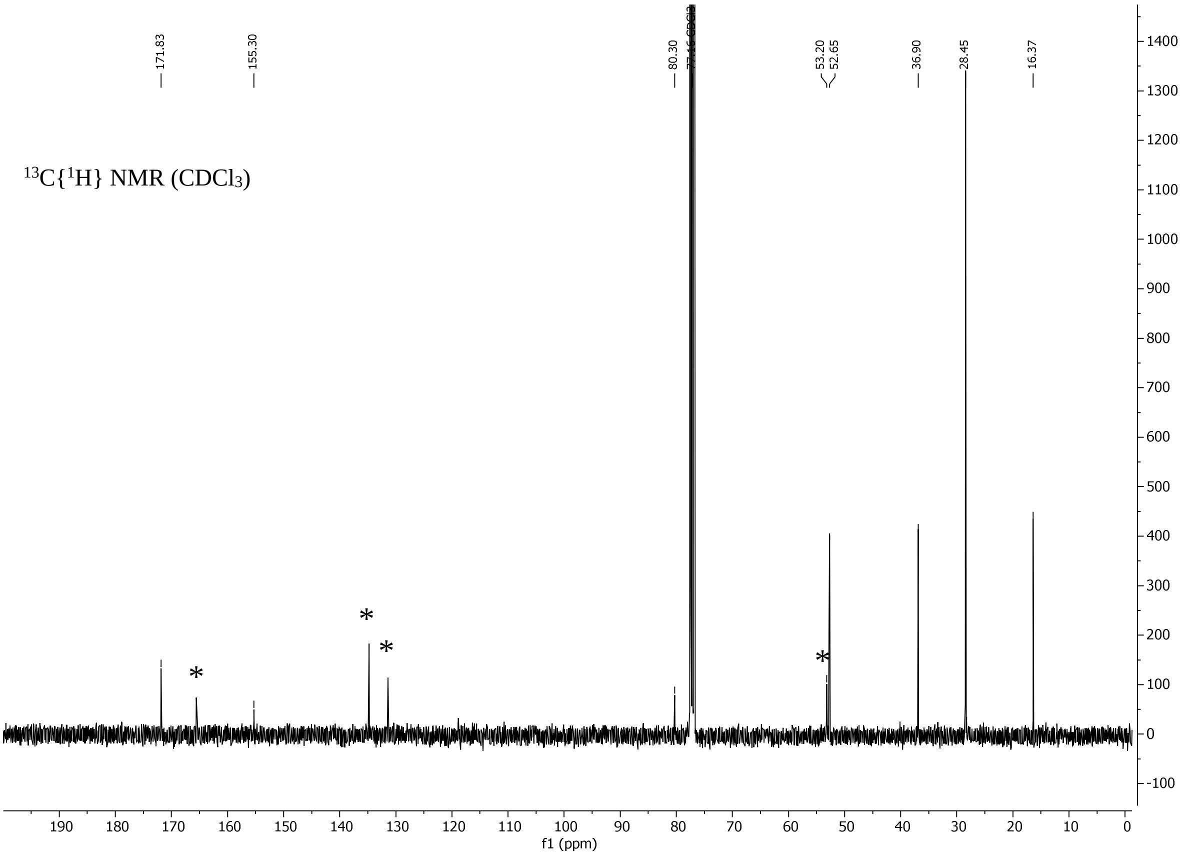

* indicates internal standard

10.16 Compound 29

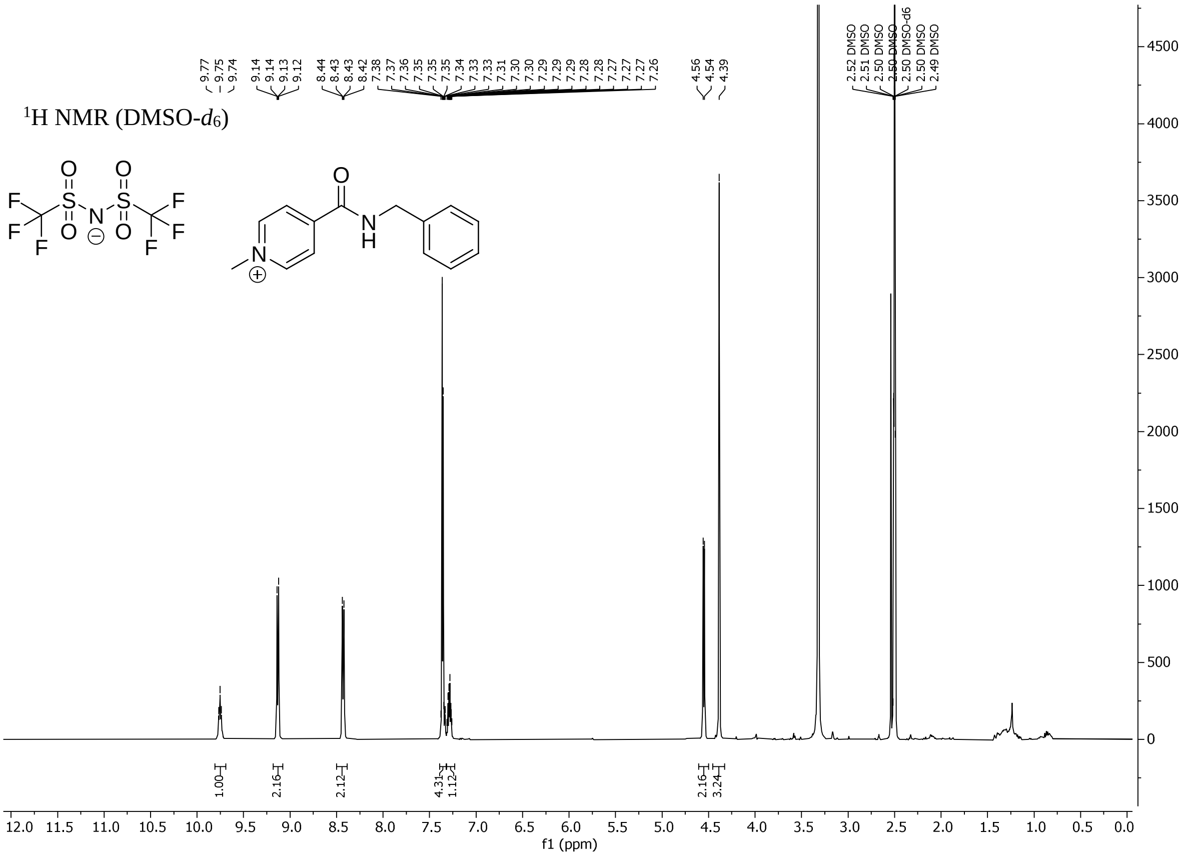

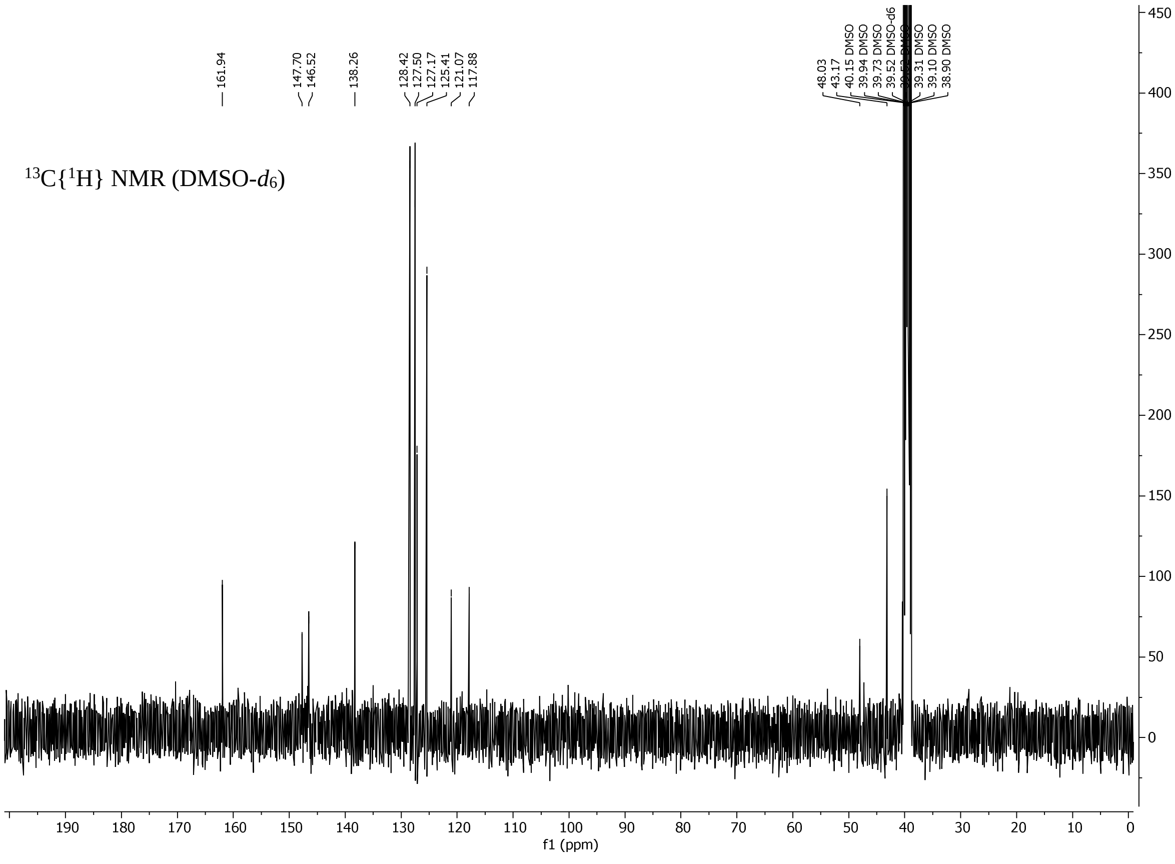

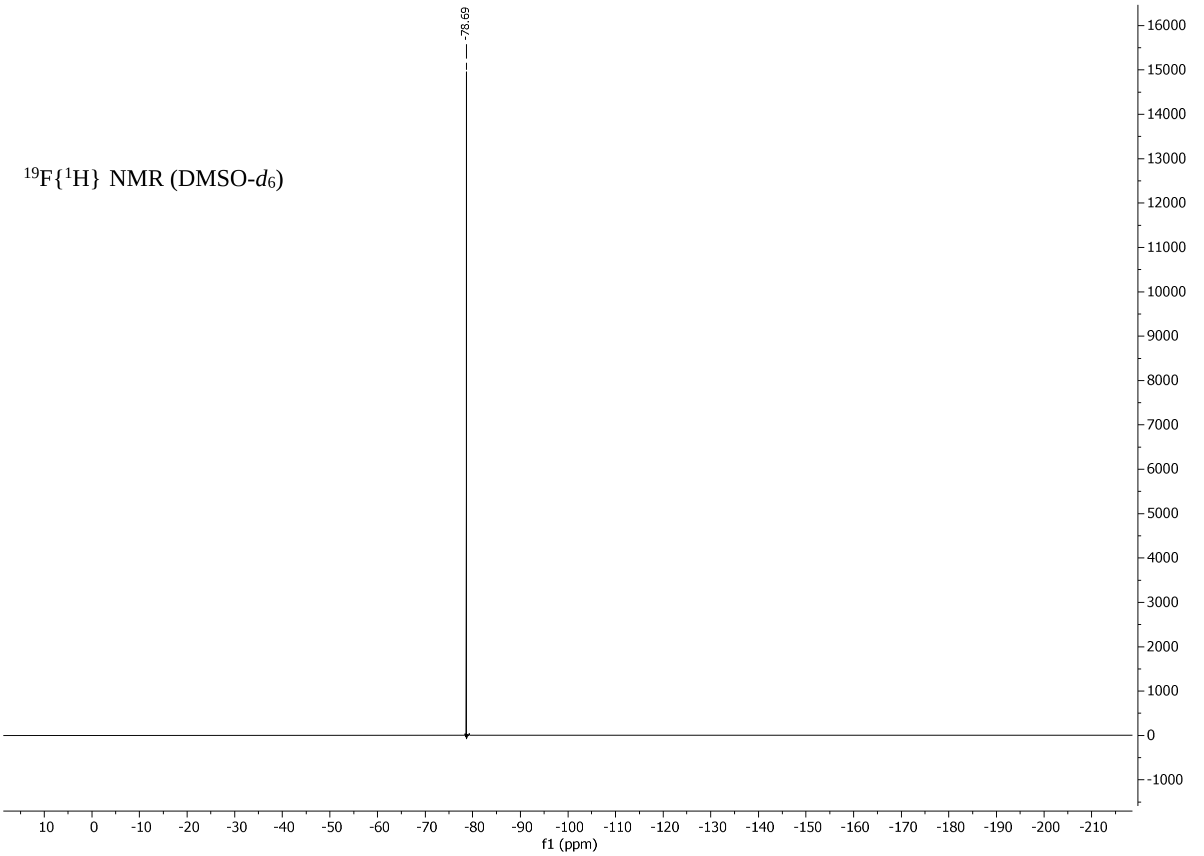

$^{19}F\{^{1}H\}$ NMR (DMSO-d_6)

10.17 Compound 30

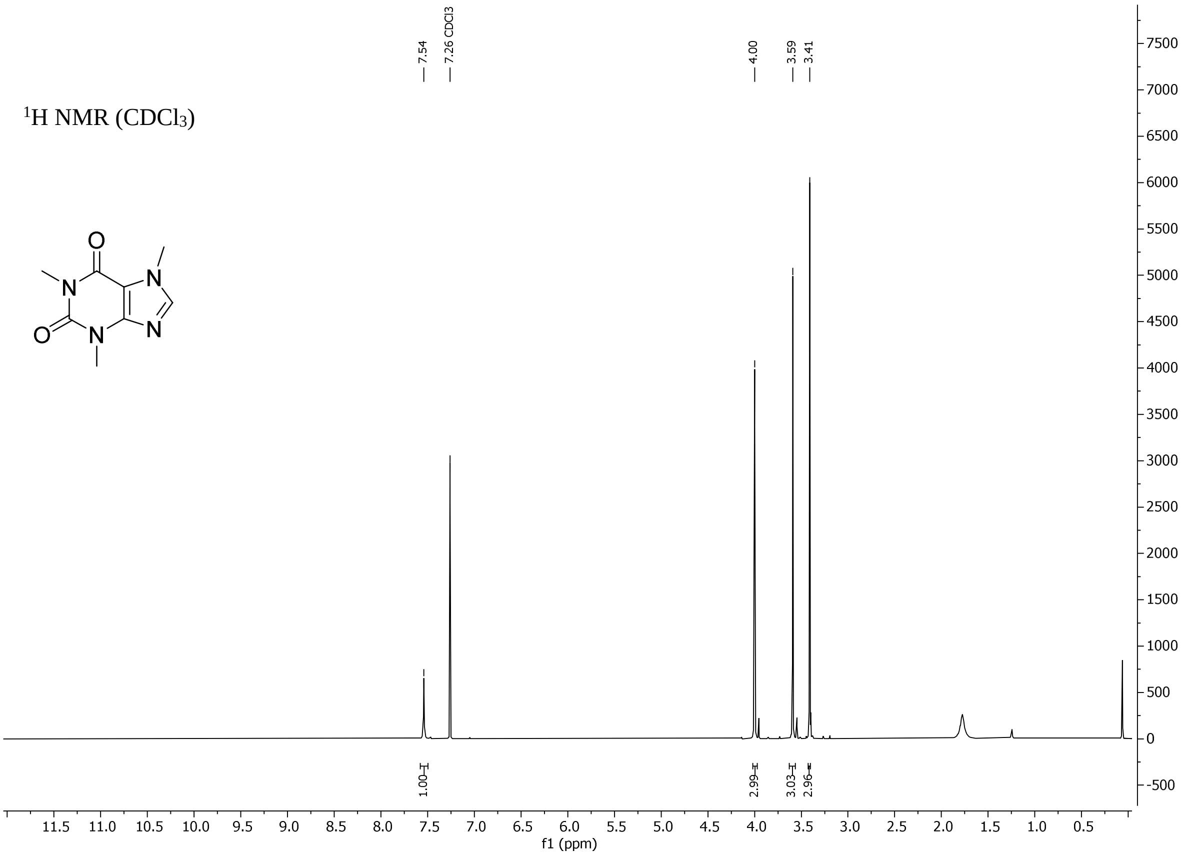

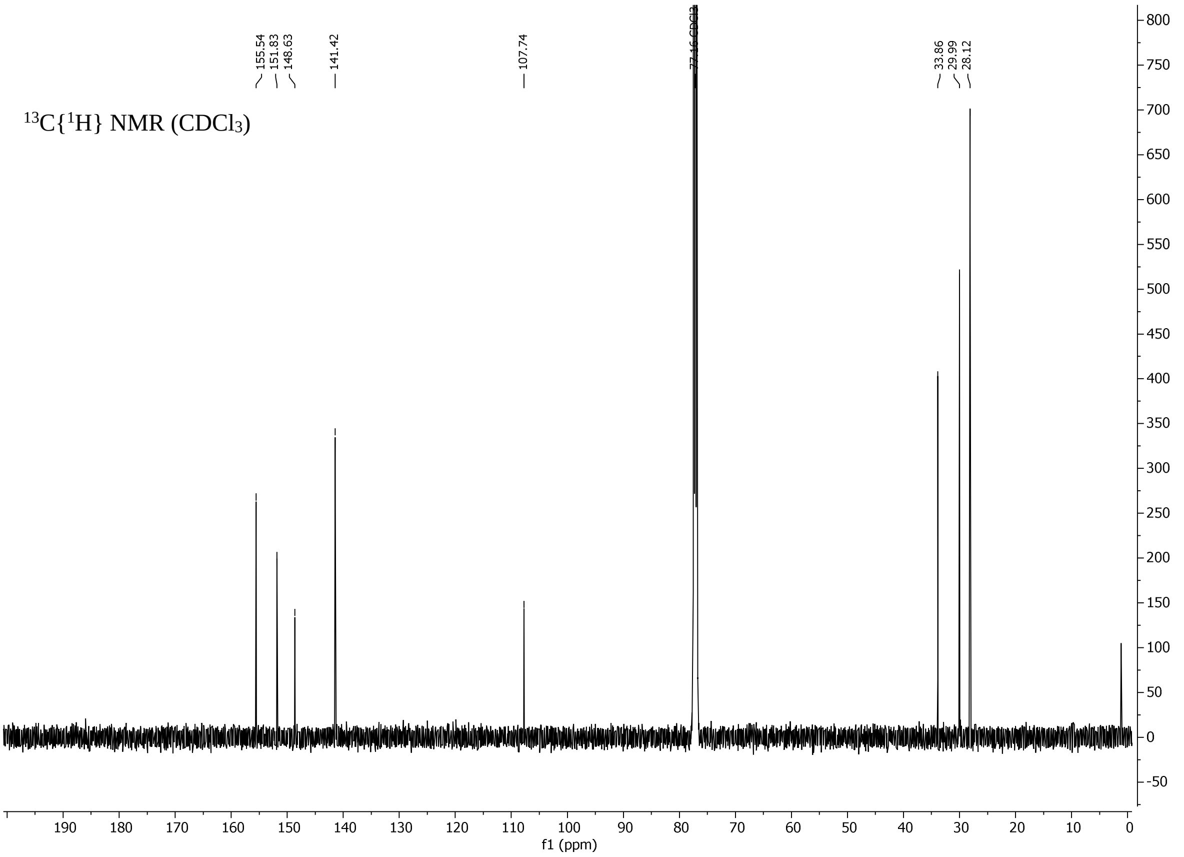

10.18 Compound 31

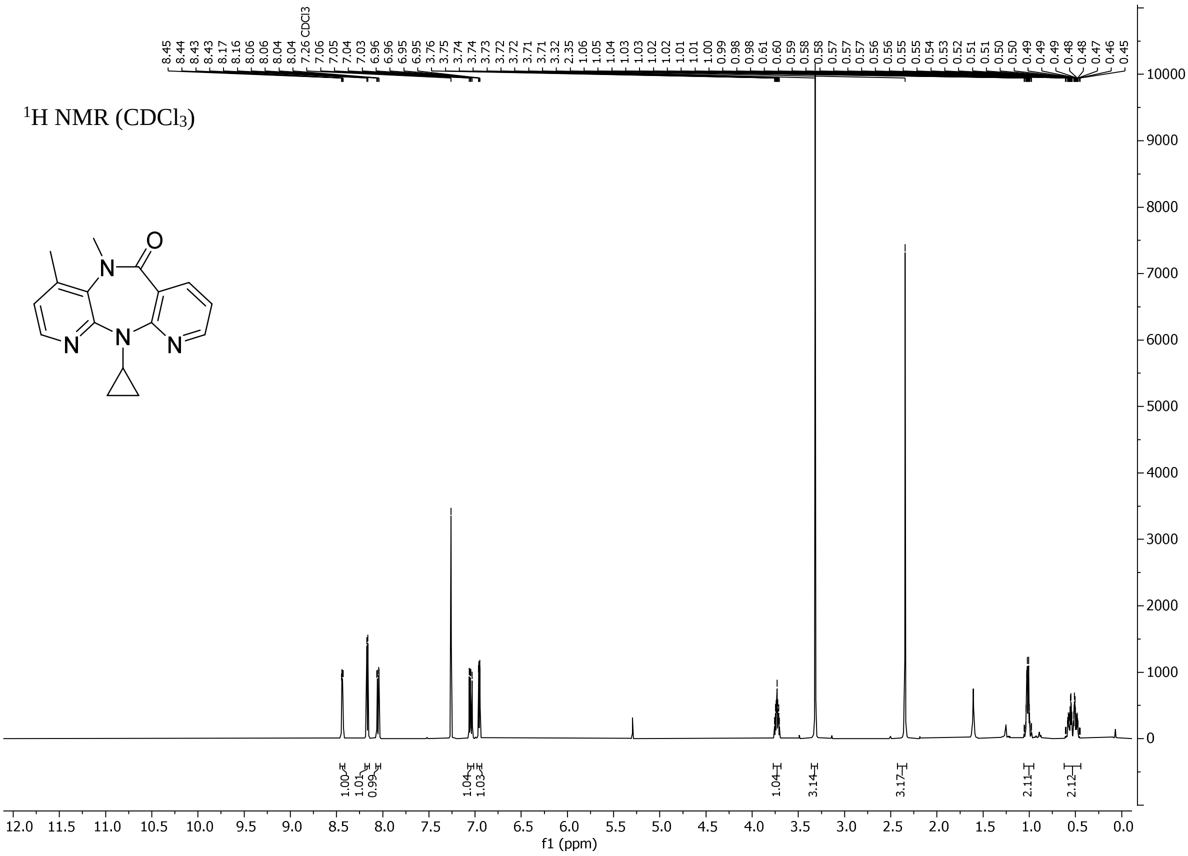

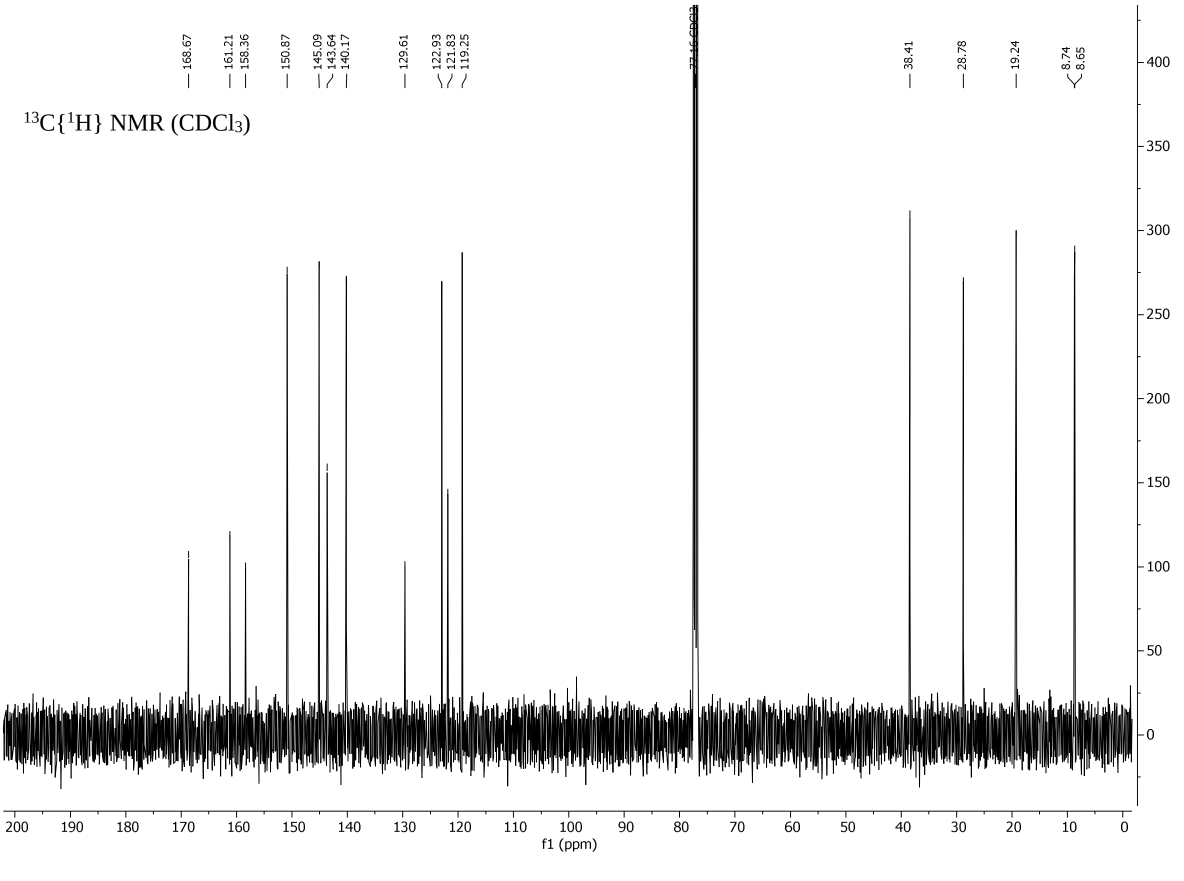

10.19 Compound SI-10

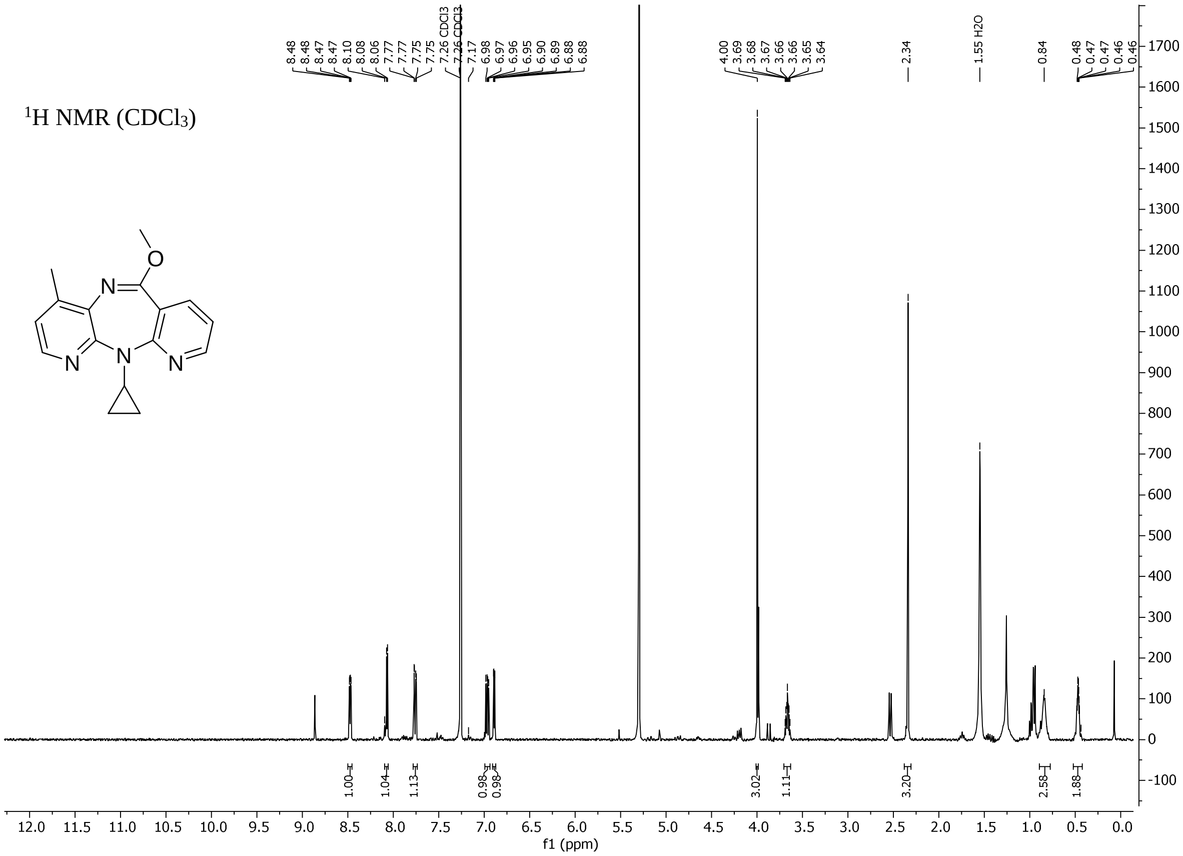

10.20 Compound 32

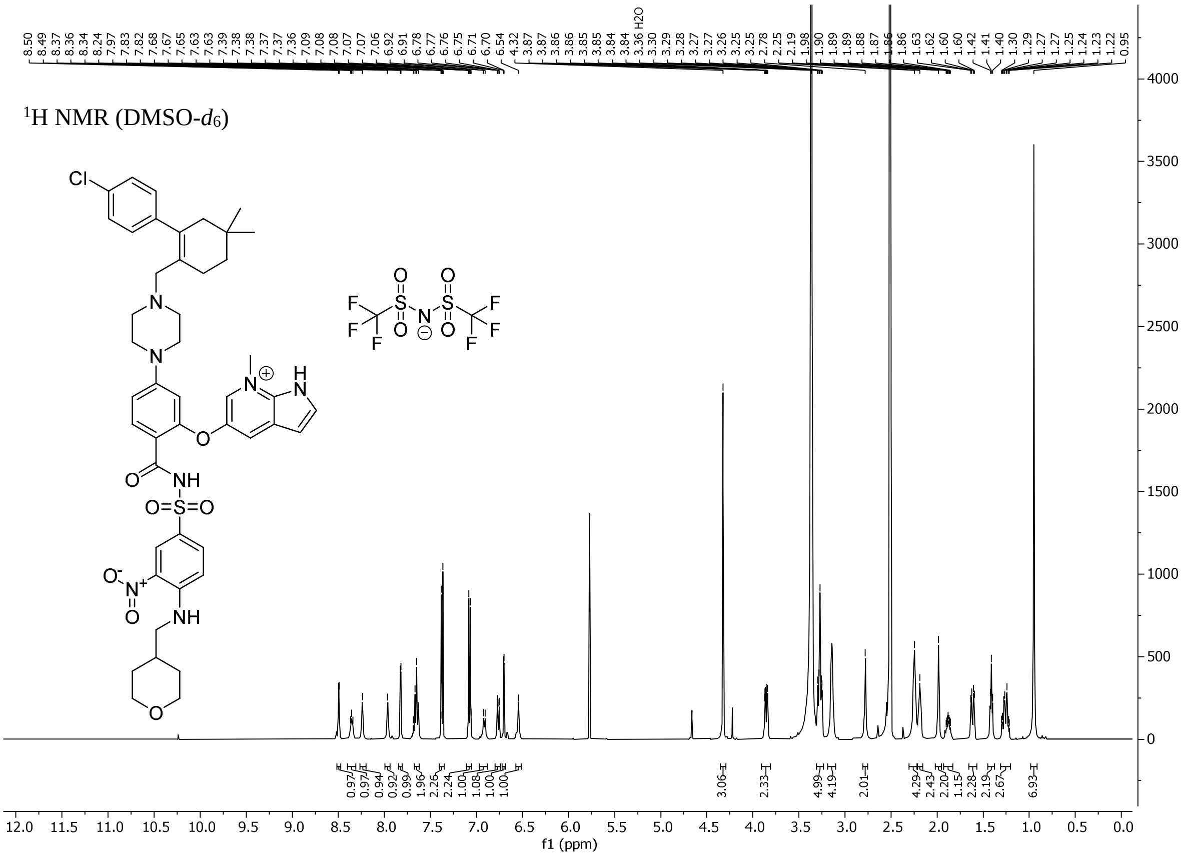

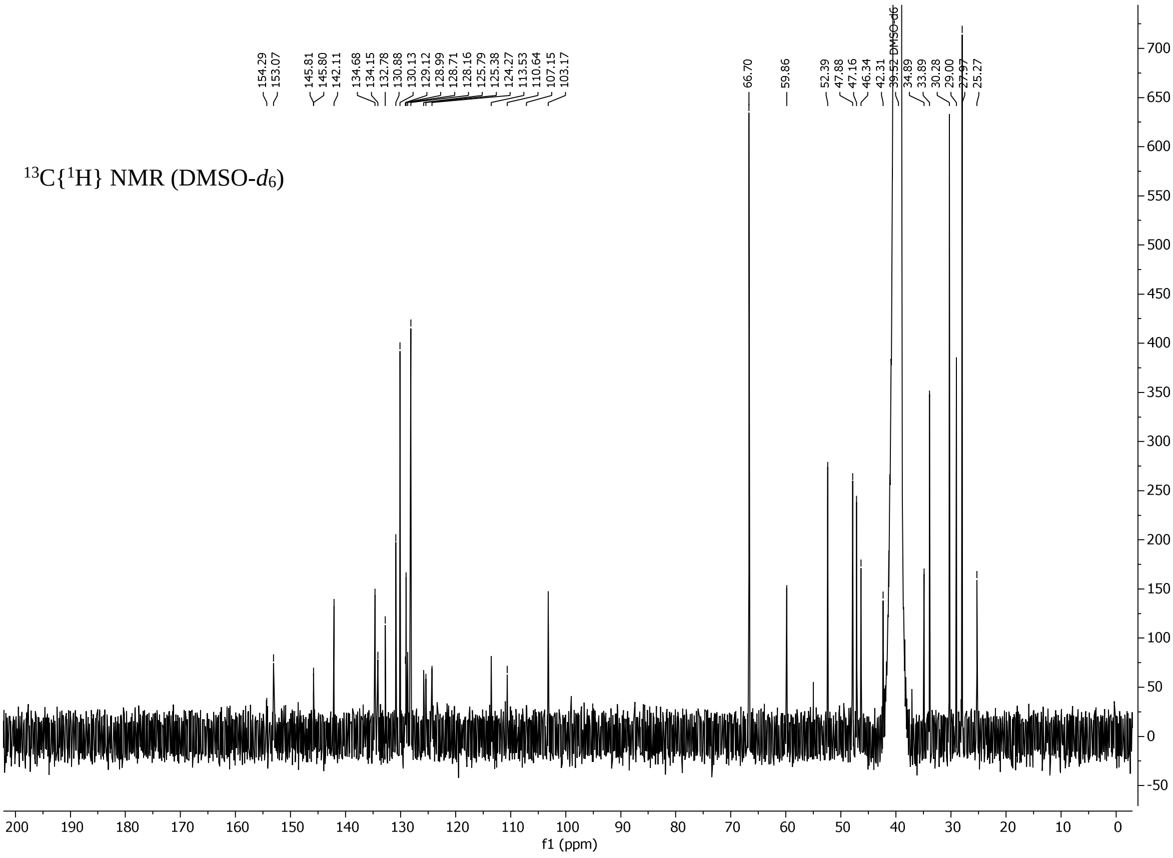

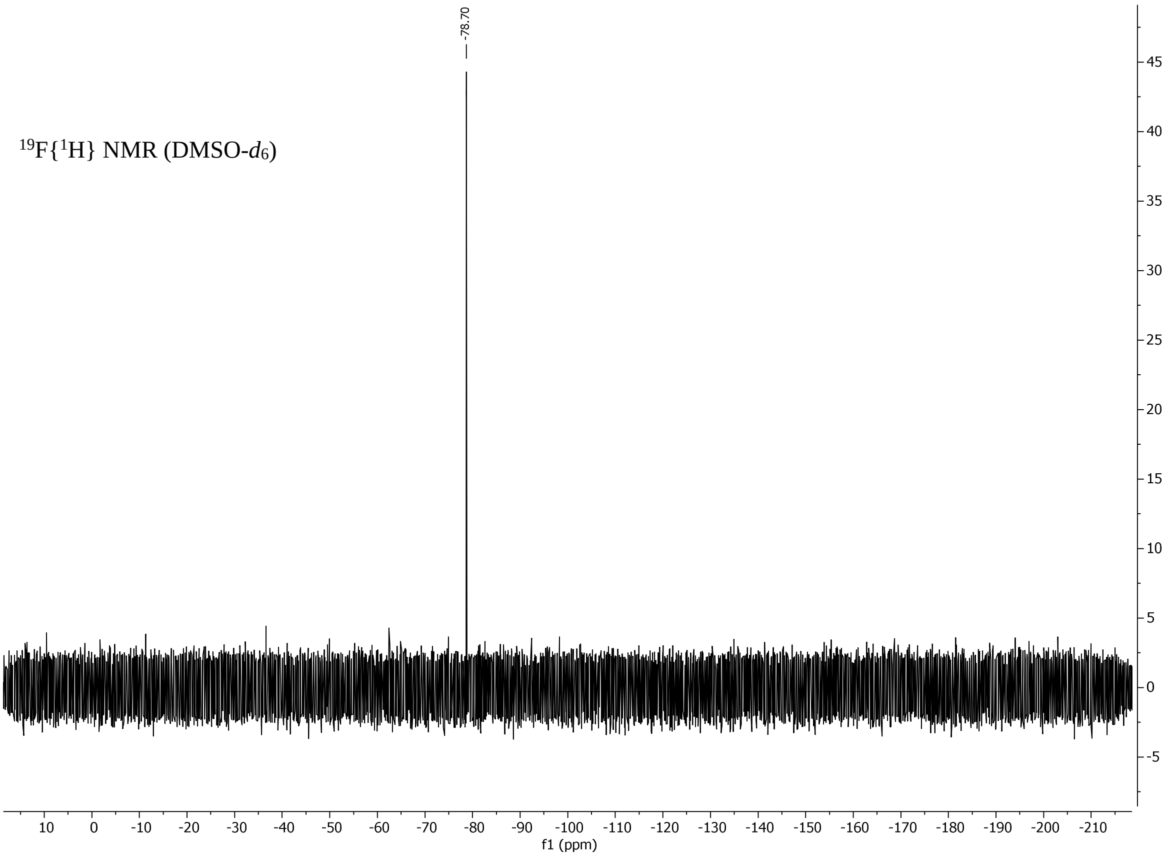
$^{19}F\{^{1}H\}$ NMR (DMSO-d_6)
-78.70
f1 (ppm)

10.21 Compound 35

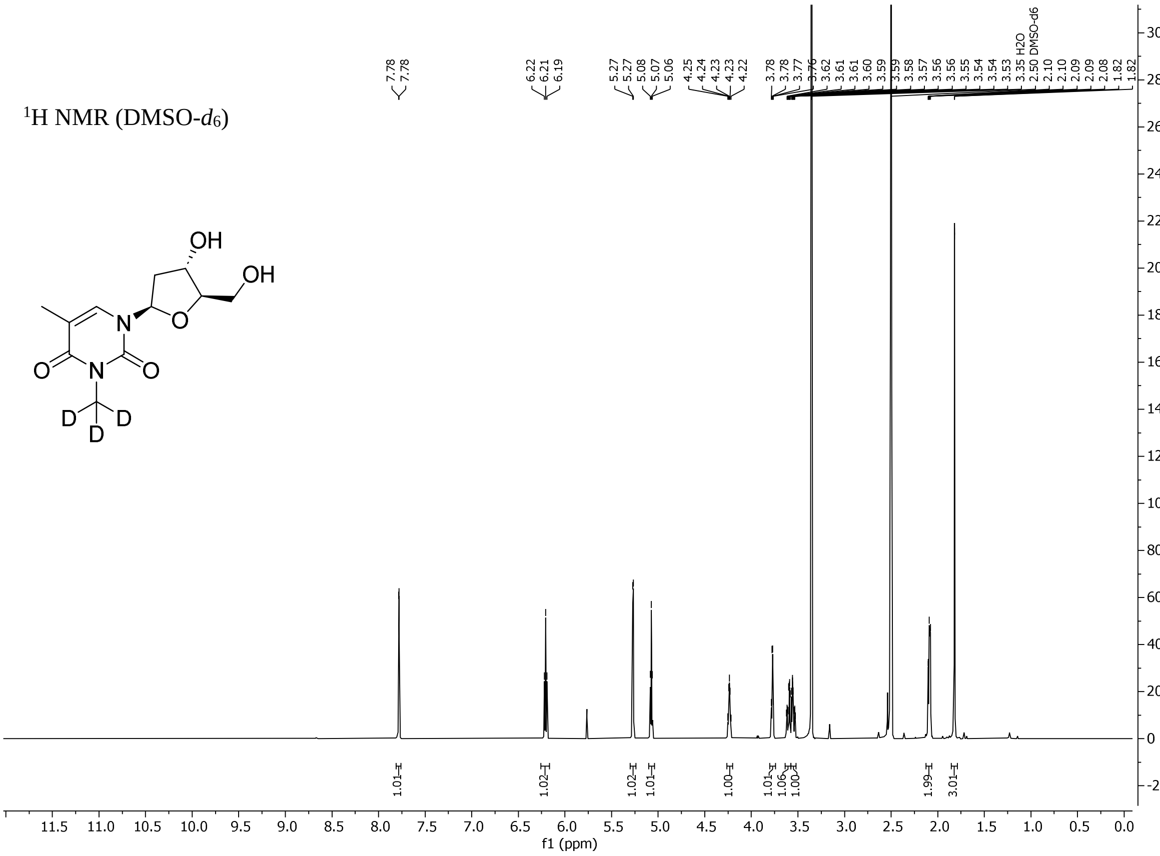

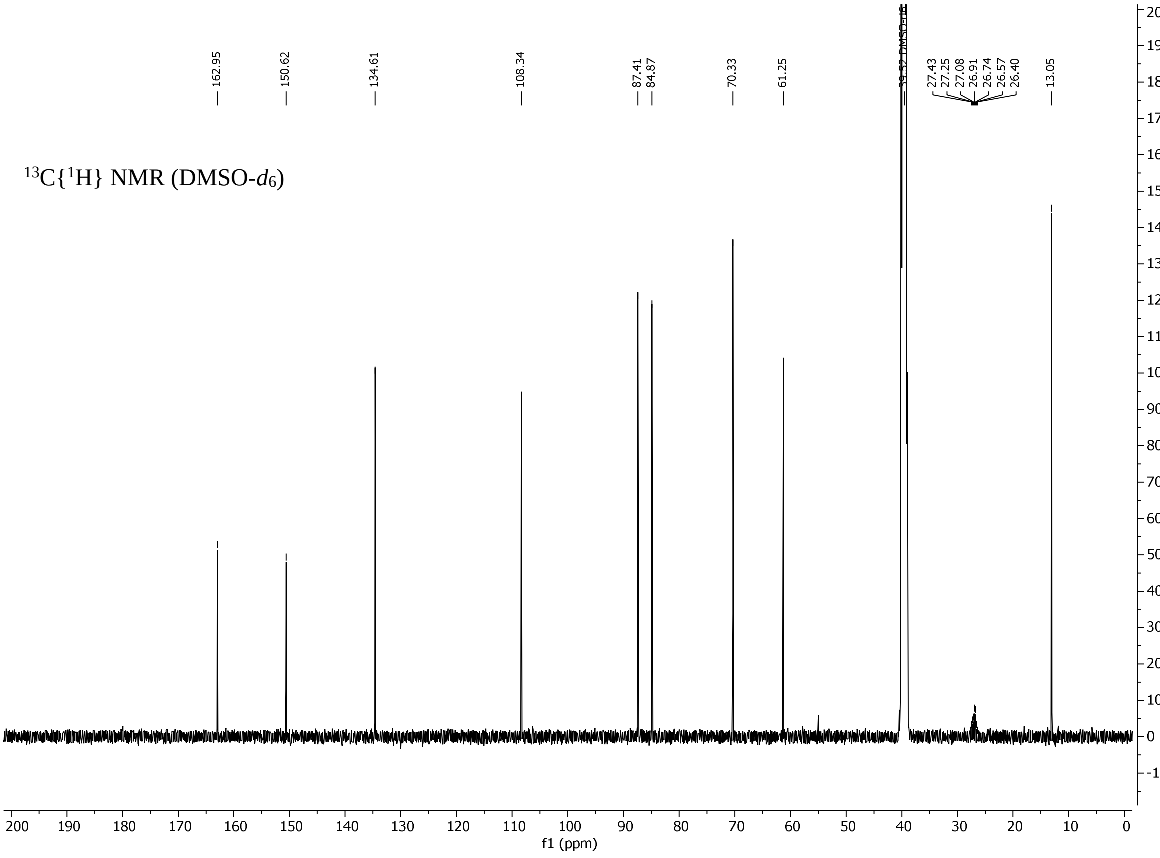

10.22 Compound 36

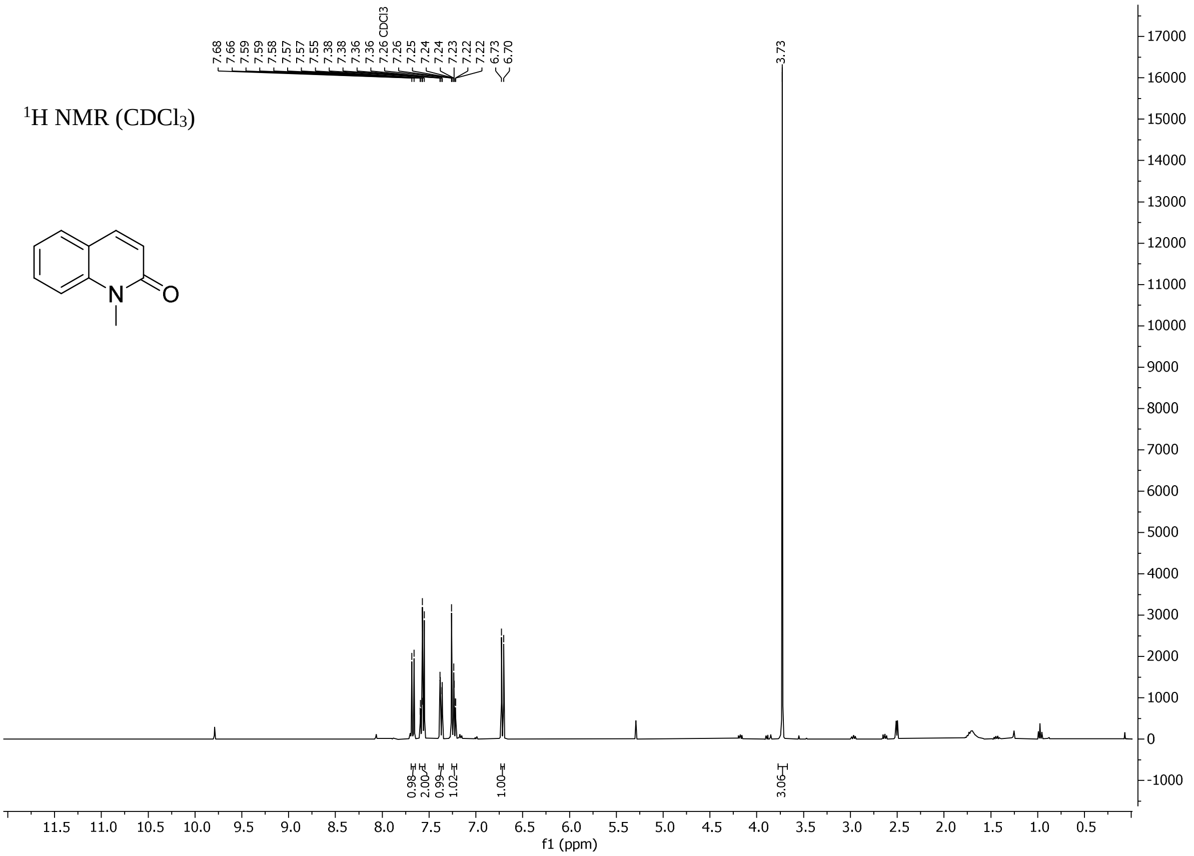

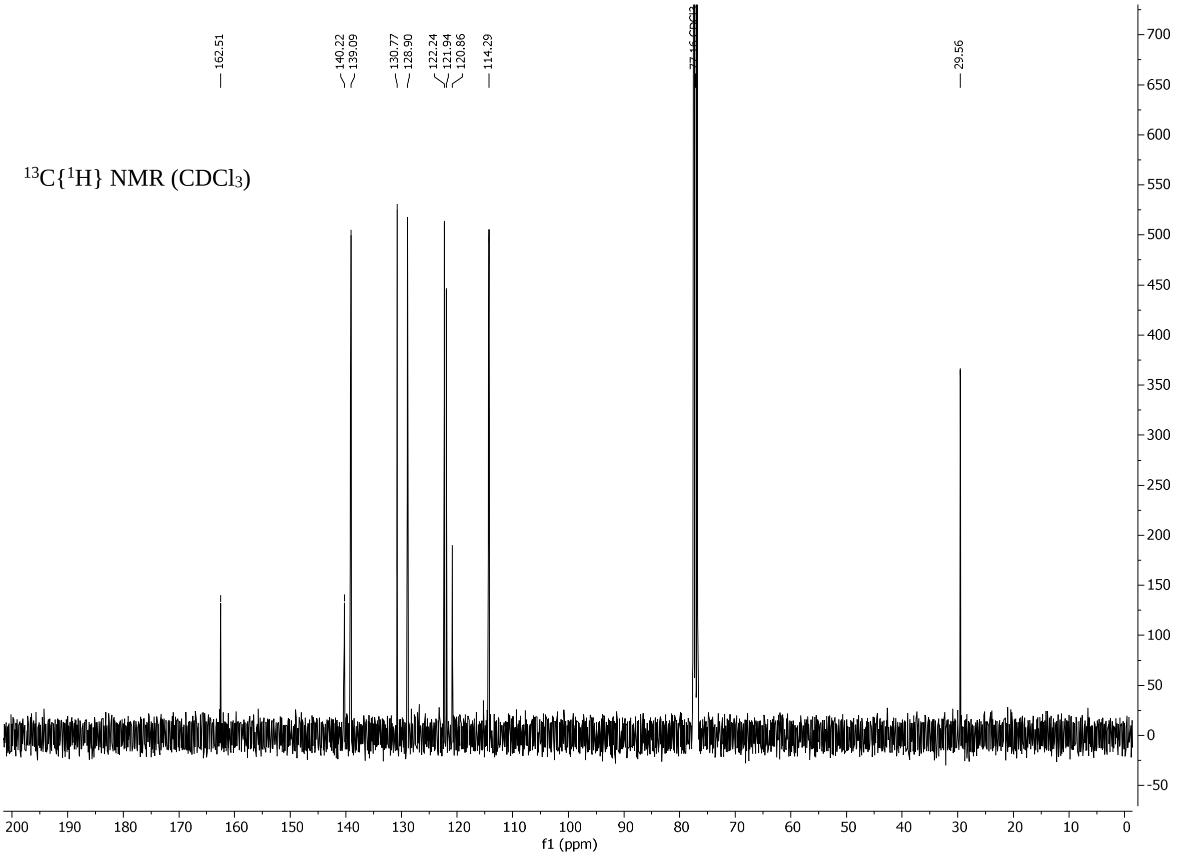

10.23 Compound 38 via flavin methodology

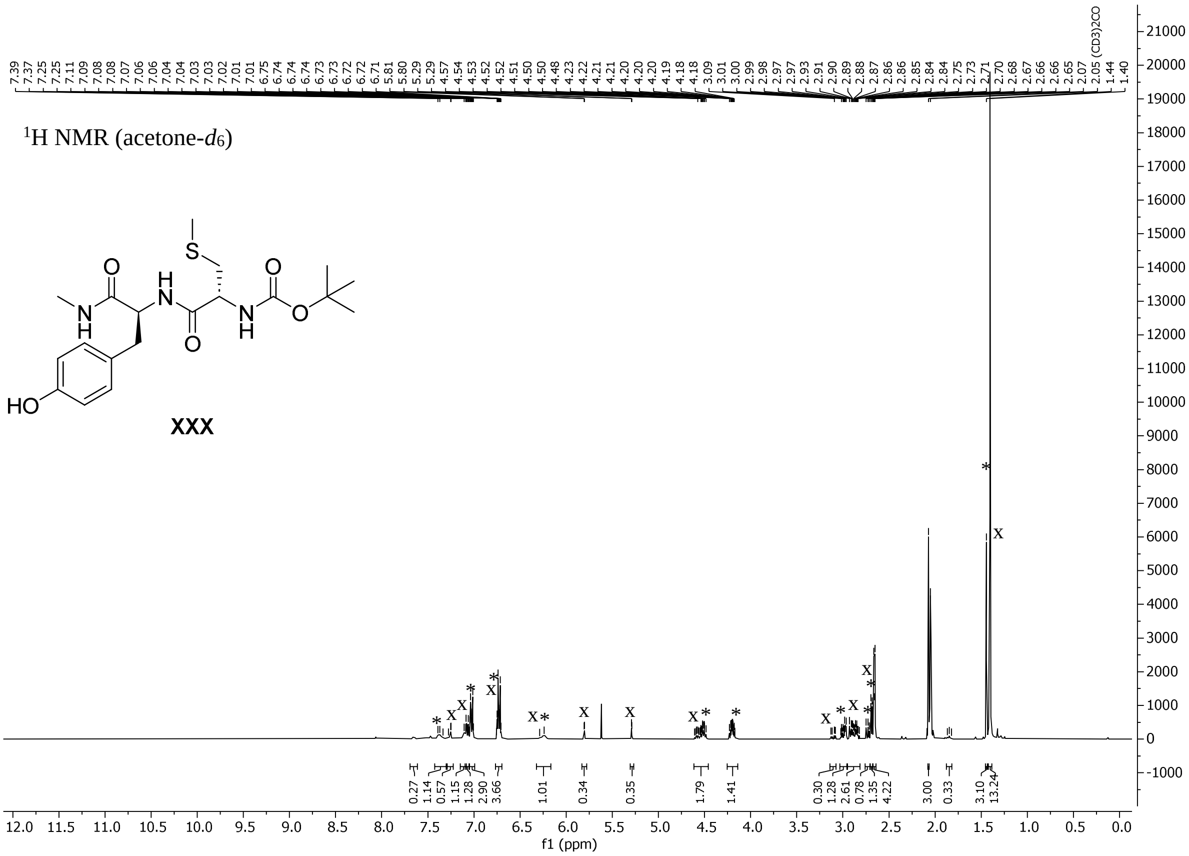

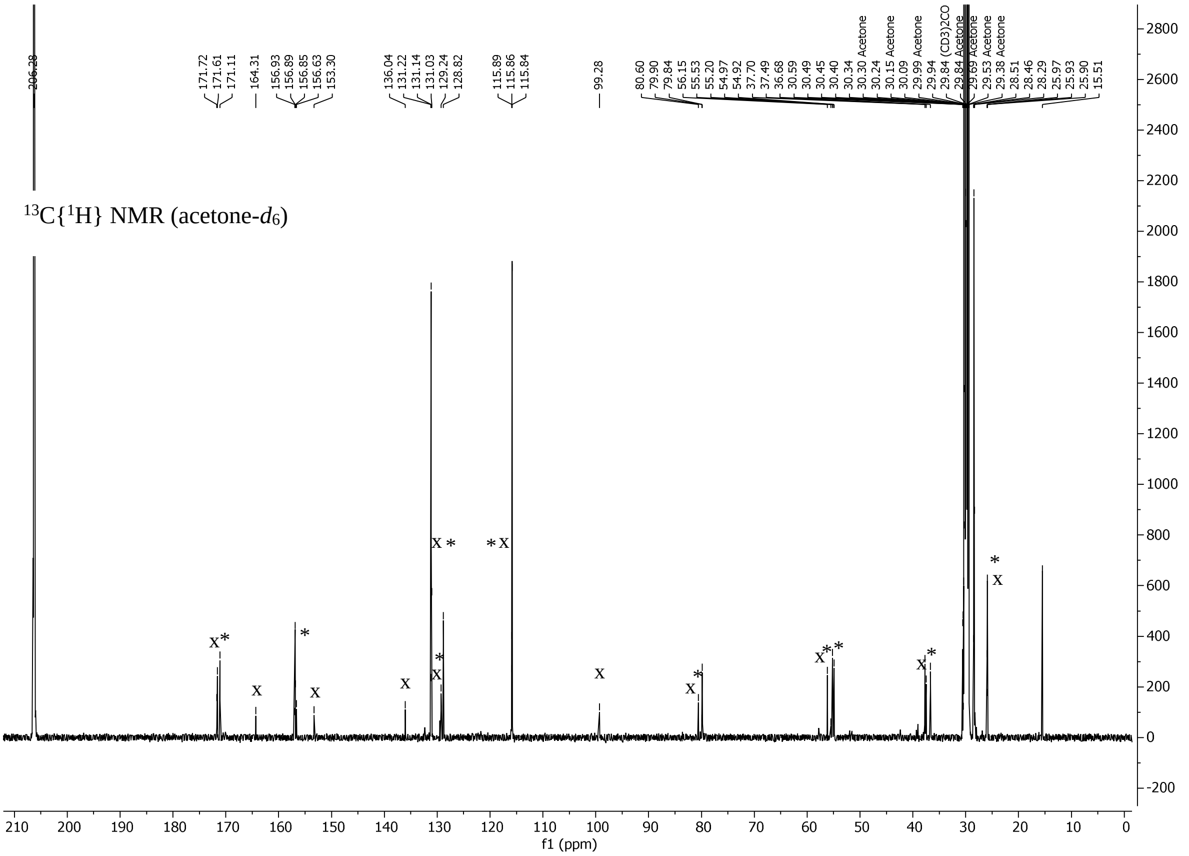

* indicates starting material ^{x} indicates side product **SI-11**

10.24 Compound 38 via methyl triflate

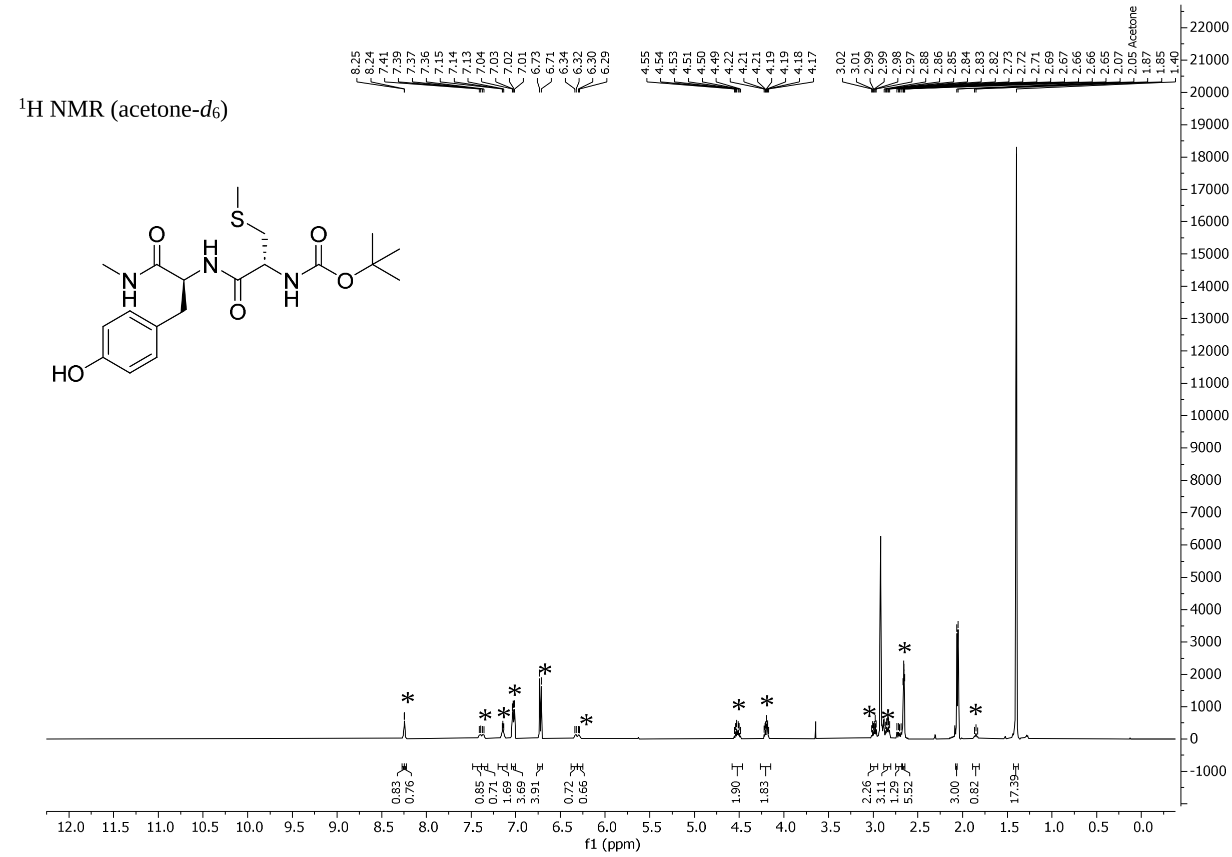

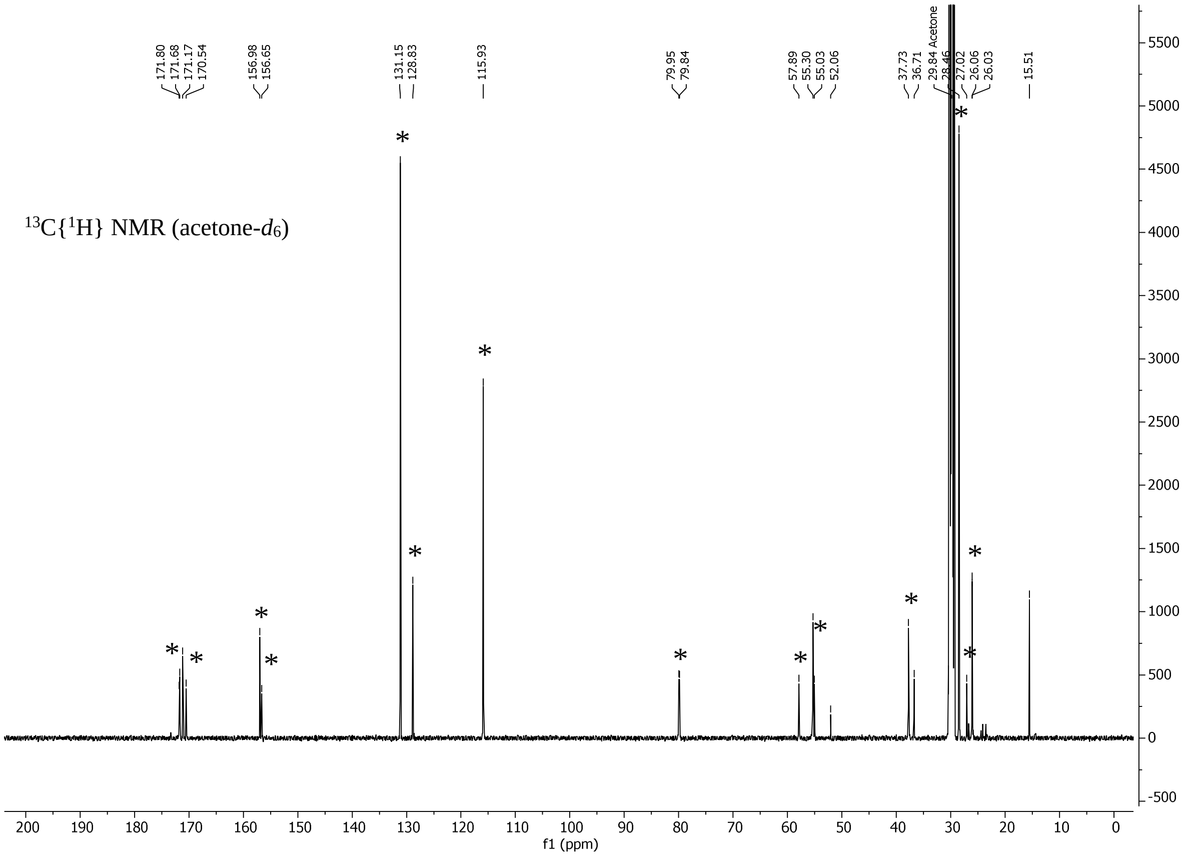

* indicates starting material

10.25 Compound 39

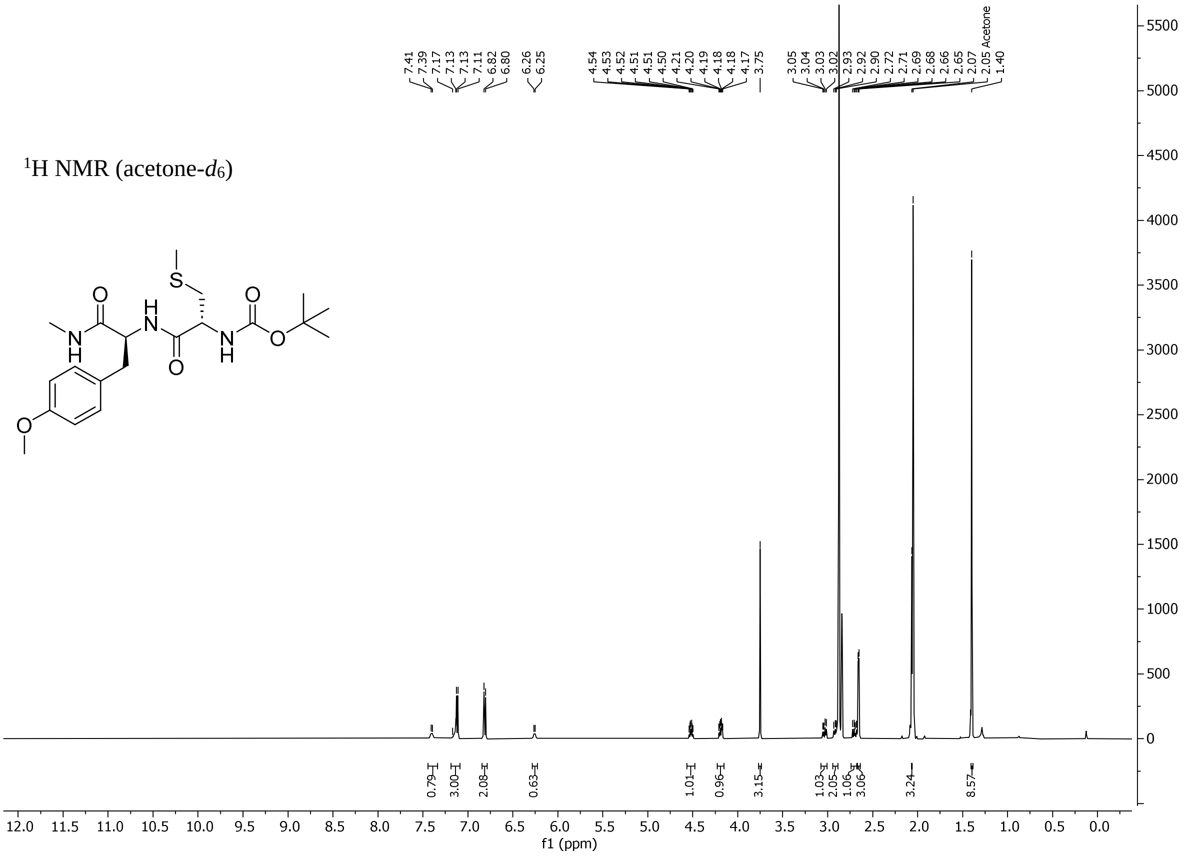

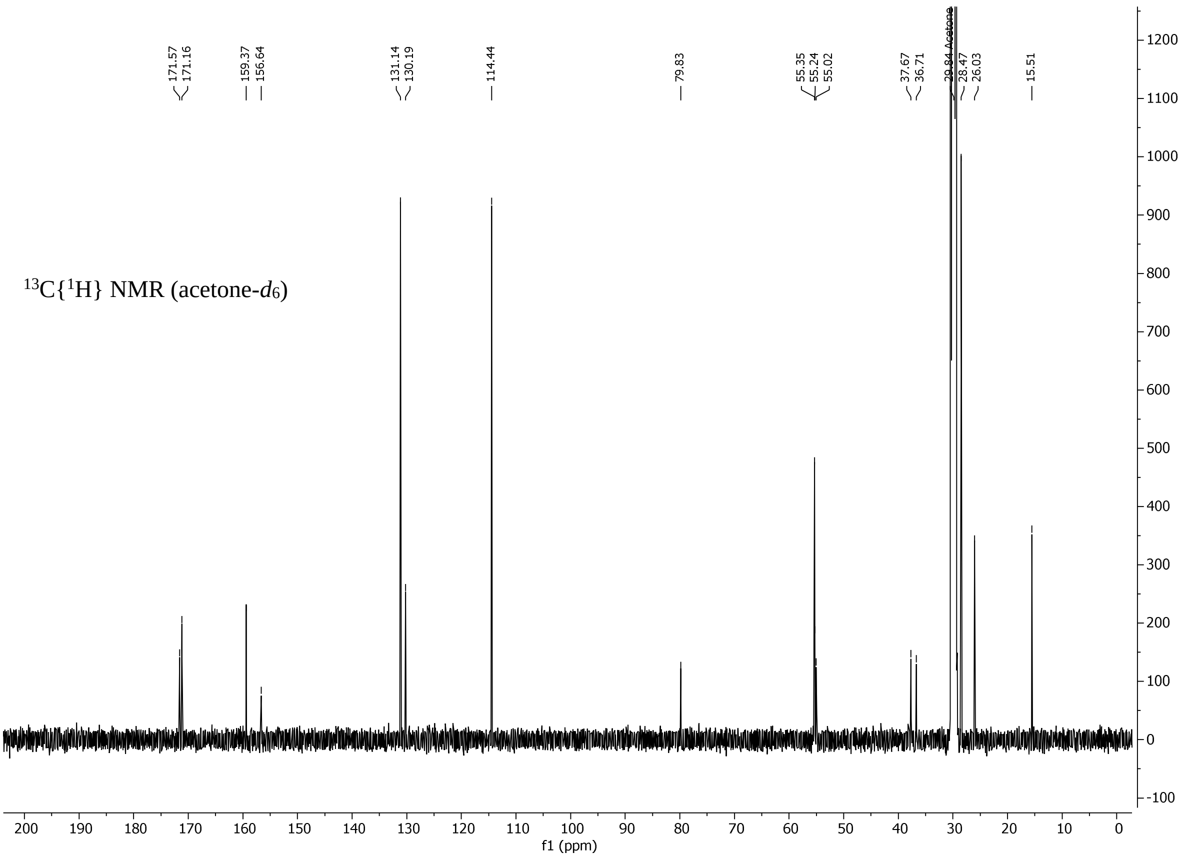

10.26 Compound 40

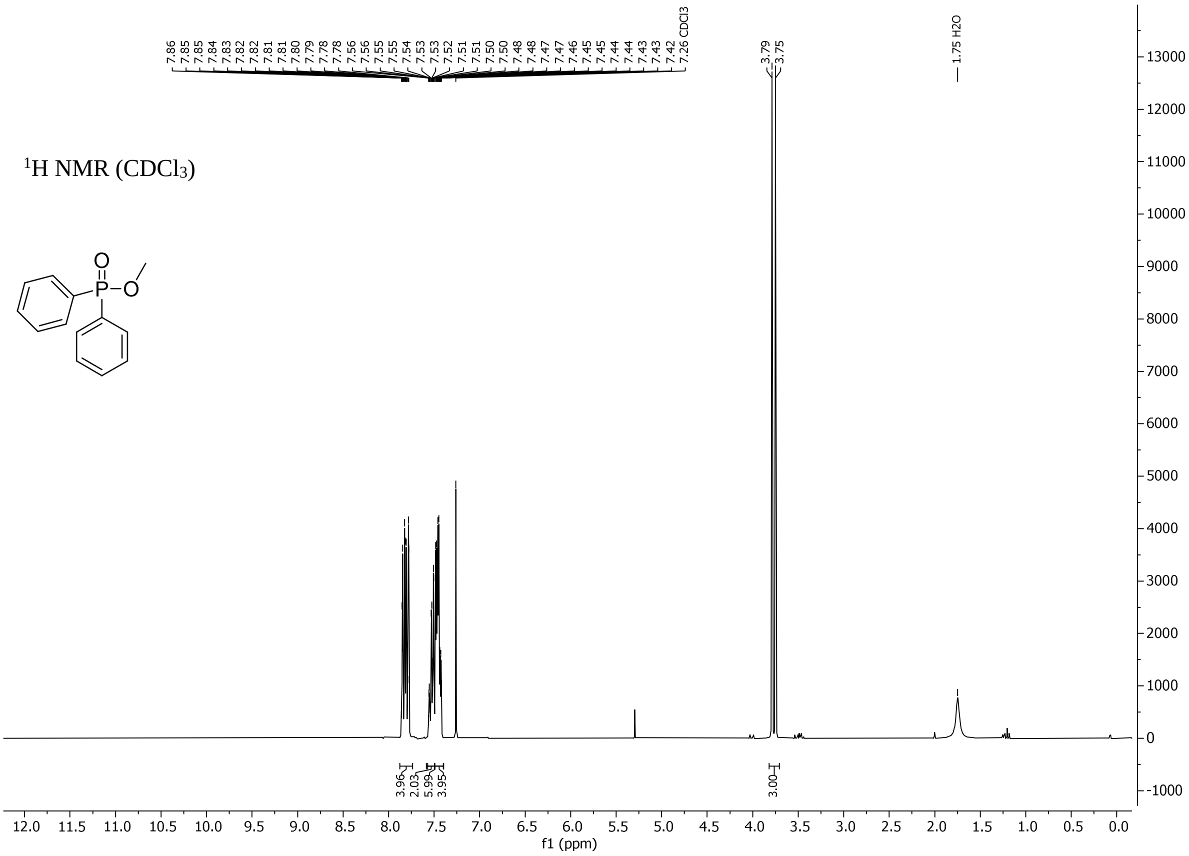

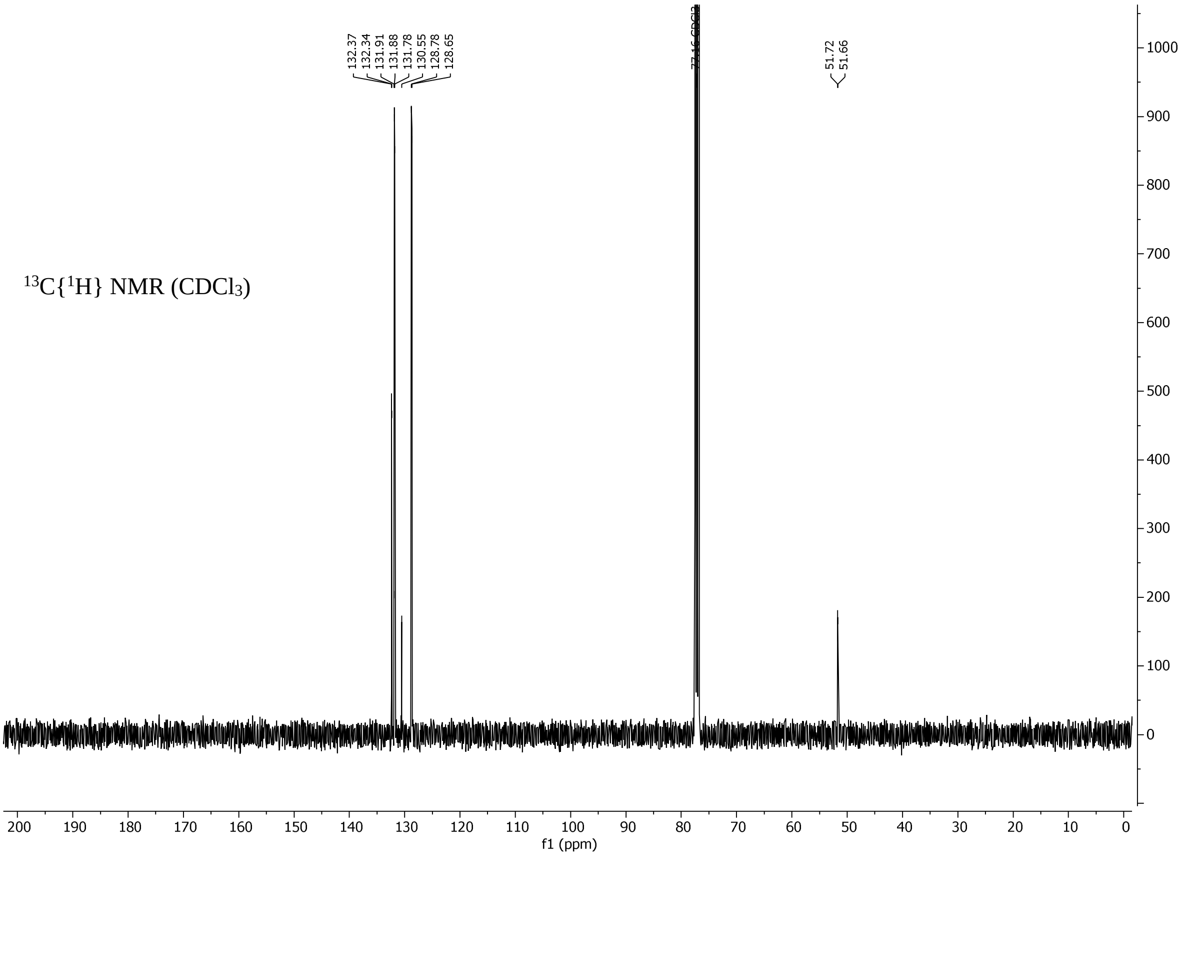

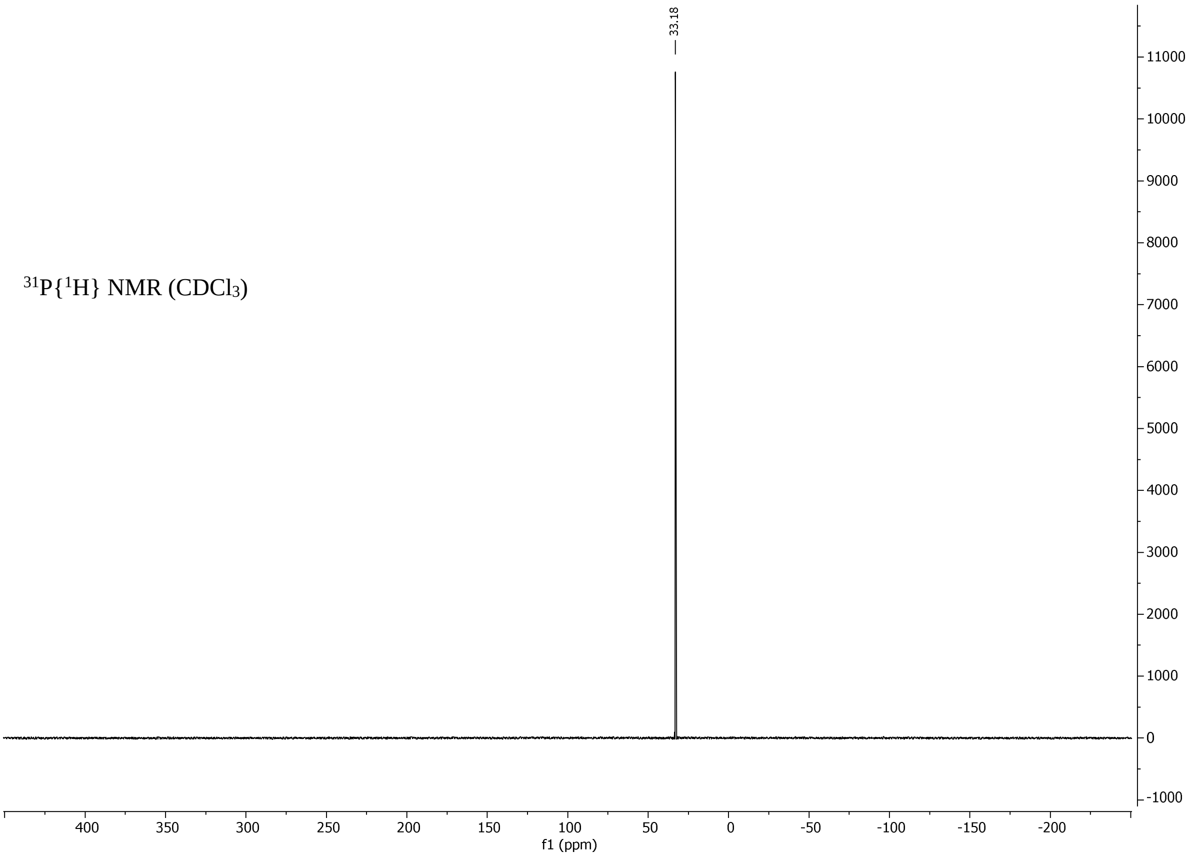

33.18
$^{31}P\{^{1}H\}$ NMR ($CDCl_3$)
11000
10000
9000
8000
7000
6000
5000
4000
3000
2000
1000
0
-1000
400
350
300
250
200
150
100
50
0
-50
-100
-150
-200
f1 (ppm)

11. References

[17] J. X. Wang, M. Q. Chen, Y. Zhang, B. Han, Z. D. Mou, X. Feng, X. Zhang, D. Niu, “A Modified Arbuzov-Michalis Reaction for Selective Alkylation of Nucleophiles” *Angew. Chem. Int. Ed.* **2024**, *63*, e202409931.

[24] T. Sakai, T. Kumoi, T. Ishikawa, T. Nitta, H. Iida, “Comparison of riboflavin-derived flavinium salts applied to catalytic H_2O_2 oxidations” *Org. Biomol. Chem.* **2018**, *16*, 3999–4007.

[25] S. Murahashi, D. Zhang, H. Iida, T. Miyawaki, M. Uenaka, K. Murano, K. Meguro, "Flavin-catalyzed aerobic oxidation of sulfides and thiols with formic acid/triethylamine" *Chem. Commun.* **2014**, *50*, 10295–10298.

[26] M. Bürchner, A. M. Erle, H. Scherer, I. Krossing, “Synthesis and Characterization of Boranate Ionic Liquids (BILs)” *Chem. Eur. J.* **2012**, *18*, 2254–2262.

[36] G. Heinrich, M. Kondratiuk, L. J. Goossen, M. P. Wiesenfeldt, "Rapid reaction optimization by robust and economical quantitative benchtop ^{19}F NMR spectroscopy" *Nat. Protoc.* **2024**, *19*, 1529–1556.

[37] A. Walter, W. Eisenreich, G. Storch, "Photochemical Desaturation and Epoxidation with Oxygen by Sequential Flavin Catalysis" *Angew. Chem. Int. Ed.* **2023**, *62*, e202310634.

[38] X. Ma, X. Yan, J. Yu, J. Guo, J. Bian, R. Yan, Q. Xu, L.-B. Han, "Metal-free catalytic nucleophilic substitution of primary alcohols with secondary phosphine oxides" *Green Chem.* **2025**, *27*, 102–108.

[39] K. S. Colle, E. S. Lewis, "Methoxyphosphonium ions; intermediates in the Arbuzov reaction" *J. Org. Chem.* **1978**, *43*, 571–574.

[40] A. Le Roch, M. Hébert, A. Gagnon, "Copper-Promoted O-Arylation of the Phenol Side Chain of Tyrosine Using Triarylbismuthines" *Eur. J. Org. Chem.* **2020**, *2020*, 5363–5367.

[41] I. Borthakur, S. Srivastava, S. Kumari, S. Kundu, "Tandem synthesis of *N*-methylated tertiary amines *via* three-component coupling of carbonyl compounds, amines, and methanol" *Chem. Commun.* **2022**, *58*, 9822–9825.

[42] A. Biswas, S. Kolb, S. H. Rottger, A. Das, L. J. Patalag, P. P. Dey, S. Sil, S. Maji, S. Chakraborty, O. S. Wenger, A. Bhunia, D. B. Werz, S. K. Mandal, "A BOIMPY Dye Enables Multi-Photoinduced Electron Transfer Catalysis: Reaching Super-Reducing Properties" *Angew. Chem. Int. Ed.* **2025**, *64*, e202416472.

[43] J. García-Méndez, A. López-Torres, M. A. Fernández-Herrera, "Improved synthesis and characterization of bile acid esters: Organogelation and supramolecular properties" *Steroids* **2025**, *214*, 109560.

[44] L. F. Toneto Novaes, C. Martins Avila, K. J. Pelizzaro-Rocha, D. B. Vendramini-Costa, M. Pereira Dias, D. B. Barbosa Trivella, J. Ernesto de Carvalho, C. V. Ferreira-Halder, R. A. Pilli, "(−)-Tarchonanthuslactone: Design of New Analogues, Evaluation of their Antiproliferative Activity on Cancer Cell Lines, and Preliminary Mechanistic Studies" *ChemMedChem* **2015**, *10*, 1687–1699.

[45] S. Malik, U. K. Nadir, P. S. Pandey, "Microwave-Assisted Efficient Methylation of Alkyl and Arenesulfonamides with Trimethylsulfoxonium Iodide and KOH" *Synth. Commun.* **2008**, *38*, 3074–3081.

[46] X.-Y. Jin, Y. M. He, T. H. Hui, L. Liu, L. Cheng, "Selective Methylation of Nucleosides via an *In Situ* Generated Methyl Oxonium" *J. Org. Chem.* **2024**, *89*, 3597–3604.

[47] F. Ishikawa, N. Tsukumo, E. Morishita, S. Asamizu, S. Kusuhara, S. Marumoto, K. Takashima, H. Onaka, G. Tanabe, "Biosynthetic diversification of non-ribosomal peptides through activity-based protein profiling of adenylation domains" *Chem. Commun.* **2023**, *59*, 9473–9476.

[48] J. Sitkowski, L. Stefaniak, L. Nicol, M. L. Martin, G. J. Martin, G. A. Webb, " Complete assignments of the ^{1}H, ^{13}C and ^{15}N NMR spectra of caffeine" *Spectrochim. Acta A Mol. Biomol. Spectrosc.* **1995**, *51*, 839–841.

[49] Z. Sun, F. He, Y. Xu, M. Lu, H. Xiong, Z. Jiang, C. Wu, " Intramolecular Palladium(II)-Catalyzed Regioselective 6-*endo* or 6-*exo* C–H Benzannulation: An Approach for the Diversity-Oriented Synthesis of Quinolinone Derivatives from Pyridones" *J. Org. Chem.* **2024**, *89*, 7058–7064.

[50] Y. Naganawa, K. Sakamoto, A. Fujita, K. Morimoto, M. Ratanasak, J. Y. Hasegawa, M. Yoshida, K. Sato, Y. Nakajima, "One-Step Esterification of Phosphoric, Phosphonic and Phosphinic Acids with Organosilicates: Phosphorus Chemical Recycling of Sewage Waste" *Angew. Chem. Int. Ed.* **2025**, *64*, e202416487.

[51] *APEX4 Suite of Crystallographic Software, Version 2021-10.0*, Bruker AXS Inc., Madison, Wisconsin, USA, **2021**.

[52] Bruker, *SAINT, V8.40B*, Bruker AXS Inc., Madison, Wisconsin, USA.

[53] L. Krause, R. Herbst-Irmer, G. M. Sheldrick, D. Stalke, "Comparison of silver and molybdenum microfocus X-ray sources for single-crystal structure determination" *J. Appl. Cryst.* **2015**, *48*, 3–10.

[54] G. M. Sheldrick, "SHELXT – Integrated space-group and crystal-structure determination" *Acta Cryst.* **2015**, *A71*, 3–8.

[55] G. M. Sheldrick, "Crystal structure refinement with *SHELXL*" *Acta Cryst.* **2015**, *C71*, 3–8.

[56] C. B. Hübschle, G. M. Sheldrick, B. Dittrich, "*ShelXle*: a Qt graphical user interface for *SHELXL*" *J. Appl. Cryst.* **2011**, *44*, 1281–1284.

[57] Ed. E. Prince, *International Tables for Crystallography Volume C, Mathematical, Physical and Chemical Tables,* International Union of Crystallography, Chester, England, **2006**, 500–502; 219–222; 193–199.

[58] C. R. Groom, I. J. Bruno, M. P. Lightfoot, S. C. Ward, "The Cambridge Structural Database" *Acta Cryst.* **2016**, *B72*, 171–179.

[59] D. Kratzert, *FinalCif, V144,* https://dkratzert.de/finalcif.html.
